# Polymerization of Allenes by Using an Iron(II) β‐Diketiminate Pre‐Catalyst to Generate High *M*_n_ Polymers

**DOI:** 10.1002/chem.202101078

**Published:** 2021-07-16

**Authors:** Callum R. Woof, Derek J. Durand, Ruth L. Webster

**Affiliations:** ^1^ Department of Chemistry University of Bath Claverton Down Bath BA2 7AY UK; ^2^ School of Chemistry University of Bristol Cantock's Close Bristol BS8 1TS UK

**Keywords:** allenes, cycloaddition, homogeneous catalysis, iron, polymers

## Abstract

Herein, we report an iron(II)‐catalyzed polymerization of arylallenes. This reaction proceeds rapidly at room temperature in the presence of a hydride co‐catalyst to generate polymers of weight up to *M*_n_=189 000 Da. We have determined the polymer structure and chain length for a range of monomers through a combination of NMR, differential scanning calorimetry (DSC) and gel permeation chromatography (GPC) analysis. Mechanistically, we postulate that the co‐catalyst does not react to form an iron(II) hydride in situ, but instead the chain growth is proceeding via a reactive Fe(III) species. We have also performed kinetic and isotopic experiments to further our understanding. The formation of a highly unusual 1,3‐substituted cyclobutane side‐product is also investigated.

## Graphical abstract

**A rare example** of transition metal‐catalyzed polymerization of aryl allenes is presented. Reactions are mild and generate very high *M*_n_ polymers with narrow polydispersity by using a well‐defined Fe(II) pre‐catalyst and a hydride additive. [2+2] cycloaddition to form cyclobutanes is also viable using the same catalytic system.

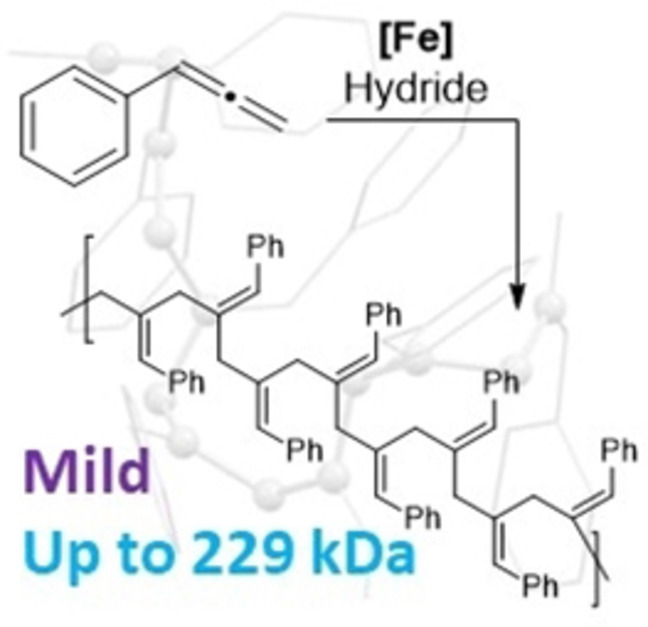

## Introduction

Allenes are emerging as a powerful and unique building block in molecular synthesis, owing to their facile reactivity, range of functionalities and unusual stereochemistry.^[1]^ There are many reported studies that use allenes as coupling partners in annulation and addition reactions,^[2]^ as building blocks in organic synthesis,^[3]^ and as structural features in molecular materials.^[4]^ In comparison to these, there are relatively few studies into polymerization reactions involving allenes, the first being by Wotiz using Co_2_(CO)_8_.^[5]^ These polymers have attracted interest due to their unusual structure, opportunities for further functionalization^[6]^ and development of complex molecular architecture.^[7]^ For example, Li has reported hyperbranched RAFT polymerization using allene‐derived monomers.^[8]^

**Scheme 1:**
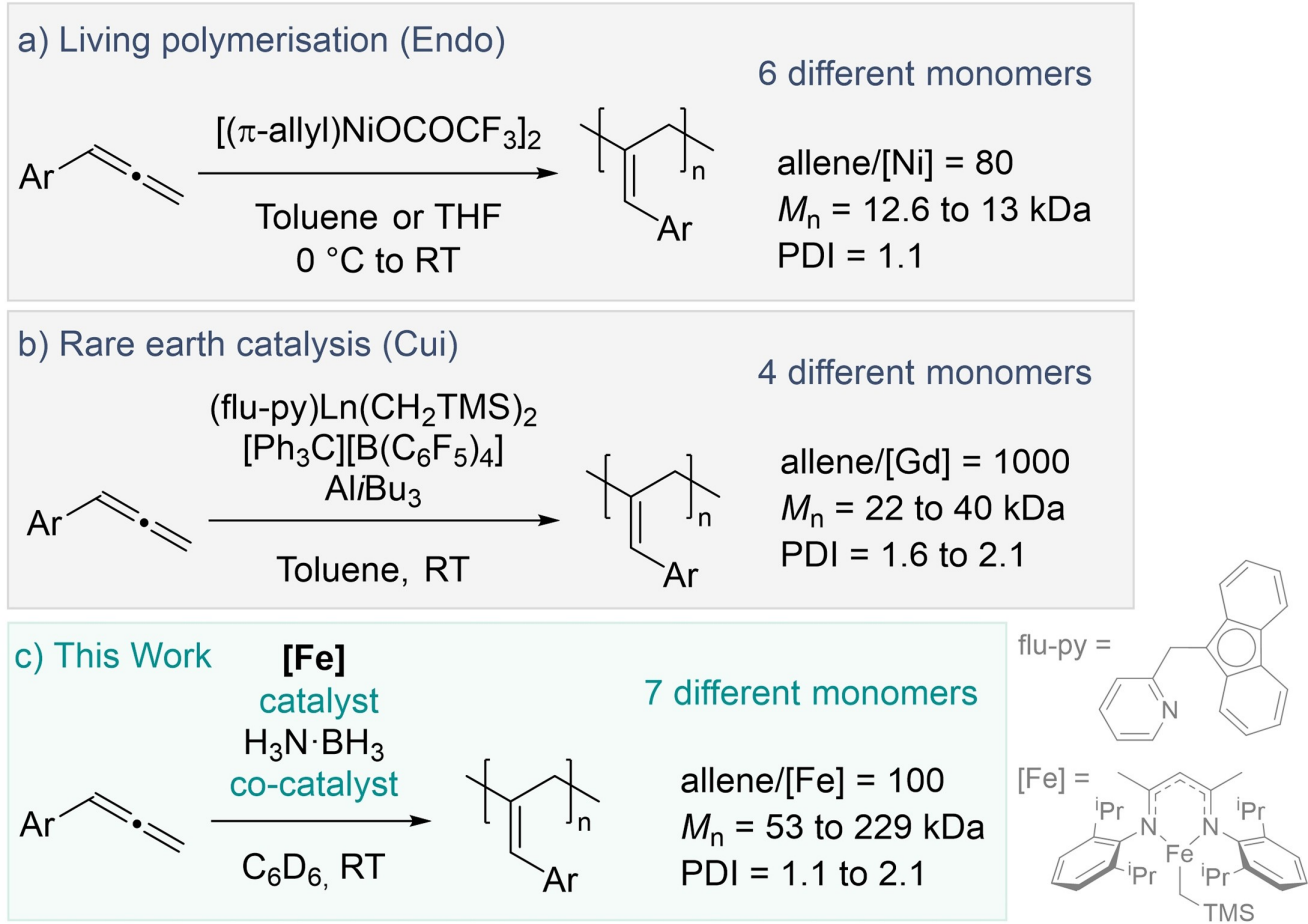
Examples of previously reported phenylallene polymerization catalysts, and the reaction reported herein.

Owing to its reactivity and ease of handling and synthesis, phenylallene has been used as a key model substrate for these reactions. Radical polymerization of phenylallene leading to low *M*_n_ and high Ð polymers were first reported by Anderson^[9]^ and further investigated by Takeshi.^[10]^ Barrett has reported that polymeric products are observed when phenylallene is treated with Grubbs catalyst,^[11]^ while Cui has demonstrated that rare‐earth metal catalysts can generate polymers that are considerably longer in chain length and has analyzed the physical properties of the polymers (Scheme 1).^[12]^ Endo and Tomita have also reported examples of living polymerization of phenylallene using a nickel catalyst,^[13]^ as well as multi‐component coupling polymerizations.^[14]^ In this study, we report the first polymerization of phenylallene using an iron pre‐catalyst and perform mechanistic and analytical studies to further understand how the reaction proceeds and investigate the physical properties of the polymers.

## Results and Discussion

We have reported numerous alkene functionalization reactions catalyzed by an iron(II) β‐diketiminate complex **[Fe]**,^[15]^ and were interested in applying our previously reported hydroboration chemistry^[16]^ to allene substrates. To our surprise, rather than undergoing an expected hydroboration reaction with phenylallene (**1**), we instead generate a polymeric substance as the primary product; iron β‐diketiminate catalysts have previously been shown to competent for some forms of coordination polymerization, for example for polylactide^[17]^ and polyethylene synthesis.^[18]^ Following our serendipitous discovery, we proceeded to optimize the reaction. Phenylallene polymerization proceeds with one equivalent of HBpin relative to substrate (Table 1, Entry 2) and with a co‐catalytic amount of HBpin (Table 1, Entry 3). Stoichiometric loading of HBpin leads to lower yield and lower *M*_n_ polymer (53 *versus* 152 kDa), presumably due to the propensity for chain‐terminating reactions with HBpin in solution. Other hydride sources, such as H_3_N⋅BH_3_, Me_2_HN⋅BH_3_ and HSi(OEt)_3_ also give high conversions (Table 1, Entries 4 to 6). H_3_N⋅BH_3_ gives the highest *M*_n_ polymer while maintaining narrow Ð (189 kDa, Ð = 1.19), which may be linked to both the lack of reactivity H_3_N⋅BH_3_ shows in dehydropolymerization chemistry,^[15c]^ therefore chain termination or side‐reactions are minimized, along with the potential role of H_3_N⋅BH_3_ in pre‐catalyst activation (see below). ^1^H and ^13^C{^1^H}^[19]^ NMR analysis indicate the terminal (2,3)‐substituted polymer (**P1_2,3_**) is the primary structural product.

**Table 1:** Effect of hydride source on polymerization.

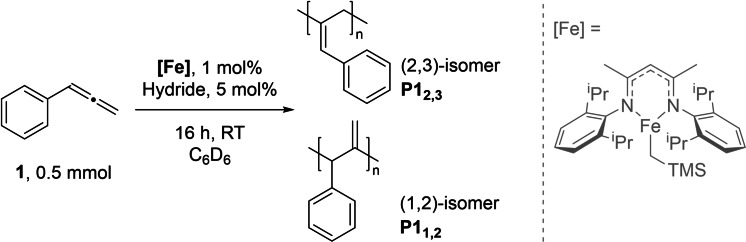						
Entry	Hydride	Conversion [%]	*M*_n_ [kDa]	Ð	**P1_2,3_** : **P1_1,2_**	DP
1	None	3	–	–	–	–
2^[a]^	HBpin	82	53	1.12	8 : 1	460
3	HBpin	91	152	1.36	5 : 1	1310
4	H_3_N⋅BH_3_	93	189	1.19	7 : 1	1630
5	Me_2_HN⋅BH_3_	89	10.5	6.25	5 : 1	91
6	HSi(OEt)_3_	92	62	1.18	6 : 1	530
7^[b]^	H_3_N⋅BH_3_	0	–	–	–	–

Conditions: 600 μL C_6_D_6_, 0.5 mmol **1**, 1 mol % **[Fe]**, 5 mol % hydride, 16 h, RT. Conversion determined by ^1^H NMR spectroscopy. [a] 1 eq. of HBpin relative to allene (0.5 mmol), 7 % conversion to hydroborated product observed. [b] No [Fe], 60 °C, 72 h.

**Figure 1:**
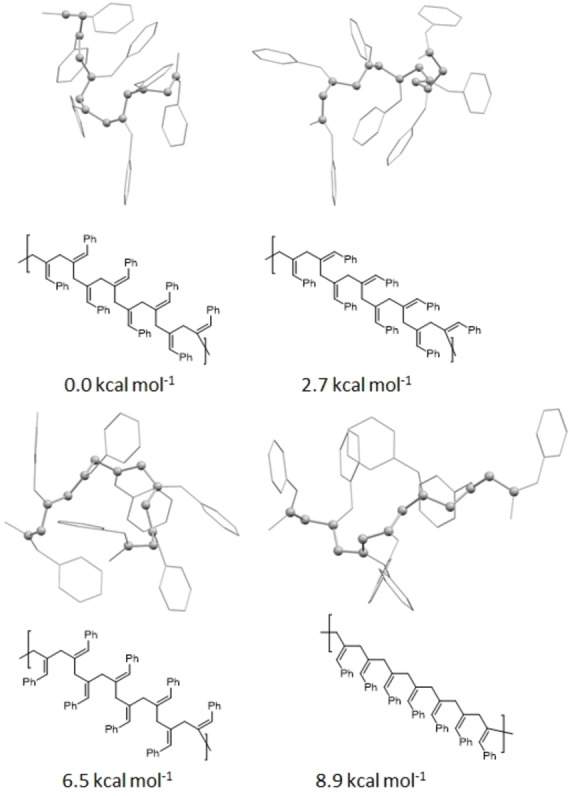
Oligomer structures optimized by DFT.

Investigating the structure of **P1_2,3_** in greater detail, the NMR spectra further show that there are three distinct proton environments (1.7 : 1.1 : 1 ratio) around the main chain, which we believe relates to the different conformations the polymer can take. NOE NMR spectroscopy indicates that these environments are conformationally distinct from each other (See Supporting Information). Diad structures would be expected to correlate through NOE and 2D NOESY NMR experiments. In both cases, no correlation is observed between the different methylene proton environments, leading to us to conclude that each proton is primarily adjacent to protons in the same environment. Although we cannot rule out a random arrangement of diads, based on the data we propose longer blocks of conformers. Using oligomers containing seven repeat units, Density Functional Theory (DFT) calculations (B3LYP‐D3/6‐31G* (SCF=Benzene), 298 K) support the notion that there are three distinct conformers present in the product (one major species and two minor species, which matches observations by NMR spectroscopy), with the ground state species (0.0 kcal mol^−1^), followed by a conformer at ΔG=+2.7 kcal mol^−1^ and one at ΔG=+6.5 kcal mol^−1^ (Figure 1). A fourth higher energy conformer appears at ΔG=+8.9 kcal mol^−1^.^[20]^ Population distributions calculated using Maxwell‐Boltzmann distribution further supports the experimental data; at raised temperatures (up to 80 °C) the signals remain distinct and do not appear to interconvert on an NMR timescale. Furthermore, heating the polymer for an extended length of time (80 °C, 3 days) does not lead to a change of intensities or other changes to the NMR spectrum (See Supporting Information). This appears to rule out structure interconversion.

As H_3_N⋅BH_3_ gives the highest conversion, *M*_n_, a narrow Ð and a reasonably high ratio of the two observed isomers (**P1_2,3_** and **P1_1,2_**), further reaction optimization was carried out using this hydride source (Table 2). Whilst there is relatively little difference between stirred and unstirred flasks (Table 2, Entry 2), there is a significant increase in *M*_n_ in more concentrated conditions, albeit at the expense of conversion to polymer (Table 2, Entries 3 and 4). In particular, solvent‐free conditions (Table 2, Entry 4) lead to a dramatic reduction in conversion, which we attribute to lack of stabilization of activated pre‐catalyst (see below). Raising the temperature has a modest effect on *M*_n_ but increases polydispersity significantly (Table 2, Entry 5). Quenching the reaction with methanol before full monomer conversion enables smaller molecular weight polymers to be isolated (Table 2, Entry 6). MALDI‐ToF analysis shows repeat units of 116 Da and end groups are proteo/allenyl in nature i. e. neither CH_2_SiMe_3_, BH_2_ nor NH_2_ fragments are incorporated into the polymer chain. Increasing the catalyst loading does not reduce the *M*_n_ (>250 kDa, Table 2, Entry 7), but as observed in Table 1, varying the quantity of hydride source does appear to give more controlled polymerization. The optimized conditions for the synthesis of polymer **P1** are **[Fe]** (1 mol %), H_3_N⋅BH_3_ (5 mol %), C_6_D_6_ (0.6 mL), RT, 16 h; the resultant 189 kDa polymer is around seven times that reported by Cui^[12]^ and over twenty times that reported by Endo.^[13a]^ Importantly, polymerization catalyzed by **[Fe]** is not at the expense of Ð (1.2 compared to 1.6–2.1 and 1.1 from Cui and Endo respectively).

**Table 2:** Variation of reaction conditions.

Entry	Deviation from standard conditions	Conversion [%]	*M*_n_ [kDa]	Ð
1	none	93	189	1.19
2	no stirring	91	83.9	1.38
3^[a]^	100 μl solvent	92	–	–
4^[a]^	no solvent	71	12.6	1.75
5^[b]^	heated at 80 °C	96	132	1.85
6	quenched after 5 minutes	19	10.5	1.33
7^[a]^	20 mol % catalyst loading	96	–	–

Conversion determined by ^1^H NMR spectroscopy. [a] Under these conditions, polymer length was greater than 250 kDa and beyond the resolution of the GPC column, data reported in Supporting Information. ^1^H NMR indicates the same polymer structure is forming. [b] 90 % polymer, 6 % dimer, see below for further discussion.

DSC analysis of the resultant polyphenylallene (**P1**) indicates a *T*_g_ of 64.8 °C, which is similar to the range reported by Cui (*T*_g_=61.3–61.8 °C).^[12]^ We do not observe any melting transitions, either through DSC analysis or visual observations, indicating that the **P1** is amorphous rather than crystalline. The structural nature of the polymer was investigated using WAXD studies (See Supporting Information). Analysis of a polymer fiber gave no Bragg peaks or 2D orientation, indicating that the polymer generated is amorphous.

We have applied our optimized polymerization conditions to several arylallene substrates (Table 3). All substrates lead to high conversions, high *M*_n_ and narrow Ð, although the reaction with *para*‐fluoro reagent **6** and *para*‐chloro **7** (Table 3, Entries 5 and 6) require higher catalyst loading and have lower conversion. The *M*_n_ for **P6** is particularly low compared to the other polymers produced. As expected, a larger *M*_n_ tends to lead to a wider Ð (Table 3, Entries 1, 4 and 6), whereas a low *M*_n_ gives a narrow Ð (e. g. Table 3, Entries 2 and 5). It is difficult to link the **P_2,3_** : **P_1,2_** ratio to *M*_n_ or Ð, for example, it could be assumed that reactions that have less control over polymerization and give high *M*_n_ and broad Ð may also be less selective, but this is not necessarily the case here (compare Table 3, Entries 4 and 5). *Ortho*‐methyl substrate **3** gives poor **P_2,3_** : **P_1,2_** ratio, which may be linked to a lack of isomer selectivity due to steric interference. In all cases there are distinct chain proton environments with similar NOE correlating peaks as observed with **P1** (See Supporting Information). We do not observe any polymerization occurring when using non‐aryl allene substrates, such as cyclohexylallene or methoxyallene. Presumably aryl substituents provide the electronic effects necessary to enable reactivity to proceed and has been highlighted by other researchers investigating the reactivity and functionalization of allenes.^[21]^ Furthermore, although disappointing, the lack of reactivity with non‐aryl allenes is not surprising given previous reports using the pre‐catalyst, which has been dominated by functionalization of aryl substrates[^15c^, ^22^] or intramolecular cyclizations.[^15a^, ^23^]

**Table 3:** Scope and properties of functionalized arylallene polymers.^[a]^

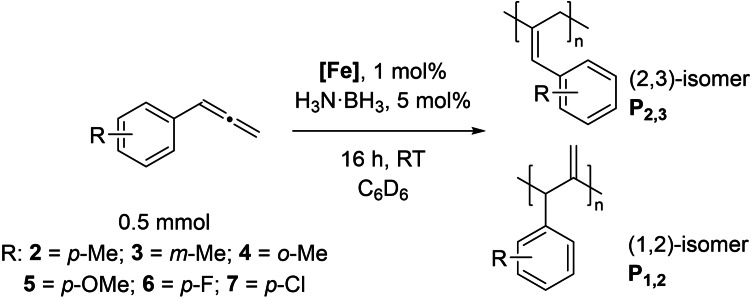								
Entry	Major isomer		Conversion [%]	**P_2,3_** : **P_1,2_**	*M*_n_ [kDa]	Ð	*DP*	*T*_g_ [°C]
1	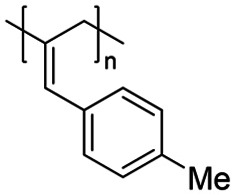	**P2_2,3_**	94	8 : 1	229	1.63	1760	86.4
2	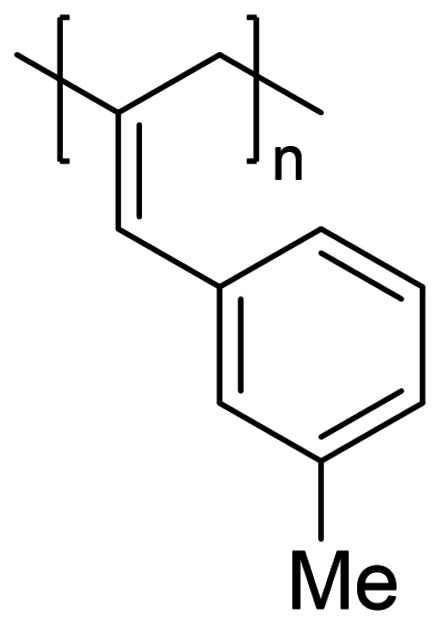	**P3_2,3_**	96	7 : 1	72	1.17	550	63.0
3	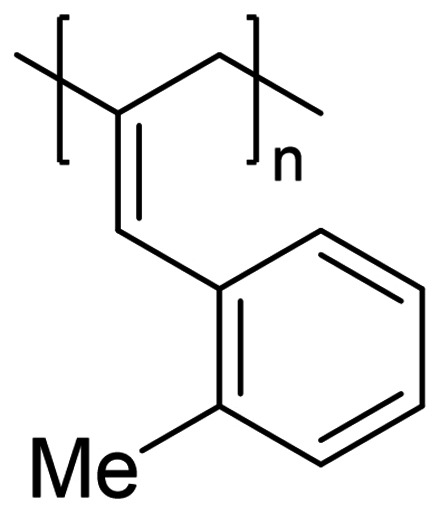	**P4_2,3_**	91	5 : 1	185	1.62	1420	75.4
4	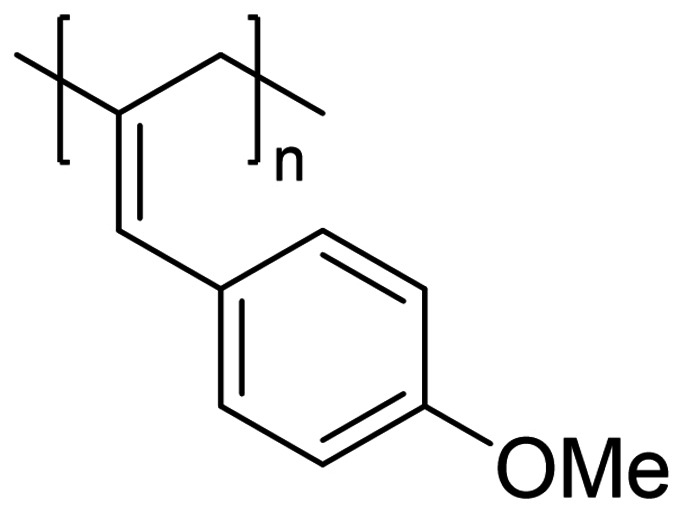	**P5_2,3_**	98	11 : 1	174	2.05	1190	81.6
5^[b]^	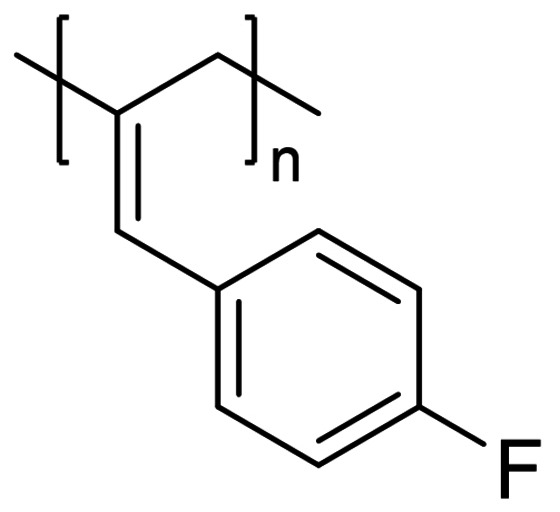	**P6_2,3_**	77	13 : 1	53	1.12	400	57.0
6^[b]^	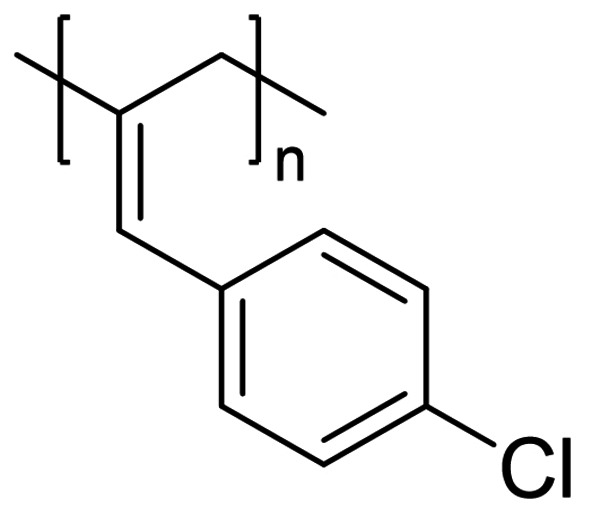	**P7_2,3_**	74	6 : 1	166	1.67	1110	97.1

[a] Conditions: 600 μL C_6_D_6_, 0.5 mmol allene, 1 mol % [Fe], 5 mol % H_3_N⋅BH_3_, 16 h, RT. Conversion determined by ^1^H NMR spectroscopy. [b] 5 mol % [Fe].

**Scheme 2:**
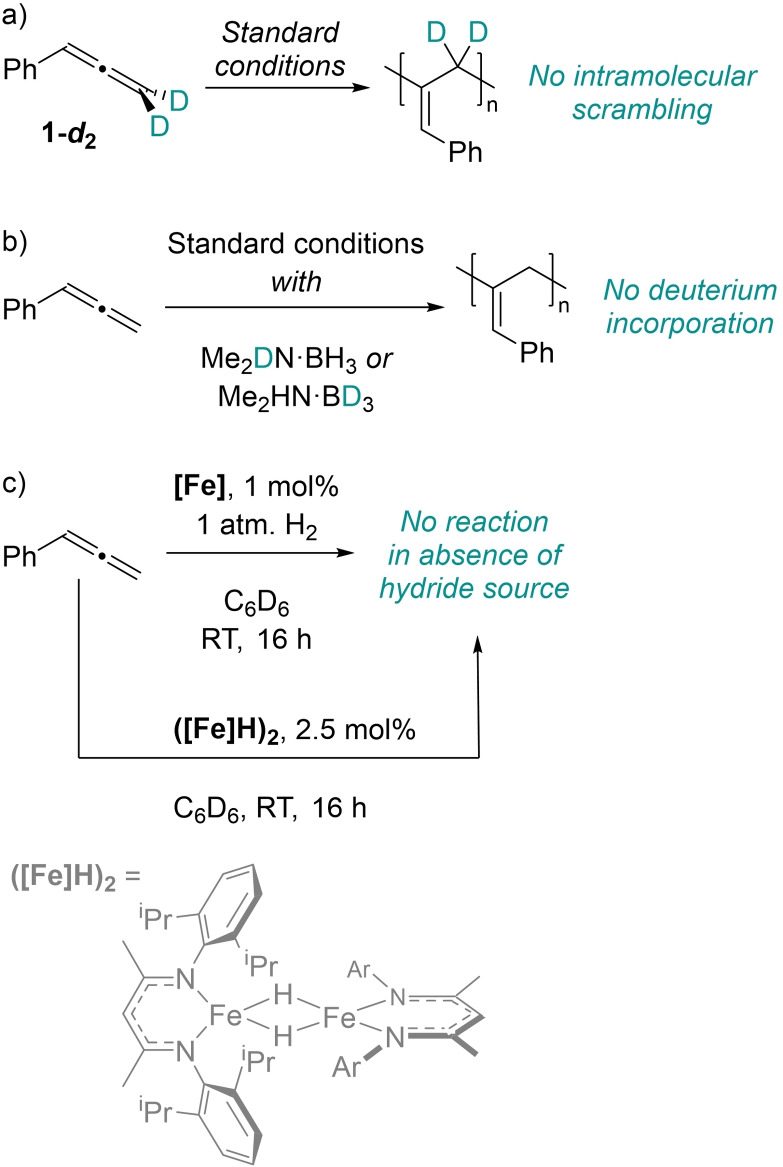
Mechanistic studies.

**Scheme 3:**
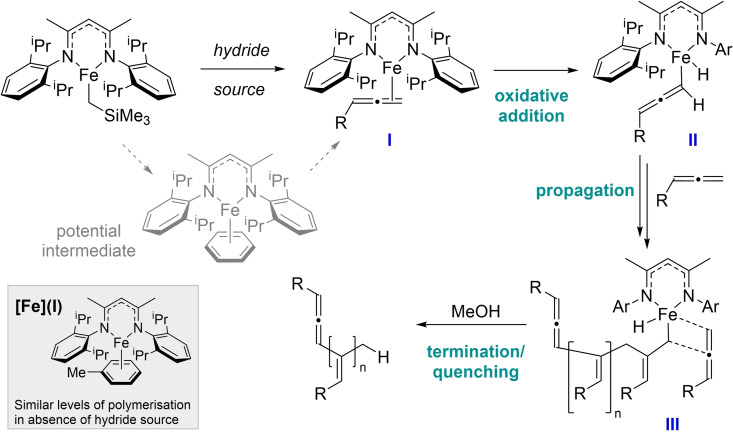
Postulated catalyst activation step and subsequent polymerization process.

To understand the nature of the polymerization, some mechanistic investigations were performed. Kinetic analysis indicates the reaction is first order with respect to the catalyst, and PMe_3_ poisoning experiments indicate the reaction is not nanoparticle mediated. Addition of a radical clock does not significantly alter rate. Application of deuterium‐labelled phenylallene (**1‐*d***_**2**_, Scheme 2a) in polymerization under standard conditions leads to clean isotopic retention in the product, as indicated by protic signals at 3.46 ppm (methylene protons) that integrate to the quantity of residual protons in **1‐*d***_**2**_, with the protic signal at 6.24 ppm (α proton) integrating to 1H. If scrambling had occurred we would expect the 6.24 ppm integral to be less than 1H. Furthermore, the ^2^H NMR spectrum, albeit weak, only shows peaks at 3.70‐3.45 ppm. Using deuterated hydride sources, such as Me_2_DN⋅BH_3_ or Me_2_HN⋅BD_3_, and quenching the reaction to ensure low *M*_n_ polymer is formed, give no apparent D‐incorporation in the polymer (Scheme 2b). We have previously reported that the bridged iron hydride dimer **([Fe]H)_2_** is an on‐cycle species some dehydrocoupling reactions.^[24]^ However, **([Fe]H)_2_** is a poor catalyst for this transformation (5 % conversion, 16 h, RT) in the absence of H_3_N⋅BH_3_. Performing the reaction with **[Fe]** under a hydrogen atmosphere does not lead to reasonable conversion; consequently, we do not believe this particular iron hydride dimer is an active catalyst. We have recently reported evidence for Fe(I) species forming from the reaction of **[Fe]** with the same hydride sources employed in this reaction. In this previous study, DFT studies supported the reaction proceeding via oxidative addition of C−H bonds to generate Fe(III) intermediates.^[25]^ In light of this, we postulate that the polymerization proceeds in a similar manner (Scheme 3): an initial pre‐catalyst activation that involves reduction to an Fe(I) species, **I**. We anticipate that formation of **I** proceeds via an η^6^‐arene stabilized intermediate, the formation of which is necessary to efficiently access **I** (supported by the drop‐off in conversion in the absence of solvent, Table 2). The η^2^‐supported allene then undergoes oxidative addition of a terminal C−H bond (**II**), consistent with no deuterium incorporation when using Me_2_HN⋅BD_3_, followed by insertion of monomer into the Fe−C bond (**III**), which is the key chain propagation process. Given the high degree of polymerization (*DP*) values across all substrates and conditions, it is likely that only a relative small amount of metal centers are active species. *In situ* wide sweep NMR spectroscopy confirms that a significant amount of pre‐catalyst remains unreacted even after full conversion of the substrate. Preparation of an analogous Fe(I)‐η^6^‐arene species, **[Fe](I)**, leads to similar reactivity without a hydride source present (90 % conversion to **P1**; 6 : 1 ratio **P1_2,3_** : **P1_1,2_**, *M*_n_=190 kDa, Ð=1.51) supporting our hypothesis.

**Scheme 4:**
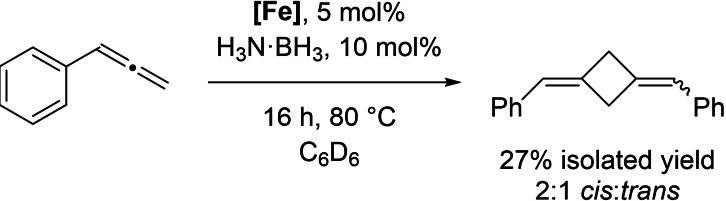
[2+2] cycloaddition of phenylallene.

Interestingly, when the polymerization is performed at 80 °C, an unusual side‐reaction is observed (Scheme 4). 1,3‐di((*Z*/*E*)‐benzylidene)cyclobutane can also be formed in modest yield in a 2 : 1 ratio of *cis* : *trans*. The product is readily isolable from the polymer residue (See Supporting Information). Attempts to increase the yield of this cyclobutane product using controlled, dilute addition using a syringe pump (e. g. using **1** in 670 μL C_6_H_6_ added at a rate of 20 μL/min to a flask containing 5 mol % H_3_N⋅BH_3_ and 1 mol % **[Fe]** in 6 mL C_6_H_6_) failed to give a substantial increase in yield, and polymerization still dominates. Undertaking this [2+2] cycloaddition with Me_2_DN⋅BH_3_ or Me_2_HN⋅BD_3_ does not lead to ^2^H incorporation in the product. These cyclobutane structures are unusual, as the thermal dimerization and [2+2] cycloadditions of allenes generally produce 1,2‐substituted rings as the major product.^[26]^ Furthermore these are challenging synthetic targets: to the best of our knowledge the only previous synthetic route to these molecules was reported by Dixneuf, which used a pinacolborane‐allene and Ru pre‐catalyst at 100 °C to generate the 1,3‐disubstituted cyclobutane product as a 1 : 2 ratio of *cis* : *trans* isomers i. e. complementary to our results.^[27]^

## Conclusion

In summary, by using an iron(II) pre‐catalyst we can polymerize phenylallene and its derivatives to form 2,3‐substituted polymers of substantial molecular weight. We have characterized these polymers by using a range of techniques and have postulated a mechanism based on our studies into the nature of the reaction. Work is ongoing to develop our polymerization chemistry with **1** further, as well as investigate its potency in other catalytic allene functionalization reactions.

## Conflict of interest

The authors declare no conflict of interest.

## Supporting information

As a service to our authors and readers, this journal provides supporting information supplied by the authors. Such materials are peer reviewed and may be re‐organized for online delivery, but are not copy‐edited or typeset. Technical support issues arising from supporting information (other than missing files) should be addressed to the authors.

Supporting Information

## References

[1a] S. R. Sandler, W. Karo, ALLENES, Academic Press 1992;

[1b] D. J. Pasto, Tetrahedron 1984, 40, 2805–2827.

[2a] S. Yu, S. Ma, Angew. Chem. Int. Ed. 2012, 51, 3074–3112; 10.1002/anie.201101460 22271630

[2b] S. Ma, Acc. Chem. Res. 2009, 42, 1679–1688; 19603781 10.1021/ar900153r

[2c] M. A. Tius, Chem. Soc. Rev. 2014, 43, 2979–3002. 24196585 10.1039/c3cs60333d

[3a] R. Zimmer, H.-U. Reissig, Chem. Soc. Rev. 2014, 43, 2888–2903; 24549322 10.1039/c3cs60429b

[3b] R. Zimmer, C. U. Dinesh, E. Nandanan, F. Ahmed Khan, Chem. Rev. 2000, 100, 3067–3126; 11749314 10.1021/cr9902796

[3c] L. Alessandro Perego, R. Blieck, A. Groué, F. Monnier, M. Taillefer, I. Ciofini, L. Grimaud, ACS Catal. 2017, 7, 4253–4264;

[3d] Y. Nagashima, K. Sasaki, T. Suto, T. Sato, N. Chida, Chem. Asian J. 2018, 13, 1024–1028; 29431908 10.1002/asia.201800134

[3e] L. Fensterbank, M. Malacria, Acc. Chem. Res. 2014, 47, 953–965. 24564512 10.1021/ar4002334

[4] P. Rivera-Fuentes, F. Diederich, Angew. Chem. Int. Ed. 2012, 51, 2818–2828; 10.1002/anie.201108001 22308109

[5] H. Greenfield, I. Wender, J. H. Wotiz, J. Org. Chem. 1956, 21, 875–878.

[6] K. Osakada, D. Takeuchi, Polymer Synthesis, Springer Berlin Heidelberg, Berlin, Heidelberg 2004, pp. 137–194.

[7] Y. Gao, D. Zhou, J. Lyu, S. A, Q. Xu, B. Newland, K. Matyjaszewski, H. Tai, W. Wang, Nat. Chem. Rev. 2020, 4, 194–212. 10.1038/s41570-020-0170-7 37128047

[8] Y. Bao, G. Shen, X. Liu, Y. Li, J. Polym. Sci. Part A 2013, 51, 2959–2969.

[9] J. Leland, J. Boucher, K. Anderson, J. Polym. Sci. Polym. Chem. Ed. 1977, 15, 2785–2788.

[10] T. Yokozawa, N. Ito, T. Endo, Chem. Lett. 1988, 17, 1955–1958.

[11] M. Ahmed, T. Arnauld, A. G. M. Barrett, D. Christopher Braddock, K. Flack, P. A. Procopiou, Org. Lett. 2000, 2, 551–553. 10814374 10.1021/ol0055061

[12] F. Lin, Z. Liu, T. Wang, D. Cui, Angew. Chem. Int. Ed. 2017, 56, 14653–14657; 10.1002/anie.201707601 28925534

[13a] K. Takagi, I. Tomita, T. Endo, Macromolecules 1997, 30, 7386–7390;

[13b] A. Yamauchi, A. Shirai, K. Kawabe, T. Iwamoto, T. Wakiya, H. Nishiyama, S. Inagi, I. Tomita, NPG Asia Mater. 2016, 8, e307–e307.

[14] K. Nakagawa, I. Tomita, Macromolecules 2007, 40, 9212–9216.

[15a] M. Espinal-Viguri, A. K. King, J. P. Lowe, M. F. Mahon, R. L. Webster, ACS Catal. 2016, 6, 7892–7897;

[15b] M. Espinal-Viguri, S. E. Neale, N. T. Coles, S. A. Macgregor, R. L. Webster, J. Am. Chem. Soc. 2019, 141, 572–582; 30518206 10.1021/jacs.8b11553

[15c] N. T. Coles, M. F. Mahon, R. L. Webster, Organometallics 2017, 36, 2262–2268.

[16] M. Espinal-Viguri, C. R. Woof, R. L. Webster, Chem. Eur. J. 2016, 22, 11605–11608. 27321704 10.1002/chem.201602818

[17] V. C. Gibson, E. L. Marshall, D. Navarro-Llobet, A. J. P. White, D. J. Williams, J. Chem. Soc. Dalton Trans. 2002, 4321–4322.

[18] M.-S. Zhou, S.-P. Huang, L.-H. Weng, W.-H. Sun, D.-S. Liu, J. Organomet. Chem. 2003, 665, 237–245.

[19] X. Tao, C. G. Daniliuc, K. Soloviova, C. A. Strassert, G. Kehr, G. Erker, Chem. Commun. 2019, 55, 10166–10169. 10.1039/c9cc04199k 31389927

[20] We also tentatively predict the presence of an atactic polymer. However we cannot be confident of the relative stability of the atactic species (+6.1 kcal mol^-1^) beyond the seven-unit oligomer, as this would not remain constant compared to the other species for longer chain lengths.

[21a] H.-Q. Geng, J.-B. Peng, X.-F. Wu, Org. Lett. 2019, 21, 8215–8218; 31565941 10.1021/acs.orglett.9b02925

[21b] X. Ma, Z. Yu, T. Liu, Comput. Theor. Chem. 2020, 1190, 113013.

[22a] A. K. King, K. J. Gallagher, M. F. Mahon, R. L. Webster, Chem. Eur. J. 2017, 23, 9039–9043; 28544315 10.1002/chem.201702374

[22b] A. K. King, A. Buchard, M. F. Mahon, R. L. Webster, Chem. Eur. J. 2015, 21, 15960–15963. 26406999 10.1002/chem.201503399

[23] E. Bernoud, P. Oulié, R. Guillot, M. Mellah, J. Hannedouche, Angew. Chem. Int. Ed. 2014, 53, 4930–4934; 10.1002/anie.201402089 24692368

[24] D. Gasperini, A. K. King, N. T. Coles, M. F. Mahon, R. L. Webster, ACS Catal. 2020, 10, 6102–6112.

[25] C. R. Woof, D. J. Durand, N. Fey, E. Richards, R. L. Webster, Chem. Eur. J. 2021, 27, 5972–5977. 33492679 10.1002/chem.202004980 PMC8048803

[26] B. Alcaide, P. Almendros, C. Aragoncillo, Chem. Soc. Rev. 2010, 39, 783–816. 20111793 10.1039/b913749a

[27] E. Bustelo, C. Guérot, A. Hercouet, B. Carboni, L. Toupet, P. H. Dixneuf, J. Am. Chem. Soc. 2005, 127, 11582–11583. 16104717 10.1021/ja051930r

