# Supplementary material for: Polymerization of Allenes by Using an Iron(II) β‐Diketiminate Pre‐Catalyst to Generate High M n Polymers
Source: Chemistry. 2021 Jul 16;27(48):12335–40. doi: 10.1002/chem.202101078 (PMC8457186; doi:10.1002/chem.202101078)
Supplement: Supplementary file 1 — Supporting Information [file CHEM-27-12335-s001.pdf]

Chemistry–A European Journal

Supporting Information

Polymerization of Allenes by Using an Iron(II) β-Diketiminate Pre-Catalyst to Generate High M_n Polymers

Callum R. Woof, Derek J. Durand, and Ruth L. Webster*

Contents

General Experimental Details & Catalyst Synthesis 2

Synthesis of Allenes 5

Synthesis of Deuterated Reagents 6

NMR Characterisation of Polymers 9

WAXD Studies of Polyphenylallene 14

MALDI Studies of Polyphenylallene 15

Optimisation and Characterisation of Dimeric Species 16

Characterisation of Allenes & Precursors 18

NMR Spectra, GPC Data Reports, DSC Traces 23

- Allenes & Precursors 23
- Deuterated Reagents & Reactions 41
- Polymers 46

Further GPC Data 120

Computational Details 174

References 177

General Experimental Details & Catalyst Synthesis

Reagents were purchased from Sigma Aldrich or Acros, with the exception of pentane and bromoform (Fisher). Solvents used in synthesis/reactions were dried with sodium/benzophenone and distilled before use. NMR data was collected on 300, 400 or 500 MHz Bruker or Agilent machines as stated and chemical shifts referenced to residual solvent peaks. All manipulations were carried out under an argon atmosphere using standard Schlenk/glovebox technique unless stated.

Molecular weights and polydispersity were determined through Size Exclusion Chromatography using an Agilent 1260 Infinity Gel Permeation Chromatography (GPC) instrument equipped with a PLgel 5 µm MIXED-D column (300 x 7.5 mm) at 1 µl min^{-1} flow rate at 35 °C. Samples were dissolved in GPC-grade THF at a concentration of 1 mg ml^{-1}. Molecular sizes and other data were determined through in-built Triple Detection analysis. Differential Scanning Calorimetry (DSC) analysis was conducted using a TA Instruments DSC Q20 Instrument. The samples were ramped from 25 °C to 200 °C at 10 °C min^{-1}, then cooled back down to 25 °C at the same rate. A second heating cycle at the same rate was then performed for purposes of analysis.

General reaction setup for polymerisation

Experiments were performed under an argon atmosphere in an M-Braun glove box. The required pre-catalyst and ammonia borane (or alternative additive) were accurately weighed out and dissolved in dry deuterated benzene (600 µl) in a J-Young tap NMR tube. To this was added the required monomer, and the tube was sealed for the time and conditions reported.

To isolate the polymer, the solvent was removed from the reaction under reduced pressure. The residue was dissolved in approximately 2 ml of dry THF and, under an argon atmosphere, passed through a plug of approximately 1 cm of silica and 1 cm of celite. The resulting solution was dried under reduced pressure, yielding the polymer as a sticky brown solid.

For the scaled-up reaction, a J-Young tapped Schlenk flask was instead used as a vessel, and the residue was dissolved in approximately 10 ml of dry THF for purification.

For preparation of samples for DSC and WAXD analysis, the isolated polymer was redissolved in the minimum amount of benzene at room temperature. Dry methanol was added to the stirred solution dropwise, until precipitation of polymer was observed. The solution was decanted, the solid washed with methanol a second time, then dried under reduced pressure. This yielded an amorphous light brown solid.

Methanol was dried by refluxing with 3 Å molecular sieves for 2 hours, distilling and storing under a dry nitrogen atmosphere with fresh sieves

Dimer formation reaction setup

The reaction was setup as per the standard procedure, with the exception of varied reaction conditions. Following the specified time and temperature, the solvent was removed under reduced pressure. The residue was dissolved in pentane and the dimer isolated by passing through a silica plug. The pentane was removed by passing the eluted solution under a flow of pentane, yielding the product as a white precipitate.

Synthesis of Catalyst and Iron Complexes

Iron catalyst **[Fe]** was prepared as previously reported. [1] Iron hydride dimer **([Fe]H)$_2$** was prepared as previously reported. [2] The iron(I) complex **[Fe](I)** was prepared using the method reported by Scheer and in our previous report. [3, 4]

Nanoparticle Test

The procedure was adapted from Morris. [5] Reactions were set up under standard conditions but additionally trimethylphosphine (1.3 µl, 2.5 mol%, 0.5 equiv. with respect to catalyst) was added. Reaction was observed to be reduced, but not fully, indicating the active catalyst in the reaction was not nanoparticle-based

Radical Clock Test

We have previously used this test in other reaction studies. [6] The reaction was initially prepared as standard, and reacted for 2 hours, where conversion was determined by NMR. Under an argon atmosphere (chloromethyl)cyclopropane (1.2 µl, 0.0125 mmol, 2.5 mol%) was added to the reaction. After a further 14 hours conversion was determined to be 91% (under standard conditions, 93%). Distillation of the reaction mixture away from catalyst/polymer indicates that the majority of the radical clock remains as starting material, with only a small (<10 %) decyclised to form 1-butene, indicating to us that it is not halting reactivity by reacting with radical species.

Reaction under Hydrogen atmosphere

Phenylallene (0.5 mmol) and catalyst **[Fe]** (2.8 mg, 0.005 mmol, 1 mol%) were dissolved in 600 µl benzene-d$_6$ under an argon atmosphere. The reaction was then degassed using standard freeze-pump-thaw technique on a high-vacuum Schlenk line, and then purged with 1 atm H_2 gas, sealed and left to run to under standard reaction conditions (16 h, RT). After this timescale, no polymer was observed to have formed, and only starting material was observed (see ^{1}H NMR below).

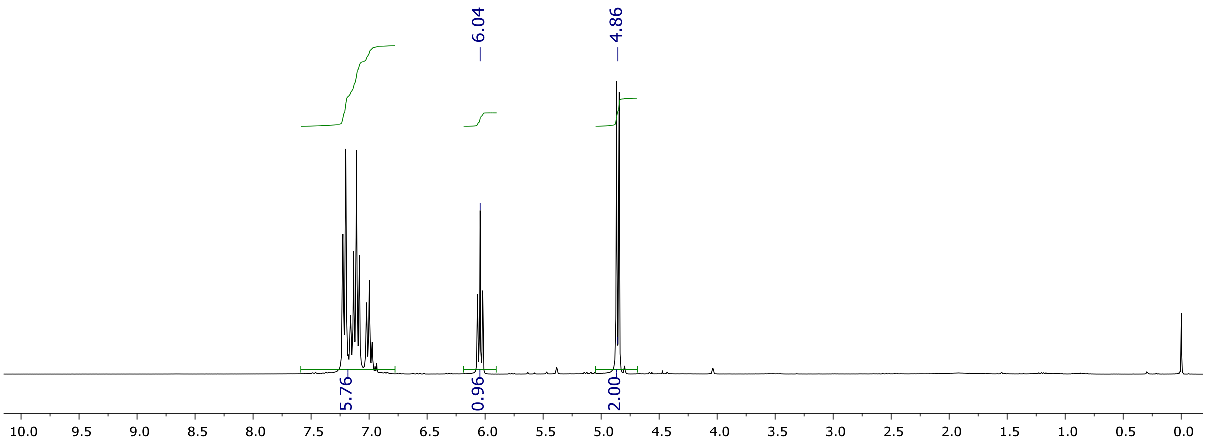

Use of [Fe](I) in catalysis

[Fe](I) was used under standard conditions except a hydride source was not required, generating a polymer with the properties below (GPC data report is in Further GPC Data section)

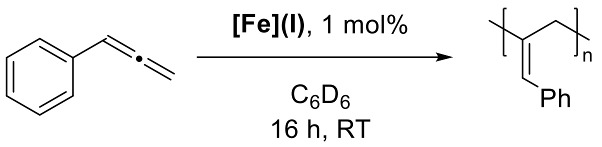

90% conversion
M_n = 190 kDa
6.2 : 1 ($P_{2,3}$:$P_{1,2}$)
D = 1.51

Blank Reactions

Phenylallene (0.5 mmol) was dissolved in 600 µl benzene-d_6 under an argon atmosphere and heated for 36 hours at 60 °C. No reaction or decomposition product was observed.

This reaction was repeated with the addition of **[Fe]** (2.8 mg, 0.005 mmol, 1 mol%) – in this case 3 % of phenylallene was consumed. The species isolated were polymeric but could not be characterised. Similar or lower conversions were observed when the reaction was run at room temperature, and at 5 mol% loading of catalyst.

Variable Temperature (VT) NMR Experiments

Experiments were performed on a Bruker 400 MHz NMR equipped with variable temperature apparatus. The isolated polymer (approx. 20 mg) was dissolved in 600 µl C_6D_6 and heated to 60 °C and then to 80 °C. Although the peaks belonging to the HC_3 positions broaden as the temperature is raised, they do not appear to interconvert or converge at raised temperature.

Heating the reaction for a continued period of time (80 °C, 3 days) under the conditions above does not alter the NMR spectra or ratio of intensities.

Room temperature spectra shown in **red**, 60 °C shown in **green**, 80 °C shown in **blue**

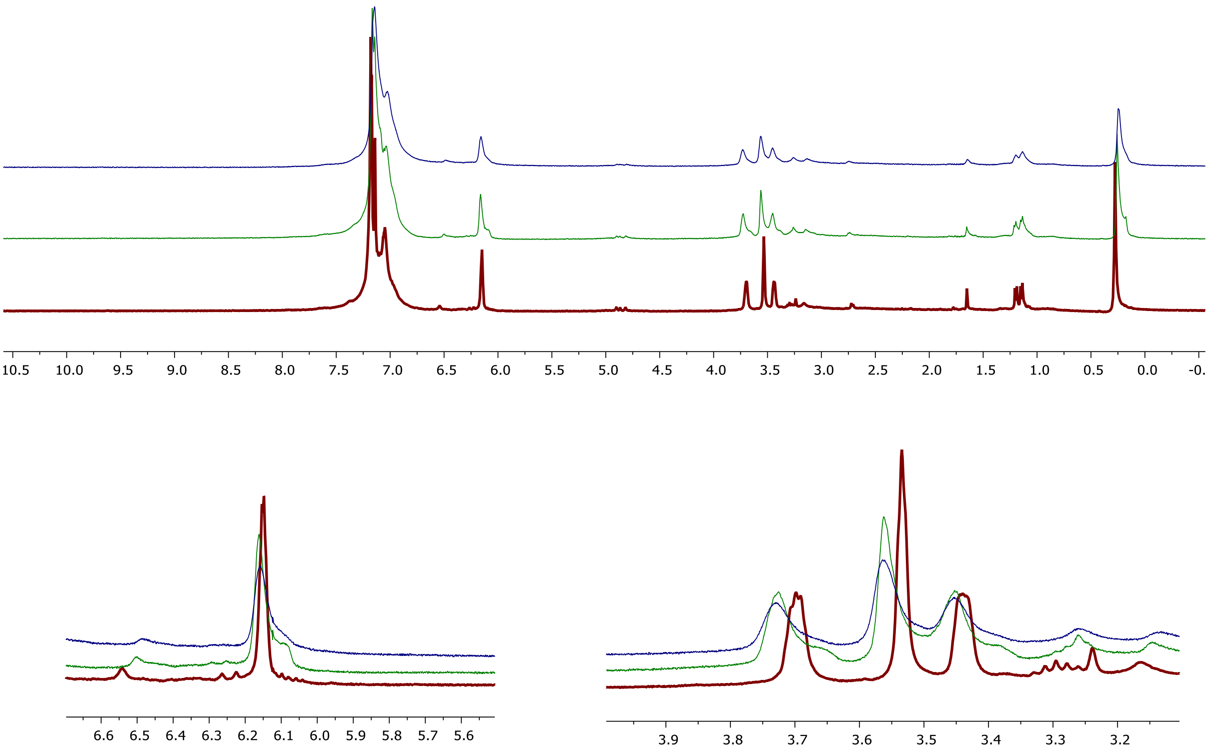

Competing Oxidative Addition Studies

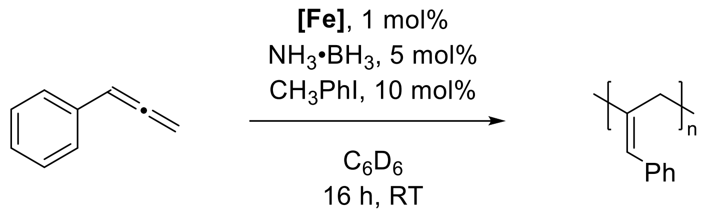

A catalytic amount of 4-iodotoluene (0.05 mmol, 10.9 mg) was added to the reaction (standard conditions) to observe if initiation could be altered through oxidative addition to form an Fe(III) species. No change to the reaction outcome was observed, and no incorporation of tolyl species was observed on the polymer.

Synthesis of Allenes

The general synthesis was adapted from the procedure reported by Buono [7].

Preparation of Styrenes (where required)

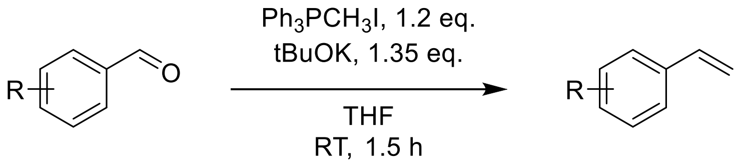

In a Schlenk flask under a nitrogen atmosphere, methyltriphenylphosphonium iodide (7.2 g, 18 mmol, 1.2 eq.) was dissolved in 50 ml dry THF, cooled to 0 °C and stirred. In a separate Schlenk flask, potassium *tert*-butoxide (2.28 g, 20.4 mmol, 1.35 eq) was dissolved in 12 ml dry toluene, and the resultant slurry added slowly to the first solution. The resultant solution was allowed to stir for 15 minutes at room temperature, following which it was cooled to 0 °C and the required aldehyde (15 mmol) was added dropwise (if the required aldehyde was solid, it was dissolved in 10 ml dry THF beforehand and the solution added dropwise). The reaction was warmed to room temperature and allowed to stir for 90 minutes. Following this, the reaction was quenched with ammonium chloride solution (2.5 g in 25 ml deionized water), and the product was extracted with ethyl acetate (3 x 50 ml). The combined organic phases were washed with water (100 ml), brine (100 ml) and dried with magnesium sulfate. Volatiles were removed under reduced pressure and the products purified with silica gel column chromatography using 10:1 hexane : ethyl acetate as the eluent.

Synthesis of gem dibromocyclopropanes

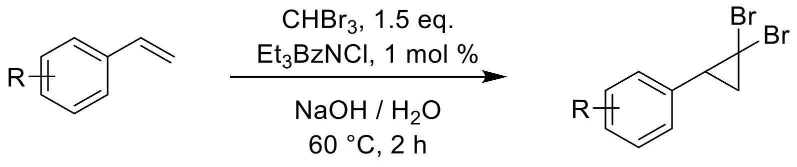

This reaction was performed under air. Sodium hydroxide (3.2 g, 80 mmol, 4 eq.) was dissolved in 3 ml of deionized water and allowed to cool to room temperature. To this was added the required styrene (20 mmol), bromoform (2.6 ml, 30 mmol, 1.5 eq.) and benzyl triethylammonium chloride (50

mg, 0.2 mmol, 0.01 eq.). The reaction was stirred at 60 °C for 2 hours, and then quenched by addition of 20 ml of water. The product was extracted with dichloromethane (3 x 50 ml), washed with brine (50 ml) and dried with magnesium sulphate. Volatiles were then removed under reduced pressure. The product was then purified by silica gel chromatography using 100% petroleum ether as the eluent, except for 5A, where 4:96 Ethyl Acetate : Petroleum Ether was used.

Synthesis of allenes

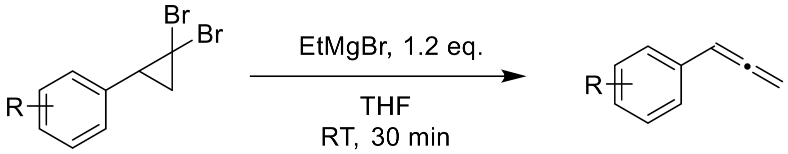

This reaction was performed under a nitrogen atmosphere using standard Schlenk techniques. The required dibromocyclopropane (10 mmol) was added to 20 ml of dry THF and cooled to 0 °C. To this, ethylmagnesium bromide (3.0 M in diethyl ether, 4 ml, 12 mmol, 1.2 equiv) was added dropwise, and the reaction was allowed to warm to room temperature. The reaction was cooled to 0 °C and quenched after 30 minutes through careful addition of deionized water (2 ml). 15 ml of 1 M hydrochloric acid was added to the reaction, and the product was extracted with petroleum ether (2 x 10 ml). The organic layers were combined, dried with magnesium sulphate and the solvent removed under reduced pressure. In most cases the product was found to be pure, however if needed further purification was undertaken by silica gel chromatography using pentane as the eluent. The product was then dried with calcium hydride, distilled, degassed and then stored in a freezer under an argon atmosphere.

Synthesis of Deuterated Reagents

Deuterated amine boranes ($Me_2ND \cdot BH_3$ and $Me_2NH \cdot BD_3$) were synthesised as previously reported. [8]

The synthesis of 3,3-d_2-phenylallene and intermediates *en route* described below were adapted from the procedure reported by Murphy. [9]

Synthesis of CD_3PPh_3I

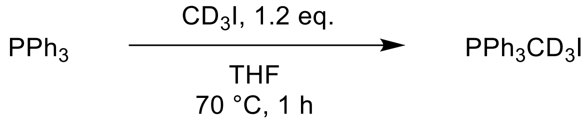

Triphenylphosphine (5.62 g, 21.4 mmol) was dissolved in 40 ml dry THF in a flame-dried Schlenk flask under a nitrogen atmosphere. Iodomethane-d_3 (1.61 ml, 3.67 g, 25.7 mmol) was added dropwise, and the flask was then sealed and heated to 70 °C for 1 hour. A white precipitate was observed to form. The precipitate was filtered on a Buchner funnel and washed with xylenes. Vacuum drying for 1 hour led to a white precipitate forming (8.67 g, 97% yield, 98% d-incorporation).

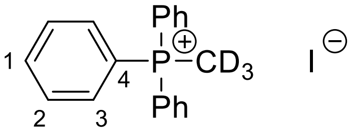

^{1}H (500 MHz, $CDCl_3$) 7.86-7.64 (ar, m, 15H)

$^{13}C\{^{1}H\}$ (126 MHz, $CDCl_3$) 135.4 (C_1, d, ^{4}J = 3.0 Hz), 133.5 (C_3, d, ^{2}J = 10.9 Hz), 130.6 (C_2, d, ^{3}J = 12.9 Hz), 119.0 (C_4, d, ^{1}J = 88.7 Hz), 25.8 (Me, s)

^{2}H (77 MHz, $CDCl_3$) 3.21 (s)

$^{31}P\{^{1}H\}$ (202 MHz, $CDCl_3$) 22.5 (s)

Synthesis of Styrene-β,β-d_2

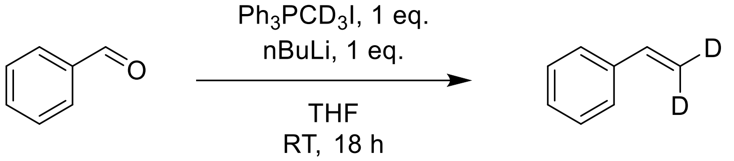

Methyltriphenylphosphonium iodide-d3 (4.87 g, 12 mmol) was dissolved in 30 ml of dry THF in a Schlenk flask under an argon atmosphere. The solution was cooled to -78 °C and n-butyl lithium solution (1.43 M in hexanes, 8.4 ml, 12 mmol, 1 eq) was added dropwise. After 15 minutes, the solution was allowed to warm to room temperature, and stirred for an additional thirty minutes. The solution was then cooled again and benzaldehyde (1.21 ml, 12 mmol, 1 eq) was added dropwise. After 20 minutes the reaction was allowed to warm to room temperature, and stirred for a further 18 hours. The reaction was then quenched by addition of 1 M HCl solution. The crude product was extracted with petroleum ether (2 × 25 ml), dried with magnesium sulfate and the solvent removed under reduced pressure. The product was purified by silica column chromatography using 100% pentane and the solvent was removed under reduced pressure yielding a colourless oil (479 mg, 38% yield).

Synthesis of (2,2-dibromocyclopropyl-3,3-d_2)benzene

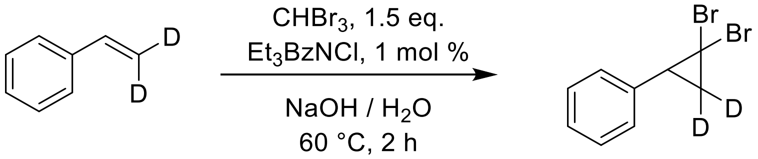

This reaction was performed under air. Sodium hydroxide (0.64 g, 16 mmol, 4 eq.) was dissolved in 0.6 ml of deionized water and allowed to cool to room temperature. To this was added styrene-β,β-d_2 (423 mg, 4 mmol), bromoform (0.52 ml, 6 mmol, 1.5 eq.) and benzyl triethylammonium chloride (10 mg, 0.04 mmol, 0.01 eq.). The reaction was stirred at 60 °C for 2 hours, and then quenched by addition of 20 ml of water. The product was extracted with dichloromethane (3 x 20 ml), washed with brine (50 ml) and dried with magnesium sulphate. Volatiles were then removed under reduced pressure. The product was then purified by silica gel chromatography using 100% petroleum ether as the eluent, yielding a clear oil (242 mg, 22% yield).

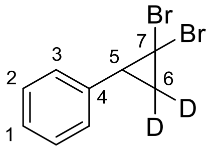

^{1}H (500 MHz, $CDCl_3$) δ 7.39-7.27 (ar, m, 5H) 2.95 (HC_5, br s, 1H)

$^{13}C\{^1H\}$ (126 MHz, $CDCl_3$) δ 136.4 (C_4), 129.3 (C_3), 128.7 (C_1), 128.0 (C_2), 36.1 (C_5), 28.7 (C_7) (C_6 not observed)

^{2}H (77 MHz, $CDCl_3$) δ 2.06 (d, ^3J = 8.9 Hz)

Synthesis of 3,3-d$_2$-phenylallene

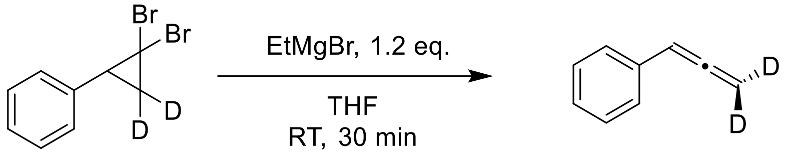

This reaction was performed under a nitrogen atmosphere using standard Schlenk techniques. (2,2-dibromocyclopropyl-3,3-d$_2$)benzene (278 mg, 1 mmol) was added to 10 ml of dry THF and cooled to 0 °C. To this, ethylmagnesium bromide (3.0 M in diethyl ether, 0.5 ml, 1.5 mmol, 1.5 equiv) was added dropwise, and the reaction was allowed to warm to room temperature. The reaction was cooled to 0 °C and quenched after 30 minutes through careful addition of deionized water (2 ml). 10 ml of 1 M hydrochloric acid was added to the reaction, and the product was extracted with petroleum ether (2 x 10 ml). The organic layers were combined, dried with magnesium sulphate and the solvent removed under reduced pressure, leaving a clear oil. (114 mg, 84% yield, 93% D-incorporation)

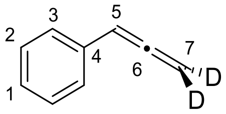

^{1}H (500 MHz, C_6D_6) δ 7.26-6.93 (ar, m, 5H), 6.05 (HC_5, s, 1H)

$^{13}C\{^1H\}$ (126 MHz, C_6D_6) δ 210.2 (C_6), 134.3 (C_3), 128.9 (C_2), 127.2 (C_1), 127.1 (C_3), 94.6 (C_5), 78.5 (m, C_7)

^{2}H (77 MHz, C_6D_6) δ 4.84 (s)

Use of 3,3-d$_2$-phenylallene in catalysis

Conditions and workup used were identical to the protic substrate. The ^{2}H NMR signals appear as a single broad peak rather than three distinct environments as observed in the protic polymer, however this is likely due to the broader signals associated with ^{2}H NMR rather than an indication of structure.

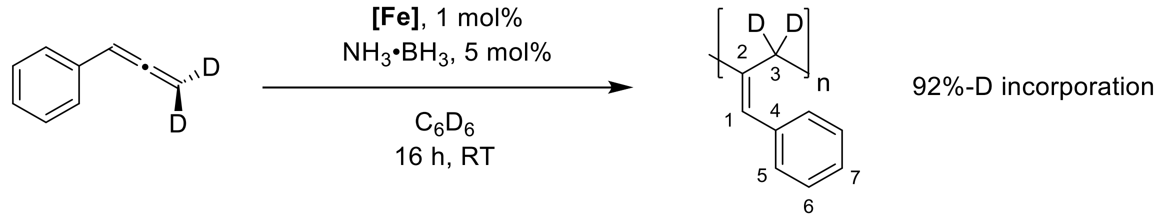

^{1}H (500 MHz, C_6D_6) δ 7.29-7.01 (ar, m, 5H), 6.24 (HC_1, s, 1H), 3.67/3.57/3.52 (residual protic HC_3, three m)

^{13}C{^{1}H} (126 MHz, C_6D_6) δ 138.3 (C_4), 138.2 (C_2), 128.8 (C_7), 127.7 (C_5), 126.6 (C_6), 123.0 (C_1), (C_3 not observed)

^{2}H (77 MHz, C_6D_6) δ 3.70/3.54/3.45 (all s)

NMR Characterisation of Polymers

Polyphenylallene (P1)

93% spectroscopic conversion, 6.5 : 1 (2,3) : (1,2) conformation, 82% isolated product (48 mg, brown solid)

$P1_{2,3}$ Isomer

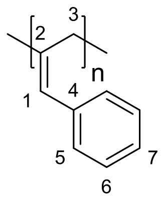

^{1}H (500 MHz, C_6D_6) δ 7.29-7.01 (ar, m, 5H) 5.97 (HC_1, s, 1H), 3.71/3.55/3.47 (HC_3, three s, 2H, 0.5/0.9/0.6 signal intensity ratio)

^{13}C{^{1}H} (126 MHz, C_6D_6) δ 137.9 (C_4), 137.9/137.8/137.7 (C_2), 128.4 (C_7), 127.3 (C_5), 126.2 (C_6), 122.6/122.4 (C_1) 42.4/42.0/41.6 (C_3)

$P1_{1,2}$ Isomer

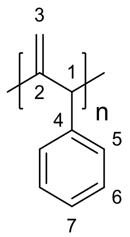

^{1}H (500 MHz, C_6D_6) δ 7.29-7.01 (ar, m, 5H), 4.83/5.38 (HC_3, two s, 2H, 1:1 signal intensity ratio), 3.26 (HC_1, s, 1H),

^{13}C{^{1}H} (126 MHz, C_6D_6) δ 56.7/42.9 (C_1), 105.2/112.4 (C_3), 150.6/142.1 (C_2) (aryl signals could not be determined)

Poly(4-methylphenyl) allene (P2)

94% spectroscopic conversion, 6.5 : 1 (2,3) : (1,2) conformation, 79% isolated product (51 mg, brown solid)

P2$_{2,3}$ Isomer

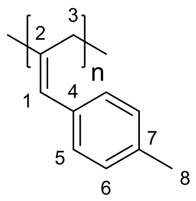

^{1}H (500 MHz, C_6D_6) δ 7.26-6.93 (ar, m, 4H), 6.22 (HC_1, s, 1H), 3.81/3.62/3.52 (HC_3, three s, 2H, 0.5/1/0.5 signal intensity), 2.15 (HC_8, s, 3H)

^{13}C{^{1}H} (126 MHz, C_6D_6) δ 137.1 (C_7), 136.0 (C_2), 135.5 (C_4), 129.5 (C_6), 127.7 (C_5), 122.8/122.7 (C_1), 42.7/42.3/41.9 (C_3), 21.2 (C_8)

P2$_{1,2}$ Isomer

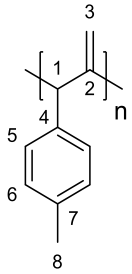

^{1}H (500 MHz, C_6D_6) δ 7.26-6.93 (ar, m, 4H), 4.96/5.40 (HC_3, two s, 2H, 1:1 signal intensity ratio), 4.11 (HC_1, s, 1H), 2.08 (HC_8, s, 3H)

^{13}C{^{1}H} (126 MHz, C_6D_6) δ 142.8 (C_2), 137.3 (C_7), 111.8 (C_3), 30.3 (C_1), 21.2 (C_8) (remaining aryl peaks not determined)

Poly(3-methylphenyl) allene (P3)

96% conversion, 7:1 (2,3) : (1,2) conformation, 80% isolated product (58 mg, brown oil)

P3$_{2,3}$ Isomer

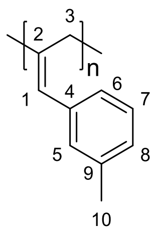

^{1}H (500 MHz, C_6D_6) δ 7.21-6.98 (ar, m, 4H), 6.22 (HC_1, m 1H), 3.83/3.62/3.52 (HC_3, three s, 2H, 0.5/0.9/0.5 signal intensity ratio), 2.15 (HC_{10}, s, 3H)

^{13}C{^{1}H} (126 MHz, C_6D_6) δ 138.2/138.1/138.0 (C_4), 138.0 (C_9), 137.8 (C_2), 128.7 (aryl), 128.6 (aryl), 127.4 (C_5), 124.7/124.8 (aryl), 122.9/123.1 (C_1), 41.9/42.5/42.8 (C_3), 21.6 (C_{10})

Although the signals at δ = 128.7 ppm, 128.6 ppm and 124.7/125.8 ppm respectively could be identified as aryl carbons, they could not be definitively assigned to the specific positions of C_6, C_7 and C_8.

P3$_{1,2}$ Isomer

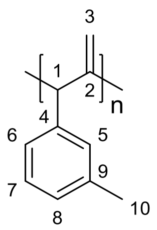

^{1}H (500 MHz, C_6D_6) δ 7.21-6.98 (ar, m, 4H), 4.99/4.89 (HC_3, two s, 2H, 1:1 signal intensity ratio) , 3.30 (HC_1, s, 1H), 2.19 (HC_{10}, s, 3H)

^{13}C{^{1}H} (126 MHz, C_6D_6) δ 148.0 (C_4), 140.1 (C_9), 136.4 (C_2), 112.6 (C_3), 28.4 (C_1), 21.6 (C_{10}) (remaining aryl signals not determined)

Poly(2-methylphenyl)allene (P4)

91% conversion, 5 : 1 (2,3) : (1,2) conformation, 82% isolated product (52 mg, brown oil)

P4$_{2,3}$ Isomer

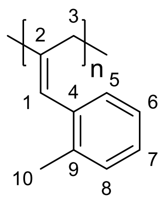

^{1}H (500 MHz, C_6D_6) δ 7.21-6.98 (ar, m, 4H), 6.41 (HC_1, m, 1H), 3.67/3.53/3.51 (HC_3, m, 2H, 0.5/0.8/0.6 signal intensity ratio), 2.17 (HC_{10}, s, 3H)

^{13}C{^{1}H} (126 MHz, C_6D_6) δ 141.3 (C_4), 138.2/138.3 (C_2), 136.4/136.6 (C_9), 130.6 (C_8), 127.3 (aryl), 126.8 (aryl), 126.2 (aryl), 120.3/120.1 (C_1), 42.5/41.9/41.2 (C_3), 20.0 (C_{10})

Although the signals at δ = 127.3 ppm, 126.8 ppm and 126.2 ppm respectively could be identified as aryl carbons, they could not be affirmatively assigned to the specific positions of C_5, C_6 and C_7. (C_8 was identified through strong HMBC coupling with HC_{10})

P4$_{1,2}$ Isomer

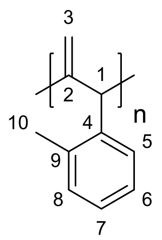

^{1}H (500 MHz, C_6D_6) δ 6.90-7.41 (ar, m, 4H), 4.85-5.11 (HC_3, m, 2H), 2.98 (HC_1, m, 1H), 2.19 (HC_{10}, s, 3H)

$^{13}C\{^1H\}$ (126 MHz, C_6D_6) δ 161.5 (C_2), 142.8 (C_4), 136.6 (C_9), 125.9 (aryl) 123.6 (aryl), 94.3 (C_3), 28.7 (C_1), 20.8 (C_{10}) (Remaining aryl signals not determined, the signals for C_9 for each isomer are also partially overlayed)

Poly(4-methoxyphenyl)allene (P5)

98% conversion, 11 : 1 (2,3) : (1,2) conformation, 92% isolated product (64 mg, brown solid)

P5$_{2,3}$ Isomer

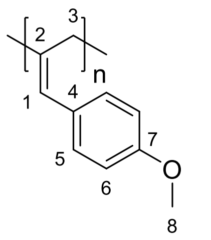

^1H (500 MHz, C_6D_6) δ 7.18 (HC_5, m, 2H) 6.80 (HC_6, m, 2H), 6.22 (HC_1, s, 1H), 3.80/3.66/3.57 (HC_3, 3 s, 2H, 1/0.5/1 signal intensity ratio), 3.36 (HC_8, s, 3H)

$^{13}C\{^1H\}$ (126 MHz, C_6D_6) δ 158.7 (C_7), 135.9 (C_2), 131.1 (C_5), 128.8 (C_4), 122.4 (C_1), 114.3 (C_6), 54.9 (C_8), 42.6/42.4/41.6 (C_3)

P5$_{1,2}$ Isomer

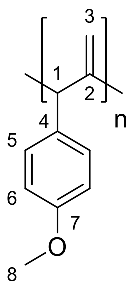

^1H (500 MHz, C_6D_6) δ 7.31-6.70 (aryl, m, 4H) 5.44/4.97/4.88 (HC_3, 3 s, 2H, 1/2/2 signal intensity ratio), 3.29 (HC_8) (HC_1 is obscured by (2,3) polymer methoxy group and chain peaks)

$^{13}C\{^1H\}$ (126 MHz, C_6D_6) δ 159.5 (C_7), 148.4 (C_2), 135.7 (C_4), 130.4/130.1 (C_3), 54.8 (C_8) (Remaining aryl and C_1 peaks not determined)

Poly(4-fluorophenyl)allene (P6)

77% spectroscopic conversion, 13 : 1 (2,3) : (1,2) conformation, 62% isolated product (42 mg, brown solid)

P6$_{2,3}$ Isomer

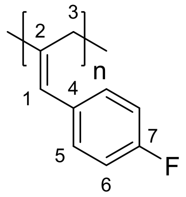

^{1}H (500 MHz, C_6D_6) δ 7.03-6.62 (ar, m, 4H), 6.03 (HC_1, m, 1H), 3.58/3.49/3.41 (HC_3, m, 2H, 0.5:0.5:1 signal intensity ratio)

^{13}C{^{1}H} (126 MHz, C_6D_6) δ 161.9 (C_7, d, ^{1}J = 245.8 Hz), 137.4/137.3/137.3 (C_2, three s), 130.4 (C_4, s), 129.1 (C_5, d, ^{3}J = 7.4 Hz), 121.8/121.6 (C_1, two s), 115.7 (C_6, d, ^{2}J = 21.4 Hz), 42.5/42.0/41.3 (C_3, three s)

^{19}F{^{1}H} (470 MHz, C_6D_6) -115.5 (s)

P6$_{1,2}$ Isomer

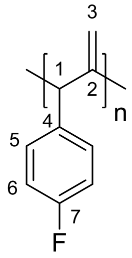

^{1}H (500 MHz, C_6D_6) δ 7.03-6.62 (ar, m, 4H), 4.89-4.71 (HC_3, m, 2H), 4.47 (HC_1, m, 1H)

^{19}F{^{1}H} (470 MHz, C_6D_6) δ -114.5 (s)

The yield of the (1,2) polymer is considerably reduced for this substrate, and as such ^{13}C{^{1}H} NMR signals were too weak to yield conclusive assignment

Poly(4-chlorophenyl)allene (P7)

74% spectroscopic conversion, 6 : 1 (2,3) : (1,2) conformation, 61% isolated product (45 mg, brown solid)

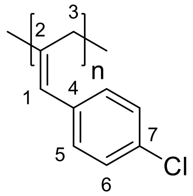

P7$_{2,3}$ Isomer

^{1}H (500 MHz, C_6D_6) δ 7.18 (HC_5, m, 2H), 6.87 (HC_6, m, 2H), 6.17 (HC_1, s, 1H), 3.43/3.34 (HC_3, two s, 2H, 0.5/1.5 signal intensity ratio)

^{13}C{^{1}H} (126 MHz, C_6D_6) δ 138.7/138.5 (C_2), 136.4 (C_4), 132.4 (C_7), 129.0 (C_5), 128.8 (C_6), 121.8/121.6 (C_1), 42.7/42.1/41.5 (C_3)

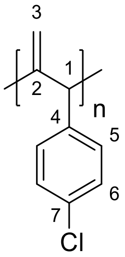

P7$_{1,2}$ Isomer

^{1}H (500 MHz, C_6D_6) δ 7.23-6.75 (ar, m, 4H), 4.89/4.84 (HC_3, two s, 2H, 1:1 signal intensity ratio), 4.47 (HC_1, s, 1H)

^{13}C{^{1}H} (126 MHz, C_6D_6) δ 142.8 (C_2), 94.3 (C_3), 20.8 (C_1) (aromatic peaks not determined)

WAXD Studies of Polyphenylallene

Experiments were performed on an Anton Paar SAXSpoint instrument with wavelength 0.154 nm, and sample-detector distance 119 mm.

A fibre of polymer was prepared by heating the polymer above T_g temperature and drawing the sample as it cooled. In WAXD analysis two broad rings (see image and 1D plot below) are observed at q = 5 and 13 nm^{-1}. Converting the q values into d-spacings gives distances of approximately 12.5 and 5 Angstroms. No Bragg peaks were observed from the sample, and no orientation could be seen in the 2D pattern, further supporting the hypothesis that the polymer is amorphous rather than crystalline. The observation of two diffraction rings is similar to that reported by Fujimori for amorphous comb copolymers. [1]

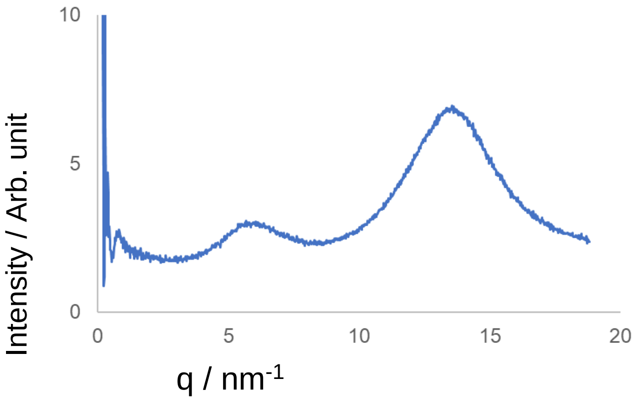

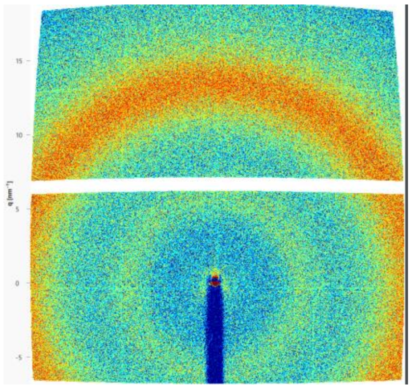

MALDI Studies of Polyphenylallene

Matrix-assisted laser-desorption time-of-flight (MALDI-ToF) analysis was performed using a Bruker Autoflex speed instrument using a DCTB matrix (trans2-[3-(4-tertbutylphenyl)-2-methyl-2-propenylidene]malononitrile) in a 5 : 1 mixture of matrix : polymer (both dissolved and prepared as 10 mg / ml solutions in THF). The equipment was operated in a linear acquisition mode using POS voltage polarity.

Output Spectra in 1000-3500 Da region

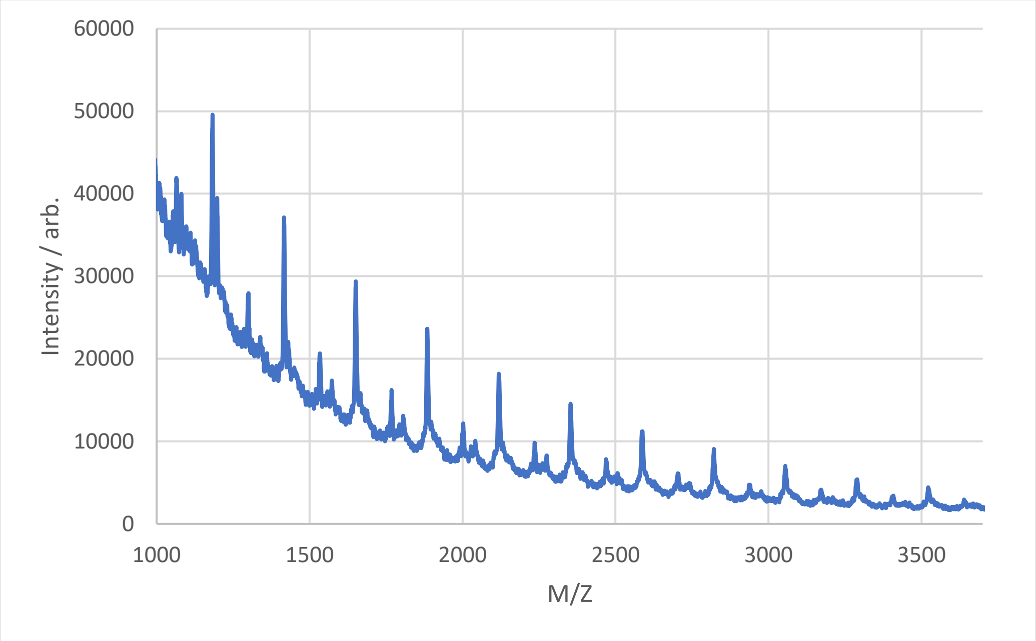

Summary of key peaks

m/z	time	Intens.	SN	Res.	Area	Rel. Intens.	FWHM
1009.119	21973.98	41172.000	3.555	131.143	81832.583	0.830	7.695
1065.348	22556.79	41713.000	3.831	189.463	52822.430	0.841	5.623
1182.494	23723.56	49581.461	10.008	224.182	124571.286	1.000	5.275
1299.581	24833.26	27985.000	3.148	224.696	38789.094	0.564	5.784
1416.600	25893.40	37132.000	9.622	252.285	124698.186	0.749	5.615
1533.614	26910.54	20660.000	3.183	216.054	41715.767	0.417	7.098
1650.891	27891.70	29385.000	9.140	266.149	130399.629	0.593	6.203
1768.111	28838.14	16231.000	3.384	327.153	35322.843	0.327	5.405
1884.755	29749.26	23618.000	8.593	298.995	129314.730	0.476	6.304
2118.921	31496.90	18189.000	7.384	283.094	118577.979	0.367	7.485
2353.199	33151.10	14554.000	6.587	289.998	113645.497	0.294	8.115
2587.007	34721.73	11197.000	5.699	311.302	96319.795	0.226	8.310
2821.237	36225.47	9074.000	4.945	330.467	74028.665	0.183	8.537
3054.910	37664.62	7031.000	4.129	308.015	62368.426	0.142	9.918
3289.027	39052.27	5355.535	3.190	351.508	45290.432	0.108	9.357

The above data suggest sodium adducts of short polymer lengths, example structures for the peak at m/z = 1416.6 are shown below. The data supports the notion that the allene remains intact at the point of initiation (as opposed to hydrogen-atom terminated polymer *e.g.* 1418.7). We cannot rule out the presence of cyclic species at lower M_n.

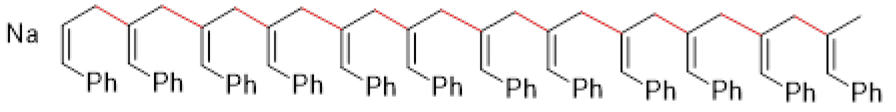

1418.7

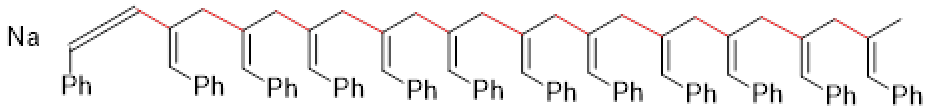

1416.7

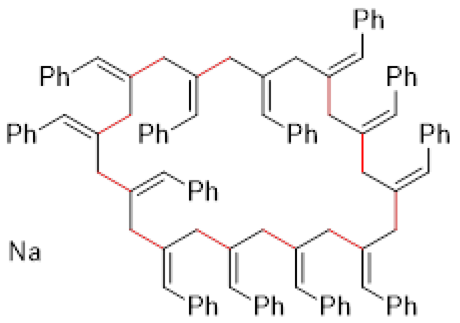

1416.7

Optimisation and Characterisation of Dimeric Species

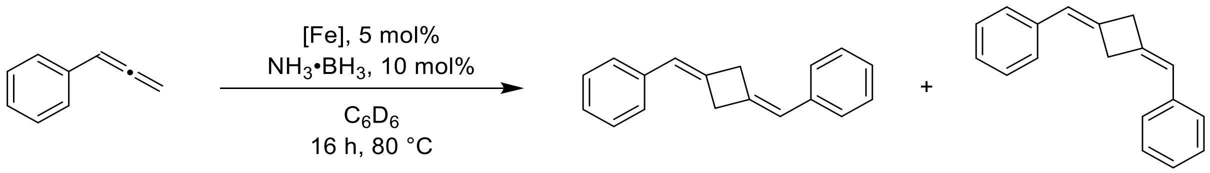

Studies were initially undertaken by altering the standard conditions (above and in general considerations section)

Entry	Variation from conditions above	Dimer Conversion / %	*Trans* : *Cis* Selectivity
1	16 h, 60 °C	16	1 : 2.0
2	16 h, RT	*Trace*	-
3	3 ml instead of 600 µl solvent	17	1 : 1.7
4	1 equivalent DMAP added	25	1 : 2.0
5	MeCN-d_3 instead of C_6D_6	-	-
6	1 h, 80 °C	13	1 : 1.8
7	HBpin instead of $NH_3 \cdot BH_3$	13	1 : 3.0
8	H_3SiPh instead of $NH_3 \cdot BH_3$	19	1 : 2.5
9	$KHBEt_3$ instead of $NH_3 \cdot BH_3$	-	-
10	$Me_2NH \cdot BH_3$ instead of $NH_3 \cdot BH_3$	25	1 : 2.0
11	No $NH_3 \cdot BH_3$	-	-

Further optimisation attempts were undertaken by slow addition of phenylallene solution into the reaction mixture. Phenylallene (0.5 mmol, 10% vol solution in benzene, 670 µl) was added to a solution of [Fe] (1 mol%) and $H_3N{\cdot}BH_3$ (5 mol%) in 6 ml benzene using a Cole-Parmer Syringe Pump at 20 µl/min. Conversion was determined by isolated product.

Entry	Catalyst Loading	Volume / ml	Temp / Time	Conversion / %
Standard Conditions	5%	0.6	80 °C, 16 h	27
12	5%	6 ml	80 °C, 16 h	28
13	1%	6 ml	80 °C, 16 h	7
14	5%	6 ml	RT, 16 h	-
15	1%	6 ml	RT, 16 h	-

NMR assignment of dimer

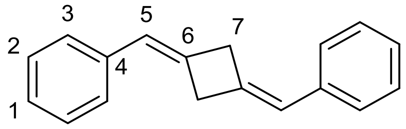

***Trans* Isomer**

^{1}H (500 MHz, $CDCl_3$) δ 7.36-7.15 (ar, m, 5H), 6.33 (HC_5, t, 2H, ^4J = 2.5 Hz) , 3.87 (HC_7, t, 4H, ^4J = 2.5 Hz)

^{13}C{^{1}H} (126 MHz, $CDCl_3$) δ 138.3 (C_6), 137.8 (C_4), 128.6 (C_2), 127.3 (C_3), 126.4 (C_1), 122.3 (C_5), 42.5 (C_7)

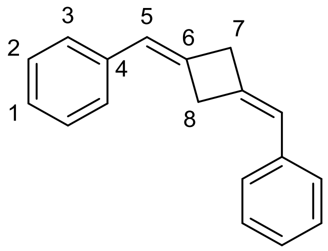

***Cis* Isomer**

The ^{1}H and ^{13}C{^{1}H} signals are distinct for HC_7/HC_8 and C_7/C_8, and were determined as the *J* coupling value between HC_8 and HC_5 is larger than HC_7 and HC_5

^{1}H (500 MHz, $CDCl_3$) δ 7.36-7.15 (ar, m, 5H), 6.30 (HC_5, dd, 2H, ^4J = 2.9 Hz, ^4J = 2.0 Hz), 4.01 (HC_8, dd, 2H, ^4J = 5.9 Hz, ^4J = 2.9 Hz), 3.71 (HC_7, dd, 2H, ^4J = 5.9 Hz, ^4J = 2.0 Hz)

^{13}C{^{1}H} (126 MHz, $CDCl_3$) δ 138.2 (C_6), 137.8 (C_4), 128.6 (C_2), 127.3 (C_3), 126.4 (C_1), 122.5 (C_5), 42.8 (C_7), 42.0 (C_8)

Characterisation of Allenes & Precursors

1A

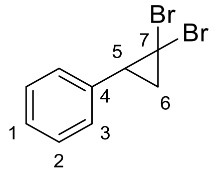

Obtained from standard procedure, 60% isolated yield (3.32 g), colourless liquid

^{1}H (500 MHz, $CDCl_3$) δ 7.42-7.25 (ar, m, 5H), 2.99 (HC_5, dd, ^{3}J = 10.5 Hz, ^{3}J = 8.1 Hz, 1H), 2.16 (HC_6, dd, ^{3}J = 10.5 Hz, ^{2}J = 7.7 Hz, 1H), 2.05 (HC_6, dd, ^{3}J = 8.1 Hz, ^{2}J = 7.7 Hz, 1H)

$^{13}C\{^{1}H\}$ (126 MHz, $CDCl_3$) δ 136.1 (C_4), 129.0 (C_3), 128.4 (C_1), 127.7 (C_2), 36.1 (C_5), 28.6 (C_7), 27.4 (C_6)

TOF-MS [M+H] 273.8978 (theoretical) 273.8993 (observed)

Data concordant with previous literature [7]

1B

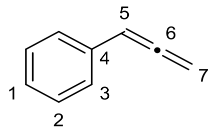

Obtained from standard procedure, 81% isolated yield (0.94 g), colourless liquid

^{1}H (500 MHz, $CDCl_3$) δ 7.34-7.17 (ar, m, 5H), 6.17 (HC_5, t, ^{4}J = 6.8 Hz, 1H), 5.15 (HC_7, d, ^{4}J = 6.8 Hz, 2H)

$^{13}C\{^{1}H\}$ (126 MHz, $CDCl_3$) δ 209.9 (C_6), 134.0 (C_4), 128.7 (C_2), 127.0 (C_1), 126.8 (C_3), 94.1 (C_5), 78.9 (C_7)

TOF-MS [M+H] 116.0626 (theoretical) 116.0728 (observed)

Data concordant with previous literature [7]

2A

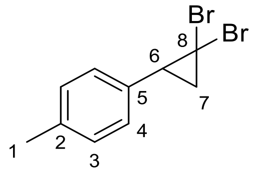

Obtained from standard procedure, 46% isolated yield (2.65 g), colourless liquid

^{1}H (500 MHz, $CDCl_3$) δ 7.19-7.12 (ar, m, 4H), 2.92 (HC_6, dd, ^{3}J = 10.5 Hz, ^{3}J = 8.3 Hz, 1H), 2.35 (HC_1, s, 3H), 2.12 (HC_7, dd, ^{3}J = 10.5 Hz, ^{2}J = 7.7 Hz, 1H), 2.01 (HC_7, dd, ^{3}J = 8.3 Hz, ^{2}J = 7.7 Hz, 1H)

$^{13}C\{^{1}H\}$ (126 MHz, $CDCl_3$) δ 137.5 (C_5), 133.1 (C_2), 129.2 (C_3), 128.9 (C_4), 35.8 (C_6), 29.0 (C_8), 27.3 (C_7), 21.4 (C_1)

TOF-MS [M+H] 287.9149 (theoretical), 287.9142 (observed)

Data concordant with previous literature [7]

2B

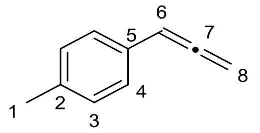

Obtained from standard procedure, 92% yield (1.20 g), colourless oil

^{1}H (500 MHz, CDCl$_3$) δ 7.20 (HC$_4$, d, ^{3}J = 7.9 Hz, 2H), 7.12 (HC$_3$, d, ^{3}J = 7.9 Hz, 2H), 6.14 (HC$_6$, t, ^{4}J = 6.8 Hz, 1H), 5.13 (HC$_8$, d, ^{4}J = 6.8 Hz, 2H) 2.33 (Me, s, 3H)

^{13}C{^{1}H} (126 MHz, CDCl$_3$) δ 209.8 (C$_7$), 136.8 (C$_2$), 131.0 (C$_5$), 129.5 (C$_3$), 129.4 (C$_4$), 126.7 (C$_3$), 93.9 (C$_6$), 78.8 (C$_8$), 21.3 (C$_1$)

TOF-MS [M+H] 130.0783 (theoretical), 130.0763 (observed)

Data concordant with previous literature [7]

3A

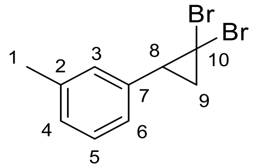

Obtained from standard procedure, 52% yield (3.01 g), colourless oil

^{1}H (500 MHz, CDCl$_3$) δ 7.43-7.02 (ar, m, 5H), 2.94 (HC$_8$, dd, ^{3}J = 10.5 Hz, ^{3}J = 8.4 Hz, 1H), 2.38 (HC$_1$, s, 3H), 2.12 (HC$_9$, dd, ^{3}J = 10.5 Hz, ^{2}J = 7.7 Hz, 1H), 2.01 (HC$_9$, dd, ^{3}J = 8.4 Hz, ^{2}J = 7.7 Hz, 1H)

^{13}C{^{1}H} (126 MHz, CDCl$_3$) δ 137.5 (C$_7$), 133.1 (C$_2$), 129.3 (C$_3$), 129.2 (C$_5$), 128.9 (C$_4$), 126.3 (C$_6$), 35.8 (C$_8$), 29.0 (C$_{10}$), 27.3 (C$_9$), 21.4 (C$_1$)

TOF-MS [M+H] 287.9195 (theoretical), 287.9149 (observed)

Data concordant with previous literature [9]

3B

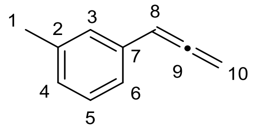

Obtained from standard procedure, 73% yield (0.95 g), colourless oil

^{1}H (500 MHz, CDCl$_3$) δ 7.34-7.17 (ar, m, 5H), 6.14 (HC$_5$, t, ^{4}J = 6.8 Hz, 1H), 5.13 (HC$_7$, d, ^{4}J = 6.8 Hz, 2H) 2.34 (Me, s, 3H)

^{13}C{^{1}H} (126 MHz, CDCl$_3$) δ 209.8 (C$_9$), 136.8 (C$_2$), 131.0 (C$_7$), 129.5 (C$_4$), 129.4 (C$_5$), 126.7 (C$_3$), 126.3 (C$_6$), 93.9 (C$_8$), 78.8 (C$_{10}$), 21.3 (C$_1$)

Data concordant with previous literature [9]

4A

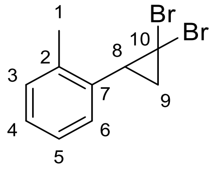

Obtained from standard procedure, 62 % yield (3.60 g), colourless oil

^{1}H (500 MHz, $CDCl_3$) δ 7.29-7.23 (HC_3/HC_6, m, 2H), 7.17 (HC_5, m, 1H) 6.98 (HC_4, m, 1H), 2.83 (HC_8, dd, 1H, ^{3}J = 10.4 Hz, ^{3}J = 8.4 Hz), 2.50 (HC_1, s, 3H), 2.15 (HC_9, dd, 1H, ^{3}J = 10.4 Hz, ^{2}J = 7.7 Hz), 2.05 (HC_9, dd, 1H, ^{3}J = 8.4 Hz, ^{2}J = 7.7 Hz)

^{13}C{^{1}H} (126 MHz, $CDCl_3$) δ 139.3 (C_7), 135.6 (C_2), 130.0 (C_3), 127.9 (C_6), 127.8 (C_4), 126.0 (C_5), 35.5 (C_8), 28.5 (C_{10}), 27.0 (C_9), 20.3 (C_1)

TOF-MS [M+H] 287.9149 (theoretical) 287.9028 (observed)

Data concordant with previous literature [7]

4B

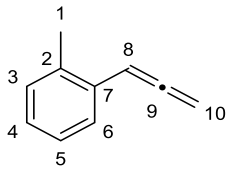

Obtained from standard procedure, 81% yield (1.05 g), pale yellow oil

^{1}H (500 MHz, $CDCl_3$) δ 7.39 (HC_6, m, 1H), 7.19-7.08 (HC_3/HC_4/HC_5, m, 3H), 6.35 (HC_8, t, 1H, ^{4}J = 6.8 Hz), 5.11 (HC_{10}, d, 2H, ^{4}J = 6.8 Hz), 2.36 (HC_1, s, 3H)

^{13}C{^{1}H} (126 MHz, $CDCl_3$) δ 210.6 (C_9), 135.0 (C_7), 132.3 (C_2), 130.6 (C_4), 127.3 (C_6), 127.0 (C_3), 126.3 (C_5), 91.3 (C_8), 78.1 (C_{10}), 20.0 (C_1)

TOF-MS [M+H] 130.0783 (theoretical) 130.0755 (observed)

Data concordant with previous literature [7]

5A

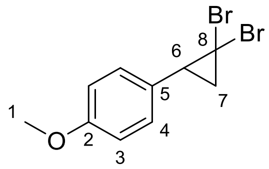

Obtained from standard procedure, 32% yield (1.98 g), colourless oil

^{1}H (500 MHz, $CDCl_3$) δ 7.17 (HC_4, d, 2H, ^{3}J = 8.7 Hz), 6.89 (HC_3, d, 2H, ^{3}J = 8.7 Hz), 3.81 (HC_1, s, 3H), 2.89 (HC_6, dd, 1H, ^{3}J = 10.6 Hz, ^{3}J = 8.2 Hz), 2.11 (HC_7, 1H, ^{3}J = 10.5 Hz, ^{2}J = 7.7 Hz), 1.95 (HC_7, 1H, ^{3}J = 8.2 Hz, ^{2}J = 7.7 Hz)

$^{13}C\{^{1}H\}$ (126 MHz, $CDCl_3$) δ 159.1 (C_2), 130.1 (C_4), 128.3 (C_5), 113.8 (C_3), 55.4 (C_1), 35.5 (C_6), 29.4 (C_8), 27.5 (C_7)

TOF-MS [M+H] 304.9098 (theoretical) 304.9223 (observed)

Data concordant with previous literature [7]

5B

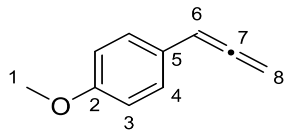

Obtained from standard procedure, 76% yield (0.98 g), pale yellow oil

^{1}H (500 MHz, $CDCl_3$) δ 7.24 (HC_4, d, 2H, ^{3}J = 8.7 Hz), 6.86 (HC_3, d, 2H, ^{3}J = 8.7 Hz) 6.13 (HC_6, t, 1H, ^{4}J = 6.8 Hz), 5.12 (HC_8, d, 2H, ^{4}J = 6.8 Hz), 3.80 (HC1, s, 3H)

$^{13}C\{^{1}H\}$ (126 MHz, $CDCl_3$) δ 209.5 (C_7), 158.9 (C_2), 127.9 (C_4), 126.3 (C_5), 114.3 (C_3), 93.5 (C_6), 78.9 (C_8), 55.4 (C_1)

TOF-MS [M+H] 147.0732 (theoretical) 147.0805 (observed)

Data concordant with previous literature [7]

6A

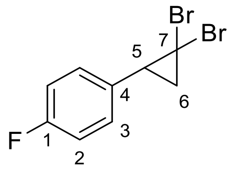

Obtained from standard procedure, 29% yield (1.71 g), colourless oil

^{1}H (500 MHz, $CDCl_3$) δ 7.26-7.19 (HC_3, m, 2H), 7.09-7.01 (HC_2, t, 2H, ^{3}J = 8.7 Hz), 2.92 (HC_5, ^{3}J = 10.5 Hz, ^{3}J = 8.3 Hz), 2.14 (HC_6, dd, 1H, ^{3}J = 10.5 Hz, ^{2}J = 7.8 Hz), 1.96 (HC_6, dd, 1H, ^{3}J = 8.3 Hz, ^{2}J = 7.8 Hz)

$^{13}C\{^{1}H\}$ (126 MHz, $CDCl_3$) δ 162.3 (C_1, d, ^{1}J = 246.6 Hz), 132.0 (C_4, d, ^{4}J = 3.2 Hz), 130.7 (C_3, d, ^{3}J = 8.2 Hz), 115.4 (C_2, d, ^{2}J = 21.7 Hz), 35.3 (C_5), 28.3 (C_7), 27.6 (C_6)

$^{19}F\{^{1}H\}$ (470 MHz, $CDCl_3$) δ -114.4 (s)

TOF-MS [M-H] 291.8899 (theoretical), 291.8990 (observed)

Data concordant with previous literature [9]

6B

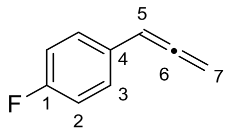

Obtained from standard procedure, 63% yield (0.85 g), colourless oil

^{1}H (500 MHz, $CDCl_3$) δ 7.24-7.19 (HC_3, m, 2H), 6.99-6.92 (HC_2, t, 2H, ^{3}J = 8.7 Hz), 6.09 (HC_5, t, 1H, ^{4}J = 6.8 Hz), 5.10 (HC_7, d, 2H, ^{4}J = 6.8 Hz)

$^{13}C\{^{1}H\}$ (126 MHz, $CDCl_3$) δ 209.7 (C_6), 162.0 (C_1, d, ^{1}J = 246.0 Hz), 130.0 (C_4, d, ^{4}J = 3.3 Hz), 128.2 (C_3, d, ^{3}J = 8.1 Hz), 115.7 (C_2, d, ^{2}J = 2.9 Hz), 93.2 (C_5), 79.2 (C_7)

$^{19}F\{^{1}H\}$ (470 MHz, $CDCl_3$) δ -115.6 (s)

TOF-MS [M+H] 134.0532 (theoretical), 134.0527 (observed)

Data concordant with previous literature [9]

7A

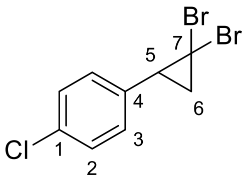

Obtained from standard procedure, 41% yield (2.55 g), colourless oil

^{1}H (500 MHz, $CDCl_3$) δ 7.33 (HC_3, d, ^{3}J = 8.5 Hz, 2H), 7.19 (HC_2, d, ^{3}J = 8.5 Hz, 2H), 2.91 (HC_5, dd, ^{3}J = 10.5 Hz, ^{3}J = 8.2 Hz, 1H), 2.15 (HC_6, dd, ^{3}J = 10.5 Hz, ^{2}J = 7.8 Hz), 1.97 (HC_6, dd, ^{3}J = 8.2 Hz, ^{2}J = 7.8 Hz, 1H)

$^{13}C\{^{1}H\}$ (126 MHz, $CDCl_3$) δ 134.7 (C_4), 133.7 (C_1), 130.4 (C_2), 128.7 (C_3), 35.5 (C_5), 27.9 (C_7), 27.6 (C_6)

TOF-MS [M+H] 307.8603 (theoretical) 307.8632 (observed)

Data concordant with previous literature [9]

7B

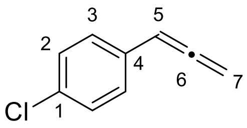

Obtained from standard procedure, 61% yield (0.92 g), colourless oil

^{1}H (500 MHz, $CDCl_3$) δ 7.27 (ar, d, ^{3}J = 8.6 Hz, 2H), 7.22 (ar, d, ^{3}J = 8.6 Hz, 2H), 6.12 (HC_5, t, ^{4}J = 6.8 Hz, 1H), 5.16 (HC_7, d, ^{4}J = 6.8 Hz, 2H)

$^{13}C\{^{1}H\}$ (126 MHz, $CDCl_3$) δ 210.0 (C_6), 132.6 (C_4), 132.6 (C_1), 128.9 (C_2), 128.0 (C_3), 93.3 (C_5), 79.3 (C_7)

TOF-MS [M+H] 150.0236 (theoretical) 150.0306 (observed)

Data concordant with previous literature [9]

NMR Spectra, GPC Data Reports, DSC Traces

Allenes & Precursors

1A

^{1}H

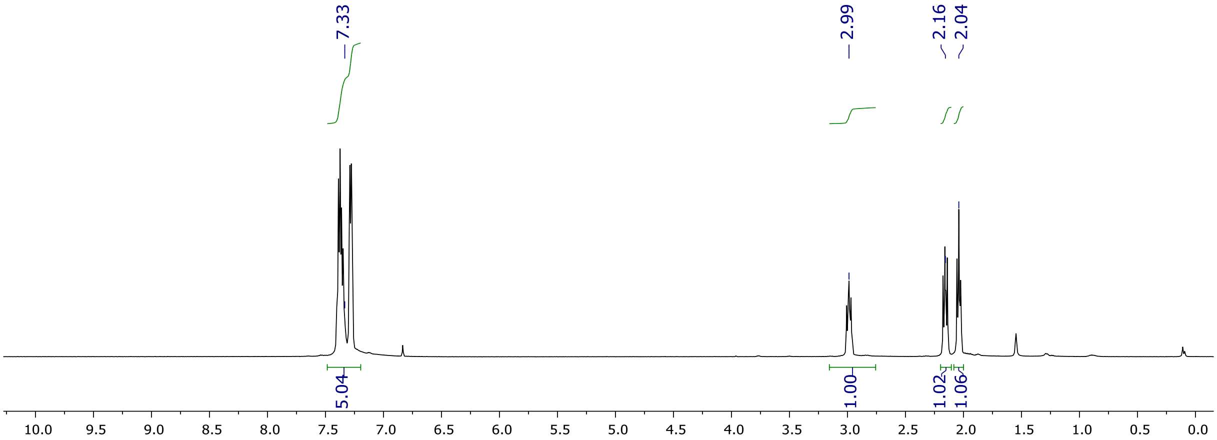

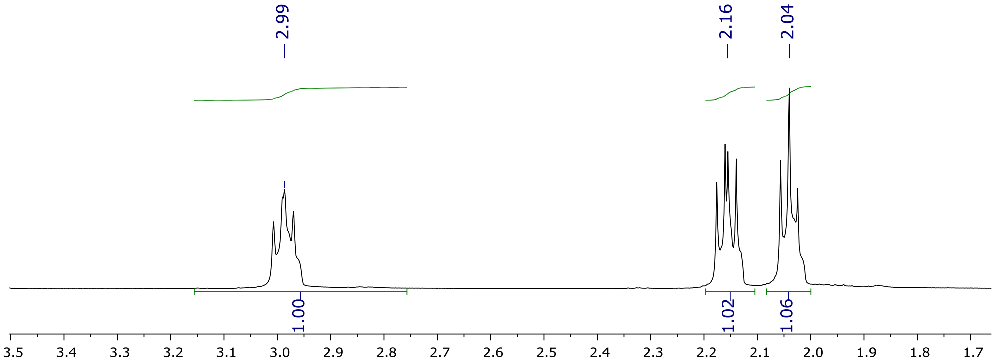

$^{13}C\{^{1}H\}$

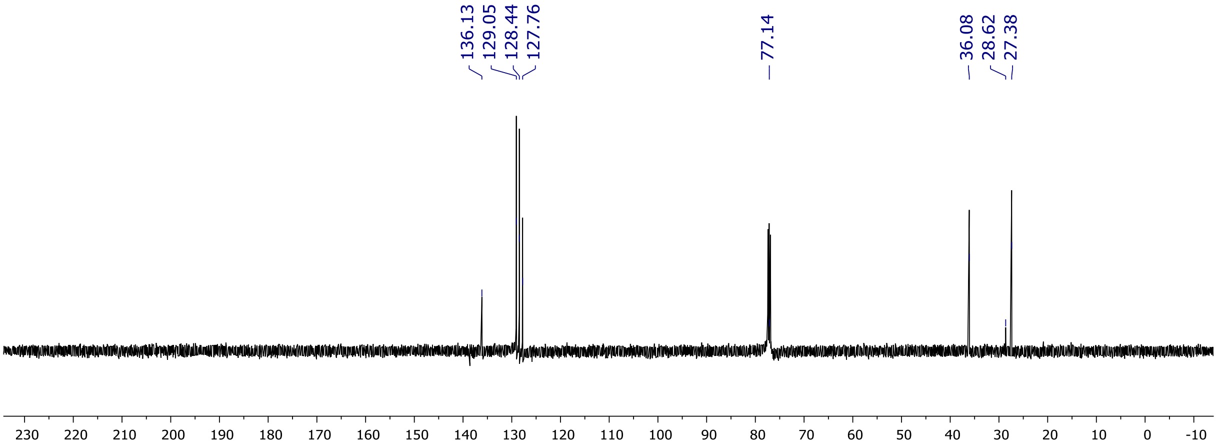
136.13
129.05
128.44
127.76
77.14
36.08
28.62
27.38
230 220 210 200 190 180 170 160 150 140 130 120 110 100 90 80 70 60 50 40 30 20 10 0 -10

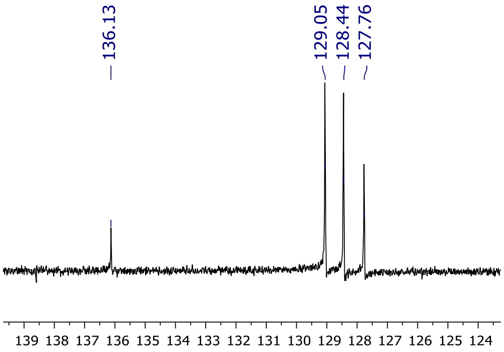
136.13
129.05
128.44
127.76
139 138 137 136 135 134 133 132 131 130 129 128 127 126 125 124

1B

^{1}H

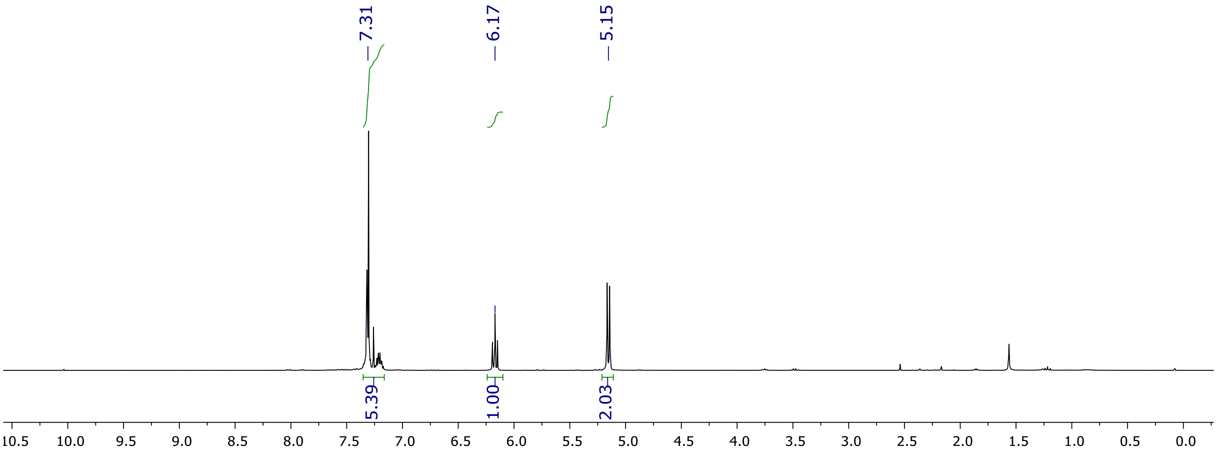

7.31
6.17
5.15
5.39
1.00
2.03
10.5 10.0 9.5 9.0 8.5 8.0 7.5 7.0 6.5 6.0 5.5 5.0 4.5 4.0 3.5 3.0 2.5 2.0 1.5 1.0 0.5 0.0

$^{13}C\{^{1}H\}$

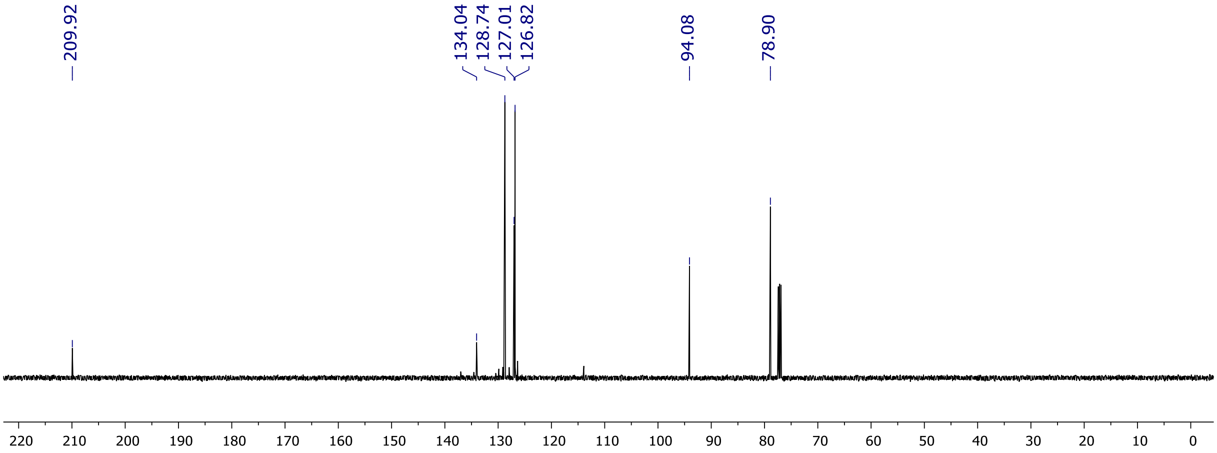

209.92
134.04
128.74
127.01
126.82
94.08
78.90
220 210 200 190 180 170 160 150 140 130 120 110 100 90 80 70 60 50 40 30 20 10 0

2A

^1H

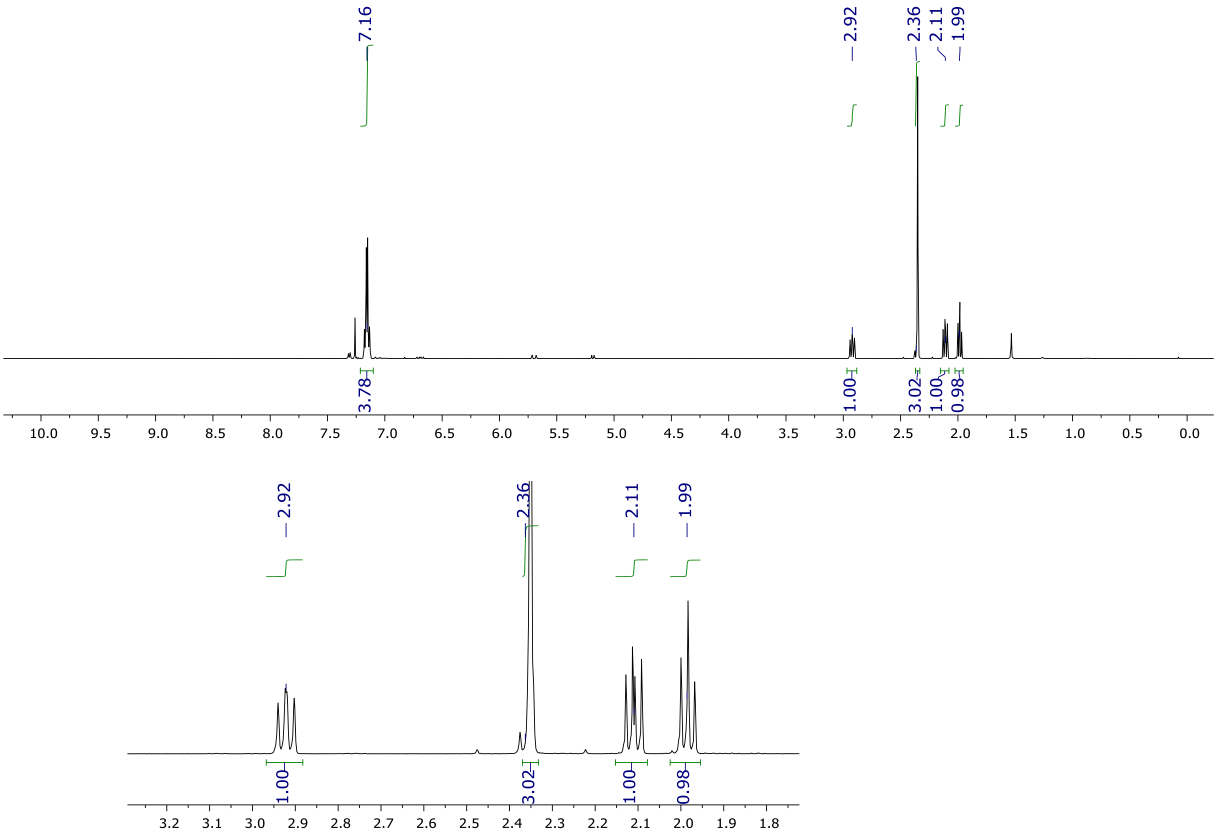

7.16
2.92
2.36
2.11
1.99
3.78
1.00
3.02
1.00
0.98
10.0
9.5
9.0
8.5
8.0
7.5
7.0
6.5
6.0
5.5
5.0
4.5
4.0
3.5
3.0
2.5
2.0
1.5
1.0
0.5
0.0
2.92
2.36
2.11
1.99
1.00
3.02
1.00
0.98
3.2
3.1
3.0
2.9
2.8
2.7
2.6
2.5
2.4
2.3
2.2
2.1
2.0
1.9
1.8

$^{13}C\{^{1}H\}$

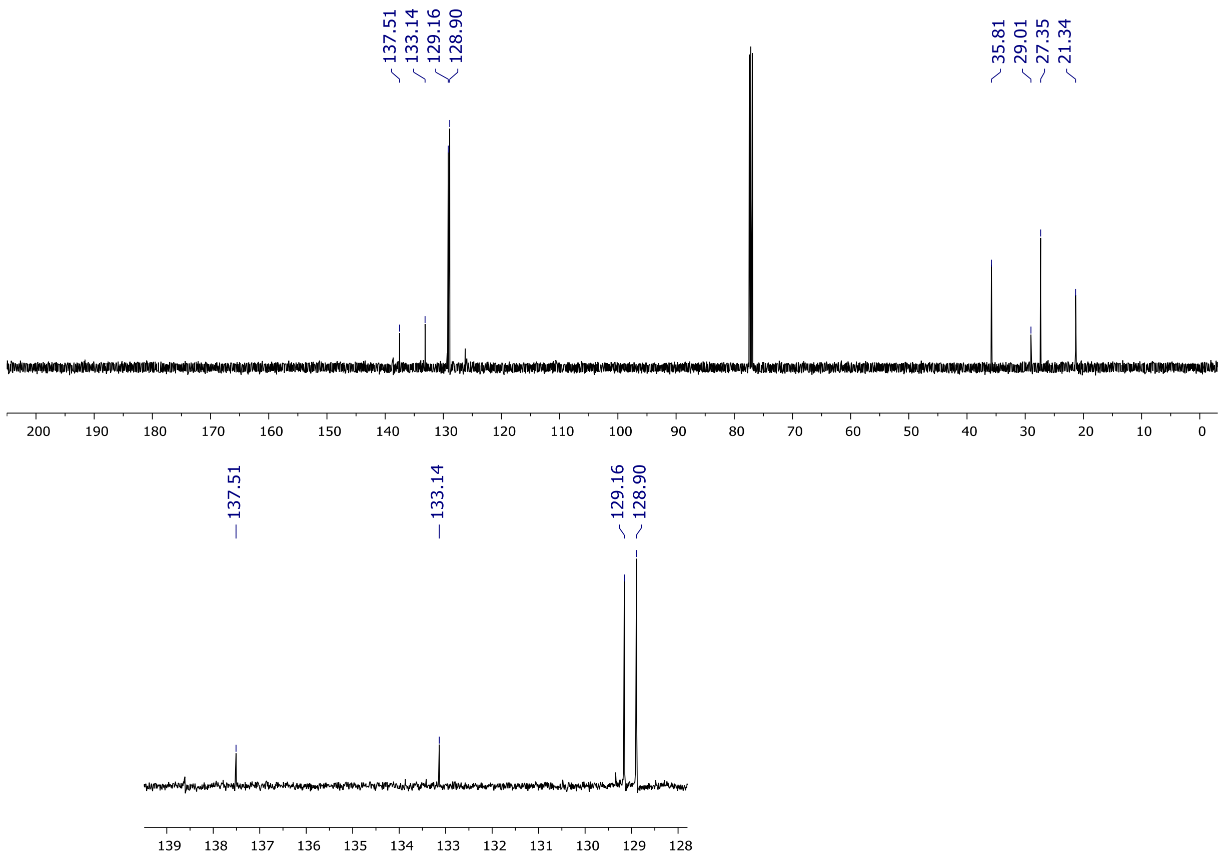
137.51
133.14
129.16
128.90
35.81
29.01
27.35
21.34
200 190 180 170 160 150 140 130 120 110 100 90 80 70 60 50 40 30 20 10 0
137.51
133.14
129.16
128.90
139 138 137 136 135 134 133 132 131 130 129 128

2B

^{1}H

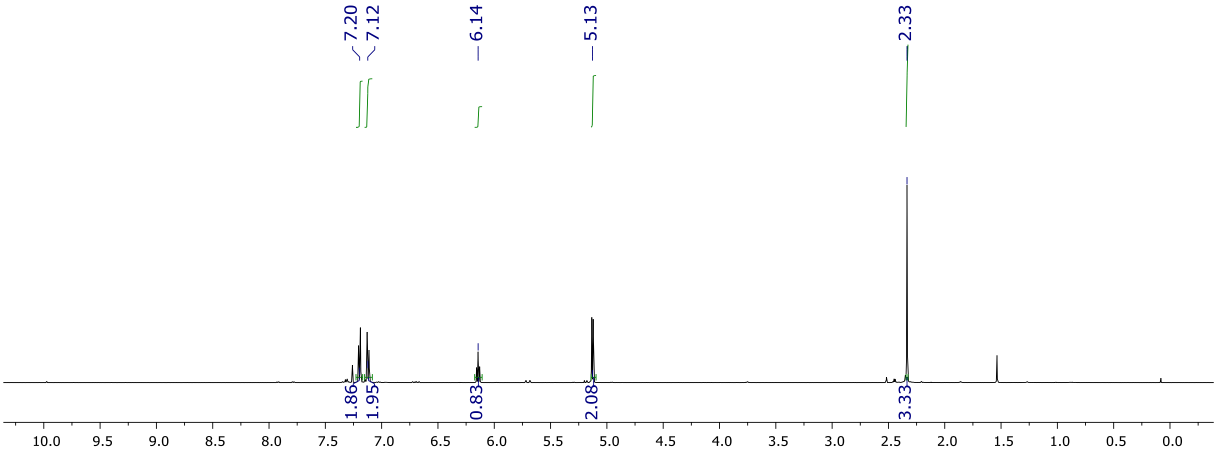
7.20
7.12
6.14
5.13
2.33
1.86
1.95
0.83
2.08
3.33
10.0 9.5 9.0 8.5 8.0 7.5 7.0 6.5 6.0 5.5 5.0 4.5 4.0 3.5 3.0 2.5 2.0 1.5 1.0 0.5 0.0

^{13}C{^{1}H}

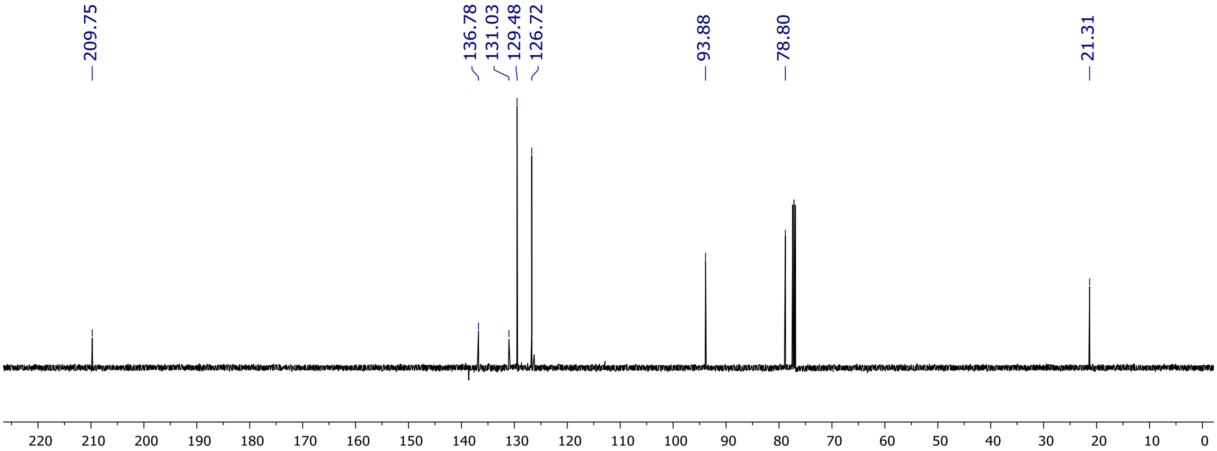
209.75
136.78
131.03
129.48
126.72
93.88
78.80
21.31
220 210 200 190 180 170 160 150 140 130 120 110 100 90 80 70 60 50 40 30 20 10 0

3A

^{1}H

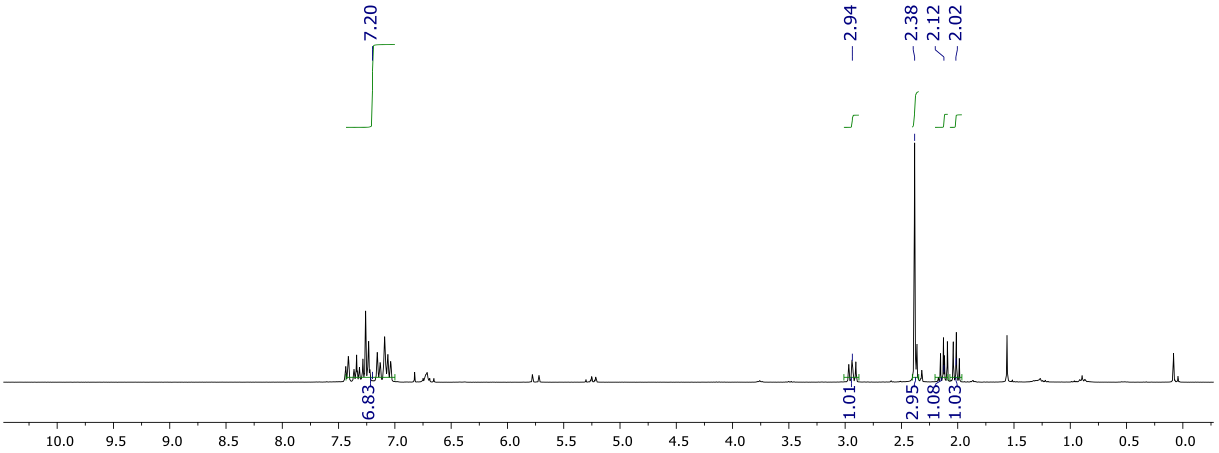
7.20
2.94
2.38
2.12
2.02
6.83
1.01
2.95
1.08
1.03
10.0 9.5 9.0 8.5 8.0 7.5 7.0 6.5 6.0 5.5 5.0 4.5 4.0 3.5 3.0 2.5 2.0 1.5 1.0 0.5 0.0

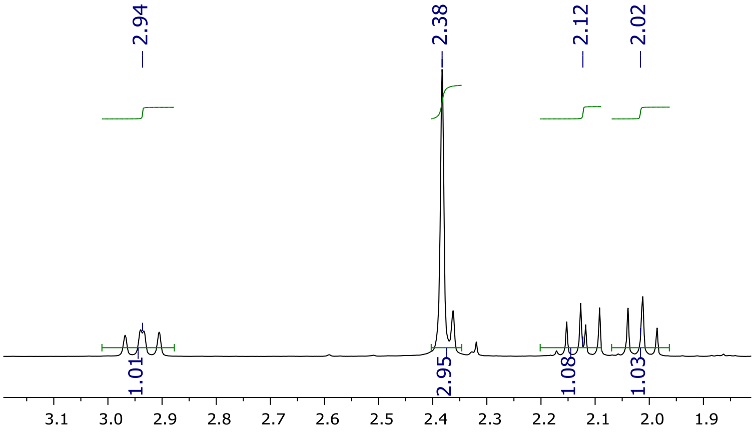
2.94
2.38
2.12
2.02
1.01
2.95
1.08
1.03
3.1 3.0 2.9 2.8 2.7 2.6 2.5 2.4 2.3 2.2 2.1 2.0 1.9

$^{13}C\{^{1}H\}$

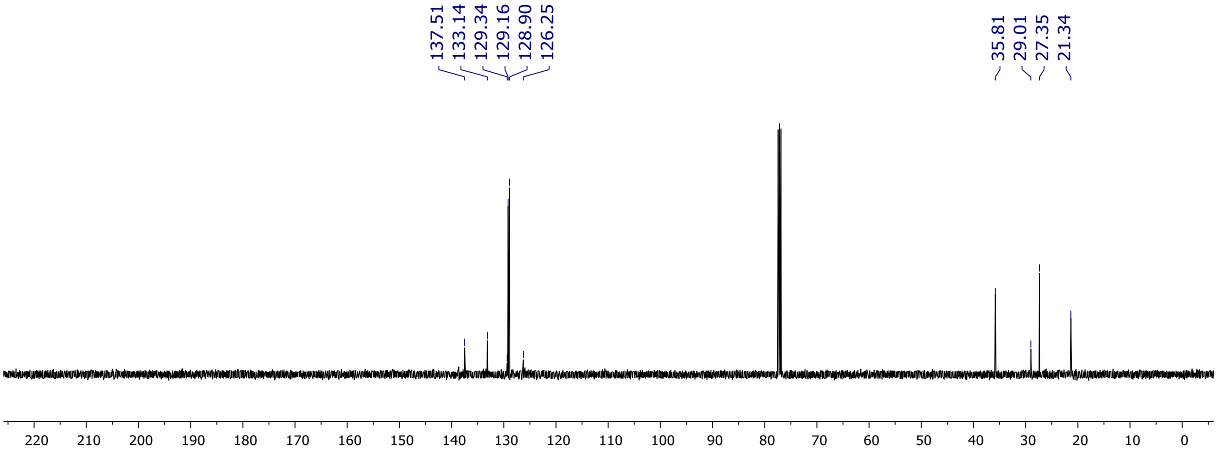
137.51
133.14
129.34
129.16
128.90
126.25
35.81
29.01
27.35
21.34
220 210 200 190 180 170 160 150 140 130 120 110 100 90 80 70 60 50 40 30 20 10 0

3B

^{1}H

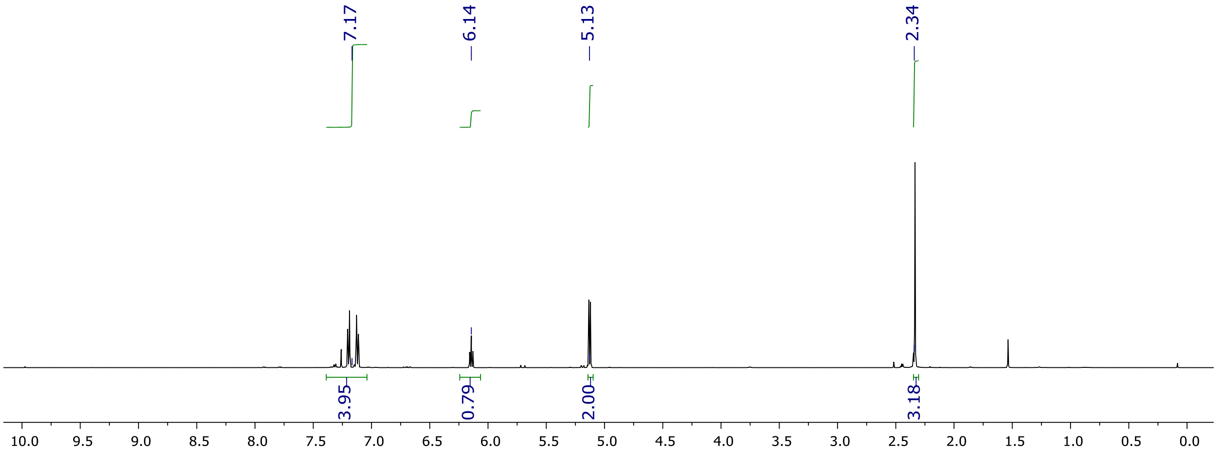

7.17
6.14
5.13
2.34
3.95
0.79
2.00
3.18
10.0 9.5 9.0 8.5 8.0 7.5 7.0 6.5 6.0 5.5 5.0 4.5 4.0 3.5 3.0 2.5 2.0 1.5 1.0 0.5 0.0

$^{13}C\{^{1}H\}$

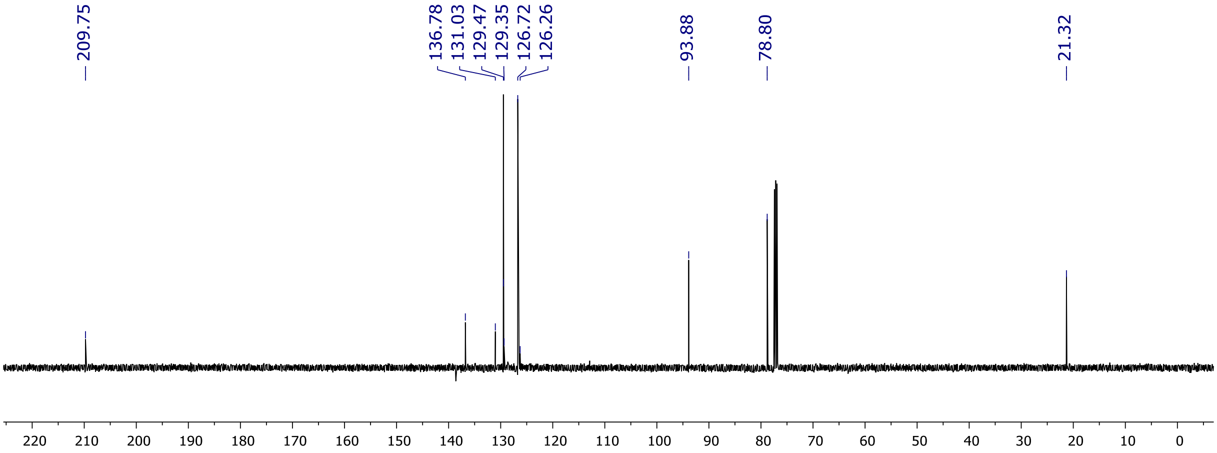

209.75
136.78
131.03
129.47
129.35
126.72
126.26
93.88
78.80
21.32
220 210 200 190 180 170 160 150 140 130 120 110 100 90 80 70 60 50 40 30 20 10 0

4A

^{1}H

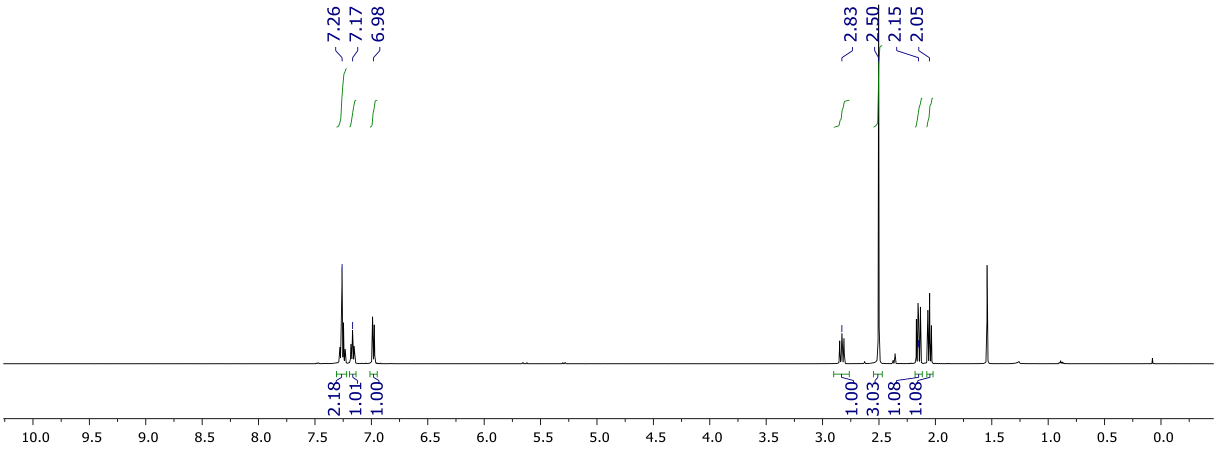

7.26
7.17
6.98
2.83
2.50
2.15
2.05
2.18
1.01
1.00
1.00
3.03
1.08
1.08
10.0 9.5 9.0 8.5 8.0 7.5 7.0 6.5 6.0 5.5 5.0 4.5 4.0 3.5 3.0 2.5 2.0 1.5 1.0 0.5 0.0

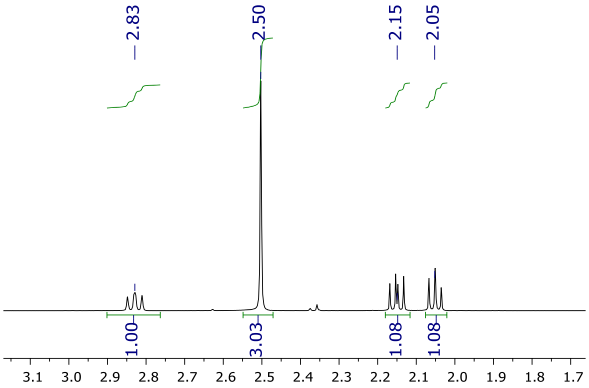

2.83
2.50
2.15
2.05
1.00
3.03
1.08
1.08
3.1 3.0 2.9 2.8 2.7 2.6 2.5 2.4 2.3 2.2 2.1 2.0 1.9 1.8 1.7

$^{13}C\{^{1}H\}$

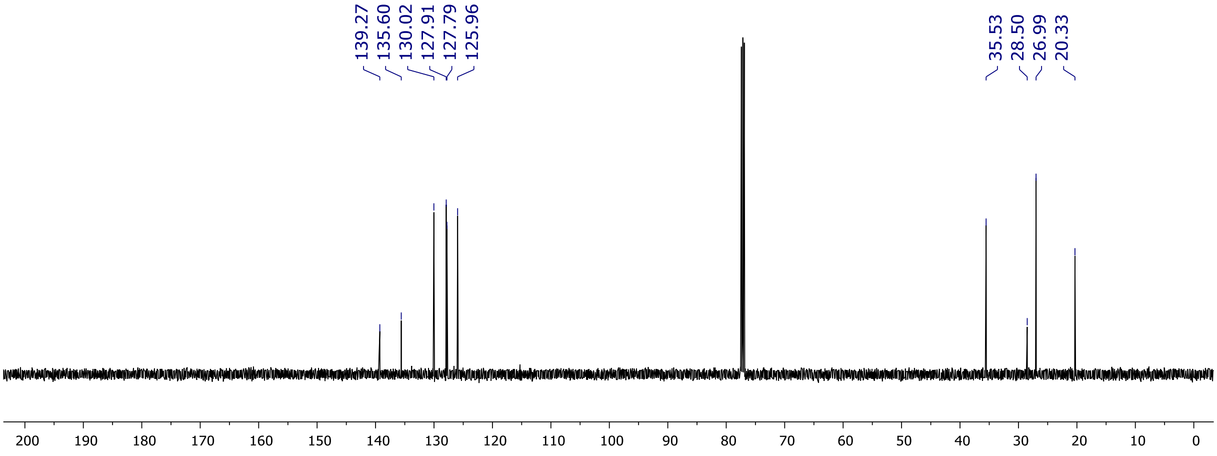

139.27
135.60
130.02
127.91
127.79
125.96
35.53
28.50
26.99
20.33
200 190 180 170 160 150 140 130 120 110 100 90 80 70 60 50 40 30 20 10 0

4B

^{1}H

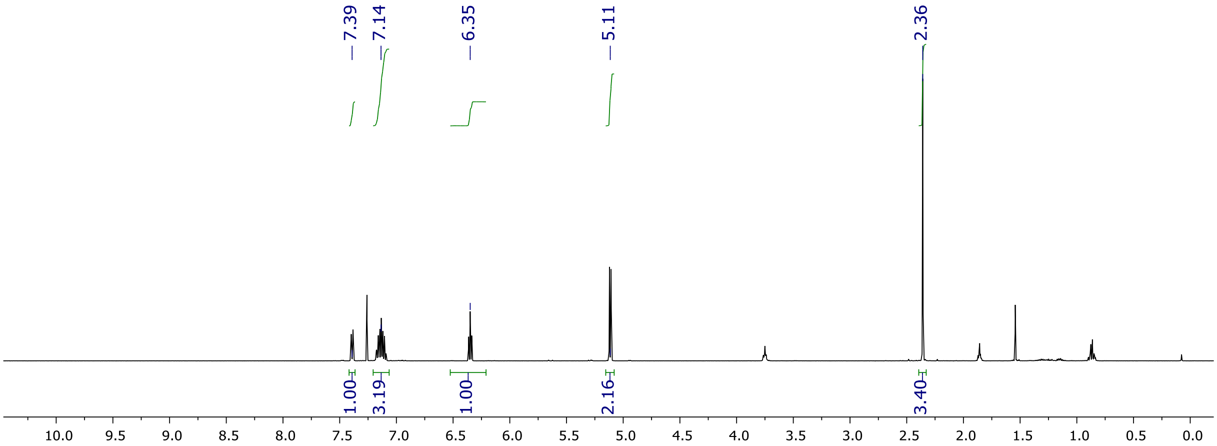

7.39
7.14
6.35
5.11
2.36
1.00
3.19
1.00
2.16
3.40
10.0 9.5 9.0 8.5 8.0 7.5 7.0 6.5 6.0 5.5 5.0 4.5 4.0 3.5 3.0 2.5 2.0 1.5 1.0 0.5 0.0

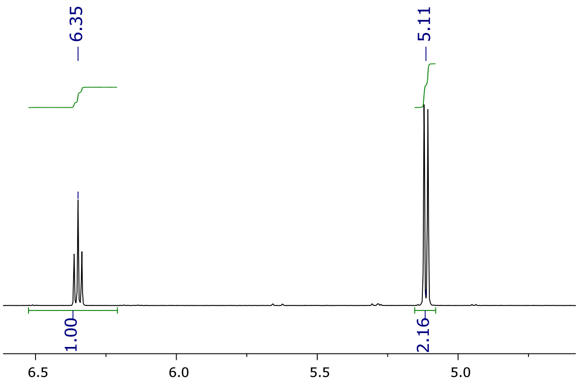

6.35
5.11
1.00
2.16
6.5 6.0 5.5 5.0

13C{1H}

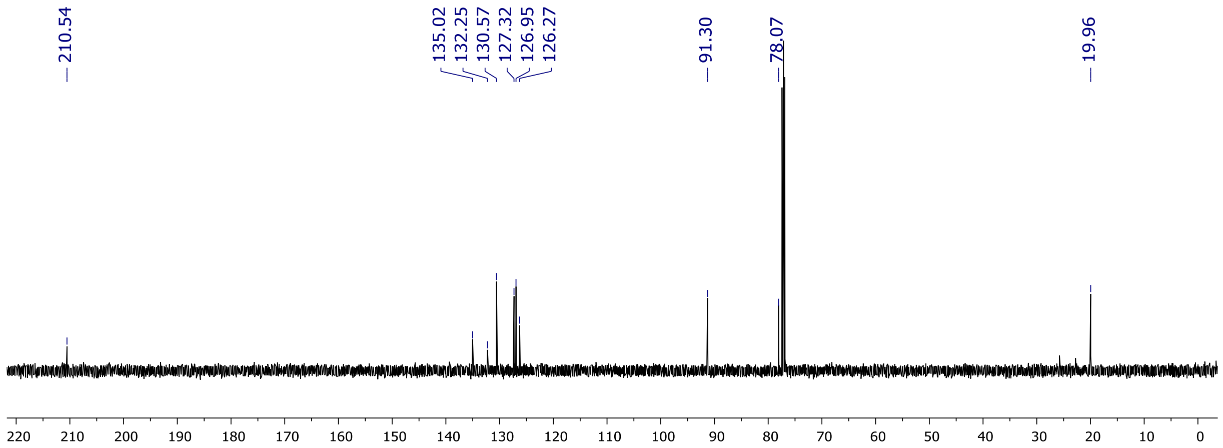
210.54
135.02
132.25
130.57
127.32
126.95
126.27
91.30
78.07
19.96
220 210 200 190 180 170 160 150 140 130 120 110 100 90 80 70 60 50 40 30 20 10 0

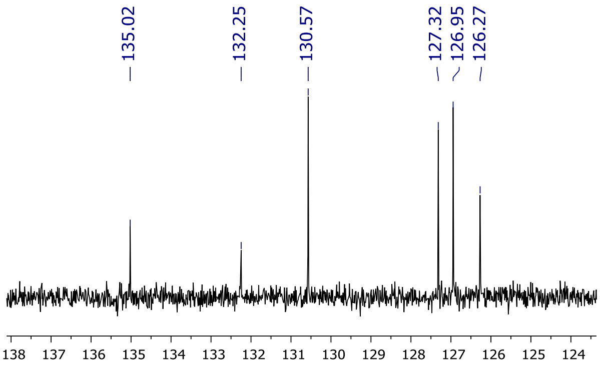
135.02
132.25
130.57
127.32
126.95
126.27
138 137 136 135 134 133 132 131 130 129 128 127 126 125 124

5A

^{1}H

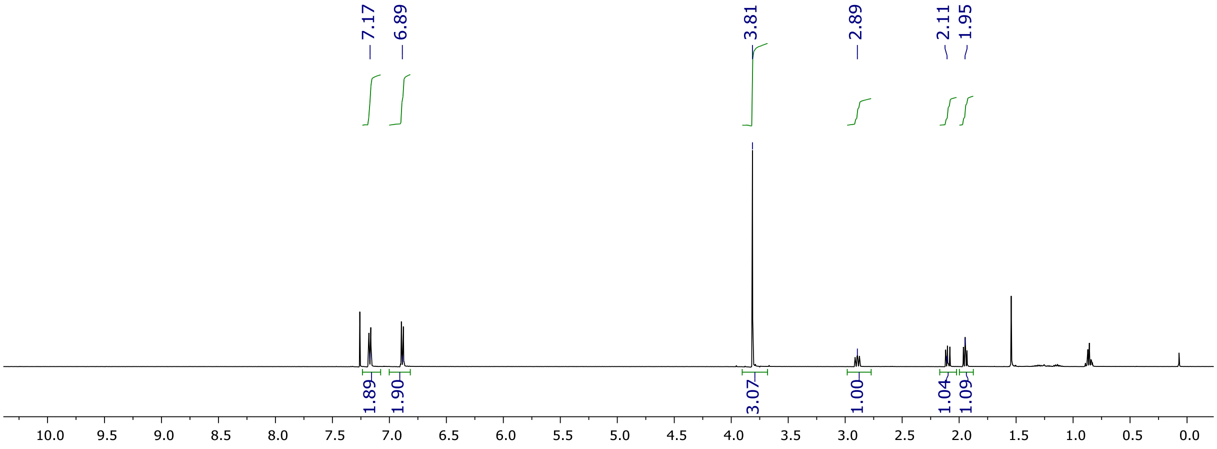
7.17
6.89
3.81
2.89
2.11
1.95
1.89
1.90
3.07
1.00
1.04
1.09
10.0 9.5 9.0 8.5 8.0 7.5 7.0 6.5 6.0 5.5 5.0 4.5 4.0 3.5 3.0 2.5 2.0 1.5 1.0 0.5 0.0

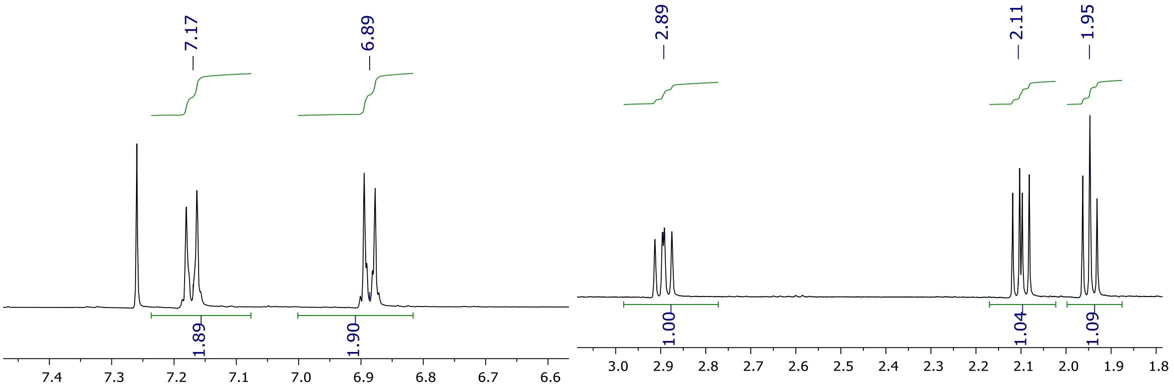
7.17
6.89
1.89
1.90
7.4 7.3 7.2 7.1 7.0 6.9 6.8 6.7 6.6
2.89
2.11
1.95
1.00
1.04
1.09
3.0 2.9 2.8 2.7 2.6 2.5 2.4 2.3 2.2 2.1 2.0 1.9 1.8

$^{13}C\{^{1}H\}$

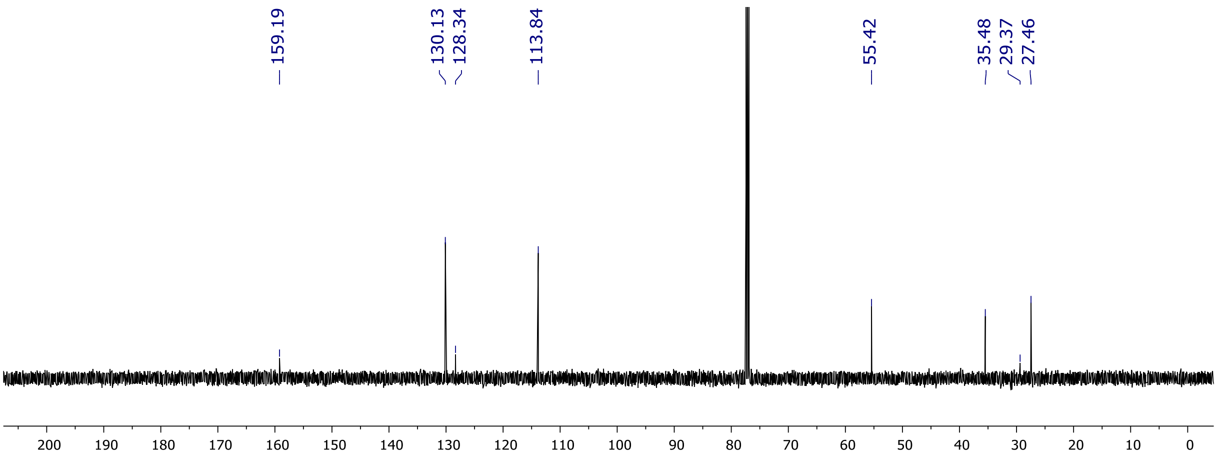
159.19
130.13
128.34
113.84
55.42
35.48
29.37
27.46
200 190 180 170 160 150 140 130 120 110 100 90 80 70 60 50 40 30 20 10 0

5B

^{1}H

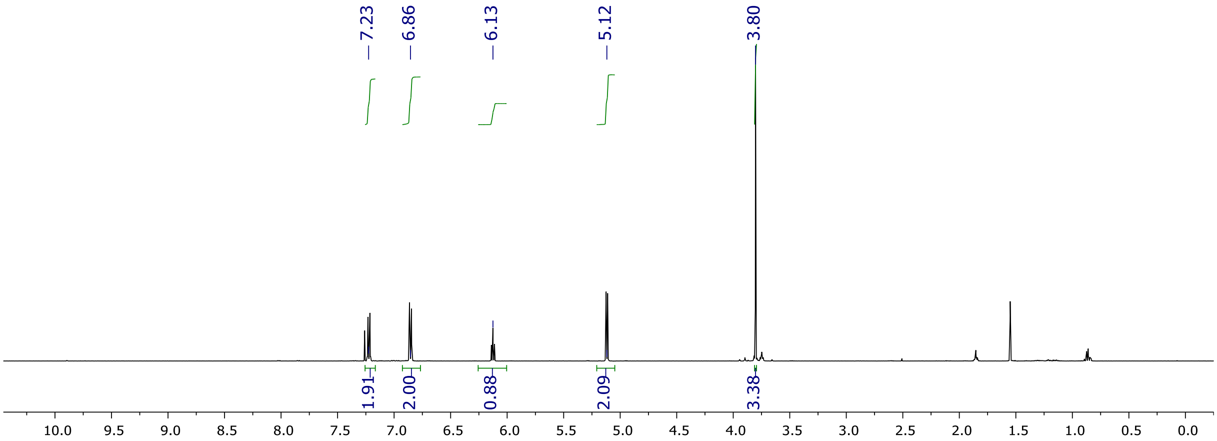

7.23
6.86
6.13
5.12
3.80
1.91
2.00
0.88
2.09
3.38
10.0 9.5 9.0 8.5 8.0 7.5 7.0 6.5 6.0 5.5 5.0 4.5 4.0 3.5 3.0 2.5 2.0 1.5 1.0 0.5 0.0

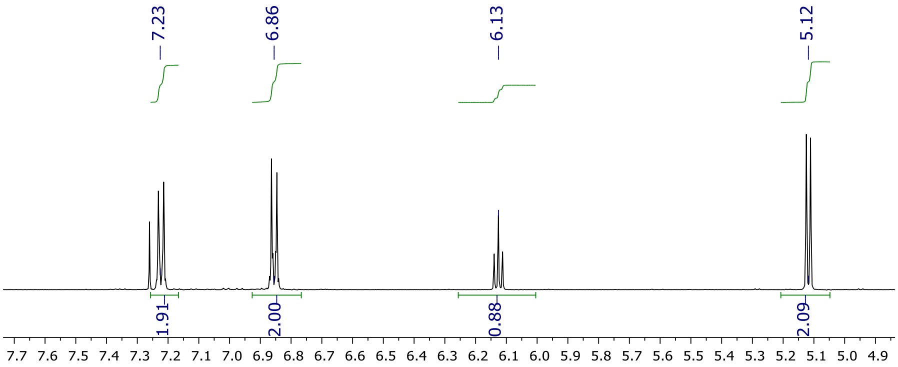

7.23
6.86
6.13
5.12
1.91
2.00
0.88
2.09
7.7 7.6 7.5 7.4 7.3 7.2 7.1 7.0 6.9 6.8 6.7 6.6 6.5 6.4 6.3 6.2 6.1 6.0 5.9 5.8 5.7 5.6 5.5 5.4 5.3 5.2 5.1 5.0 4.9

$^{13}C\{^{1}H\}$

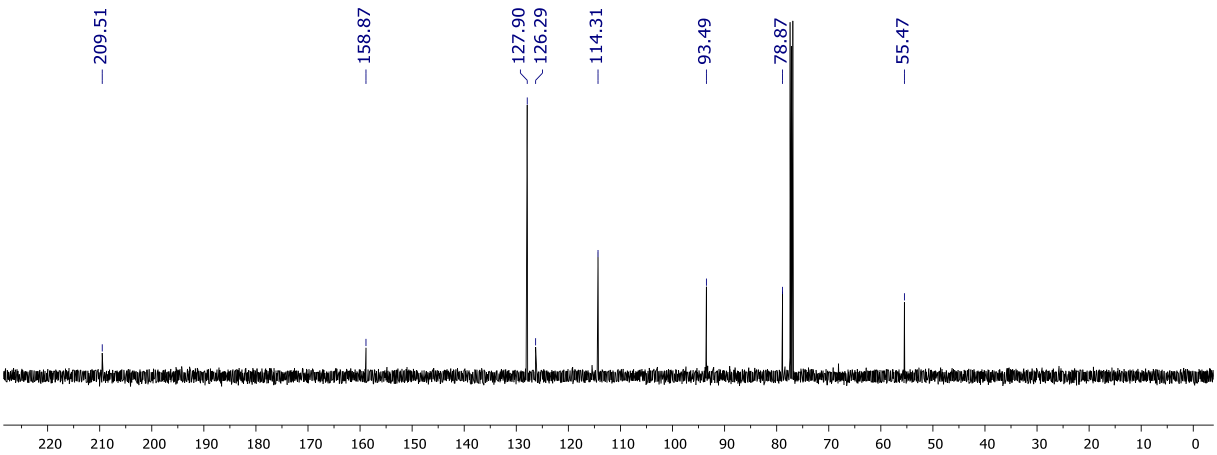

209.51
158.87
127.90
126.29
114.31
93.49
78.87
55.47
220 210 200 190 180 170 160 150 140 130 120 110 100 90 80 70 60 50 40 30 20 10 0

6A

^{1}H

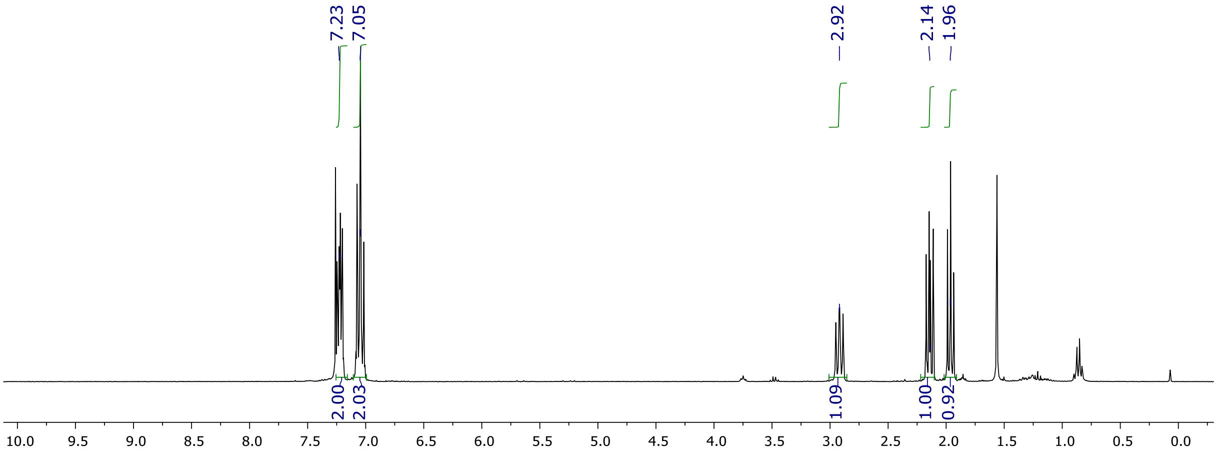

$^{13}C\{^{1}H\}$

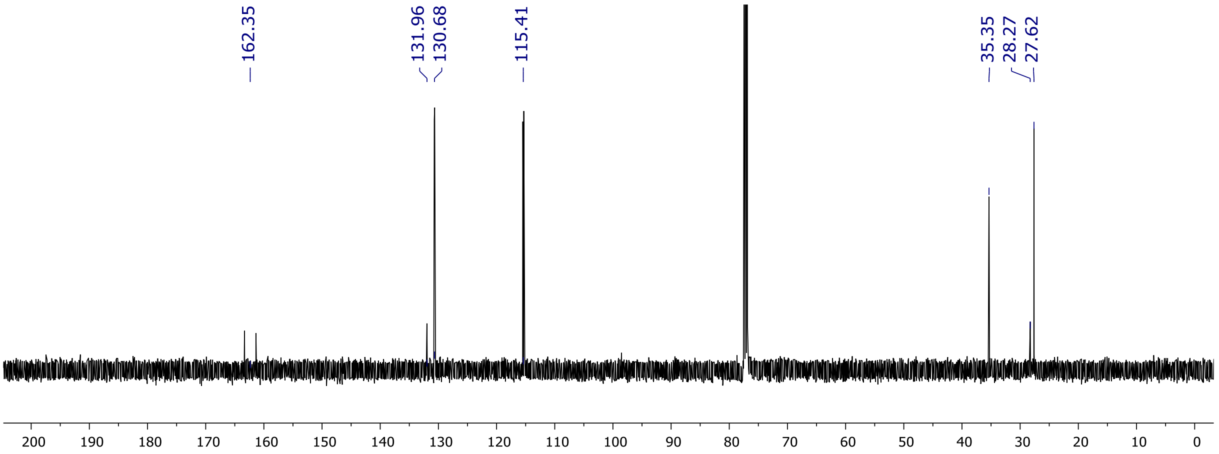

$^{19}F\{^{1}H\}$

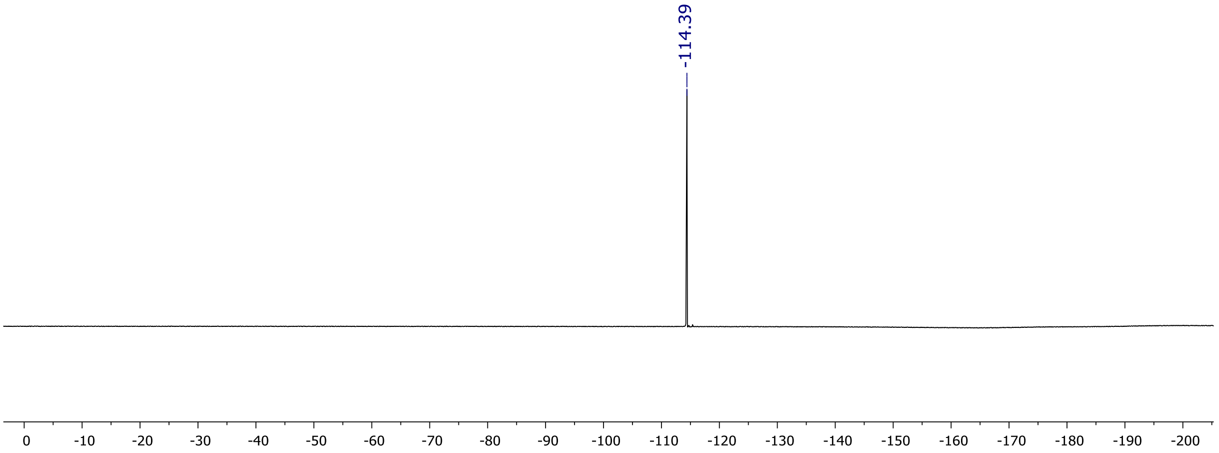

6B

^{1}H

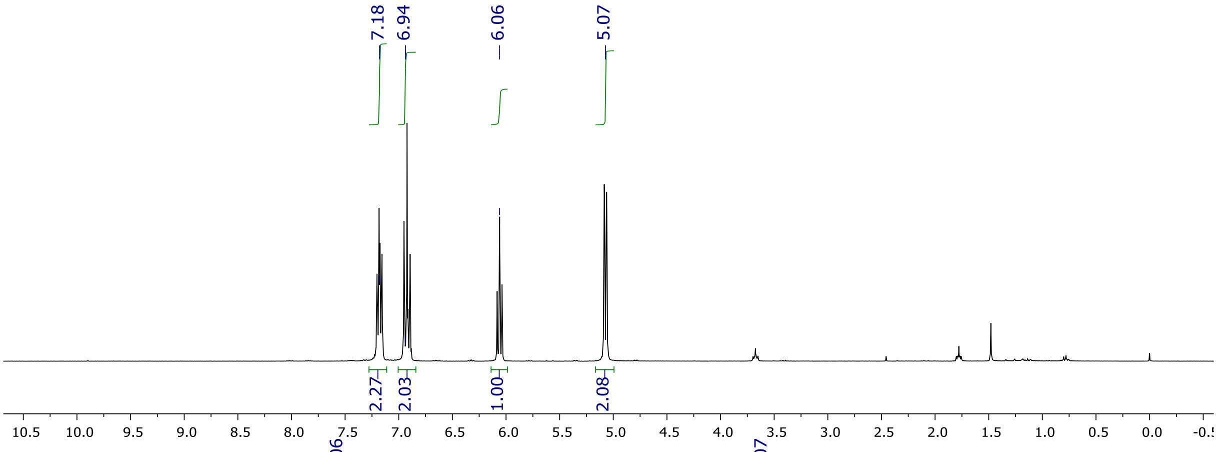
7.18
6.94
6.06
5.07
2.27
2.03
1.00
2.08

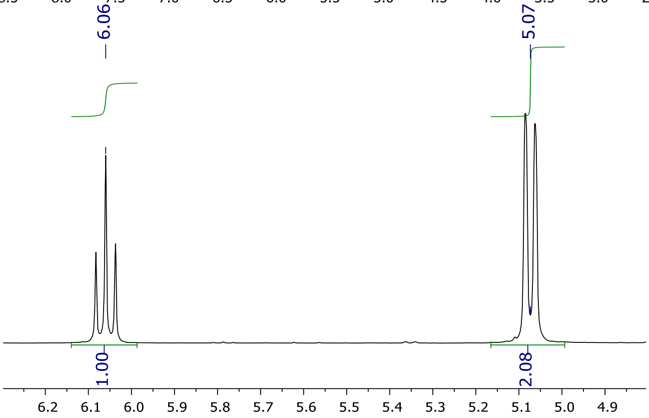
6.06
5.07
1.00
2.08

$^{13}C\{^{1}H\}$

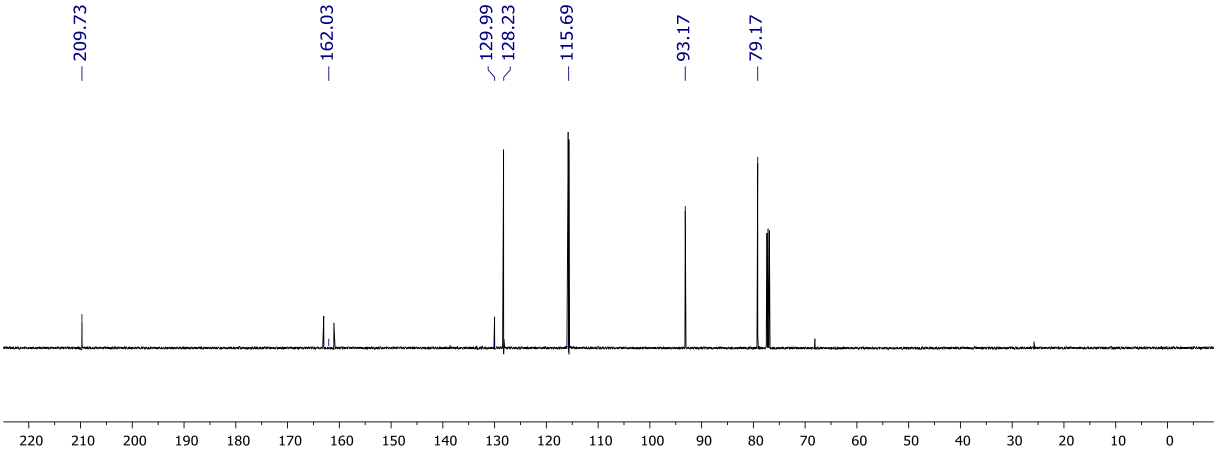
209.73
162.03
129.99
128.23
115.69
93.17
79.17

$^{19}F\{^{1}H\}$

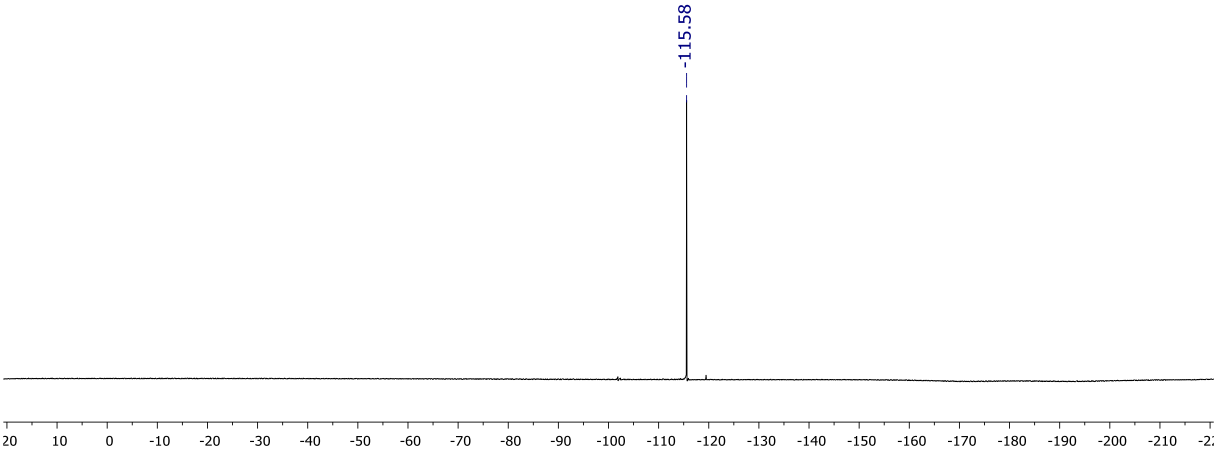
-115.58
20 10 0 -10 -20 -30 -40 -50 -60 -70 -80 -90 -100 -110 -120 -130 -140 -150 -160 -170 -180 -190 -200 -210

7A

^{1}H

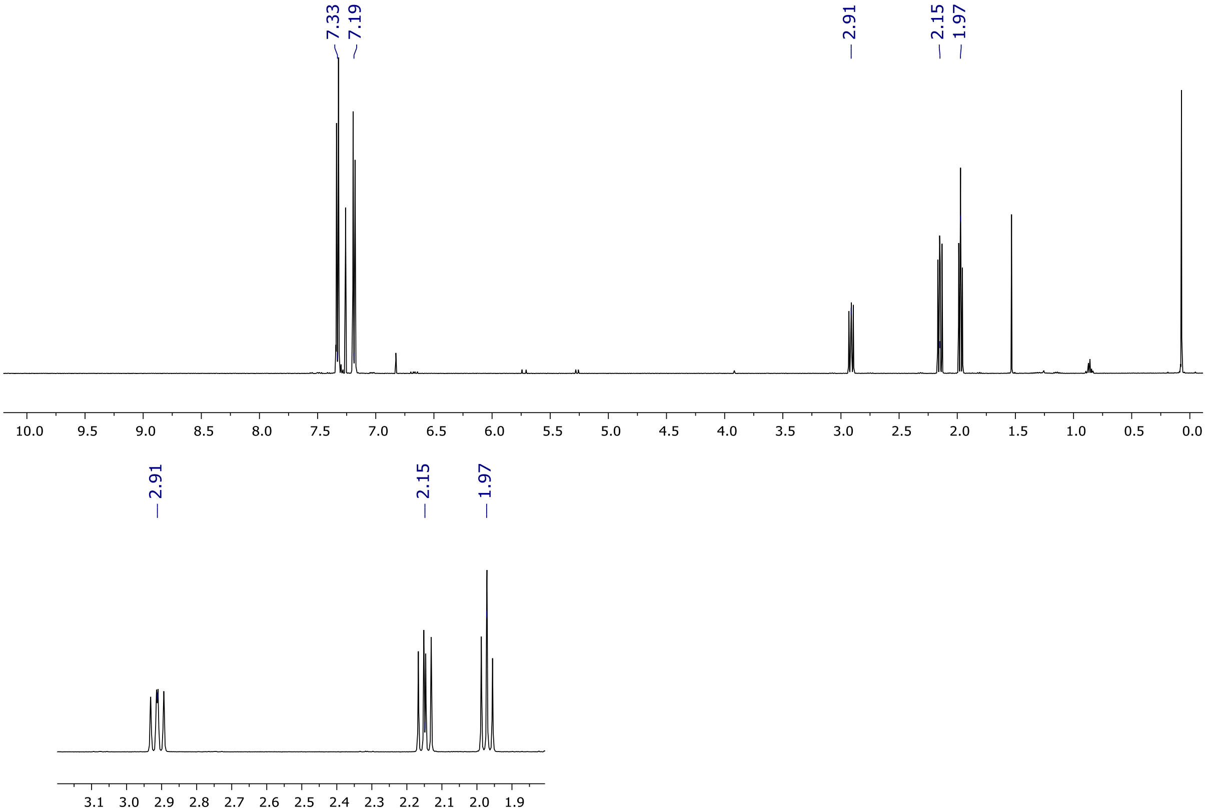
7.33
7.19
2.91
2.15
1.97
10.0 9.5 9.0 8.5 8.0 7.5 7.0 6.5 6.0 5.5 5.0 4.5 4.0 3.5 3.0 2.5 2.0 1.5 1.0 0.5 0.0
2.91
2.15
1.97
3.1 3.0 2.9 2.8 2.7 2.6 2.5 2.4 2.3 2.2 2.1 2.0 1.9

$^{13}C\{^{1}H\}$

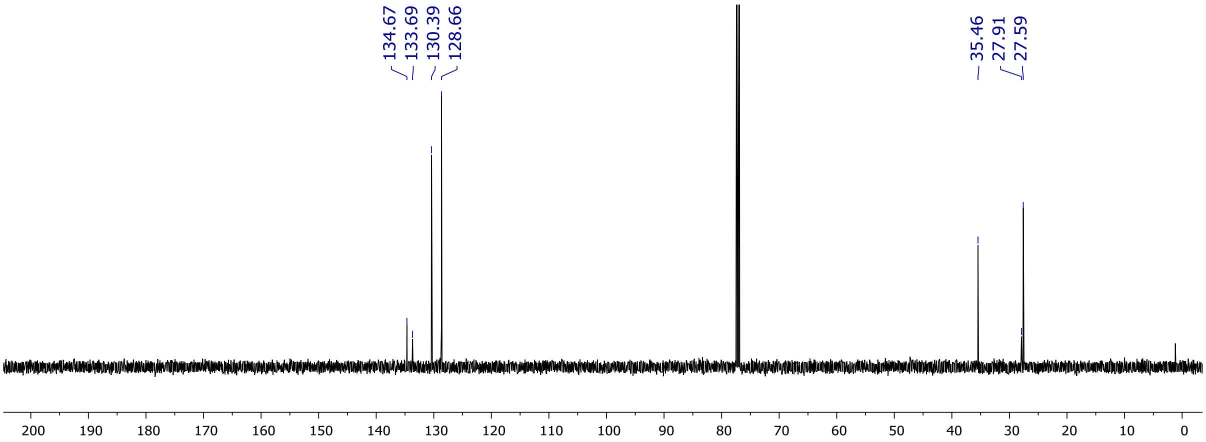

134.67
133.69
130.39
128.66
35.46
27.91
27.59

7B

^{1}H

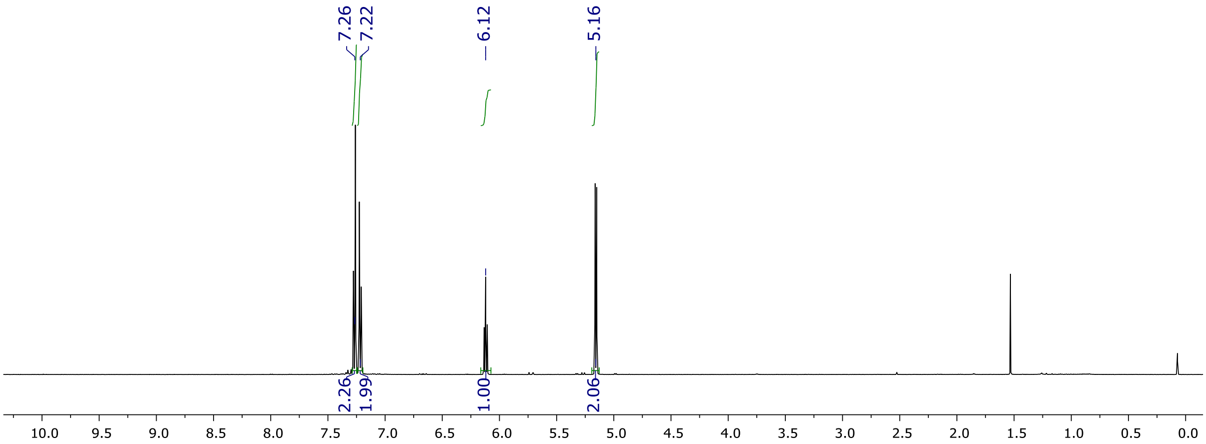

7.26
7.22
6.12
5.16
2.26
1.99
1.00
2.06

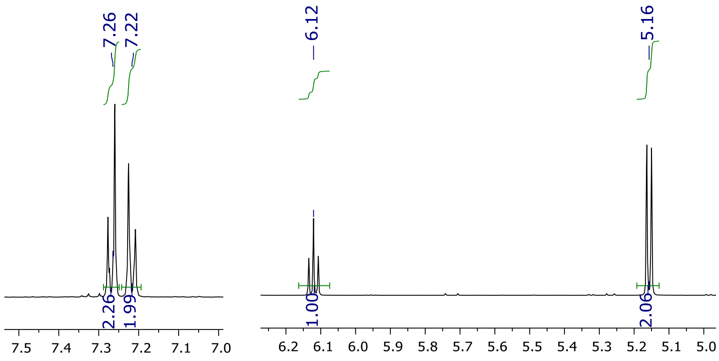

7.26
7.22
6.12
5.16
2.26
1.99
1.00
2.06

$^{13}C\{^{1}H\}$

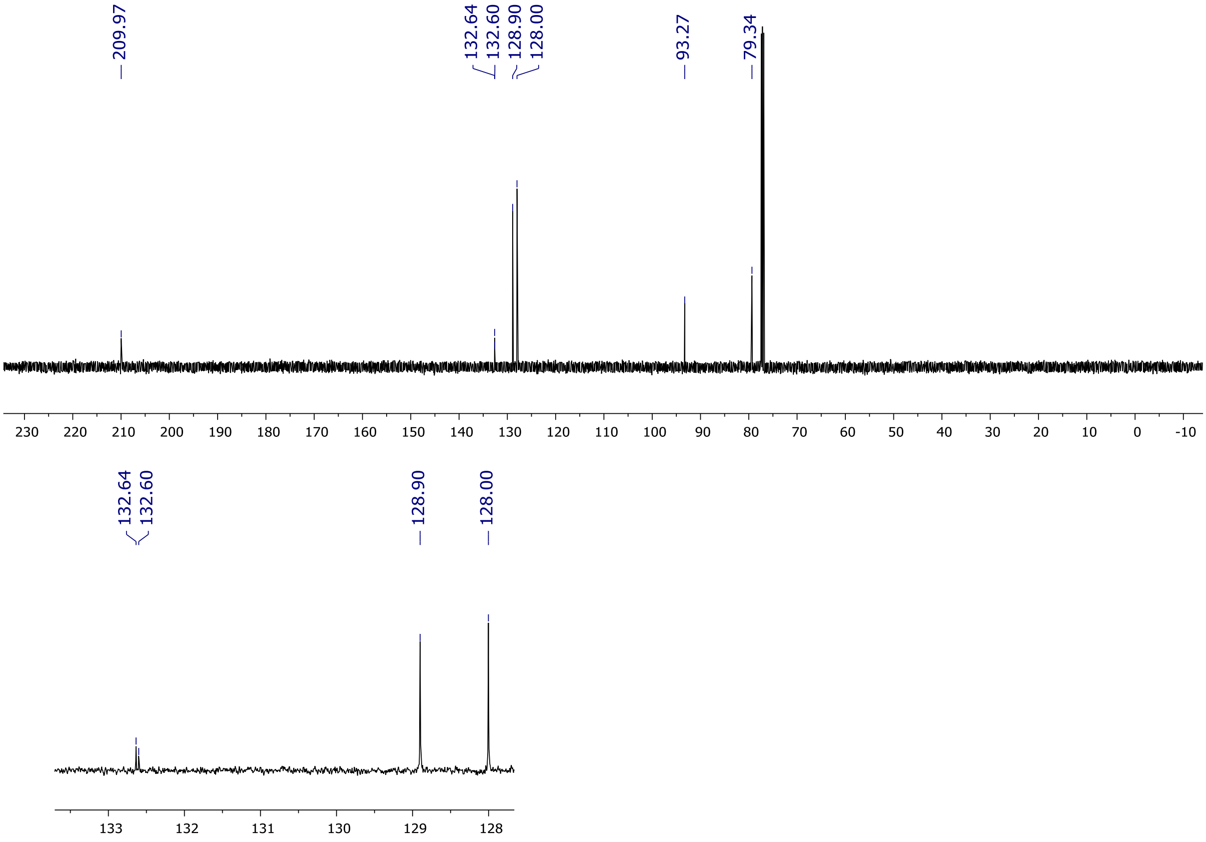

209.97
132.64
132.60
128.90
128.00
93.27
79.34
230 220 210 200 190 180 170 160 150 140 130 120 110 100 90 80 70 60 50 40 30 20 10 0 -10
132.64
132.60
128.90
128.00
133 132 131 130 129 128

CD_3PPh_3I

^1H

Some residual THF can be observed (3.76 & 1.85 ppm respectively)

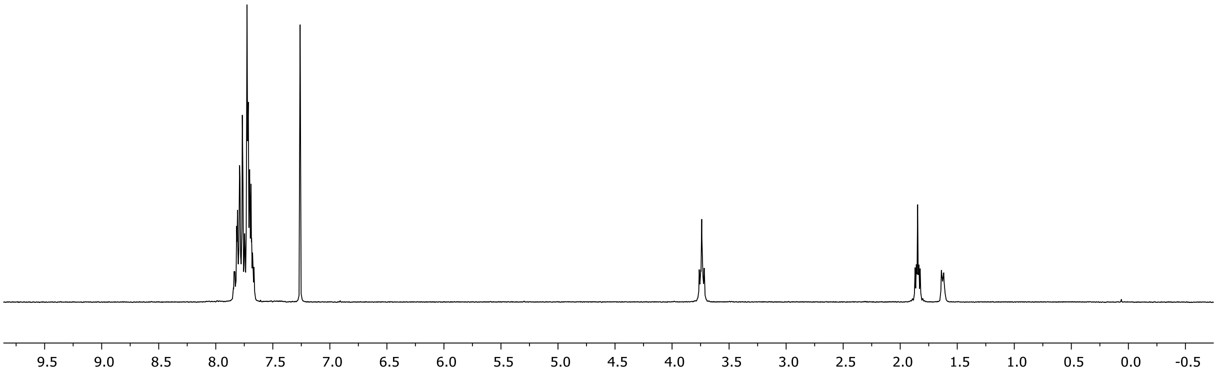

$^{13}C\{^1H\}$

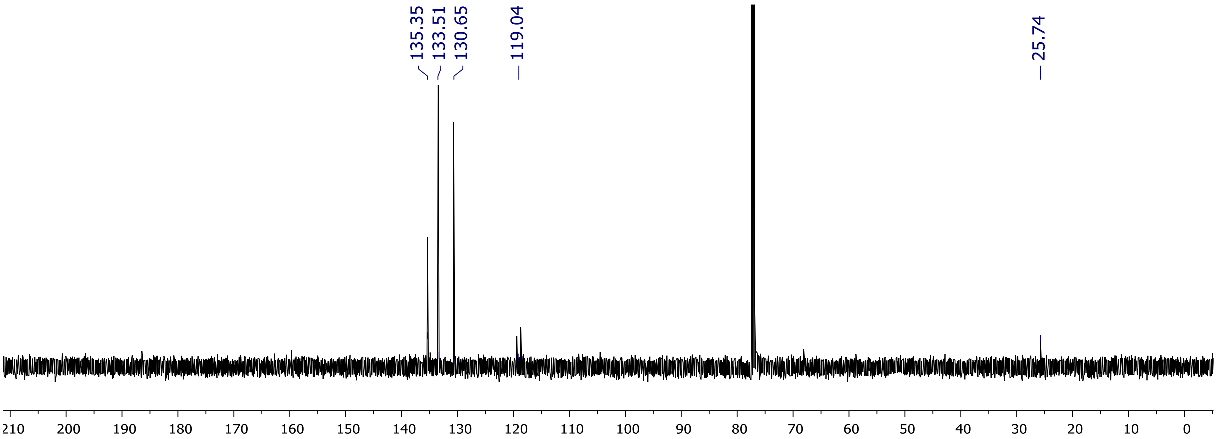

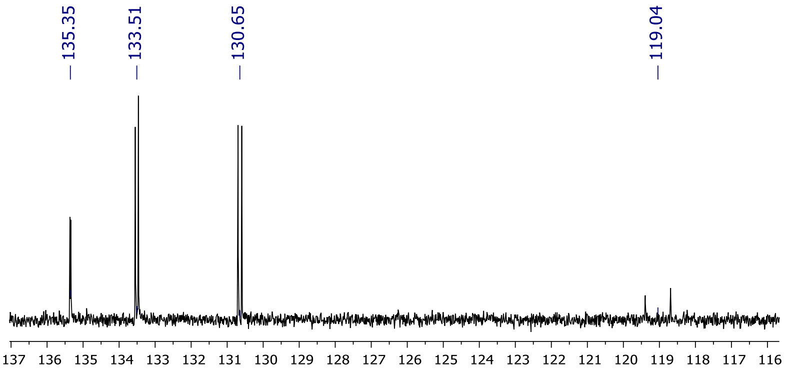

^{2}H ($CDCl_3$ peak also observed)

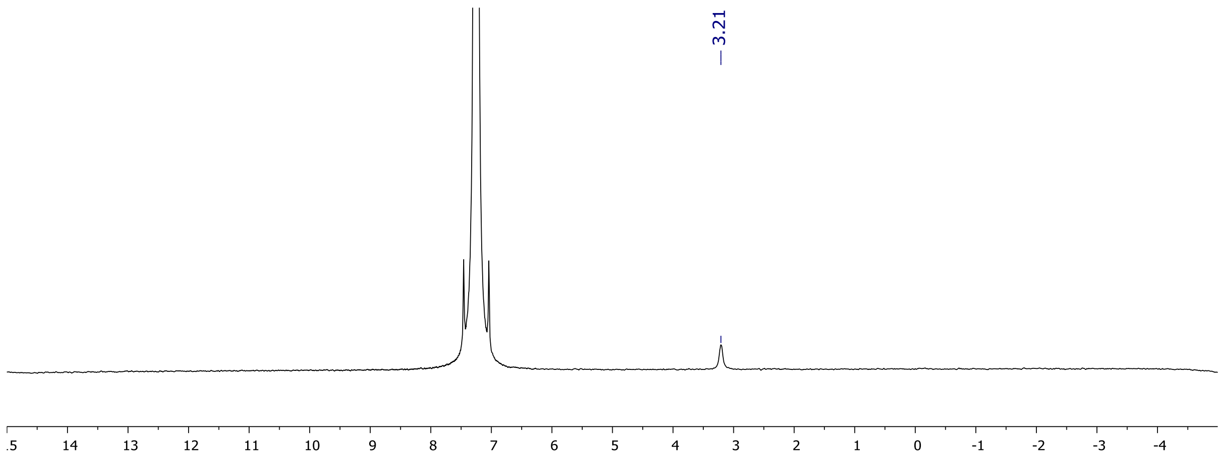

$^{31}P\{^{1}H\}$

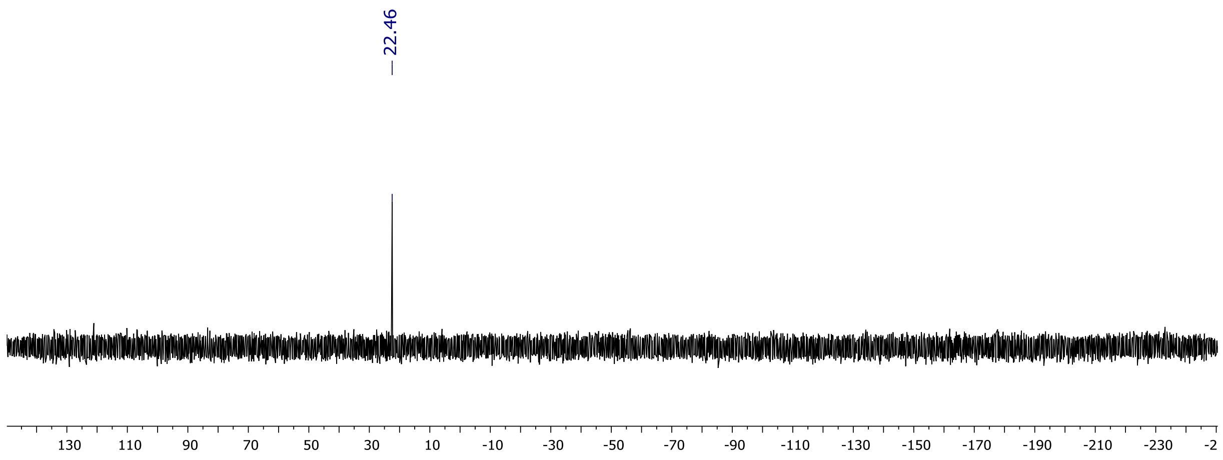

(2,2-dibromocyclopropyl-3,3-d_2)benzene

^{1}H

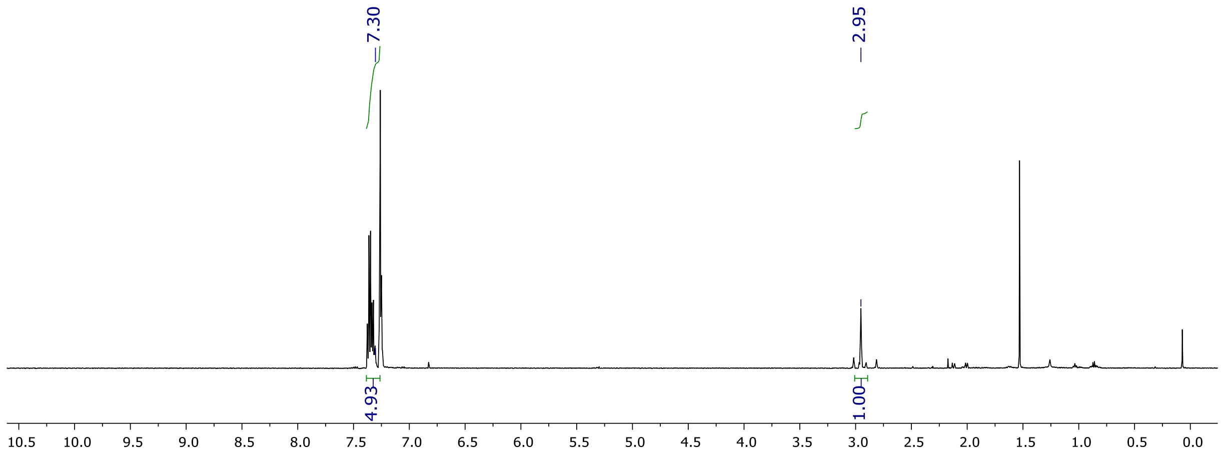

$^{13}C\{^1H\}$

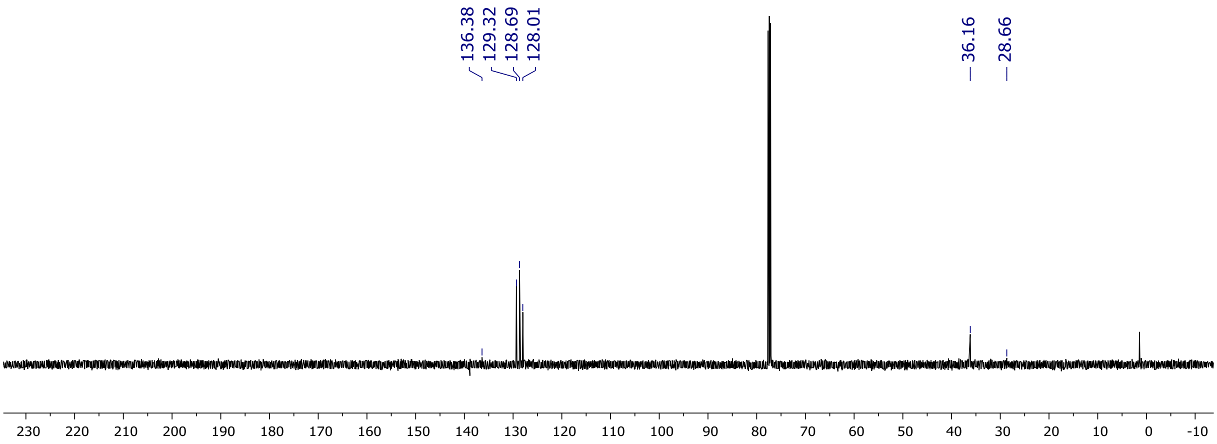
136.38
129.32
128.69
128.01
36.16
28.66
230 220 210 200 190 180 170 160 150 140 130 120 110 100 90 80 70 60 50 40 30 20 10 0 -10

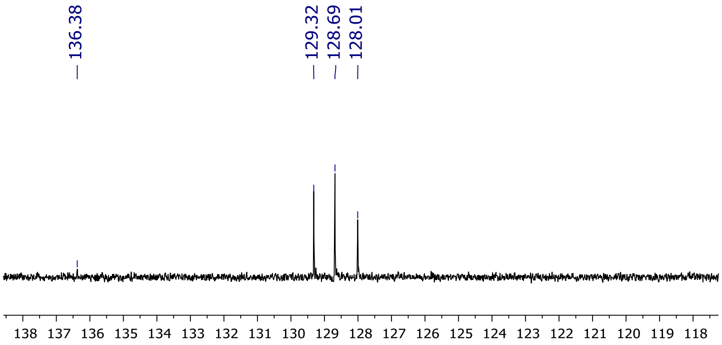
136.38
129.32
128.69
128.01
138 137 136 135 134 133 132 131 130 129 128 127 126 125 124 123 122 121 120 119 118

^2H

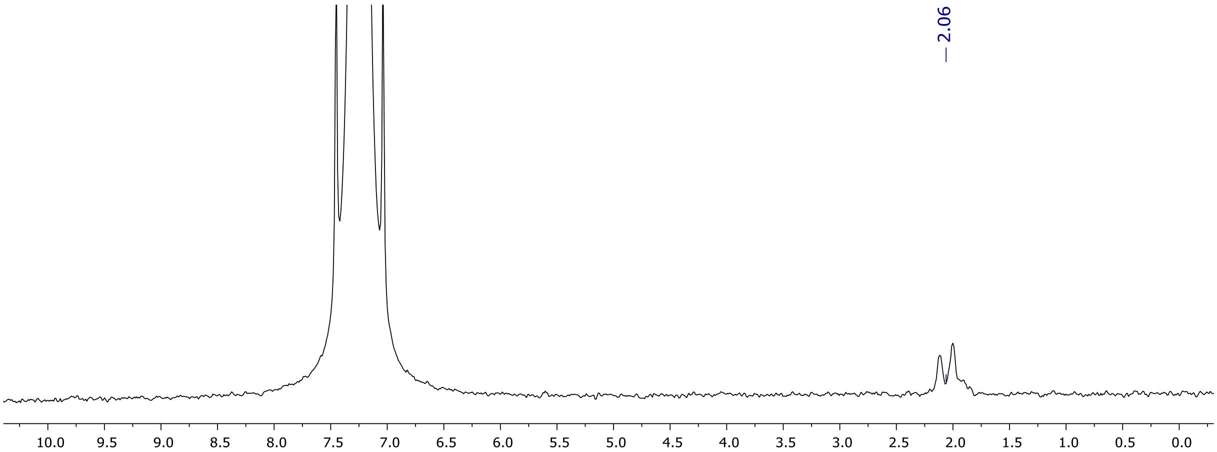
2.06
10.0 9.5 9.0 8.5 8.0 7.5 7.0 6.5 6.0 5.5 5.0 4.5 4.0 3.5 3.0 2.5 2.0 1.5 1.0 0.5 0.0

3,3-d$_2$-phenylallene

^{1}H

The residual (7%) protic peak is observed at 4.84 ppm. Some residual solvent was also present despite careful rotary evaporation

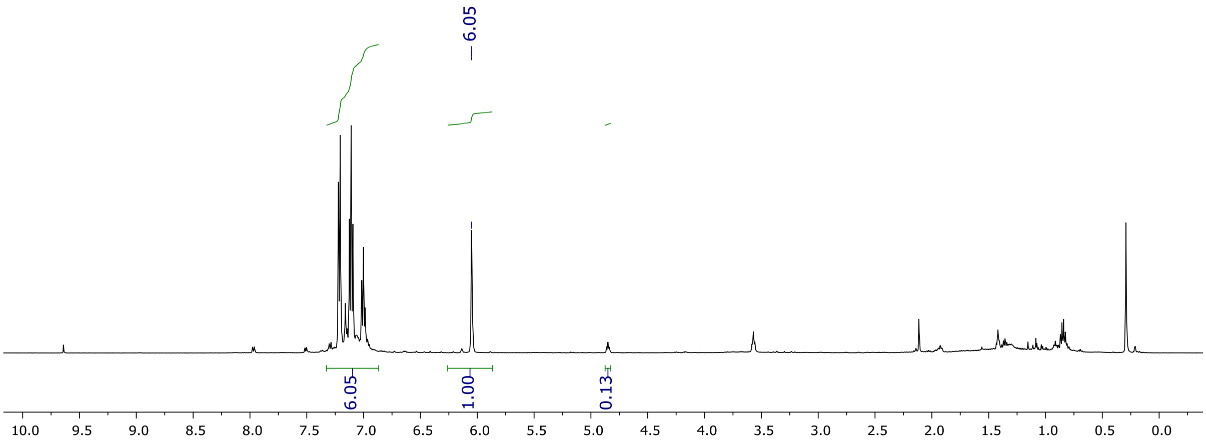

^{13}C{^{1}H}

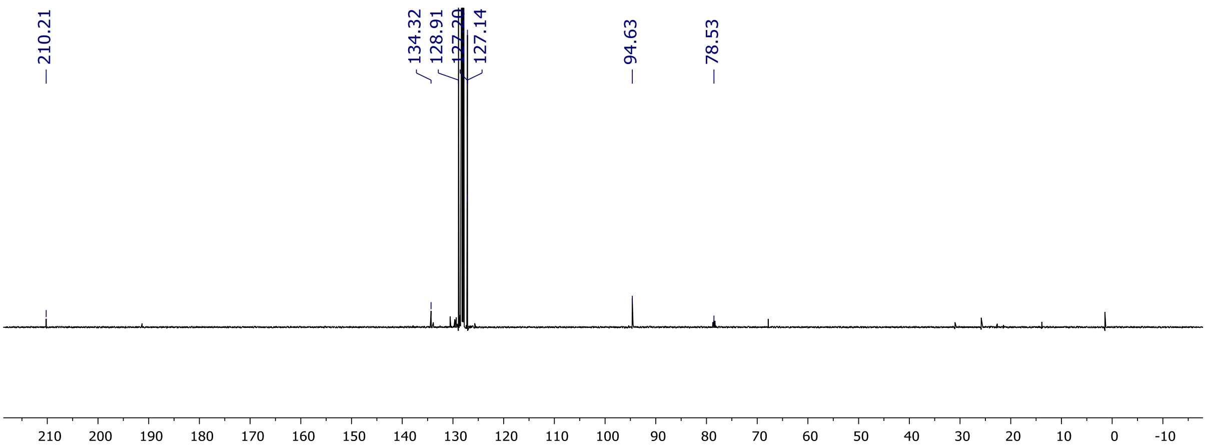

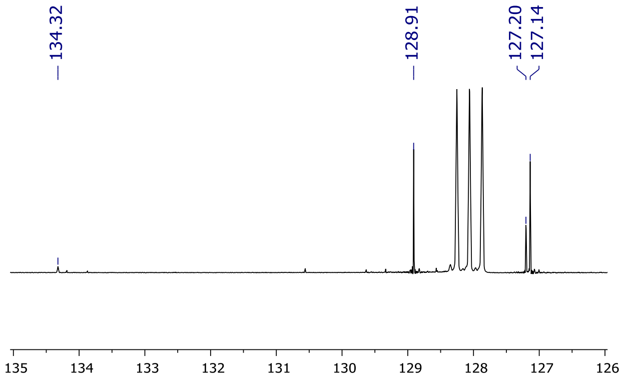

^{2}H

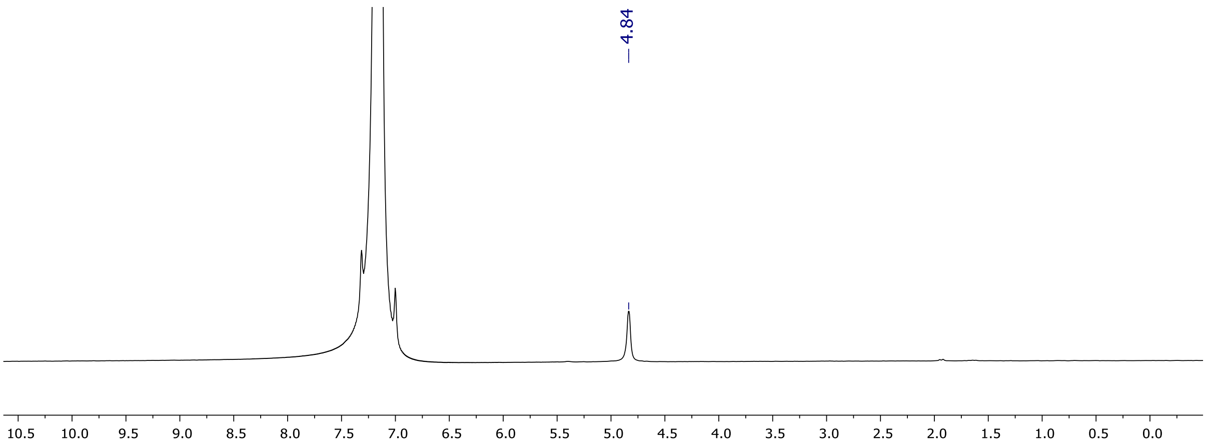

Poly(3,3-d$_2$)phenylallene

^{1}H

Internal standard peak at 3.31 ppm. 3,3-d$_2$-phenylallene observed at δ = 6.16, 4.87 ppm

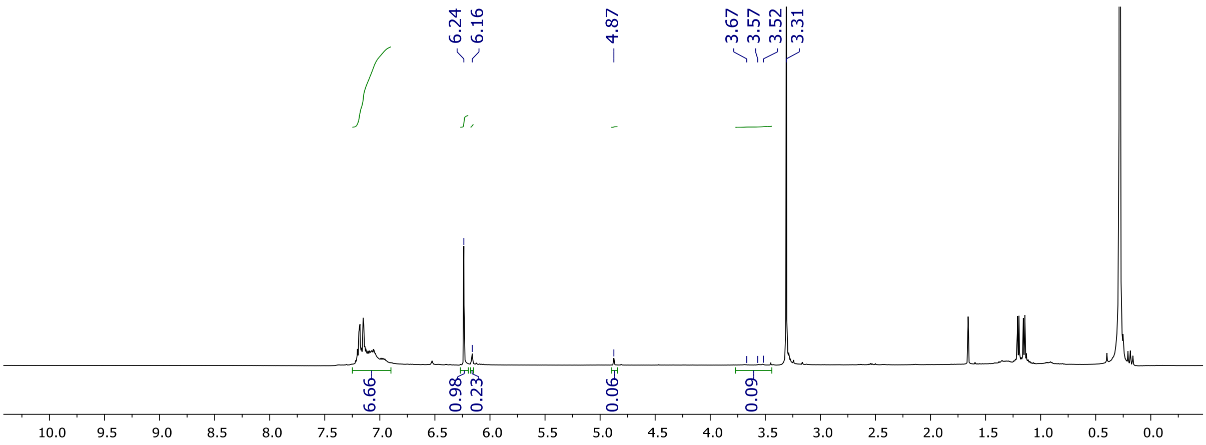

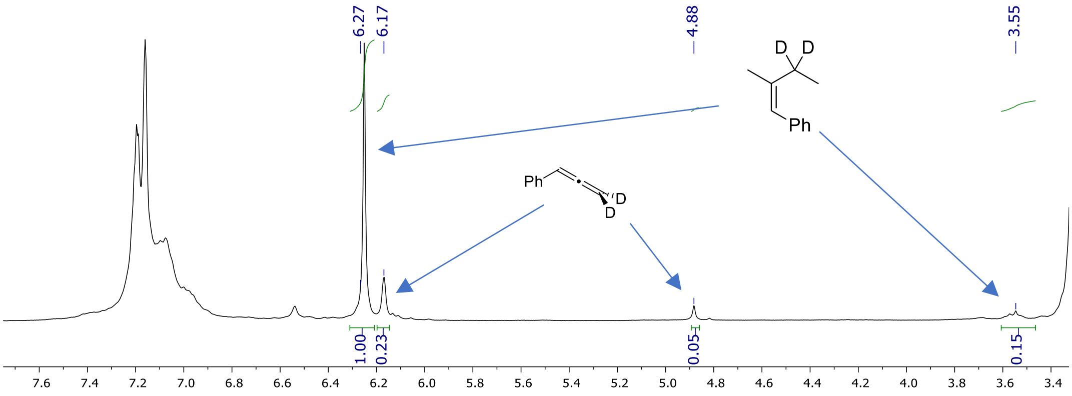

^2H

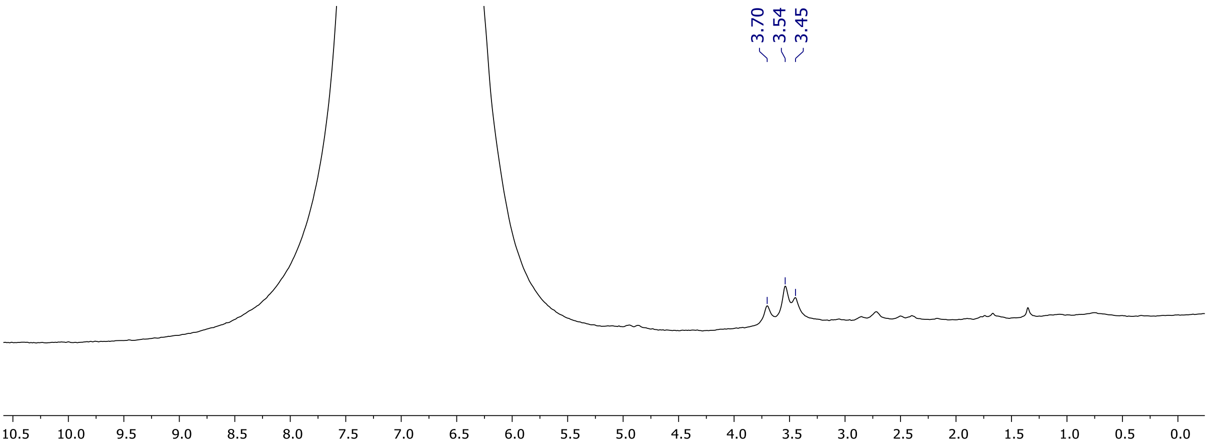

Radical Clock

^1H NMR of reaction mixture (distilled)

(Chloromethyl)cyclopropane peaks are indicated with ●, 1-butene with ●

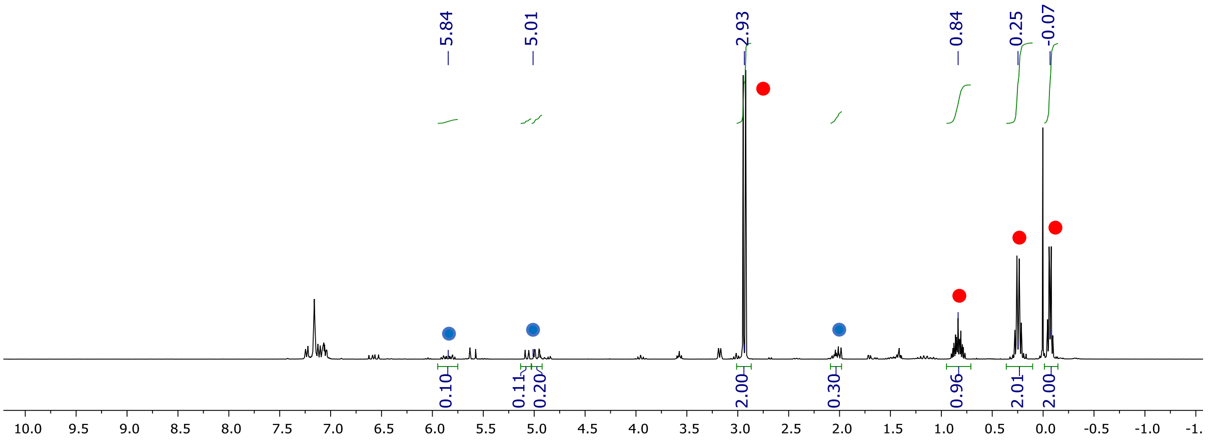

Polymers

Polyphenylallene (P1)

NMR

The major (2,3) polymer signals are denoted with ●, the minor (1,2) signals are denoted with ●

^{1}H

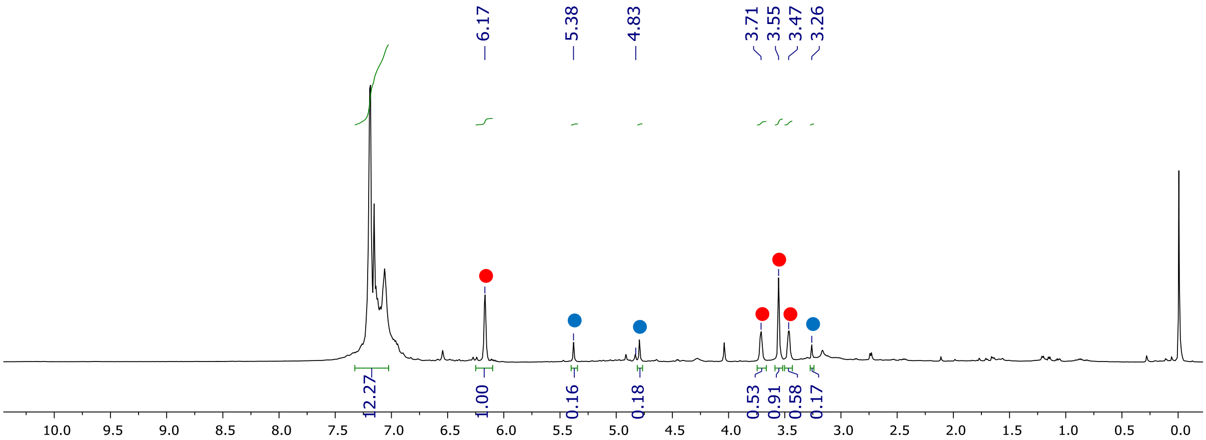

$^{13}C\{^{1}H\}$

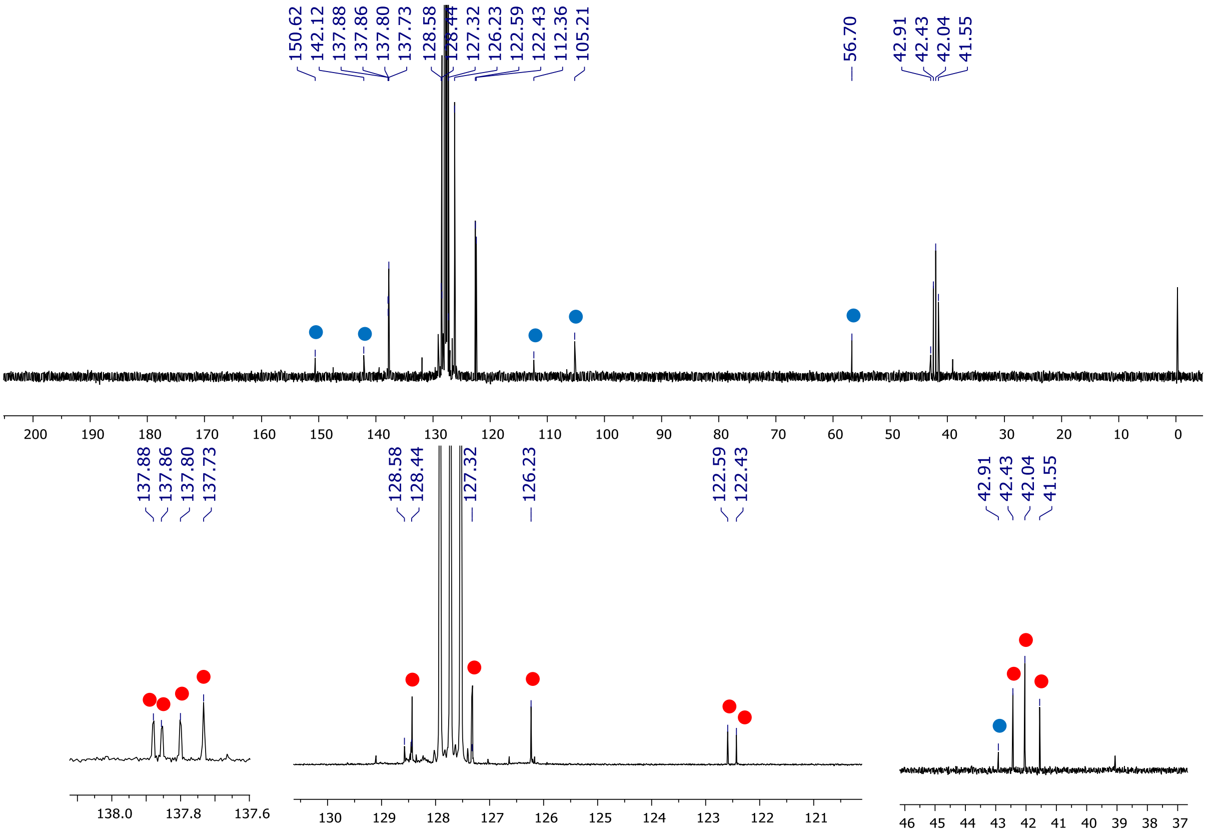

Summary of Correlating Peaks

Nuclei	^{1}H δ / ppm	$^{13}C\{^{1}H\}$ δ / ppm
HC_3 / C_3	3.72	41.5
	3.56	42.0
	3.47	42.4

HSQC of correlating area (500 MHz, C_6D_6)

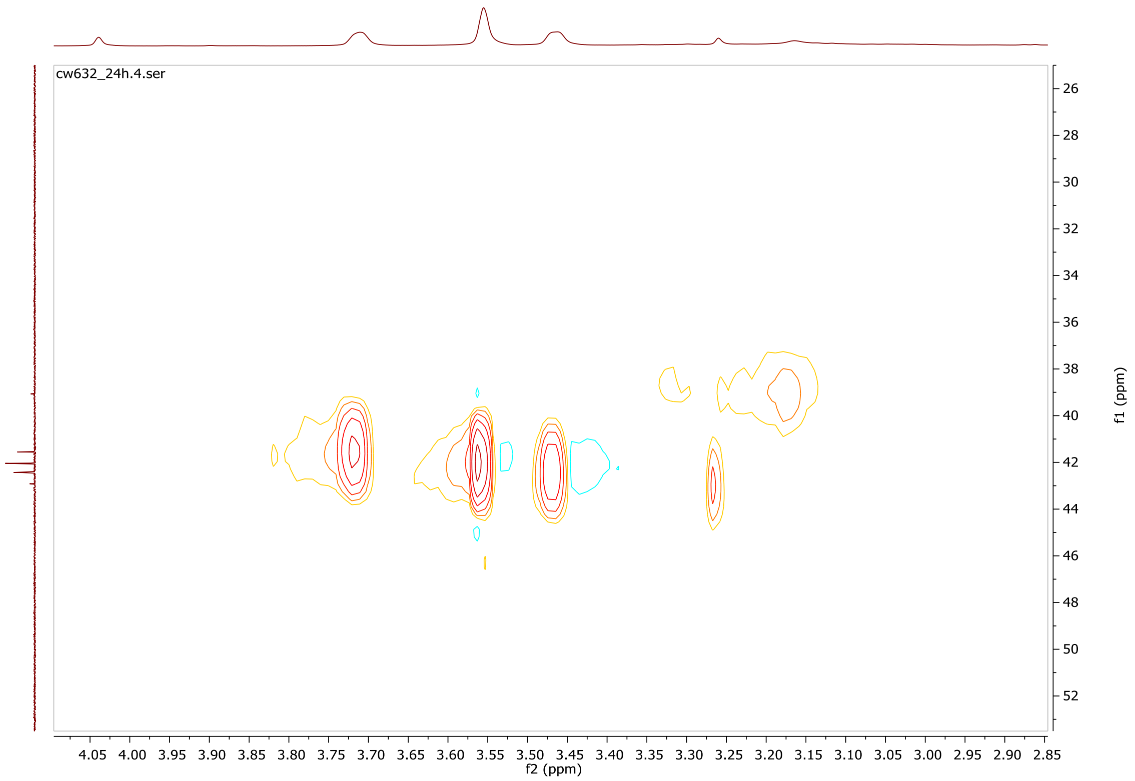

NOE NMR Data

nOe – 3.71 ppm peak

Some correlation with aryl and alkenyl protons

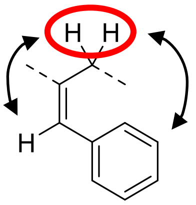

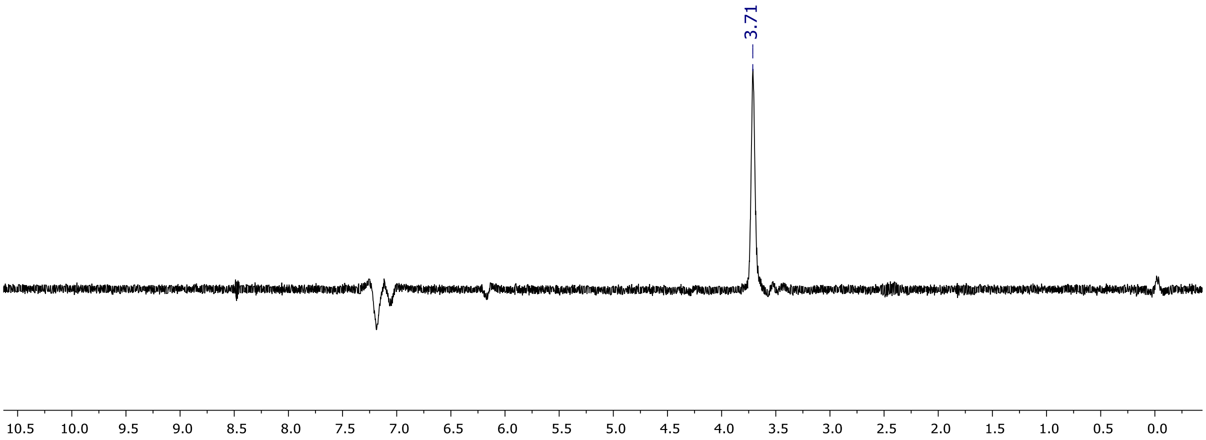

nOe – 3.55 ppm peak

Very little correlation

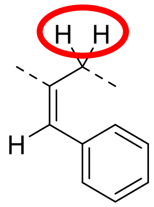

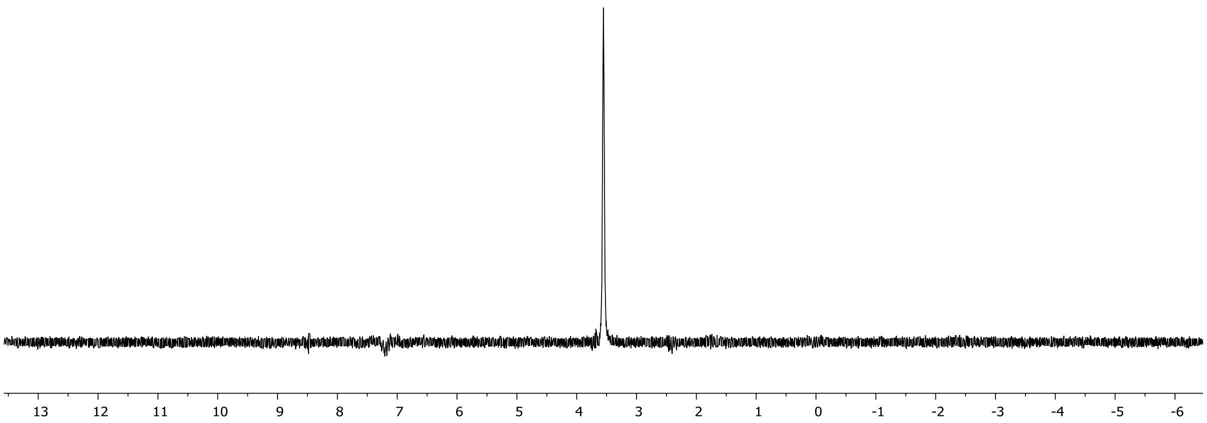

nOe – 3.46 ppm peak

Some correlation with aryl and alkenyl protons

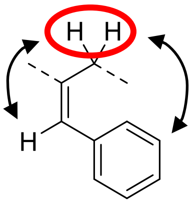

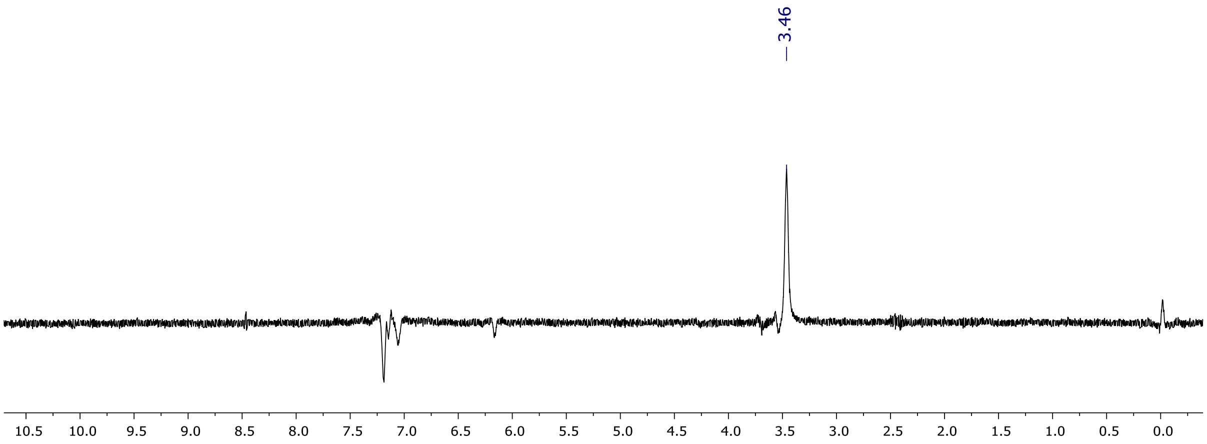

Visual Observations of **P1**

To confirm further on the Tg event observed for **P1** (and other polymers) a small amount of **P1** was heated using a Mettler Toledo FP82HT Hot Stage, and observed with a microscope camera as it was ramped from 25 °C to 200 °C. A change in form was observed in the range of 65 - 70°C, and images of the polymer before and after this transformation are shown below. The authors would like to thank Pollyanna Payne and Aneesa Al-Ani (both University of Bath) for assisting with the Hot Stage Experiments.

P1 below T_g

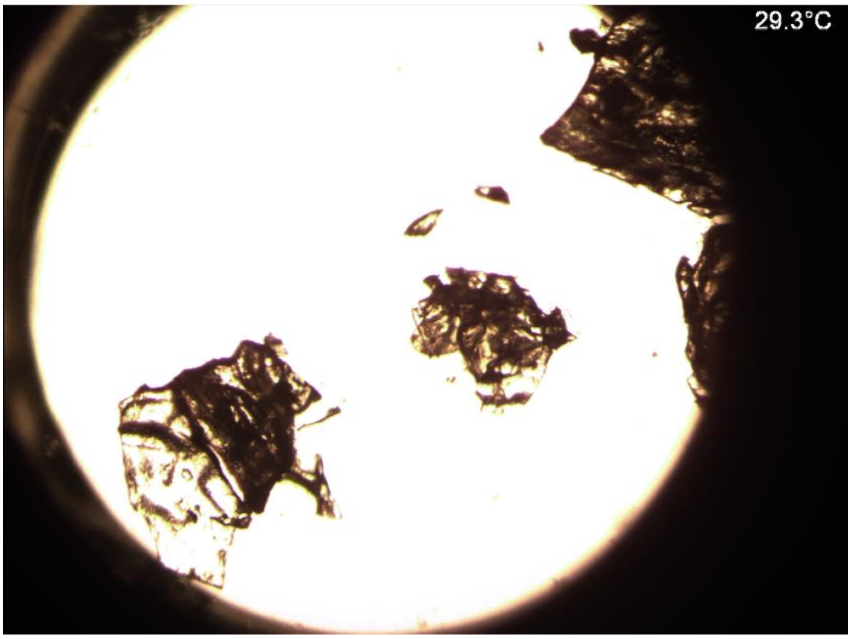

P1 above T_g

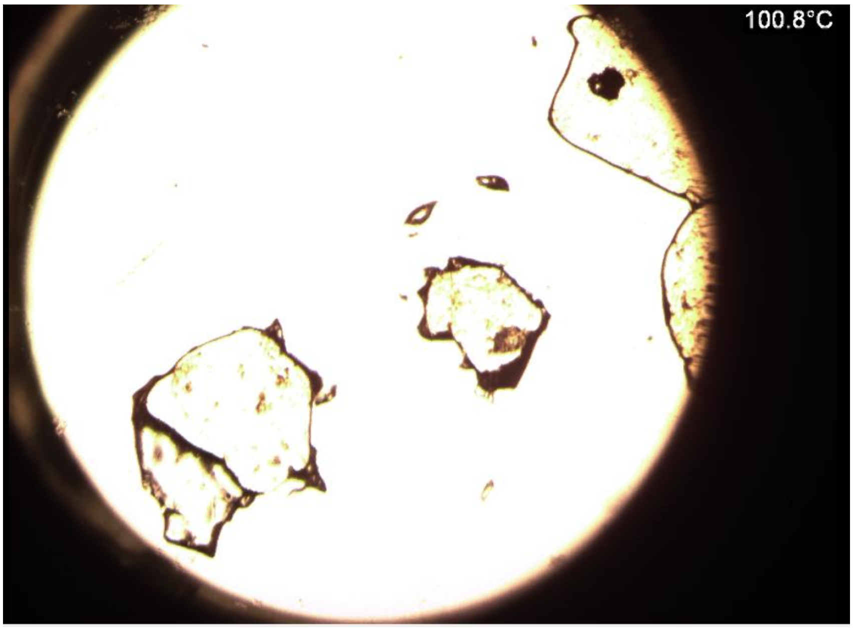

DSC Trace

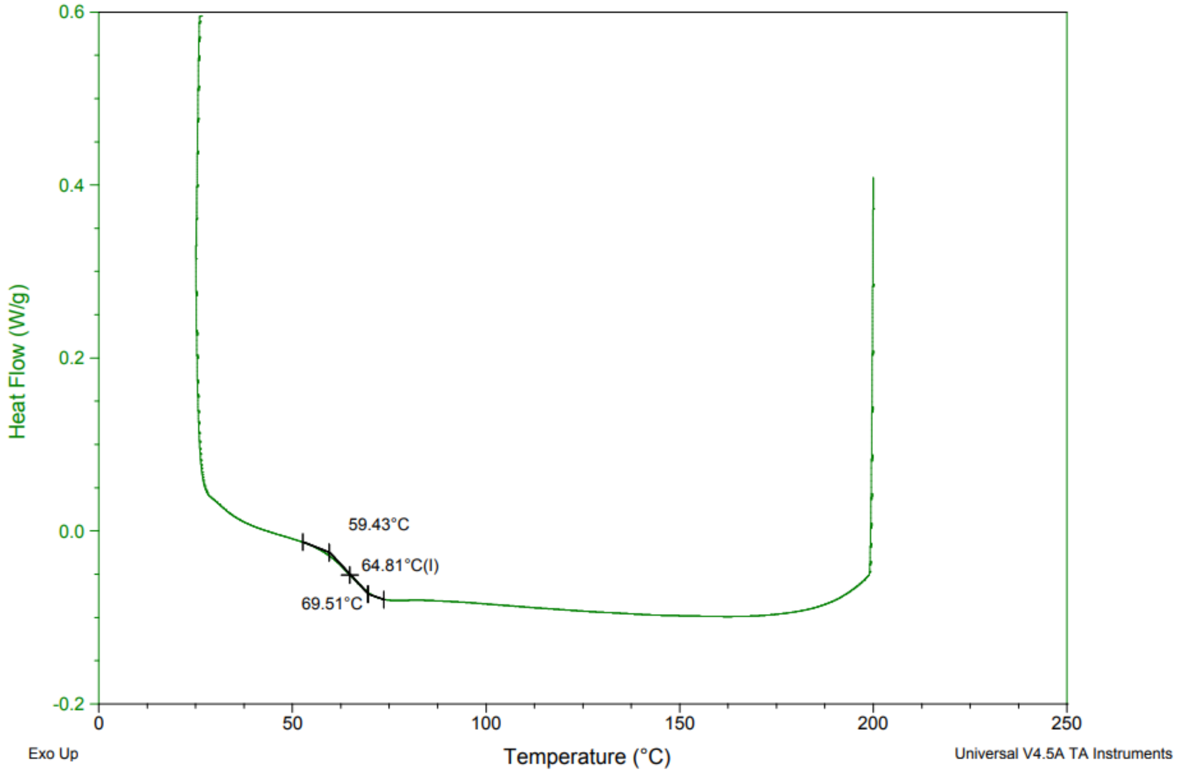

0.6
0.4
0.2
0.0
-0.2
Heat Flow (W/g)
59.43°C
64.81°C(I)
69.51°C
0
50
100
150
200
250
Exo Up
Temperature (°C)
Universal V4.5A TA Instruments

Agilent GPC/SEC Software
Sample Triple Analysis Report

Agilent Technologies

cw636

**Workspace Details**	
Workspace name	Polylactide
Location	C:\ProgramData\Agilent Technologies\GPC\Workspaces\Polylactide\
Comments	
Created by	Administrator at 13:44:31 on 15 June 2015
**Sample Properties**	
Sample name	cw636
File name	ICF_22_07_2020-0005.sample
Collected by	GPC at 13:33:15 on 22 July 2020
Instrument name	Instrument 1
**System Calibration Used**	
Created by	Administrator at 13:44:30 on 15 June 2015
Last modified by	Administrator at 13:44:30 on 15 June 2015
Comments	
Sample file used for calibration	ICF_6_18_2015-0002.sample
K (RI)	(RI) 750.958
K (LS 90°)	(RI) 30220.130
K (LS 15°)	(RI) 9870.135
K (VS DP)	(RI) 1.107
**System IDD Used**	
Last modified by	Administrator at 15:52:14 on 18 June 2015
Comments	
Reference detector	LS 90°
IDD Light scatterer (secs)	0.0
IDD RI (secs)	-3.0
IDD Viscometer (secs)	-14.0

Analyst: .... **Date:** ....

Checked By: .... **Date:** ....

Agilent GPC/SEC Software A.02.01 [9]   Page 1 of 5

Generated by GPC at 16:04 on 22 July 2020

Agilent GPC/SEC Software
Sample Triple Analysis Report

Agilent Technologies

Processing Parameters

Method	Last modified by Administrator at 13:44:30 on 15 June 2015
Concentration Detector Used in Analysis	RI
Injection volume (µL)	100.00
Flow rate (mL/min)	1.00
Concentration options	Calculate Sample Properties from Entered Sample Concentration
Entered sample concentration (mg/mL)	1.000
Calculated dn/dc (mL/g)	0.135
Calculated Ext Coeff ([(mg/mL)$^{-1}$] [cm^{-1}])	0.000
MW calculation method	Use all angles
Log Mi-v-RT curve fit options	Set the fit limits using the limits at peak width of 10 %.
Polynomial curve fit order	1
Use Constant Inlet Pressure	No
Flory-Fox	2.86e+021
DP Multiplier (mV to Pa)	1.0000
IP Multiplier (mV to kPa)	0.1000
Use IV To Calculate Rg	No

MW Ranges Method

Calculate MW Ranges No

Percentage Fractions Method

Calculate Percentage Fractions No

Results

Analysed by GPC at 16:03:58 on 22 July 2020

Comments

Peak Results

	Detector Type	Peak Max RT (mins)	Bulk MW (g/mol)	Bulk IV (dL/g)	Peak Height (mV)	Peak Height (%)	Peak Area (mV.s)	Area (%)	Conc. (mg/mL)
Peak 1	RI	10.01667	4414581	2.535040	0.221	4.88	9.350	1.54	0.015
Peak 2	RI	12.63333	215974	0.273440	4.304	95.12	598.239	98.46	0.985
Recovery (%)	100.00								

Molecular Weight Averages

Peak	Mp (g/mol)	Mn (g/mol)	Mw (g/mol)	Mz (g/mol)	Mz+1 (g/mol)	Mv (g/mol)	PD
Peak 1	4244466	3417886	3994189	4641628	5295085	4547878	1.169
Peak 2	228063	189161	223507	258809	292205	252887	1.182

Rg Results

Peak	Slope	Intercept	Rgp (nm)	Rgn (nm)	Rgw (nm)	Rgz (nm)	Rgz+1 (nm)
Peak 1	0.1204	0.9497	55.95	54.51	55.55	56.56	57.46
Peak 2	-0.7447	5.52	33.88	38.94	34.40	30.84	28.18

Analyst: .... **Date:** ....

Checked By: .... **Date:** ....

Agilent GPC/SEC Software
Sample Triple Analysis Report

Agilent Technologies

Rh Results

Peak	Slope	Intercept	Rhp (nm)	Rhn (nm)	Rhw (nm)	Rhz (nm)	Rhz+1 (nm)
Peak 1	0.5503	-1.911	54.49	48.37	52.70	57.24	61.54
Peak 2	0.5313	-1.836	10.24	9.26	10.13	10.96	11.70

IV Results

Peak	K ((10e-5) dL/g)	Alpha	IVp (dL/g)	IVn (dL/g)	IVw (dL/g)	IVz (dL/g)	IVz+1 (dL/g)
Peak 1	4.706	0.7108	2.419204	2.074013	2.316922	2.578011	2.831027
Peak 2	9.748	0.6511	0.300277	0.265851	0.296358	0.326050	0.352859

Peak Information

	Start (mins)	End (mins)
Baseline region 1	4.90000	7.46667
Baseline region 2	27.11667	30.95000
Peak 1	9.08333	10.53333
Peak 2	10.73333	14.88333

Peak Trace Information

Peak	Trace	Peak Max RT (mins)	Peak Area (mV.s)	Peak Height (mV)
Peak 1	RI	10.01667	9.350	0.221
Peak 1	VS DP	9.90000	226.977	4.177
Peak 1	VS IP	9.95000	10.900	0.482
Peak 1	LS 90°	9.91667	629.621	14.397
Peak 1	LS 15°	9.88333	350.987	7.093
Peak 2	RI	12.63333	598.239	4.304
Peak 2	VS DP	12.10000	1566.272	11.134
Peak 2	VS IP	11.81667	27.220	0.508
Peak 2	LS 90°	12.01667	2920.382	25.514
Peak 2	LS 15°	11.98333	1119.828	8.782

Log Mi Curves

Peak	Start (mins)	End (mins)	Polynomial Degree	Coeff a	Coeff b	Coeff c	Coeff d	Coeff e	Coeff f	L Point Y/N
Peak 1	9.5500	10.5167	1	13.444498	-0.68394732	0	0	0	0	N
Peak 2	11.0500	14.8667	1	7.8958874	-0.20114925	0	0	0	0	N

Structural Plot Region

Peak	Start (mins)	End (mins)
Peak 1	9.5500	10.5167
Peak 2	11.0500	14.8667

Analyst: .... **Date:** ....

Checked By: .... **Date:** ....

Agilent GPC/SEC Software
Sample Triple Analysis Report

Agilent Technologies

Chromatogram Plot

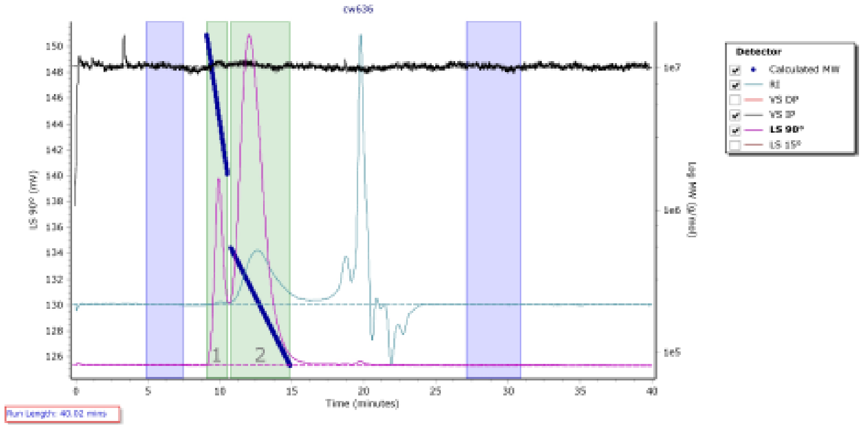

Distribution Plot

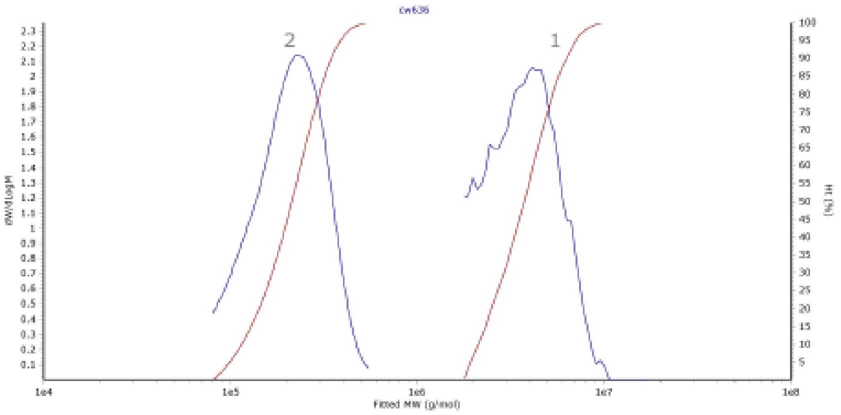

Analyst: .... **Date:** ....

Checked By: .... **Date:** ....

Agilent GPC/SEC Software A.02.01 [9] Page 4 of 5

Generated by GPC at 16:04 on 22 July 2020

Agilent GPC/SEC Software
Sample Triple Analysis Report

Agilent Technologies

Conformation Plot

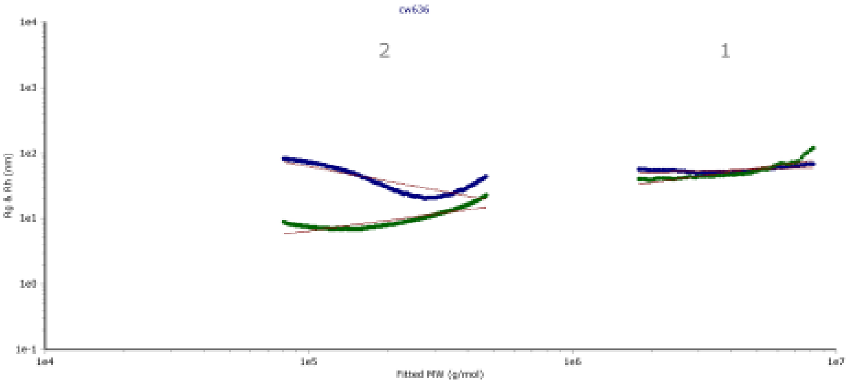

Peak 1 Rg (LS): Log Rg = (0.12) x Log MW + (0.95), Log Rh = (0.55) x Log MW + (-1.91)
Peak 2 Rg (LS): Log Rg = (-0.74) x Log MW + (5.52), Log Rh = (0.53) x Log MW + (-1.84)

Mark-Houwink Plot

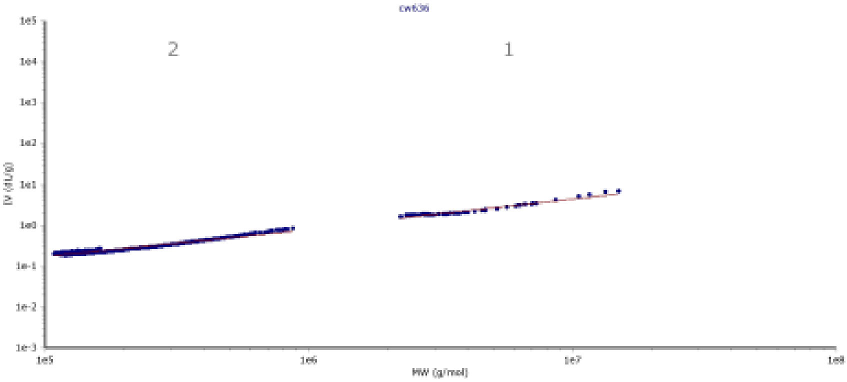

Peak 1: K = ((10e-5) dL/g) 4.71, Alpha = 0.71
Peak 2: K = ((10e-5) dL/g) 9.75, Alpha = 0.65

Analyst: .... **Date:** ....

Checked By: .... **Date:** ....

Agilent GPC/SEC Software A.02.01 [9] Page 5 of 5

Generated by GPC at 16:04 on 22 July 2020

Poly(4-methylphenyl) allene (P2)

The major (2,3) polymer signals are denoted with ●, the minor (1,2) signals are denoted with ●

^{1}H

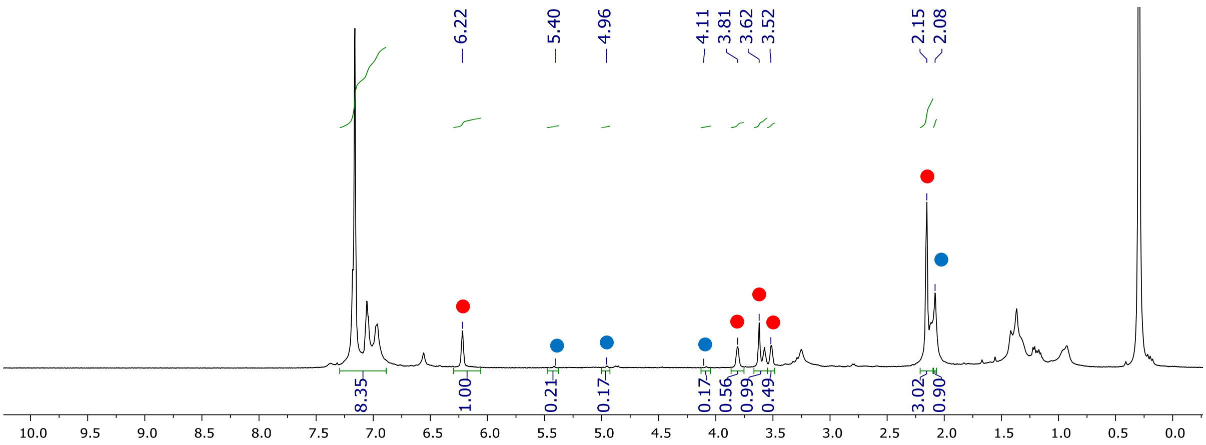

$^{13}C\{^{1}H\}$

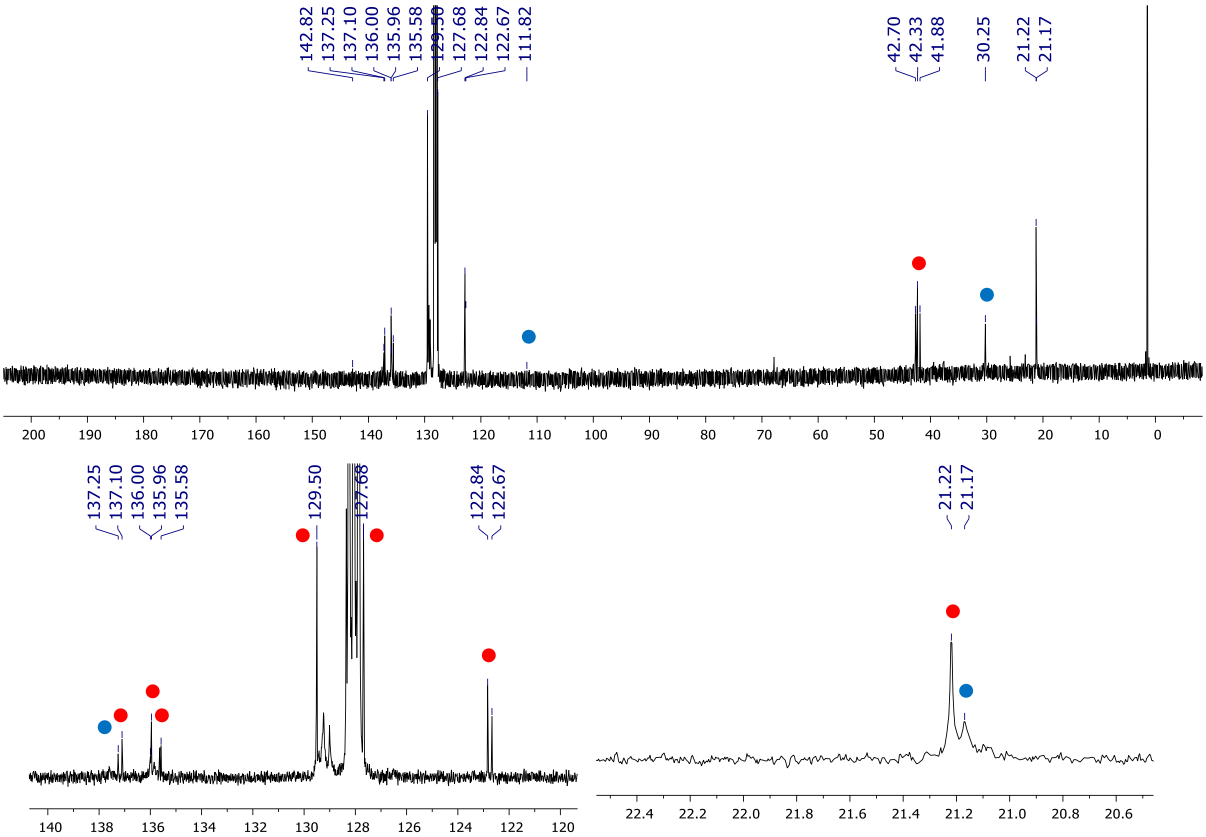

Summary of Correlating Peaks

Nuclei	^{1}H δ / ppm	$^{13}C\{^{1}H\}$ δ / ppm
HC_3 / C_3	3.81	41.9
	3.62	42.3
	3.52	42.7

HSQC of correlating area (500 MHz, C_6D_6)

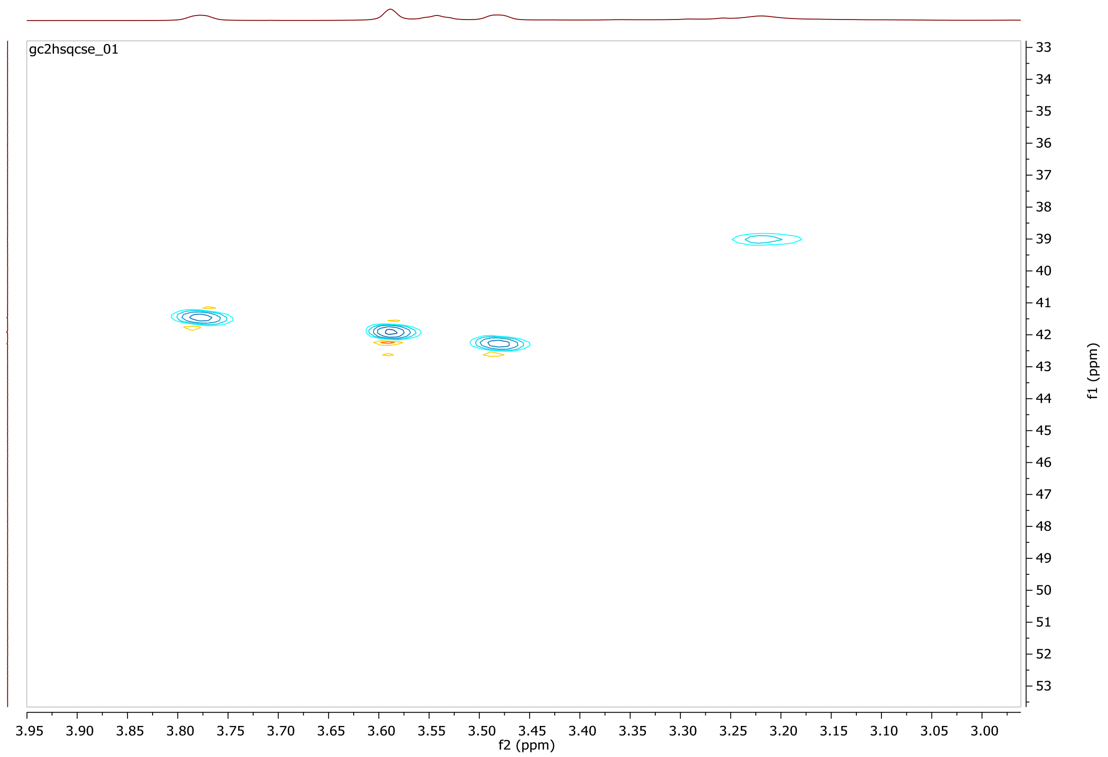

NOE NMR Data

nOe – 6.22 ppm peak

No correlation observed

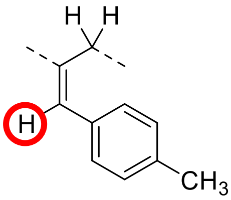

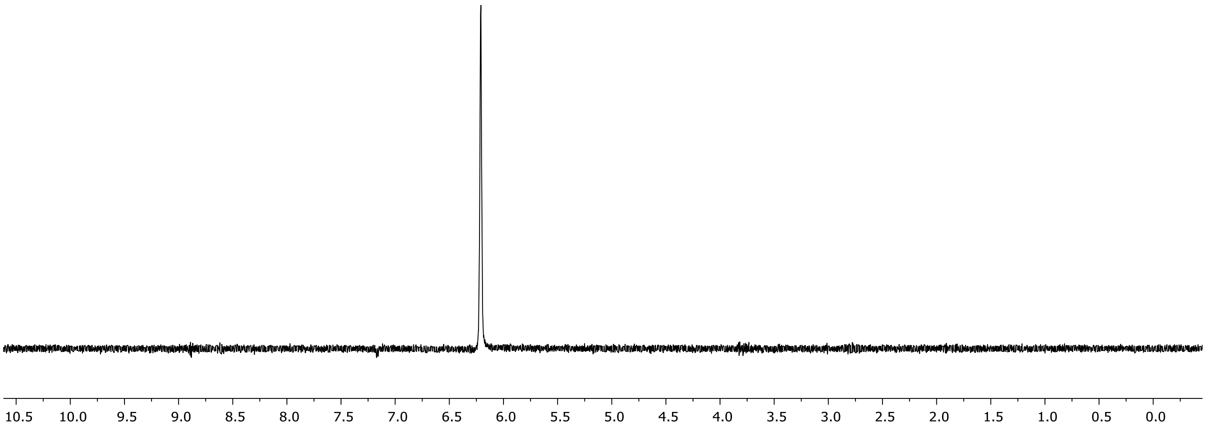

nOe – 3.81 ppm peak

Some correlation with aryl peaks

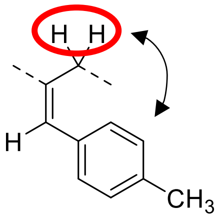

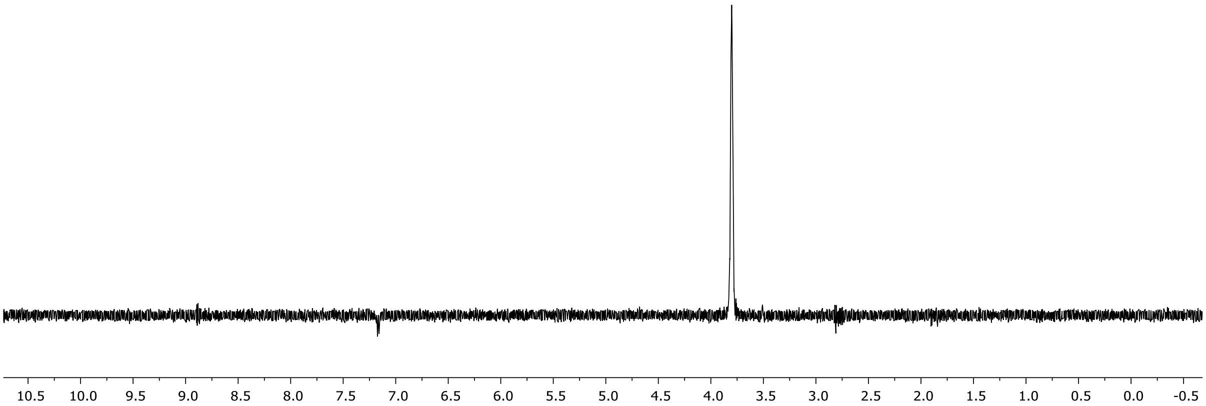

nOe – 3.62 ppm peak

Very weak correlation with aryl peaks

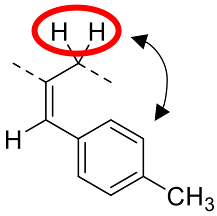

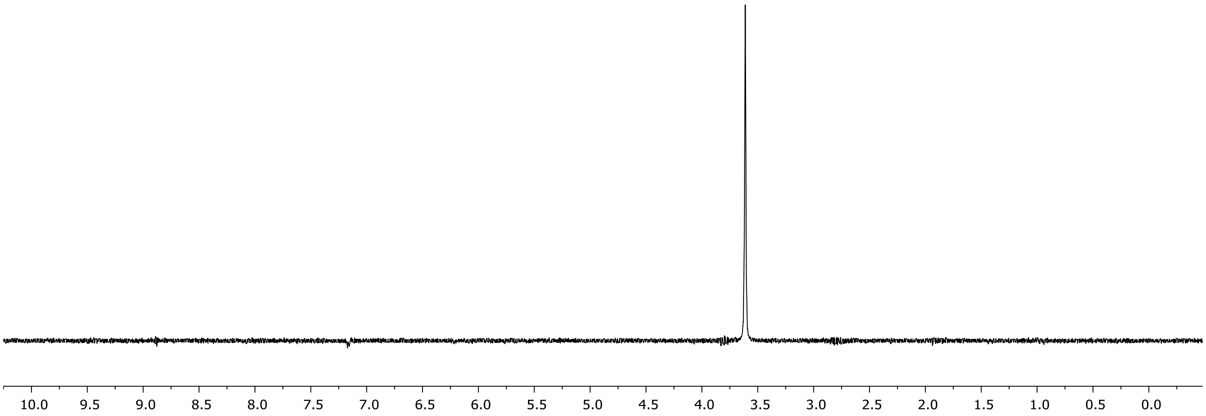

nOe – 3.51 ppm peak

No correlation observed

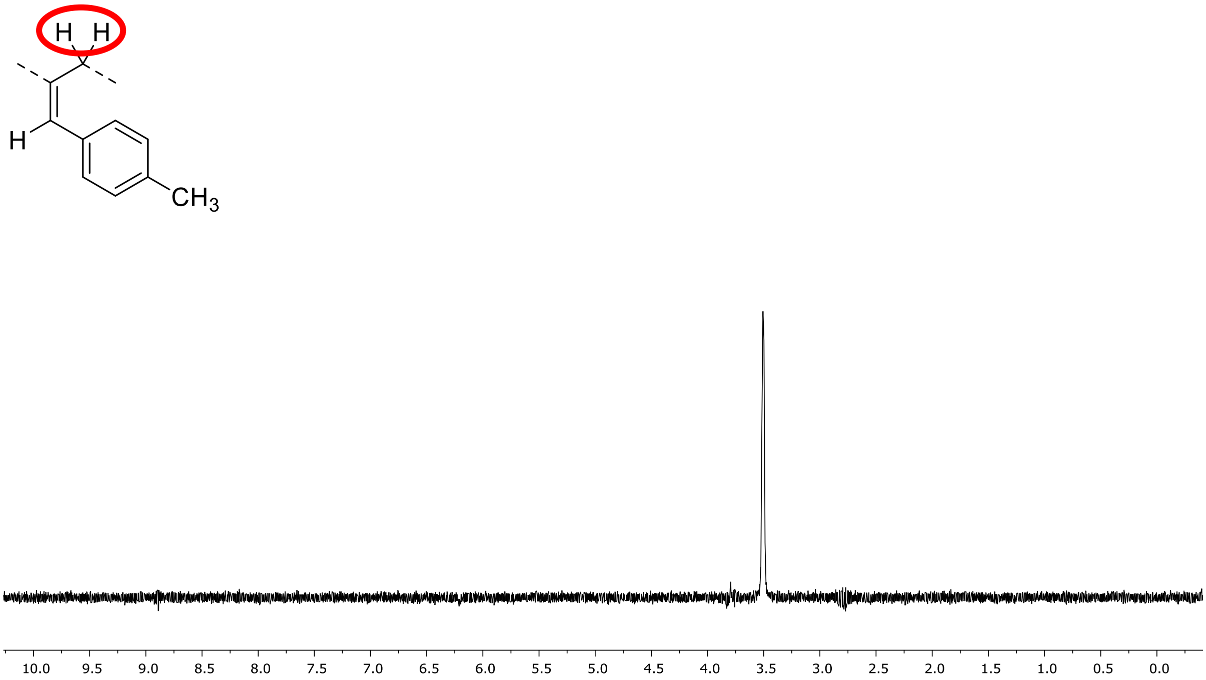

nOe – 2.15 ppm peak

No correlation observed

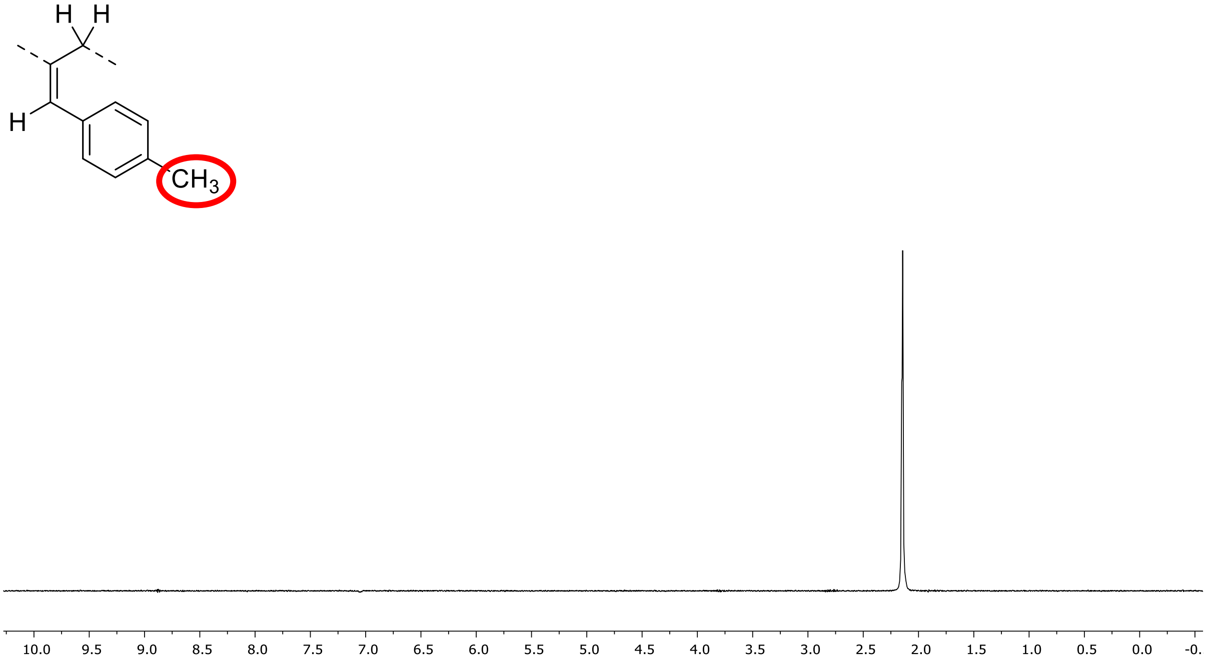

DSC Trace

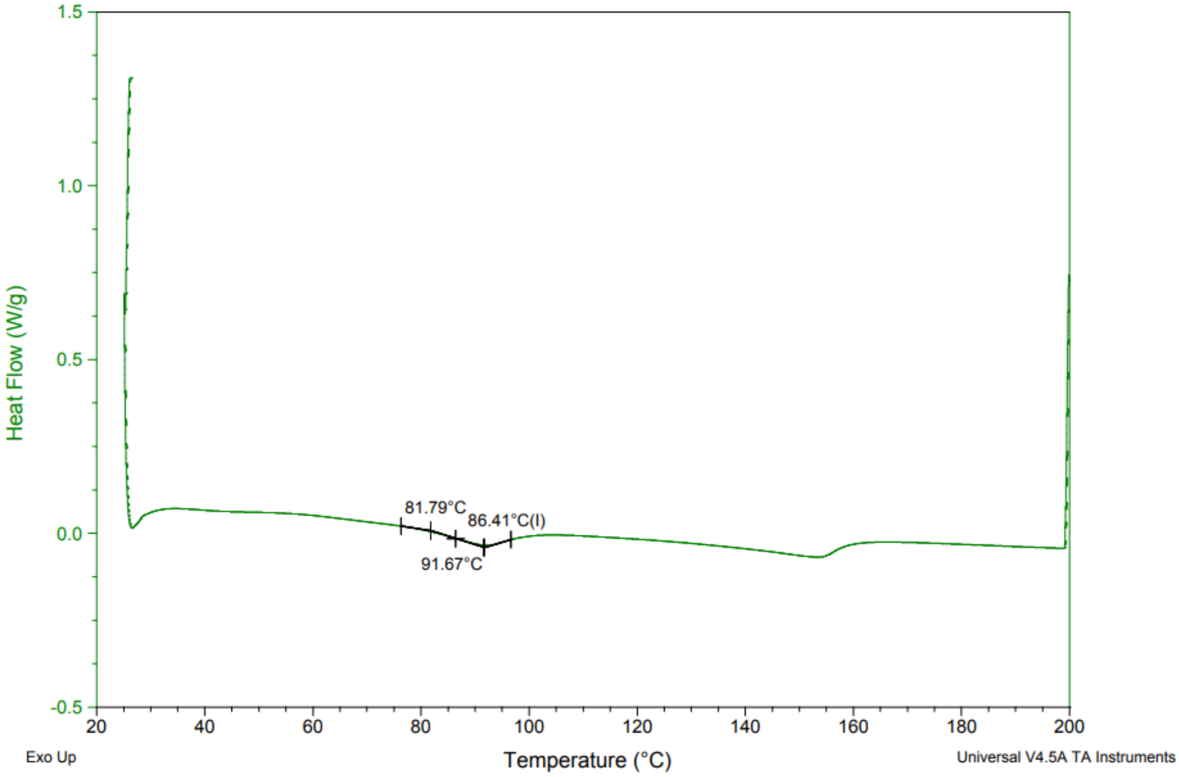

1.5
1.0
0.5
0.0
-0.5
Heat Flow (W/g)
81.79°C
86.41°C(I)
91.67°C
20
40
60
80
100
120
140
160
180
200
Exo Up
Temperature (°C)
Universal V4.5A TA Instruments

Agilent GPC/SEC Software
Sample Triple Analysis Report

Agilent Technologies

CW728A

**Workspace Details**	
Workspace name	Polylactide
Location	C:\ProgramData\Agilent Technologies\GPC\Workspaces\Polylactide\
Comments	
Created by	Administrator at 13:44:31 on 15 June 2015
**Sample Properties**	
Sample name	CW728A
File name	ICF_11_01_2021-0027.sample
Collected by	GPC at 13:22:11 on 14 January 2021
Instrument name	Instrument 1
**System Calibration Used**	
Created by	Administrator at 13:44:30 on 15 June 2015
Last modified by	Administrator at 13:44:30 on 15 June 2015
Comments	
Sample file used for calibration	ICF_6_18_2015-0002.sample
K (RI)	(RI) 750.958
K (LS 90°)	(RI) 30220.130
K (LS 15°)	(RI) 9870.135
K (VS DP)	(RI) 1.107
**System IDD Used**	
Last modified by	Administrator at 15:52:14 on 18 June 2015
Comments	
Reference detector	LS 90°
IDD Light scatterer (secs)	0.0
IDD RI (secs)	-3.0
IDD Viscometer (secs)	-14.0

Analyst: .... **Date:** ....

Checked By: .... **Date:** ....

Agilent GPC/SEC Software A.02.01 [9] Page 1 of 5

Generated by GPC at 14:26 on 14 January 2021

Agilent GPC/SEC Software
Sample Triple Analysis Report

Agilent Technologies

Processing Parameters

Method	Last modified by Administrator at 13:44:30 on 15 June 2015
Concentration Detector Used in Analysis	RI
Injection volume (µL)	100.00
Flow rate (mL/min)	1.00
Concentration options	Calculate Sample Properties from Entered Sample Concentration
Entered sample concentration (mg/mL)	1.000
Calculated dn/dc (mL/g)	0.079
Calculated Ext Coeff ([(mg/mL)-¹] [cm-¹])	0.000
MW calculation method	Use all angles
Log Mi-v-RT curve fit options	Set the fit limits using the limits at peak width of 10 %.
Polynomial curve fit order	1
Use Constant Inlet Pressure	No
Flory-Fox	2.86e+021
DP Multiplier (mV to Pa)	1.0000
IP Multiplier (mV to kPa)	0.1000
Use IV To Calculate Rg	No

MW Ranges Method

Calculate MW Ranges	No

Percentage Fractions Method

Calculate Percentage Fractions	No

Results

Analysed by	GPC at 14:26:06 on 14 January 2021
Comments	

Peak Results

	Detector Type	Peak Max RT (mins)	Bulk MW (g/mol)	Bulk IV (dL/g)	Peak Height (mV)	Peak Height (%)	Peak Area (mV.s)	Area (%)	Conc. (mg/mL)
Peak 1	RI	10.46667	5875176	1.341073	0.316	12.00	10.491	2.94	0.029
Peak 2	RI	11.90000	392256	0.411362	2.314	88.00	346.358	97.06	0.971
Recovery (%)	100.00								

Molecular Weight Averages

Peak	Mp (g/mol)	Mn (g/mol)	Mw (g/mol)	Mz (g/mol)	Mz+1 (g/mol)	Mv (g/mol)	PD
Peak 1	1545686	3331692	5903623	10426880	14325722	9469345	1.772
Peak 2	444372	229455	374781	503892	600640	450179	1.633

Rg Results

Peak	Slope	Intercept	Rgp (nm)	Rgn (nm)	Rgw (nm)	Rgz (nm)	Rgz+1 (nm)
Peak 1	0.3081	-0.3339	37.29	47.35	56.57	67.52	74.53
Peak 2	-0.6562	4.835	13.46	20.76	15.05	12.39	11.04

Analyst: .... **Date:** ....

Checked By: .... **Date:** ....

Agilent GPC/SEC Software
Sample Triple Analysis Report

Agilent Technologies

Rh Results

Peak	Slope	Intercept	Rhp (nm)	Rhn (nm)	Rhw (nm)	Rhz (nm)	Rhz+1 (nm)
Peak 1	0.5214	-1.819	25.50	38.16	51.52	69.44	82.04
Peak 2	0.3268	-0.6928	14.21	11.45	13.44	14.81	15.68

IV Results

Peak	K ((10e-5) dL/g)	Alpha	IVp (dL/g)	IVn (dL/g)	IVw (dL/g)	IVz (dL/g)	IVz+1 (dL/g)
Peak 1	21.95	0.5643	0.681813	1.051645	1.452315	2.001945	2.394960
Peak 2	1.965E+04	0.06031	0.430479	0.413656	0.426080	0.433755	0.438374

Peak Information

	Start (mins)	End (mins)
Baseline region 1	6.41667	8.20000
Baseline region 2	27.13333	32.35000
Peak 1	9.26667	10.46667
Peak 2	10.73333	15.66667

Peak Trace Information

Peak	Trace	Peak Max RT (mins)	Peak Area (mV.s)	Peak Height (mV)
Peak 1	RI	10.46667	10.491	0.316
Peak 1	VS DP	9.41667	231.187	4.103
Peak 1	VS IP	10.11667	14.060	0.627
Peak 1	LS 90°	9.38333	480.183	8.588
Peak 1	LS 15°	9.38333	305.164	8.809
Peak 2	RI	11.90000	346.358	2.314
Peak 2	VS DP	11.63333	2342.121	16.704
Peak 2	VS IP	11.80000	118.915	1.154
Peak 2	LS 90°	11.28333	2141.563	21.841
Peak 2	LS 15°	11.28333	695.978	7.078

Log Mi Curves

Peak	Start (mins)	End (mins)	Polynomial Degree	Coeff a	Coeff b	Coeff c	Coeff d	Coeff e	Coeff f	L Point Y/N
Peak 1	9.2667	10.4667	1	16.495777	-0.98628288	0	0	0	0	N
Peak 2	10.7333	15.1333	1	9.1033846	-0.2903897	0	0	0	0	N

Structural Plot Region

Peak	Start (mins)	End (mins)
Peak 1	9.2667	10.4667
Peak 2	10.7333	15.1333

Analyst: .... **Date:** ....

Checked By: .... **Date:** ....

Agilent GPC/SEC Software
Sample Triple Analysis Report

Agilent Technologies

Chromatogram Plot

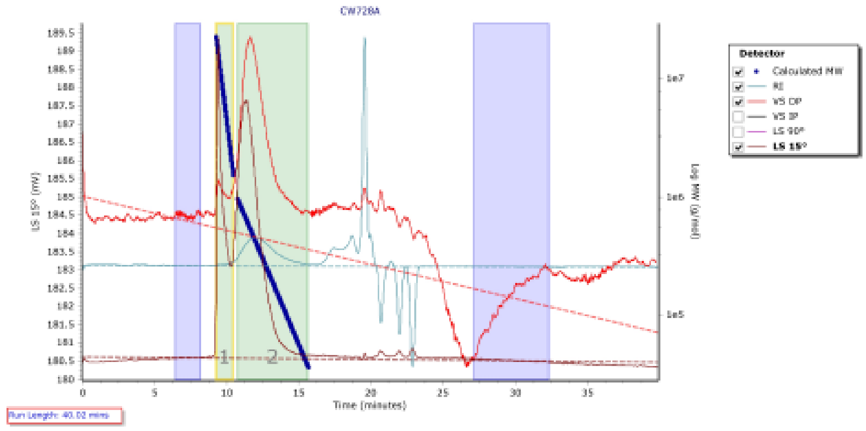

Distribution Plot

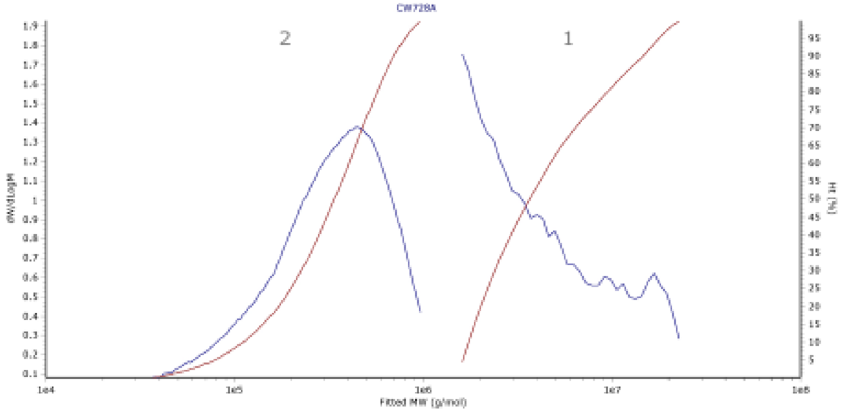

Analyst: .... **Date:** ....

Checked By: .... **Date:** ....

Agilent GPC/SEC Software
Sample Triple Analysis Report

Agilent Technologies

Conformation Plot

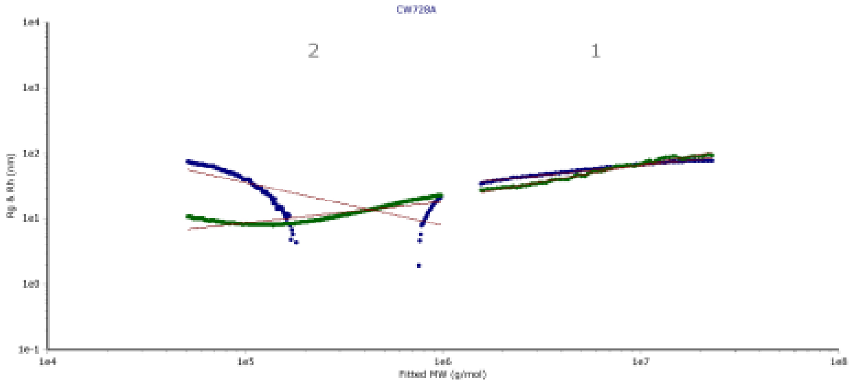

Peak 1 Rg (LS): Log Rg = (0.31) x Log MW + (-0.33), Log Rh = (0.52) x Log MW + (-1.82)
Peak 2 Rg (LS): Log Rg = (-0.66) x Log MW + (4.84), Log Rh = (0.33) x Log MW + (-0.69)

Mark-Houwink Plot

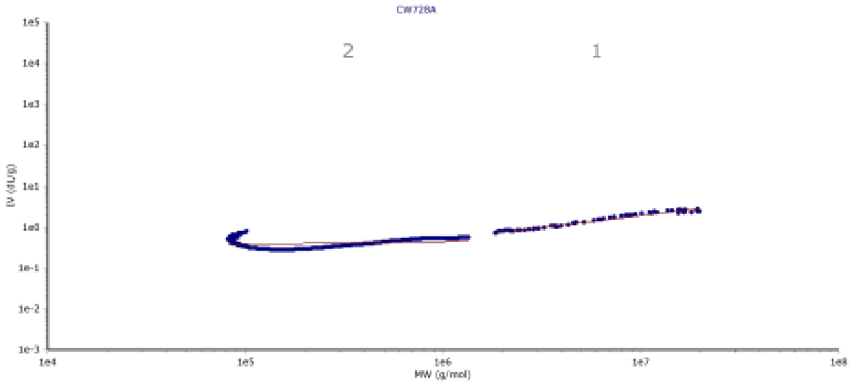

Peak 1: K = ((10e-5) dL/g) 21.95, Alpha = 0.56
Peak 2: K = ((10e-5) dL/g) 19648.56, Alpha = 0.06

Analyst: .... **Date:** ....

Checked By: .... **Date:** ....

Agilent GPC/SEC Software A.02.01 [9]   Page 5 of 5

Generated by GPC at 14:26 on 14 January 2021

Poly(3-methylphenyl) allene (P3)

NMR

The major (2,3) polymer signals are denoted with ●, the minor (1,2) signals are denoted with ●

^{1}H

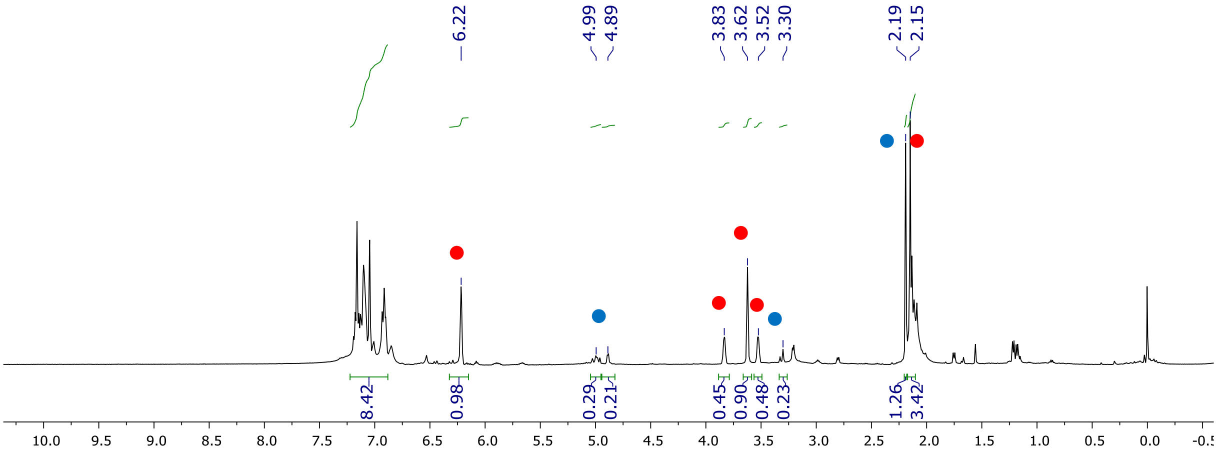

^{13}C{^{1}H}

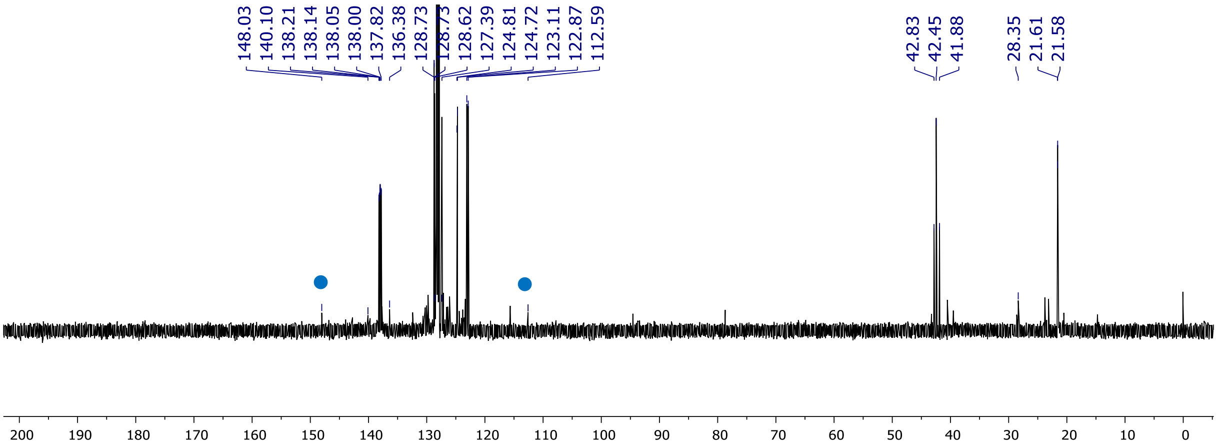

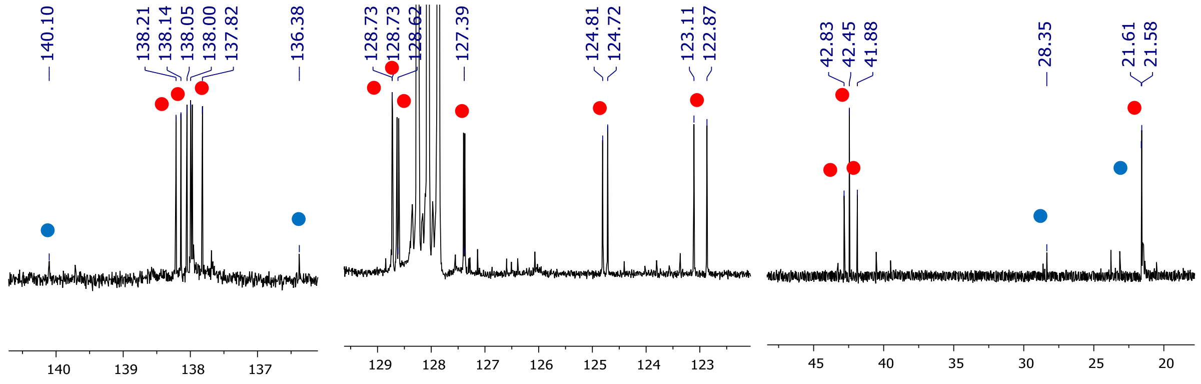

Summary of Correlating Peaks for (2,3) Polymer

Nuclei	^{1}H δ / ppm	^{13}C{^{1}H} δ / ppm
HC_3 / C_3	3.52	42.8
	3.62	42.5
	3.83	41.9

HSQC of correlating region (500 MHz, C_6D_6)

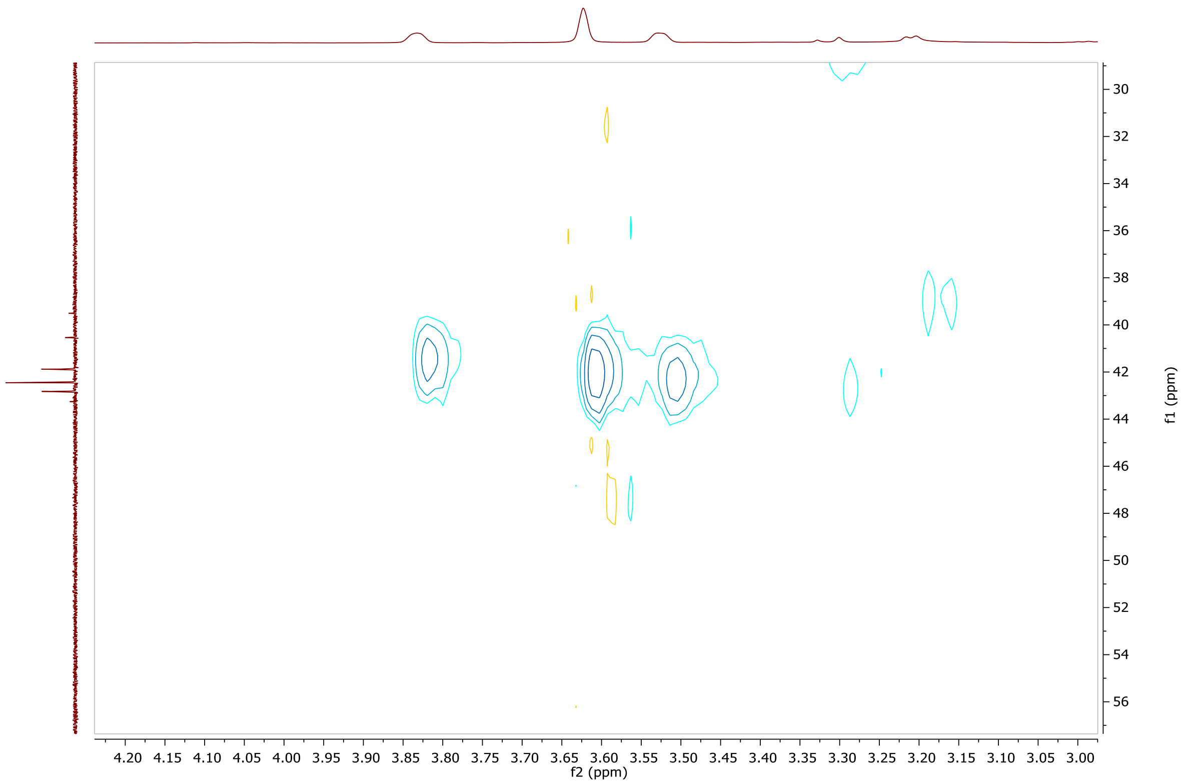

NOE

6.22 (very little correlation)

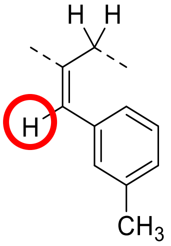

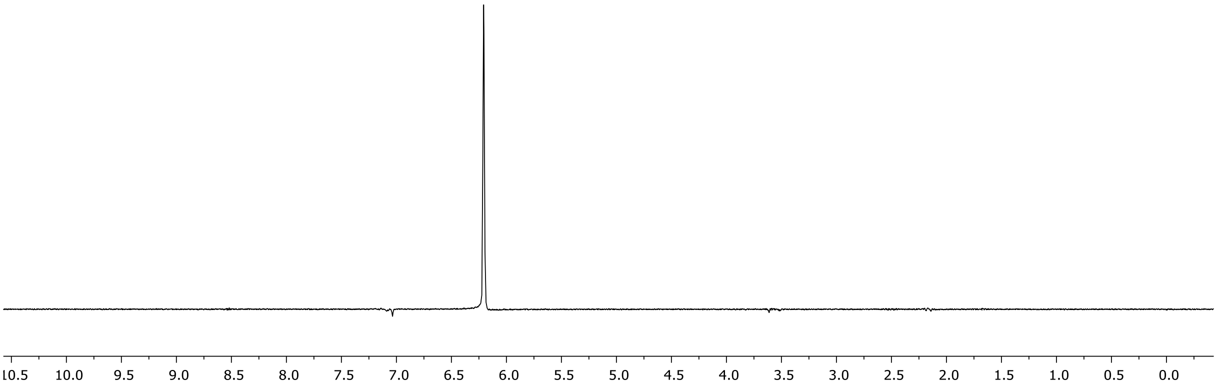

3.83 (some correlation with alkenyl, aryl and methyl protons)

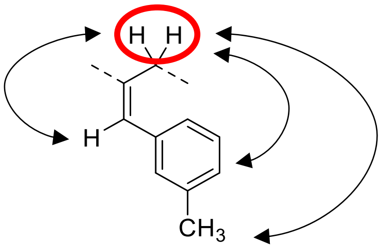

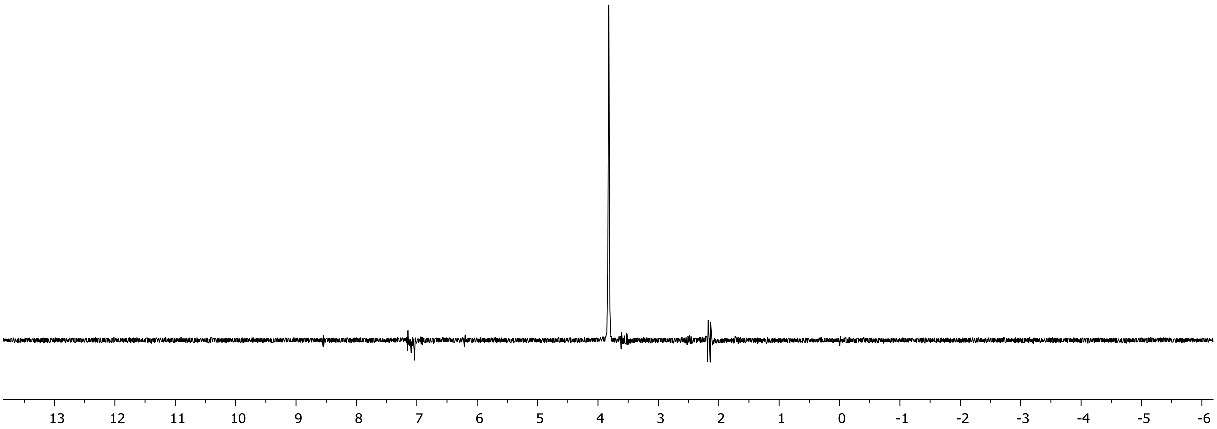

3.62 (very little correlation)

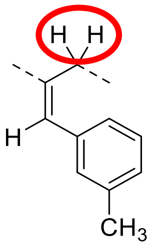

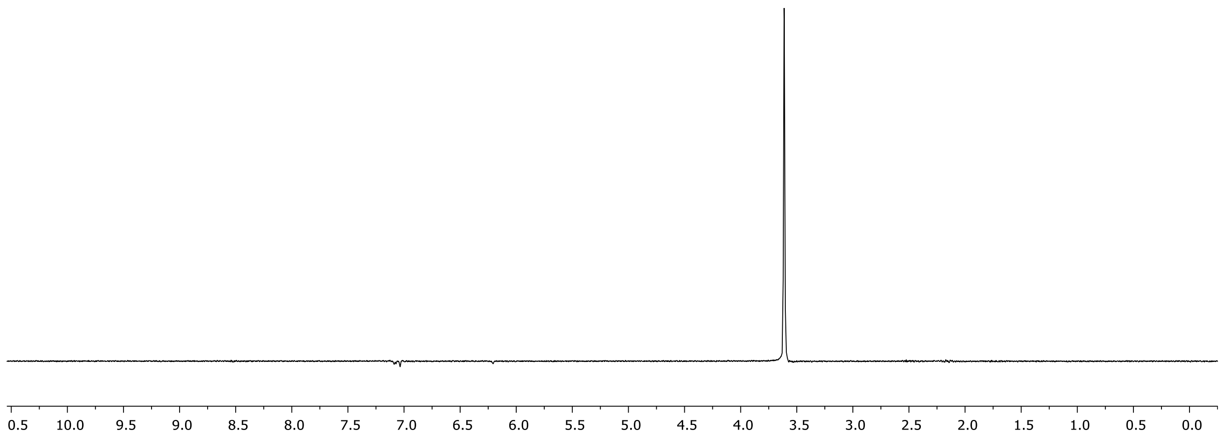

3.52 (some weak correlation aryl, alkenyl and methyl protons)

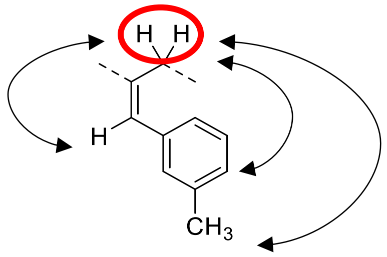

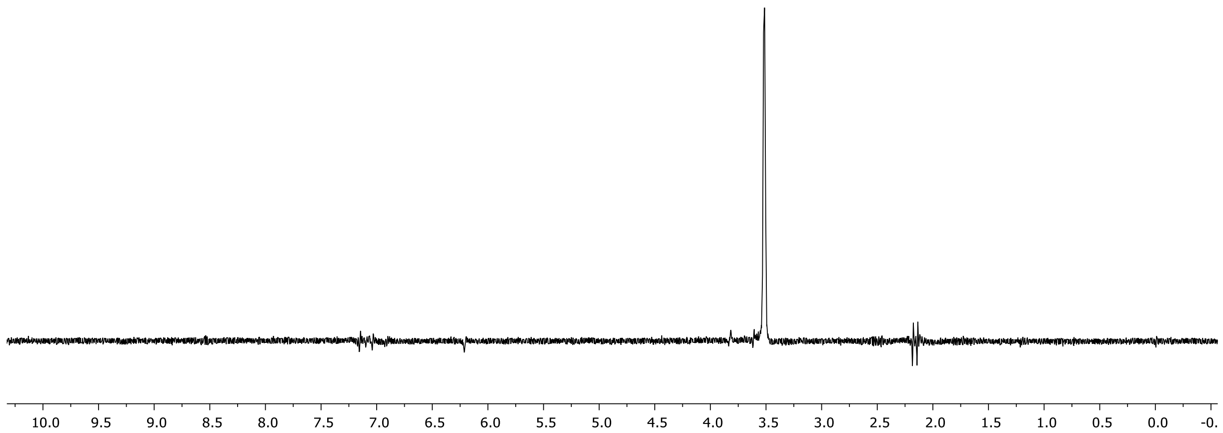

2.15 (weak correlation with main chain and aryl protons)

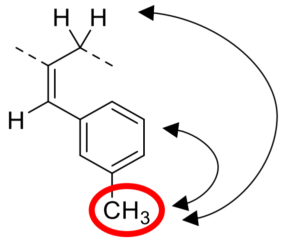

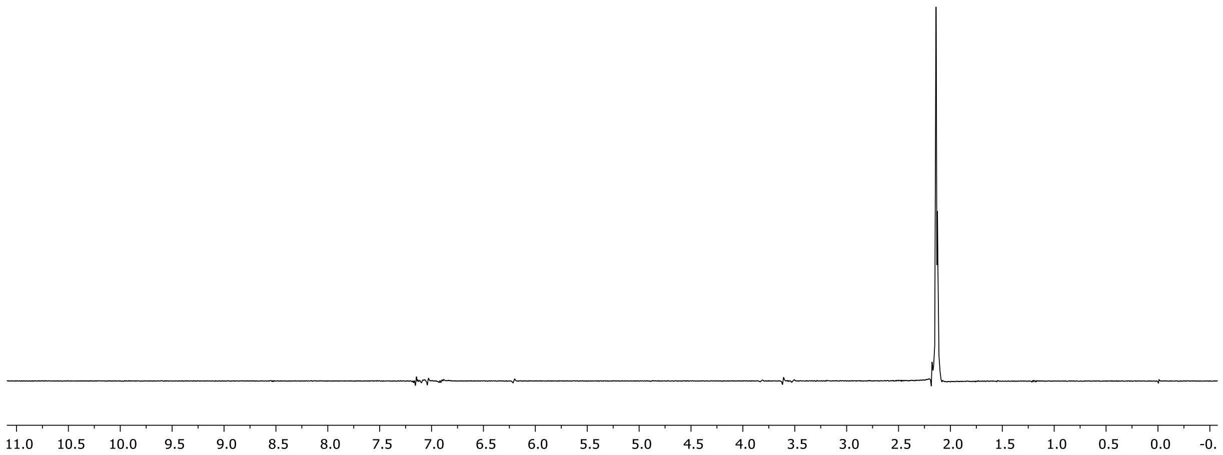

DSC Trace

Uniquely among the polymers studied, **P3** has an additional significant thermal event which we have assigned as a melting transition, with T_m determined as 149.5 °C

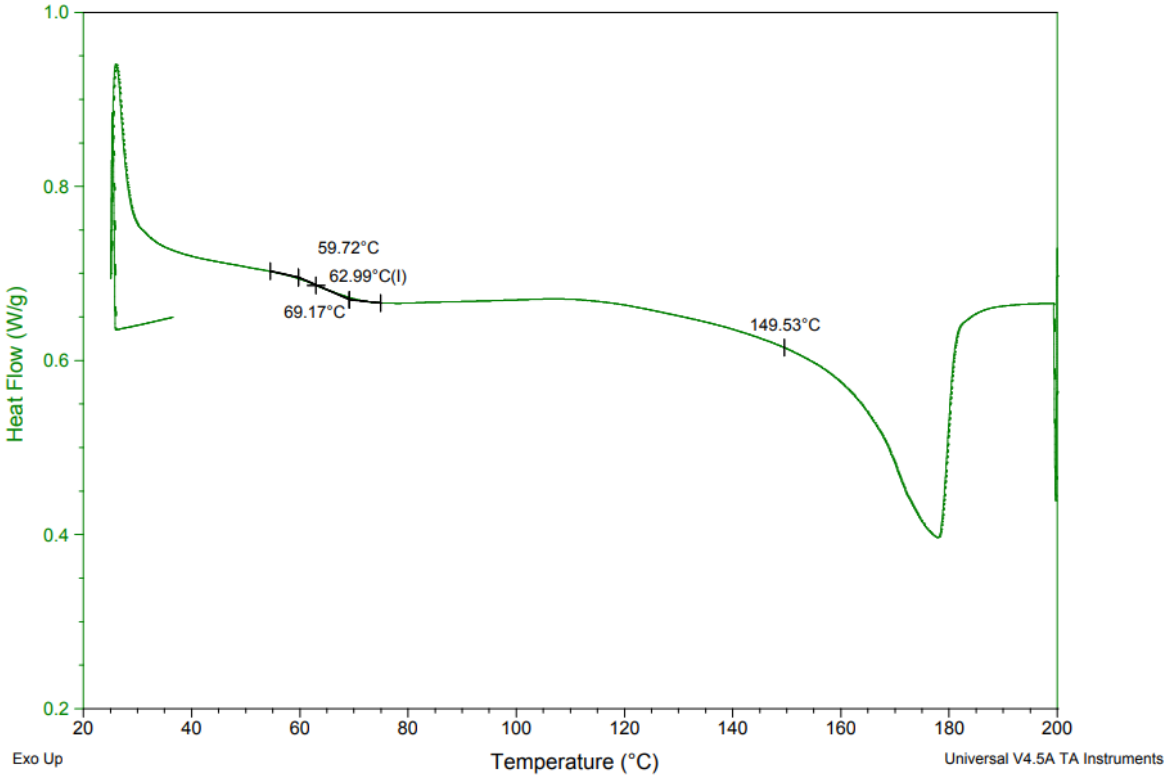

GPC Data Report

Agilent GPC/SEC Software
Sample Triple Analysis Report

Agilent Technologies

cw717_b

**Workspace Details**	
Workspace name	Polylactide
Location	C:\ProgramData\Agilent Technologies\GPC\Workspaces\Polylactide\
Comments	
Created by	Administrator at 13:44:31 on 15 June 2015
**Sample Properties**	
Sample name	cw717_b
File name	ICF_26_11_2020-0012.sample
Collected by	GPC at 17:13:15 on 26 November 2020
Instrument name	Instrument 1
**System Calibration Used**	
Created by	Administrator at 13:44:30 on 15 June 2015
Last modified by	Administrator at 13:44:30 on 15 June 2015
Comments	
Sample file used for calibration	ICF_6_18_2015-0002.sample
K (RI)	(RI) 750.958
K (LS 90°)	(RI) 30220.130
K (LS 15°)	(RI) 9870.135
K (VS DP)	(RI) 1.107
**System IDD Used**	
Last modified by	Administrator at 15:52:14 on 18 June 2015
Comments	
Reference detector	LS 90°
IDD Light scatterer (secs)	0.0
IDD RI (secs)	-3.0
IDD Viscometer (secs)	-14.0

Analyst: .... **Date:** ....

Checked By: .... **Date:** ....

Agilent GPC/SEC Software
Sample Triple Analysis Report

Agilent Technologies

Processing Parameters

Method	Last modified by Administrator at 13:44:30 on 15 June 2015
Concentration Detector Used in Analysis	RI
Injection volume (µL)	100.00
Flow rate (mL/min)	1.00
Concentration options	Calculate Sample Properties from Entered Sample Concentration
Entered sample concentration (mg/mL)	1.000
Calculated dn/dc (mL/g)	0.200
Calculated Ext Coeff ([(mg/mL)-¹] [cm-¹])	0.000
MW calculation method	Use all angles
Log Mi-v-RT curve fit options	Set the fit limits using the limits at peak width of 10 %.
Polynomial curve fit order	1
Use Constant Inlet Pressure	No
Flory-Fox	2.86e+021
DP Multiplier (mV to Pa)	1.0000
IP Multiplier (mV to kPa)	0.1000
Use IV To Calculate Rg	No

MW Ranges Method

Calculate MW Ranges	No

Percentage Fractions Method

Calculate Percentage Fractions	No

Results

Analysed by	GPC at 10:37:46 on 02 December 2020
Comments	

Peak Results

	Detector Type	Peak Max RT (mins)	Bulk MW (g/mol)	Bulk IV (dL/g)	Peak Height (mV)	Peak Height (%)	Peak Area (mV.s)	Area (%)	Conc. (mg/mL)
Peak 1	RI	11.08333	3659876	4.381577	0.160	3.03	2.586	0.29	0.003
Peak 2	RI	12.93333	82617	0.206691	5.125	96.97	896.376	99.71	0.997
Recovery (%)	100.00								

Molecular Weight Averages

Peak	Mp (g/mol)	Mn (g/mol)	Mw (g/mol)	Mz (g/mol)	Mz+1 (g/mol)	Mv (g/mol)	PD
Peak 1	804981	1392615	2165338	4013708	6412088	3635307	1.555
Peak 2	98234	71713	84082	95228	104465	94829	1.172

Rg Results

Peak	Slope	Intercept	Rgp (nm)	Rgn (nm)	Rgw (nm)	Rgz (nm)	Rgz+1 (nm)
Peak 1	0.09718	1.145	52.35	55.21	57.63	61.19	64.05
Peak 2	-1.39	8.27	21.46	33.23	26.64	22.41	19.70

Analyst: .... **Date:** ....

Checked By: .... **Date:** ....

Rh Results

Peak	Slope	Intercept	Rhp (nm)	Rhn (nm)	Rhw (nm)	Rhz (nm)	Rhz+1 (nm)
Peak 1	0.5624	-1.902	26.28	35.77	45.85	64.88	84.43
Peak 2	0.7417	-2.841	7.27	5.76	6.48	7.11	7.61

IV Results

Peak	K ((10e-5) dL/g)	Alpha	IVp (dL/g)	IVn (dL/g)	IVw (dL/g)	IVz (dL/g)	IVz+1 (dL/g)
Peak 1	17.7	0.6625	1.446942	2.080416	2.787039	4.194684	5.721134
Peak 2	0.5765	0.9181	0.220799	0.165398	0.191414	0.214588	0.233625

Peak Information

	Start (mins)	End (mins)
Baseline region 1	2.61667	6.56667
Baseline region 2	32.50000	35.13333
Peak 1	9.46667	11.08333
Peak 2	11.16667	16.90000

Peak Trace Information

Peak	Trace	Peak Max RT (mins)	Peak Area (mV.s)	Peak Height (mV)
Peak 1	RI	11.08333	2.586	0.160
Peak 1	VS DP	11.08333	73.602	1.136
Peak 1	VS IP	10.75000	3.037	-0.509
Peak 1	LS 90°	10.05000	217.429	3.279
Peak 1	LS 15°	9.96667	119.199	1.693
Peak 2	RI	12.93333	896.376	5.125
Peak 2	VS DP	12.55000	1203.629	8.778
Peak 2	VS IP	15.45000	31.252	0.630
Peak 2	LS 90°	12.35000	2553.019	20.879
Peak 2	LS 15°	12.35000	953.008	6.893

Log Mi Curves

Peak	Start (mins)	End (mins)	Polynomial Degree	Coeff a	Coeff b	Coeff c	Coeff d	Coeff e	Coeff f	L Point Y/N
Peak 1	10.4833	11.0833	1	17.333528	-1.0326273	0	0	0	0	N
Peak 2	11.3500	16.8833	1	6.7248996	-0.13396685	0	0	0	0	N

Structural Plot Region

Peak	Start (mins)	End (mins)
Peak 1	10.4833	11.0833
Peak 2	11.3500	16.8833

Analyst: .... **Date:** ....

Checked By: .... **Date:** ....

Agilent GPC/SEC Software
Sample Triple Analysis Report

Agilent Technologies

Chromatogram Plot

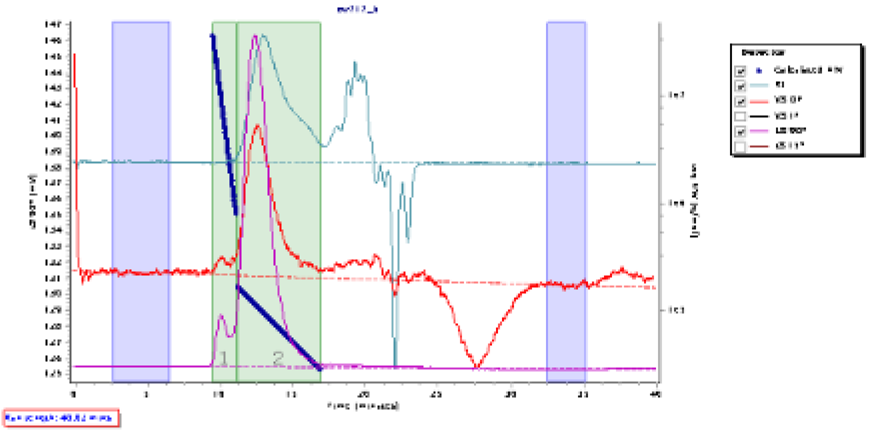

Distribution Plot

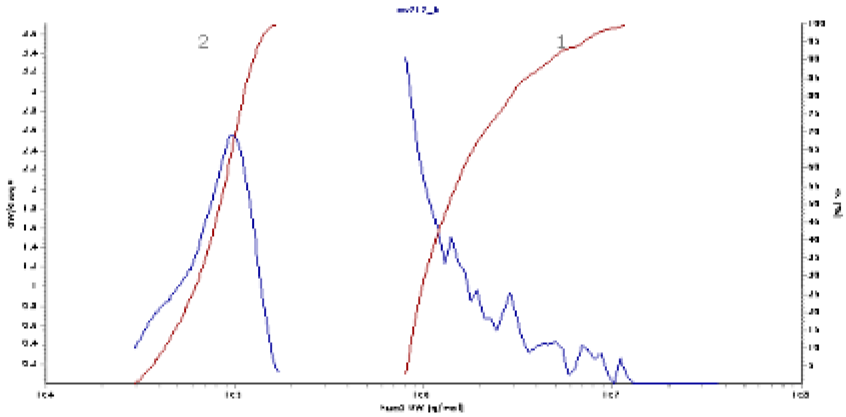

Analyst: .... **Date:** ....

Checked By: .... **Date:** ....

Agilent GPC/SEC Software
Sample Triple Analysis Report

Agilent Technologies

Conformation Plot

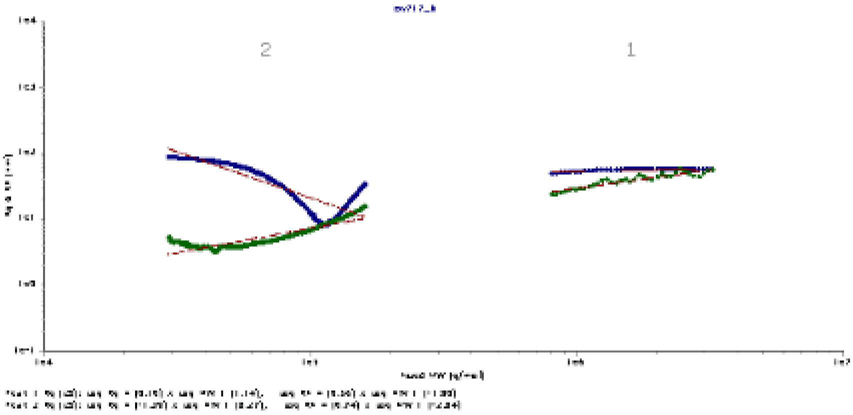

Mark-Houwink Plot

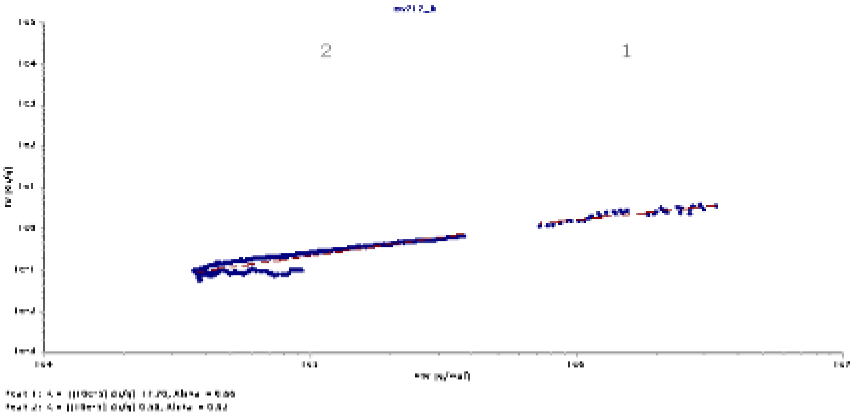

Analyst: .... **Date:** ....

Checked By: .... **Date:** ....

Agilent GPC/SEC Software A.02.01 [9]   Page 5 of 5

Generated by GPC at 10:38 on 02 December 2020

Poly(2-methylphenyl)allene (P4)

NMR

The major (2,3) polymer signals are denoted with ●, the minor (1,2) signals are denoted with ●

^{1}H

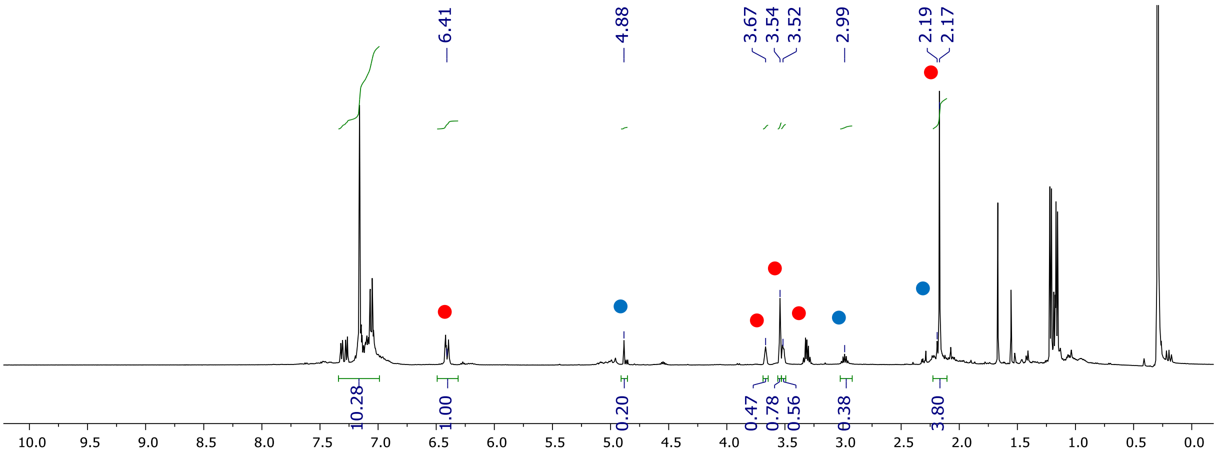

$^{13}C\{^{1}H\}$

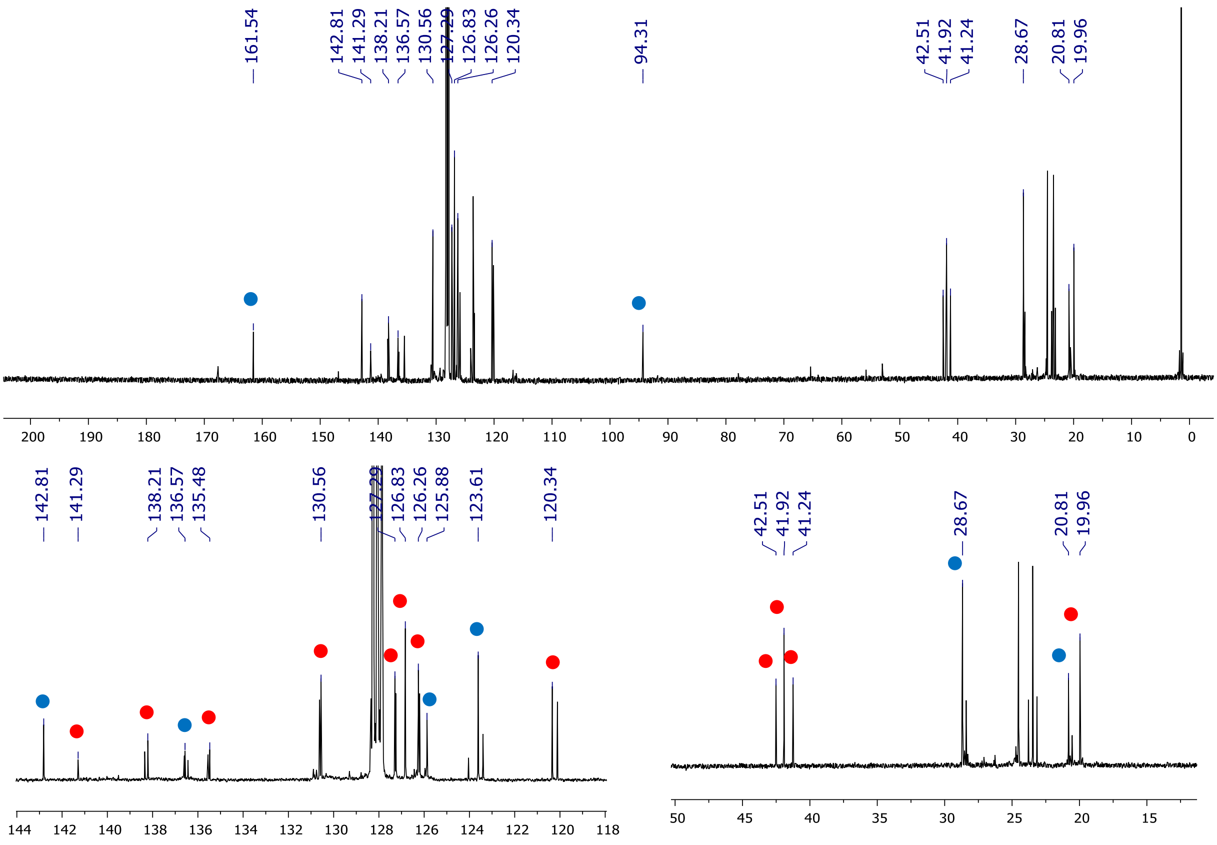

Summary of Correlating Peaks

Nuclei	^{1}H δ / ppm	$^{13}C\{^{1}H\}$ δ / ppm
HC_3 / C_3	3.51	41.2
	3.53	41.9
	3.63	42.5

HSQC of correlating area (500 MHz, C_6D_6)

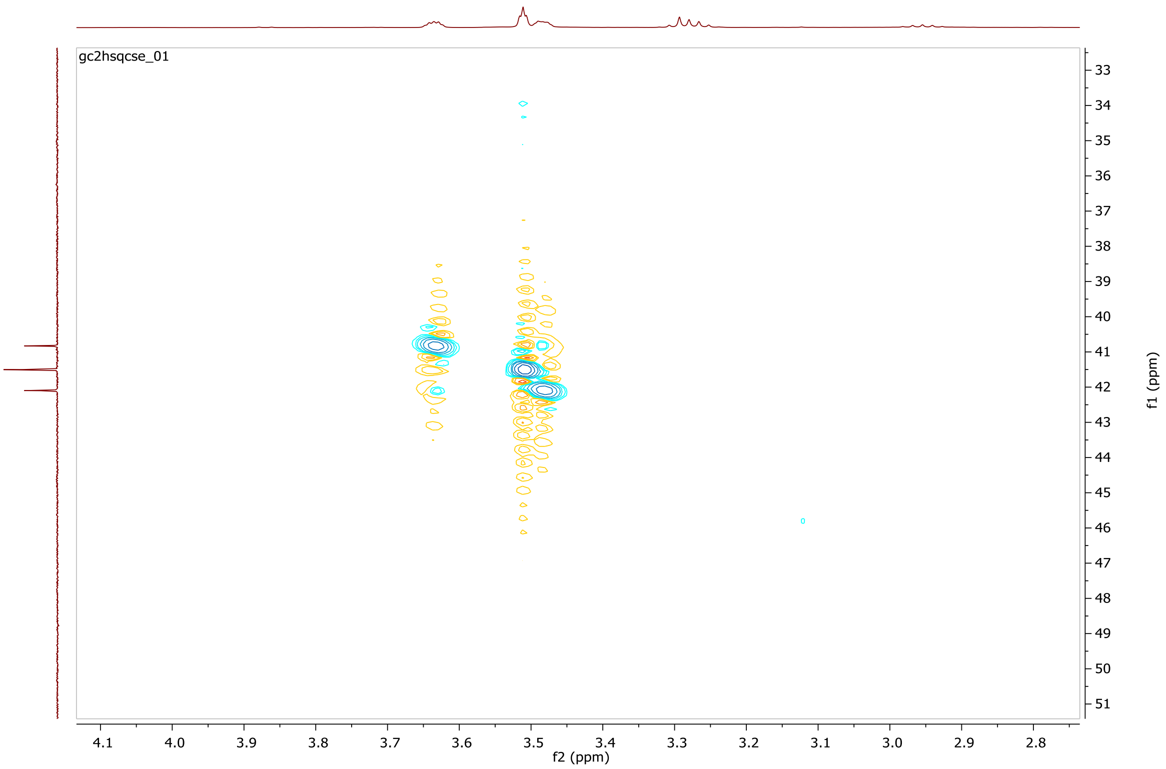

NOE NMR Data

Unlike other polymer structures, **3D** has very little NOE correlation between protons

HC_1 (6.41 ppm) – very little correlation

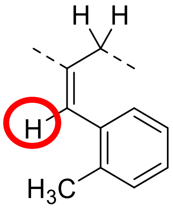

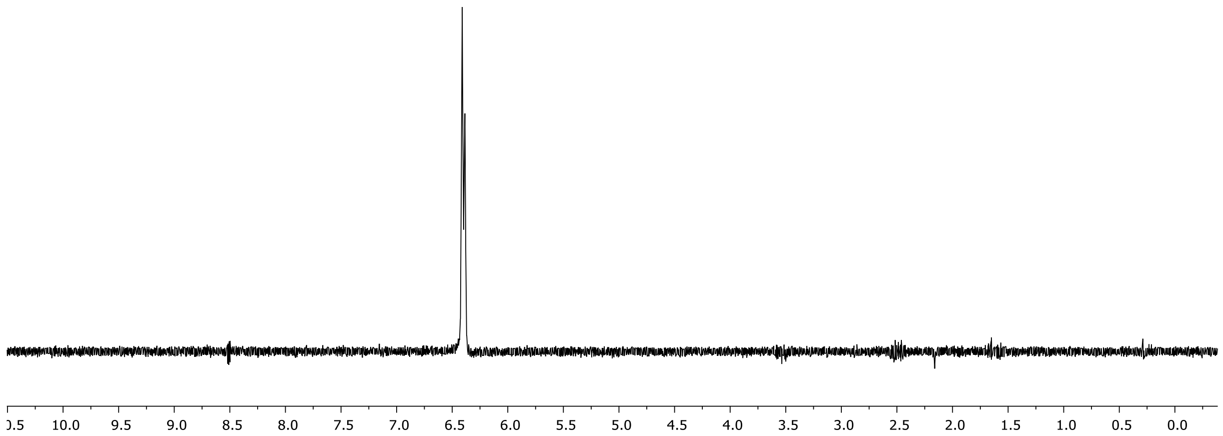

*H*C₃ (3.63 ppm) - very little correlation

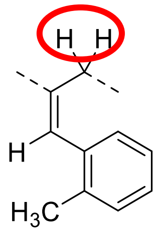

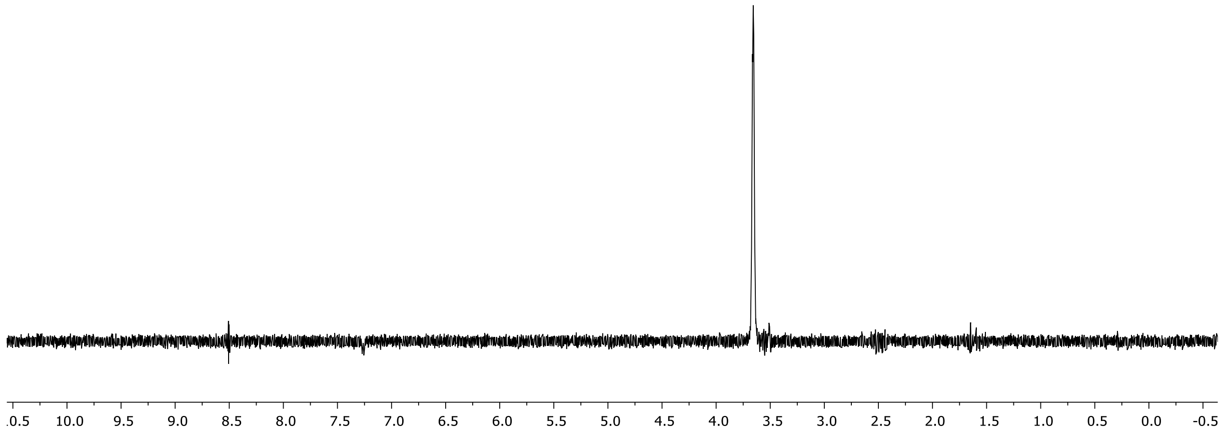

*H*C₃ (3.51/3.53 ppm) - very little correlation

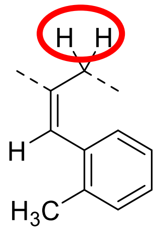

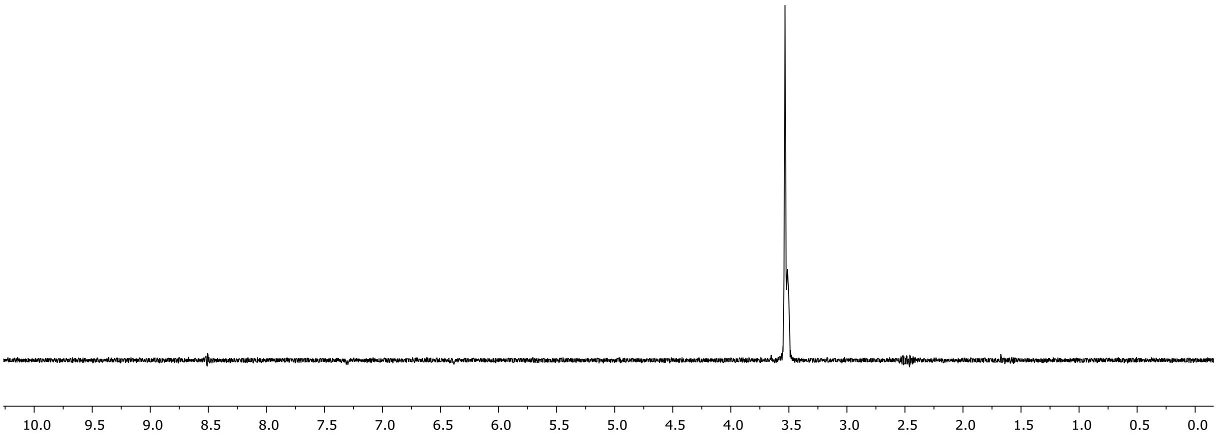

HC_{10} (2.17 ppm) - very little correlation

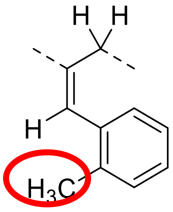

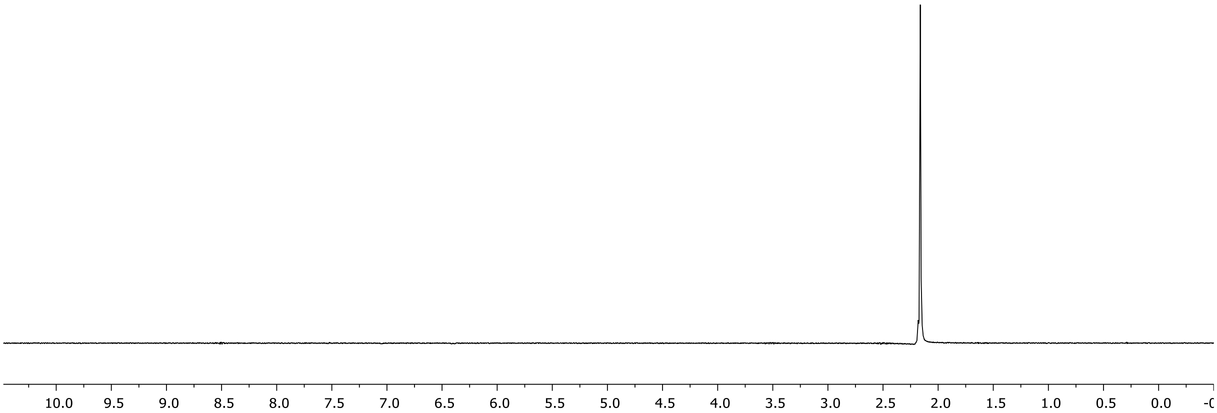

DSC Trace

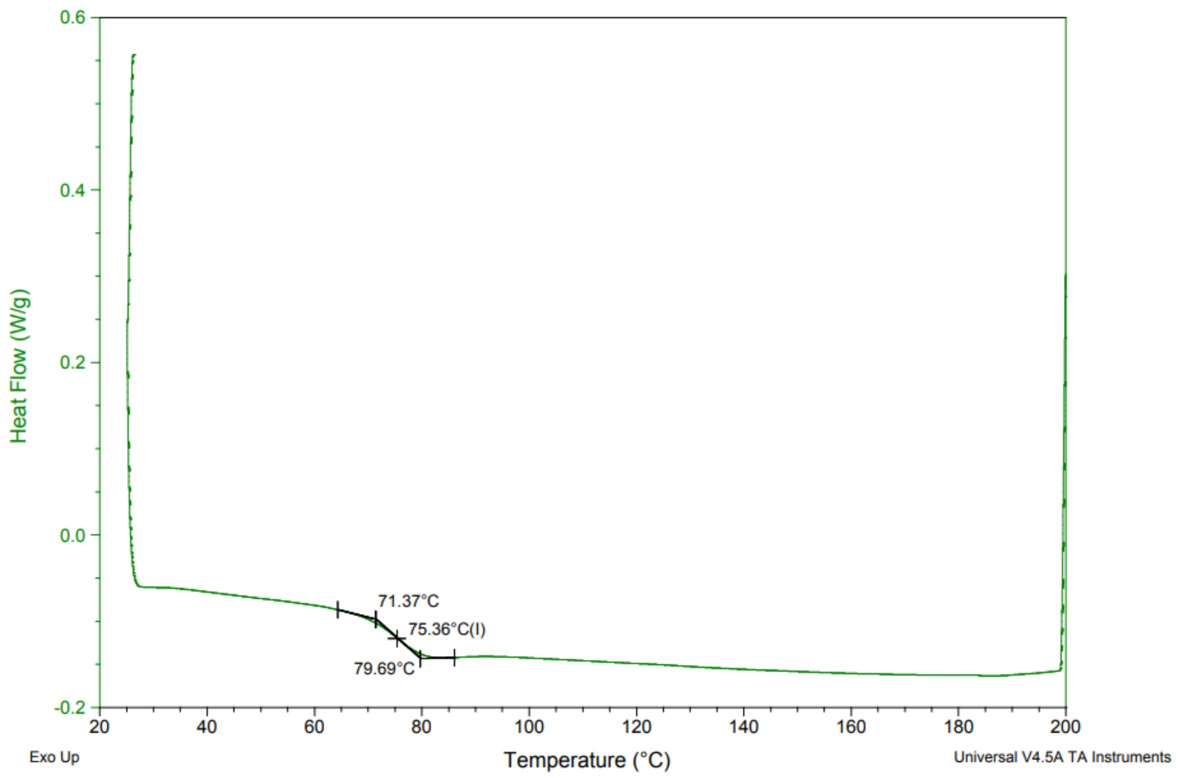

0.6
0.4
0.2
0.0
-0.2
Heat Flow (W/g)
71.37°C
75.36°C(I)
79.69°C
20
40
60
80
100
120
140
160
180
200
Exo Up
Temperature (°C)
Universal V4.5A TA Instruments

GPC Data Report

Agilent GPC/SEC Software
Sample Triple Analysis Report

Agilent Technologies

cw717_a

**Workspace Details**	
Workspace name	Polylactide
Location	C:\ProgramData\Agilent Technologies\GPC\Workspaces\Polylactide\
Comments	
Created by	Administrator at 13:44:31 on 15 June 2015
**Sample Properties**	
Sample name	cw717_a
File name	ICF_26_11_2020-0011.sample
Collected by	GPC at 16:31:22 on 26 November 2020
Instrument name	Instrument 1
**System Calibration Used**	
Created by	Administrator at 13:44:30 on 15 June 2015
Last modified by	Administrator at 13:44:30 on 15 June 2015
Comments	
Sample file used for calibration	ICF_6_18_2015-0002.sample
K (RI)	(RI) 750.958
K (LS 90°)	(RI) 30220.130
K (LS 15°)	(RI) 9870.135
K (VS DP)	(RI) 1.107
**System IDD Used**	
Last modified by	Administrator at 15:52:14 on 18 June 2015
Comments	
Reference detector	LS 90°
IDD Light scatterer (secs)	0.0
IDD RI (secs)	-3.0
IDD Viscometer (secs)	-14.0

Analyst: .... **Date:** ....

Checked By: .... **Date:** ....

Agilent GPC/SEC Software
Sample Triple Analysis Report

Agilent Technologies

Processing Parameters

Method	Last modified by Administrator at 13:44:30 on 15 June 2015
Concentration Detector Used in Analysis	RI
Injection volume (µL)	100.00
Flow rate (mL/min)	1.00
Concentration options	Calculate Sample Properties from Entered Sample Concentration
Entered sample concentration (mg/mL)	1.000
Calculated dn/dc (mL/g)	0.022
Calculated Ext Coeff ([(mg/mL)-¹][cm-¹])	0.000
MW calculation method	Use all angles
Log Mi-v-RT curve fit options	Set the fit limits using the limits at peak width of 10 %.
Polynomial curve fit order	1
Use Constant Inlet Pressure	No
Flory-Fox	2.86e+021
DP Multiplier (mV to Pa)	1.0000
IP Multiplier (mV to kPa)	0.1000
Use IV To Calculate Rg	No

MW Ranges Method

Calculate MW Ranges	No

Percentage Fractions Method

Calculate Percentage Fractions	No

Results

Analysed by	GPC at 10:35:18 on 02 December 2020
Comments	

Peak Results

	Detector Type	Peak Max RT (mins)	Bulk MW (g/mol)	Bulk IV (dL/g)	Peak Height (mV)	Peak Height (%)	Peak Area (mV.s)	Area (%)	Conc. (mg/mL)
Peak 1	RI	10.96667	24460195	0.506893	-0.043	-7.47	2.333	2.33	0.023
Peak 2	RI	14.33333	309498	0.046793	0.612	107.47	97.770	97.67	0.977
Recovery (%)	100.00								

Molecular Weight Averages

Peak	Mp (g/mol)	Mn (g/mol)	Mw (g/mol)	Mz (g/mol)	Mz+1 (g/mol)	Mv (g/mol)	PD
Peak 1	4063780	7212410	72983470	189547619	235342792	160170141	10.12
Peak 2	252147	184974	300758	473732	665819	445633	1.626

Rg Results

Peak	Slope	Intercept	Rgp (nm)	Rgn (nm)	Rgw (nm)	Rgz (nm)	Rgz+1 (nm)
Peak 1	0.09285	0.8487	29.30	30.56	36.21	38.84	39.46
Peak 2	-0.5208	3.918	12.73	14.93	11.63	9.20	7.73

Analyst: .... **Date:** ....

Checked By: .... **Date:** ....

Agilent GPC/SEC Software
Sample Triple Analysis Report

Agilent Technologies

Rh Results

Peak	Slope	Intercept	Rhp (nm)	Rhn (nm)	Rhw (nm)	Rhz (nm)	Rhz+1 (nm)
Peak 1	0.2321	-0.03264	28.60	36.49	97.52	146.27	160.36
Peak 2	0.5404	-2.184	5.43	4.59	5.98	7.65	9.20

IV Results

Peak	K ((10e-5) dL/g)	Alpha	IVp (dL/g)	IVn (dL/g)	IVw (dL/g)	IVz (dL/g)	IVz+1 (dL/g)
Peak 1	7032	0.1161	0.411423	0.439756	0.575299	0.642702	0.659052
Peak 2	0.7599	0.6891	0.040087	0.032381	0.045265	0.061906	0.078270

Peak Information

	Start (mins)	End (mins)
Baseline region 1	4.58333	5.78333
Baseline region 2	30.86667	32.48333
Peak 1	8.75000	11.48333
Peak 2	11.65000	16.21667

Peak Trace Information

Peak	Trace	Peak Max RT (mins)	Peak Area (mV.s)	Peak Height (mV)
Peak 1	RI	10.96667	2.333	-0.043
Peak 1	VS DP	11.45000	69.007	1.260
Peak 1	VS IP	11.28333	7.916	-0.498
Peak 1	LS 90°	9.80000	178.259	2.972
Peak 1	LS 15°	9.75000	80.877	1.393
Peak 2	RI	14.33333	97.770	0.612
Peak 2	VS DP	12.55000	266.755	1.448
Peak 2	VS IP	15.10000	56.962	-0.644
Peak 2	LS 90°	13.31667	134.624	0.894
Peak 2	LS 15°	13.31667	43.492	0.288

Log Mi Curves

Peak	Start (mins)	End (mins)	Polynomial Degree	Coeff a	Coeff b	Coeff c	Coeff d	Coeff e	Coeff f	L Point Y/N
Peak 1	10.4833	11.1667	1	16.343505	-0.88765117	0	0	0	0	N
Peak 2	12.3500	16.2000	1	10.045305	-0.32397566	0	0	0	0	N

Structural Plot Region

Peak	Start (mins)	End (mins)
Peak 1	10.4833	11.1667
Peak 2	12.3500	16.2000

Analyst: .... **Date:** ....

Checked By: .... **Date:** ....

Agilent GPC/SEC Software
Sample Triple Analysis Report

Agilent Technologies

Chromatogram Plot

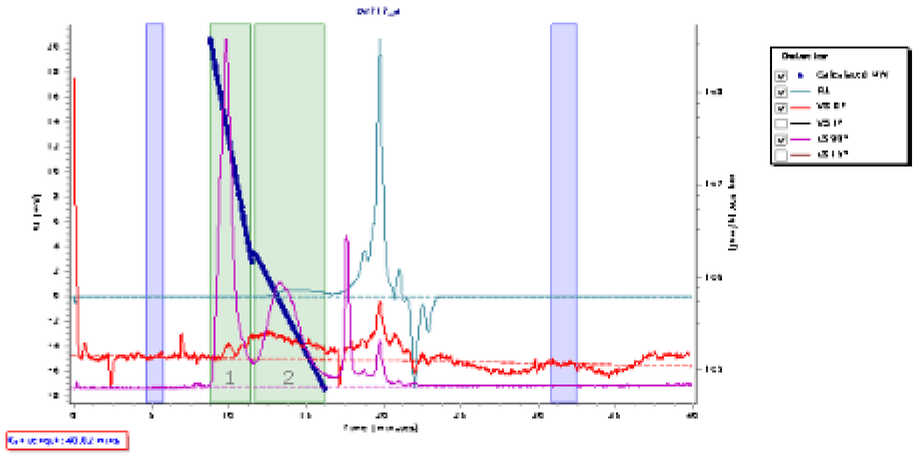

Distribution Plot

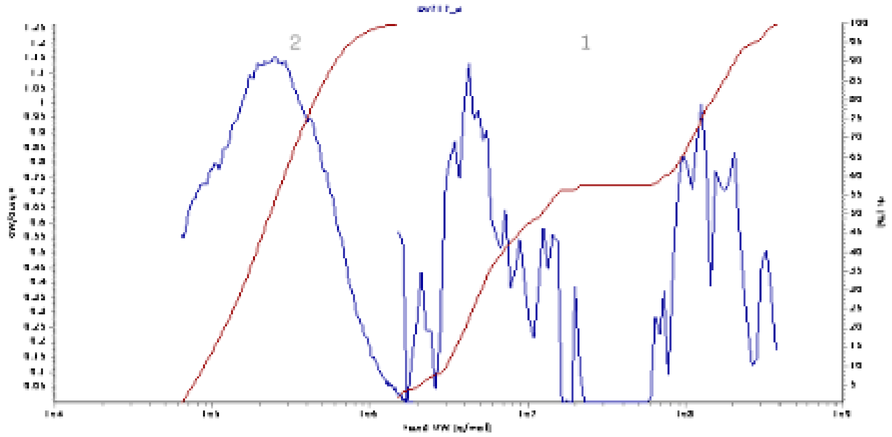

Analyst: .... **Date:** ....

Checked By: .... **Date:** ....

Agilent GPC/SEC Software A.02.01 [9] Page 4 of 5

Generated by GPC at 10:35 on 02 December 2020

Conformation Plot

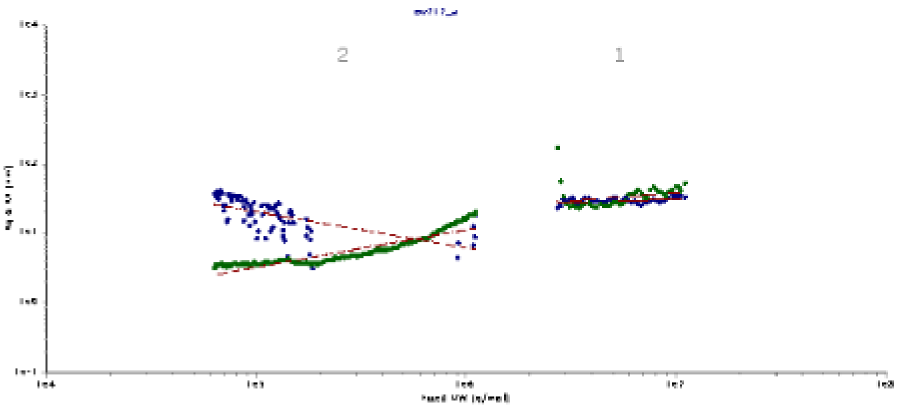

[illegible]
[illegible]

Mark-Houwink Plot

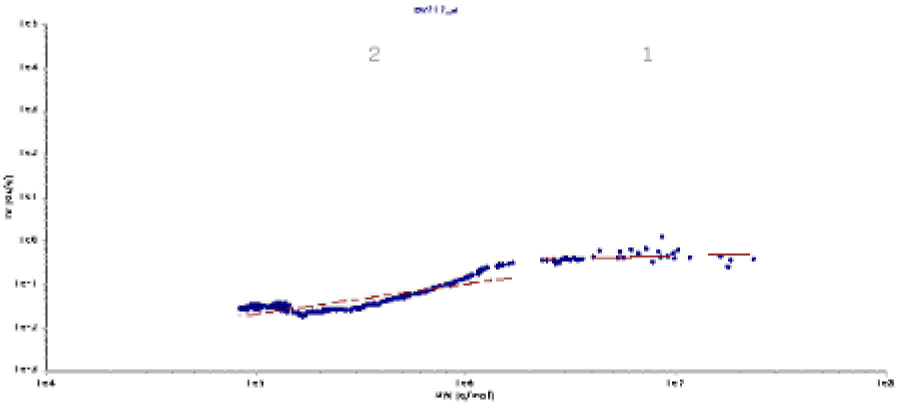

[illegible]
[illegible]

Analyst: .... **Date:** ....

Checked By: .... **Date:** ....

Poly(4-methoxyphenyl)allene (P5)

NMR

The major (2,3) polymer signals are denoted with ●, the minor (1,2) signals are denoted with ●

^{1}H

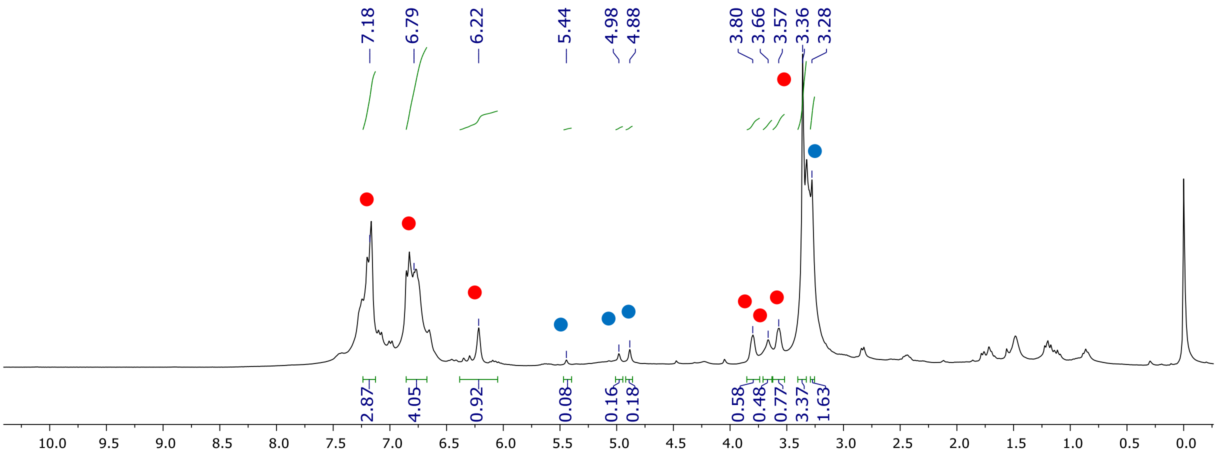

^{13}C{^{1}H}

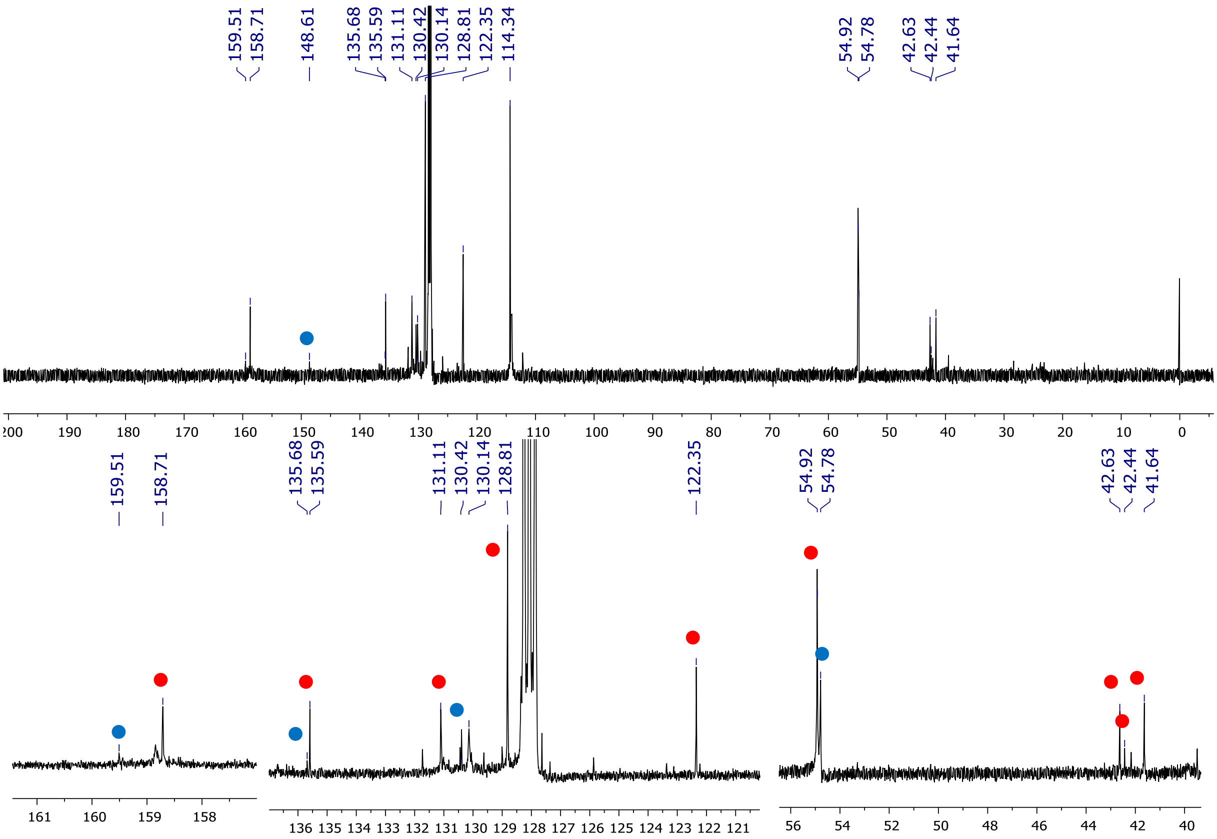

Summary of H/C correlating peaks for (2,3) isomer

Nuclei	^{1}H δ / ppm	$^{13}C\{^{1}H\}$ δ / ppm
HC_3 / C_3	3.80	41.6
	3.66	42.4
	3.57	42.6

HSQC of correlating area

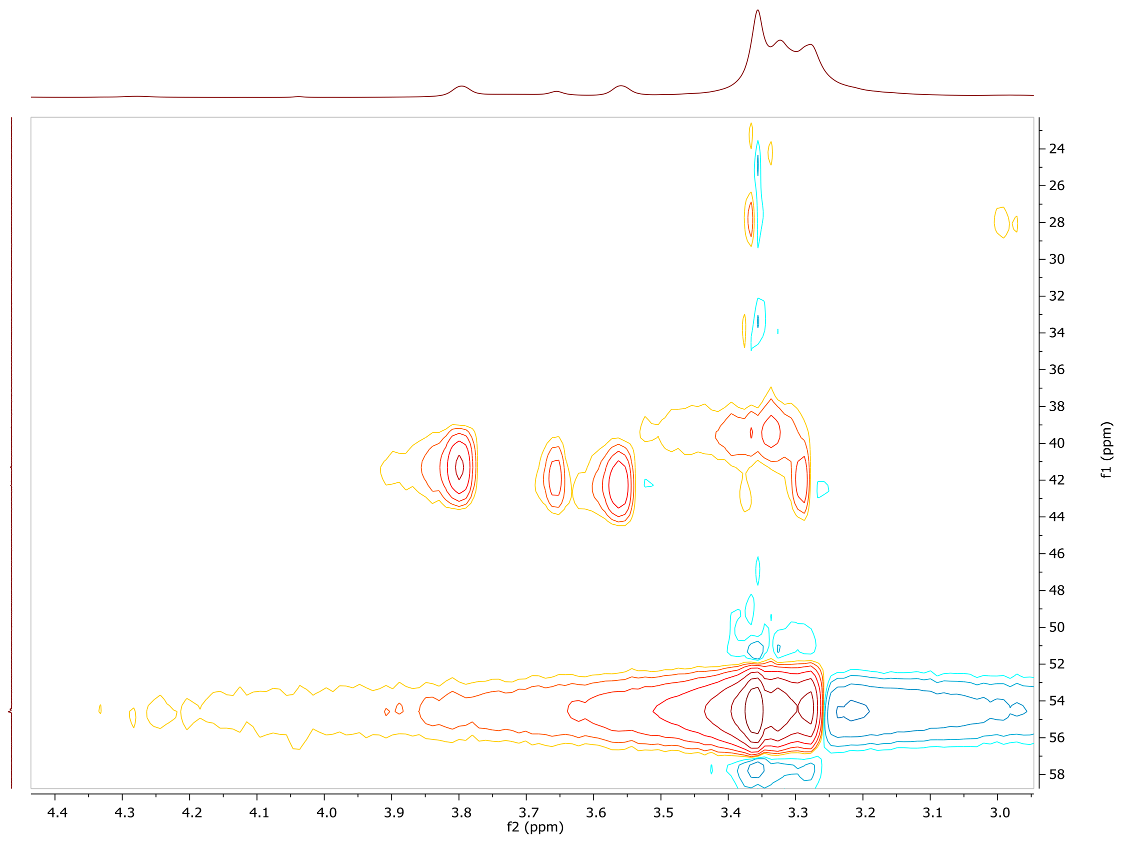

NOE

nOe – 6.22 ppm peak

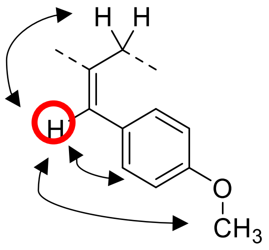

Some correlation with chain and aryl peaks, strong correlation with methoxy peak

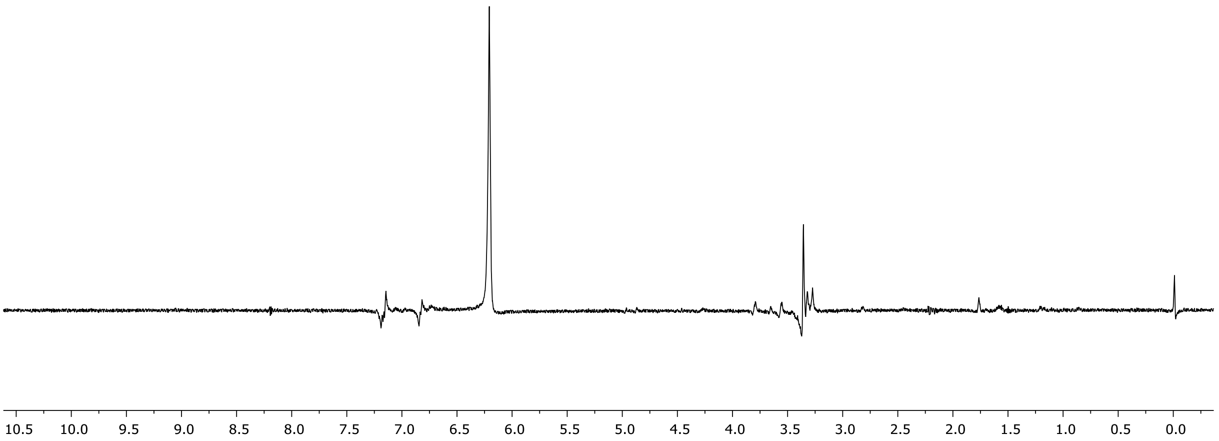

Some correlation with peaks at 3.80 ppm / 3.57 ppm with alkenyl/aryl protons, very little with 3.66 ppm

nOe - 3.80 ppm peak

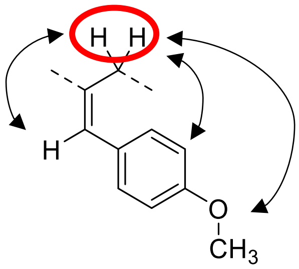

Some correlation with aryl, alkenyl and methoxy peaks

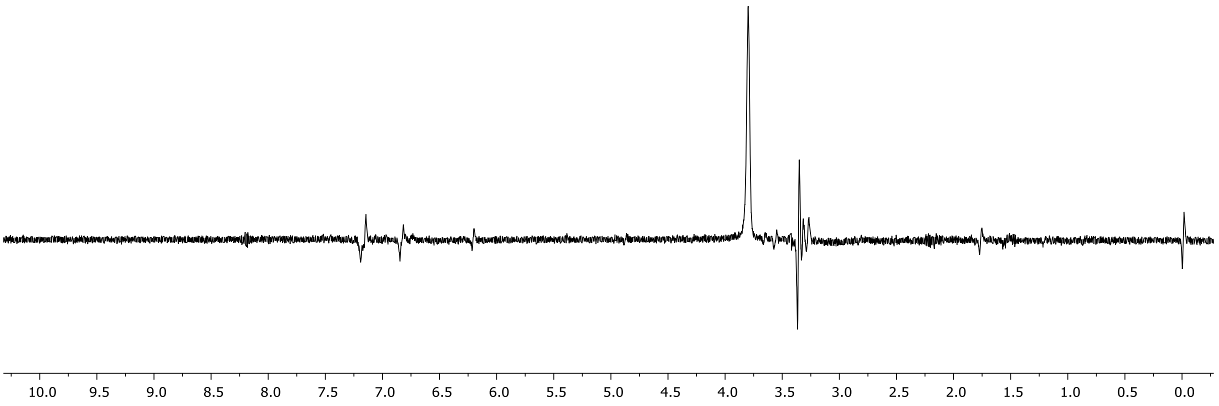

nOe - 3.66 ppm peak

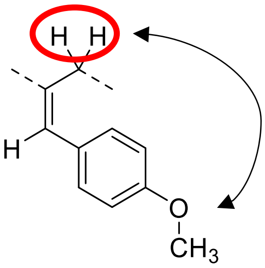

Some correlation with methoxy peak only

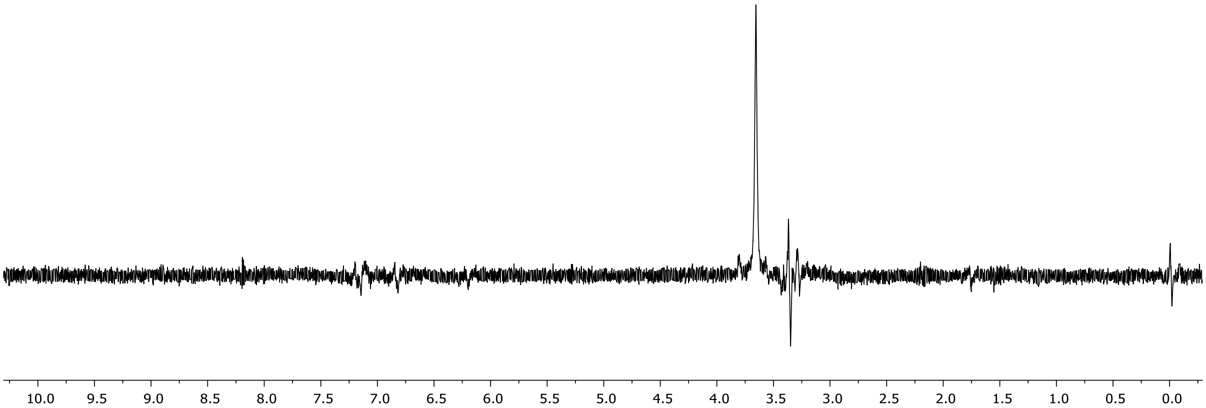

nOe - 3.57 ppm peak

Weak correlation with aryl and methoxy peaks

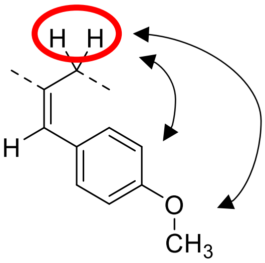

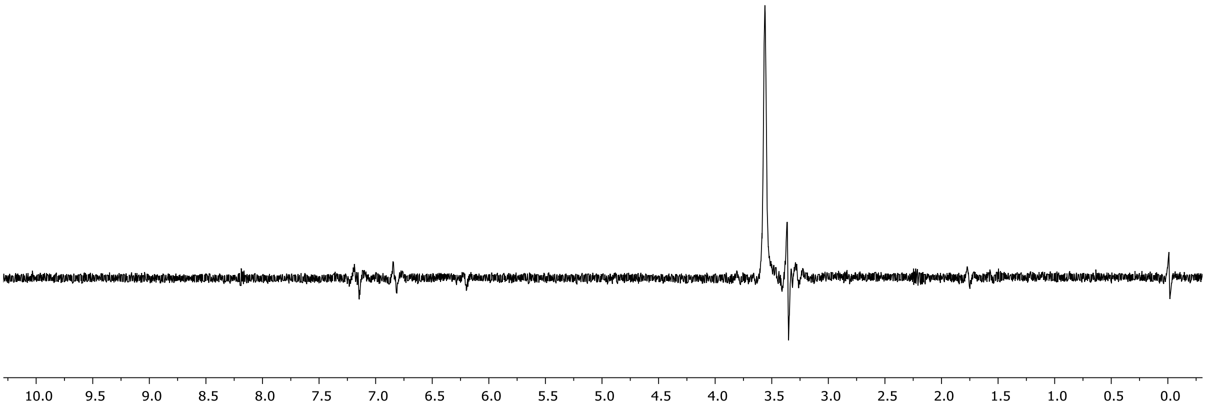

3.36 ppm (Methoxy signal, no strong correlation)

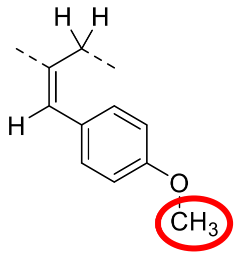

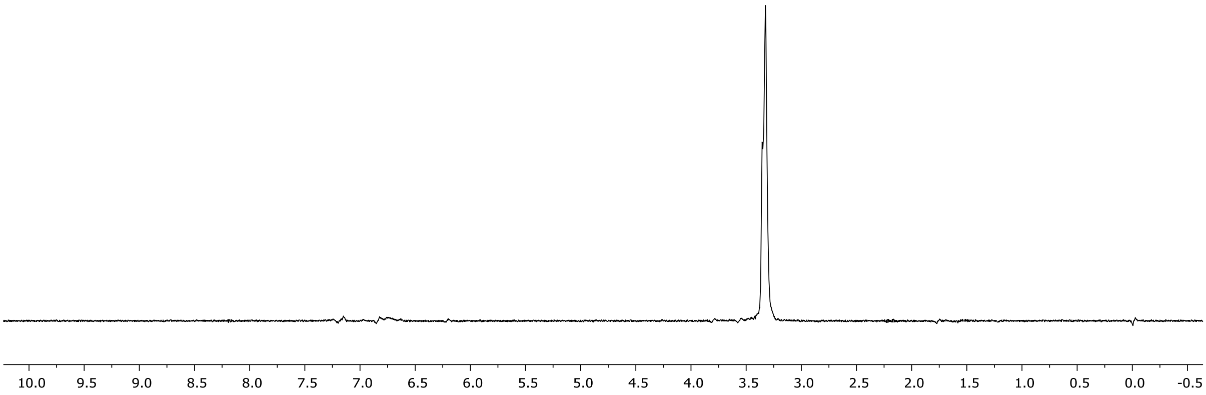

DSC Trace

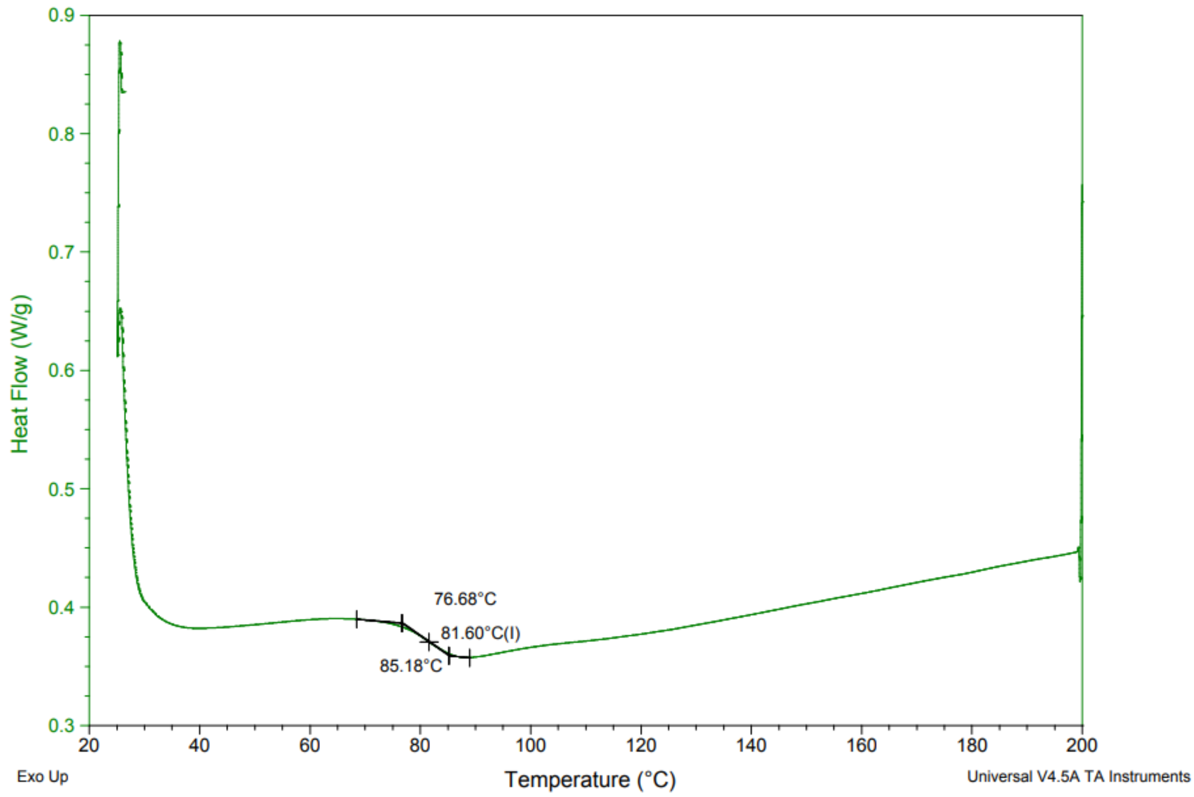

GPC Data Report

Agilent GPC/SEC Software
Sample Triple Analysis Report

Agilent Technologies

cw664

**Workspace Details**	
Workspace name	Polylactide
Location	C:\ProgramData\Agilent Technologies\GPC\Workspaces\Polylactide\
Comments	
Created by	Administrator at 13:44:31 on 15 June 2015
**Sample Properties**	
Sample name	cw664
File name	ICF_06_10_2020-0007.sample
Collected by	GPC at 13:39:48 on 06 October 2020
Instrument name	Instrument 1
**System Calibration Used**	
Created by	Administrator at 13:44:30 on 15 June 2015
Last modified by	Administrator at 13:44:30 on 15 June 2015
Comments	
Sample file used for calibration	ICF_6_18_2015-0002.sample
K (RI)	(RI) 750.958
K (LS 90°)	(RI) 30220.130
K (LS 15°)	(RI) 9870.135
K (VS DP)	(RI) 1.107
**System IDD Used**	
Last modified by	Administrator at 15:52:14 on 18 June 2015
Comments	
Reference detector	LS 90°
IDD Light scatterer (secs)	0.0
IDD RI (secs)	-3.0
IDD Viscometer (secs)	-14.0

Analyst: .... **Date:** ....

Checked By: .... **Date:** ....

Agilent GPC/SEC Software
Sample Triple Analysis Report

Agilent Technologies

Processing Parameters

Method	Last modified by Administrator at 13:44:30 on 15 June 2015
Concentration Detector Used in Analysis	RI
Injection volume (µL)	100.00
Flow rate (mL/min)	1.00
Concentration options	Calculate Sample Properties from Entered Sample Concentration
Entered sample concentration (mg/mL)	1.000
Calculated dn/dc (mL/g)	0.074
Calculated Ext Coeff ([(mg/mL)-¹] [cm-¹])	0.000
MW calculation method	Use all angles
Log Mi-v-RT curve fit options	Set the fit limits using the limits at peak width of 10 %.
Polynomial curve fit order	1
Use Constant Inlet Pressure	No
Flory-Fox	2.86e+021
DP Multiplier (mV to Pa)	1.0000
IP Multiplier (mV to kPa)	0.1000
Use IV To Calculate Rg	No

MW Ranges Method

Calculate MW Ranges	No

Percentage Fractions Method

Calculate Percentage Fractions	No

Results

Analysed by	GPC at 10:21:43 on 07 October 2020
Comments	

Peak Results

	Detector Type	Peak Max RT (mins)	Bulk MW (g/mol)	Bulk IV (dL/g)	Peak Height (mV)	Peak Height (%)	Peak Area (mV.s)	Area (%)	Conc. (mg/mL)
Peak 1	RI	12.50000	358485	0.098300	2.034	100.00	335.201	100.00	1.000
Recovery (%)	100.00								

Molecular Weight Averages

Peak	Mp (g/mol)	Mn (g/mol)	Mw (g/mol)	Mz (g/mol)	Mz+1 (g/mol)	Mv (g/mol)	PD
Peak 1	331914	174508	358939	576112	750476	559361	2.057

Rh Results

Peak	Slope	Intercept	Rhp (nm)	Rhn (nm)	Rhw (nm)	Rhz (nm)	Rhz+1 (nm)
Peak 1	0.6343	-2.602	7.94	5.28	8.34	11.26	13.32

IV Results

Peak	K ((10e-5) dL/g)	Alpha	IVp (dL/g)	IVn (dL/g)	IVw (dL/g)	IVz (dL/g)	IVz+1 (dL/g)
Peak 1	0.2902	0.819	0.096435	0.056960	0.102820	0.151483	0.188107

Analyst: .... **Date:** ....

Checked By: .... **Date:** ....

Agilent GPC/SEC Software
Sample Triple Analysis Report

Agilent Technologies

Peak Information

	Start (mins)	End (mins)
Baseline region 1	3.53333	6.05000
Baseline region 2	30.65000	32.76667
Peak 1	10.86667	15.38333

Peak Trace Information

Peak	Trace	Peak Max RT (mins)	Peak Area (mV.s)	Peak Height (mV)
Peak 1	RI	12.50000	335.201	2.034
Peak 1	VS DP	11.76667	573.704	4.582
Peak 1	VS IP	13.75000	63.741	0.601
Peak 1	LS 90°	11.58333	1883.926	15.982
Peak 1	LS 15°	11.48333	579.317	5.001

Log Mi Curves

Peak	Start (mins)	End (mins)	Polynomial Degree	Coeff a	Coeff b	Coeff c	Coeff d	Coeff e	Coeff f	L Point Y/N
Peak 1	10.8667	15.3667	1	10.063926	-0.36343203	0	0	0	0	N

Structural Plot Region

Peak	Start (mins)	End (mins)
Peak 1	10.8667	15.3667

Chromatogram Plot

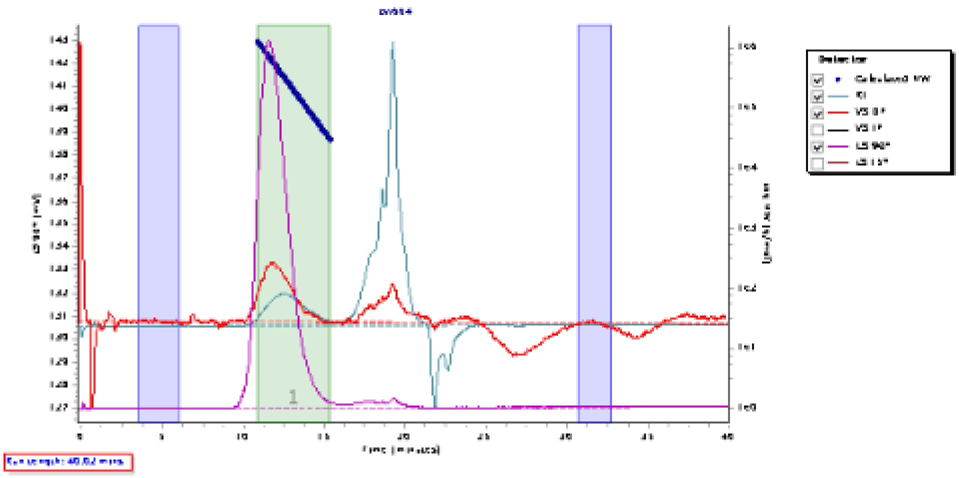

Analyst: .... **Date:** ....

Checked By: .... **Date:** ....

Agilent GPC/SEC Software
Sample Triple Analysis Report

Agilent Technologies

Distribution Plot

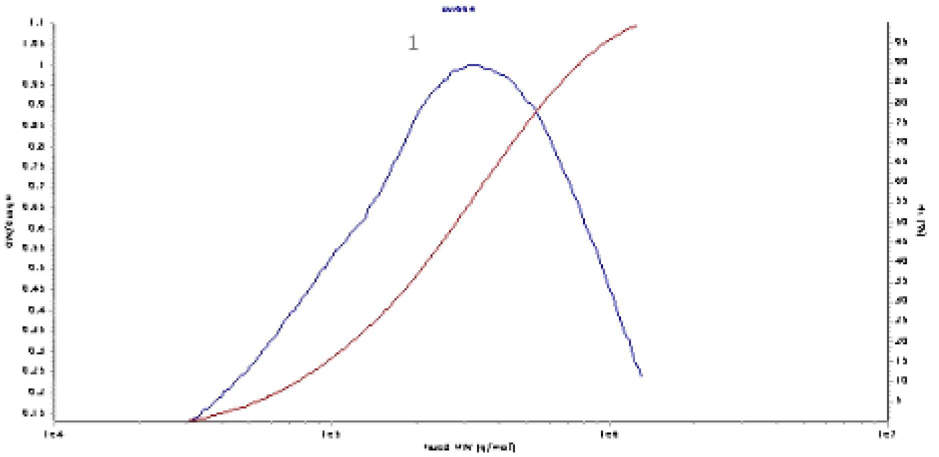

Conformation Plot

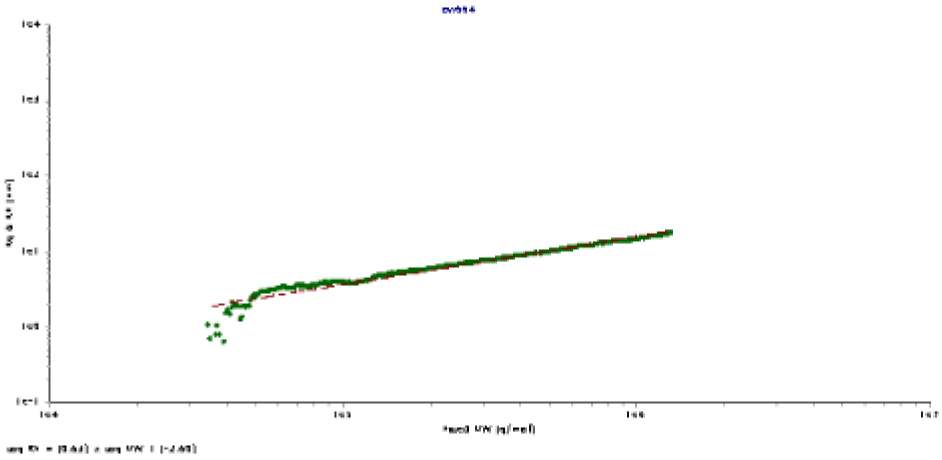

Analyst: .... **Date:** ....

Checked By: .... **Date:** ....

Mark-Houwink Plot

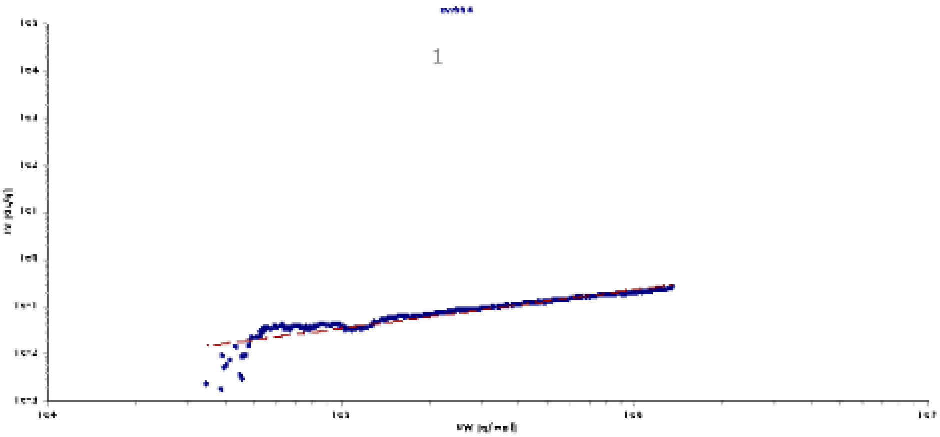

[illegible]

Analyst: ....   **Date:** ....

Checked By: ....   **Date:** ....

Poly(4-fluorophenyl)allene (P6)

NMR

The major (2,3) polymer signals are denoted with ●, the minor (1,2) signals are denoted with ●

^{1}H

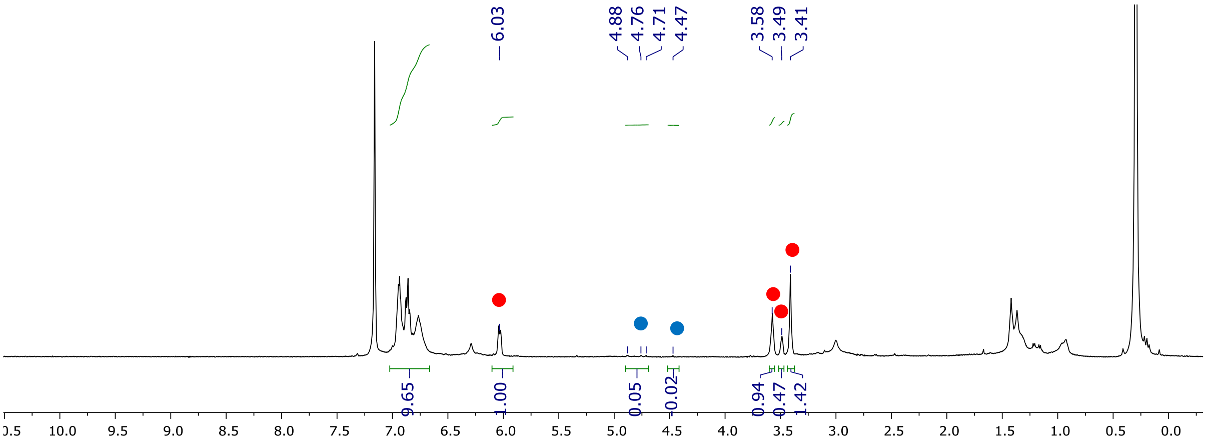

$^{13}C\{^{1}H\}$ (Major product assigned only)

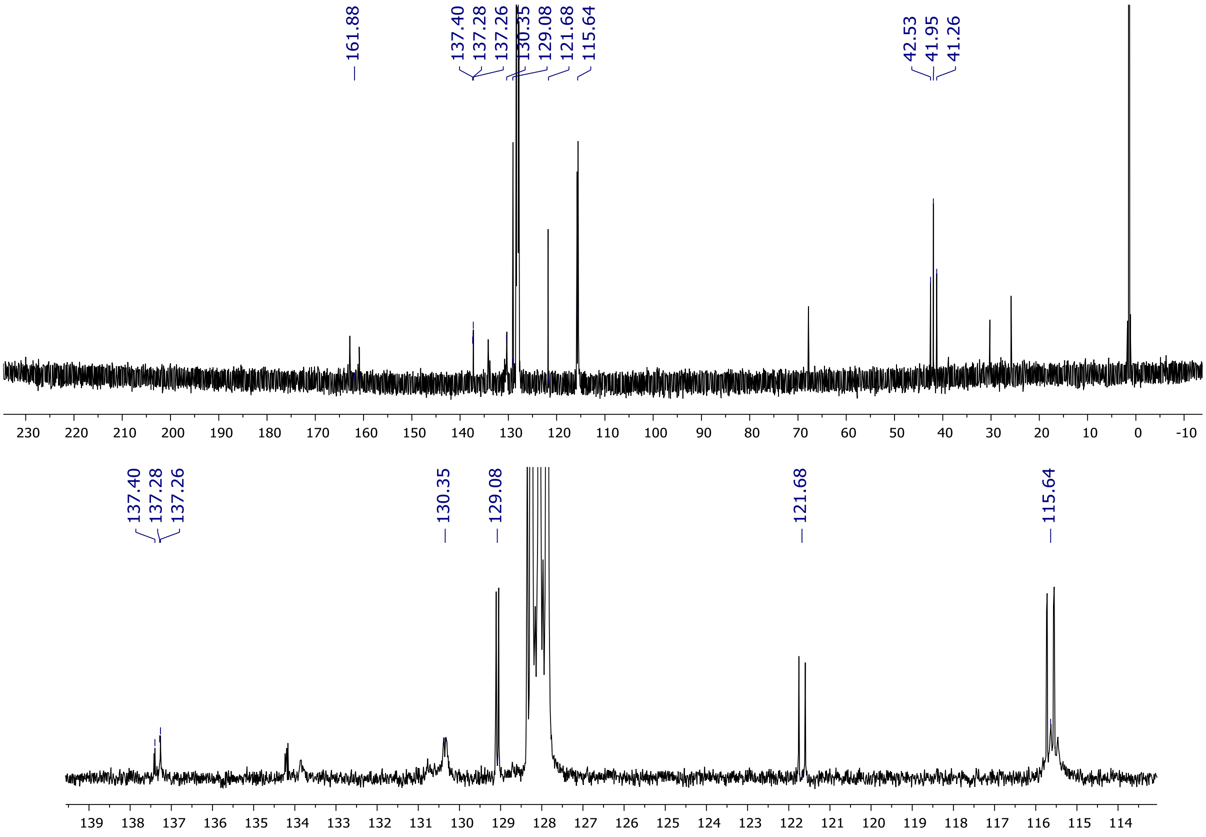

^{19}F

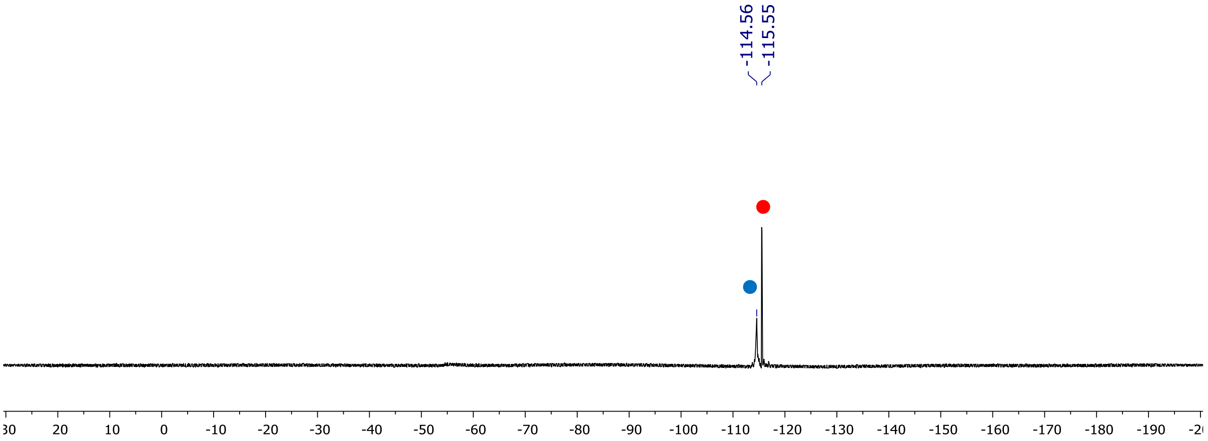

Summary of correlating peaks

Nuclei	^{1}H δ / ppm	$^{13}C\{^{1}H\}$ δ / ppm
HC_3 / C_3	3.58	41.2
	3.49	41.9
	3.41	42.5

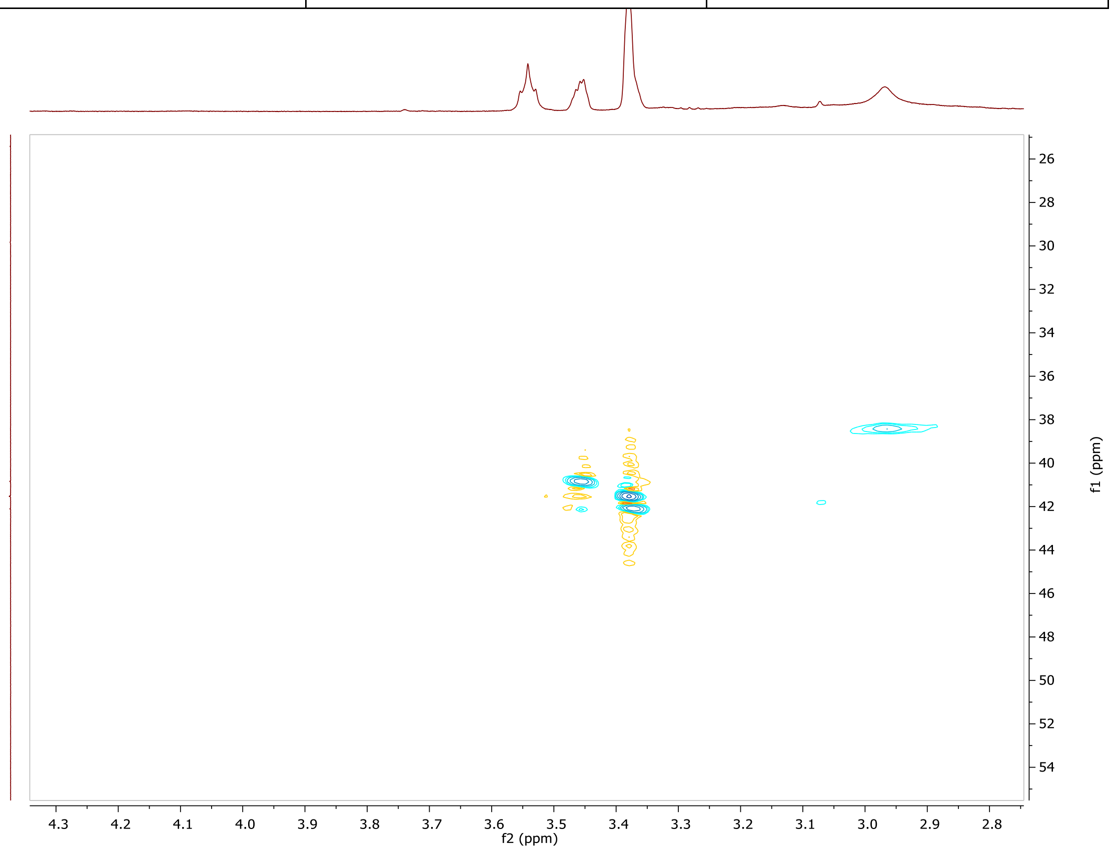

NOE NMR

nOe – 6.03 ppm peak

No strong correlation

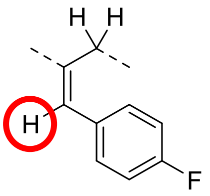

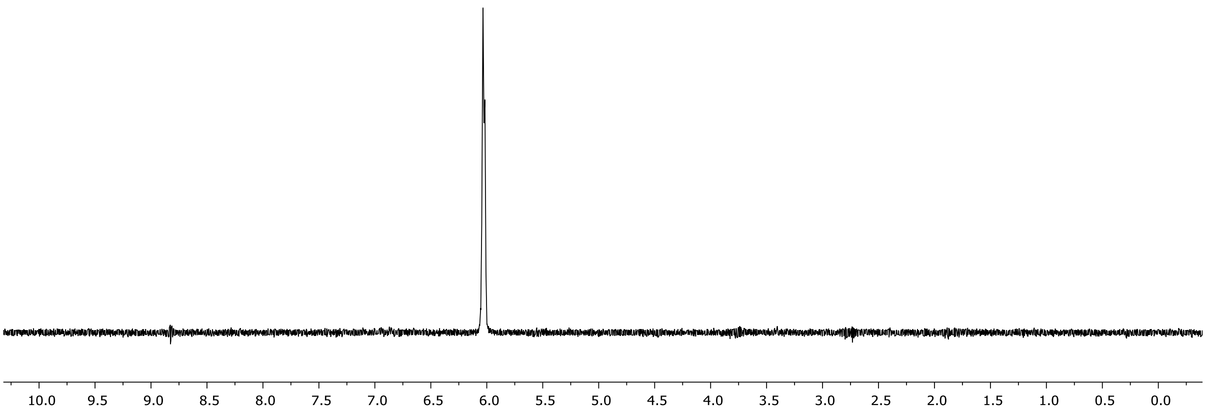

nOe – 3.57 ppm peak

No strong correlation

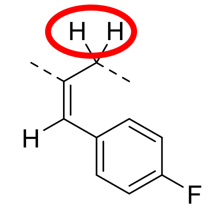

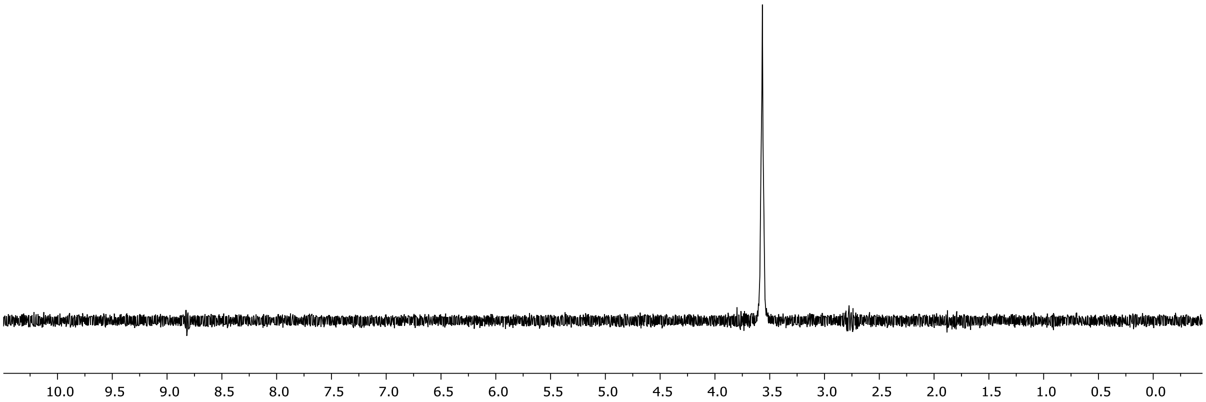

nOe – 3.48 ppm peak

Very weak correlation with aryl and alkenyl protons

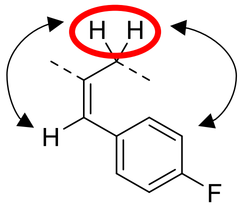

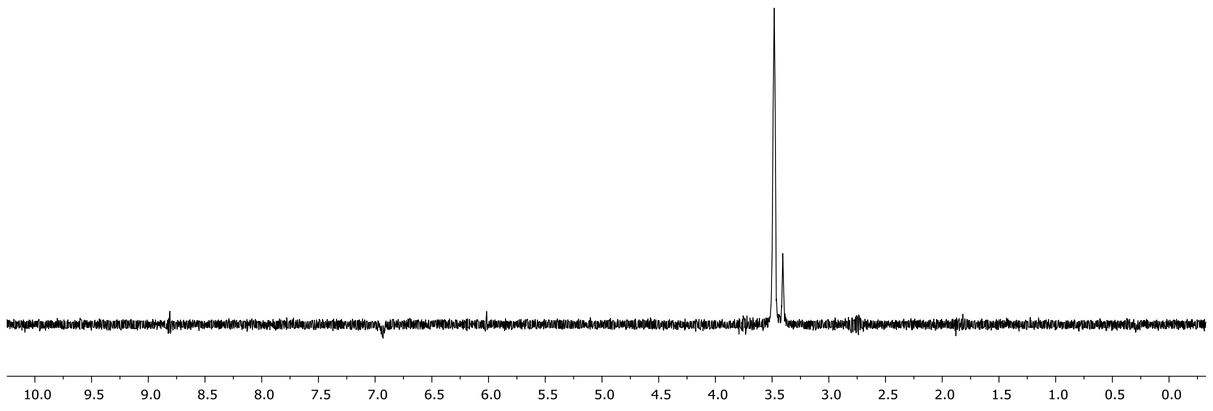

nOe – 3.40 ppm peak

No strong correlation

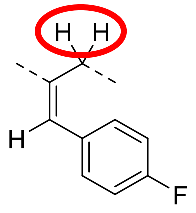

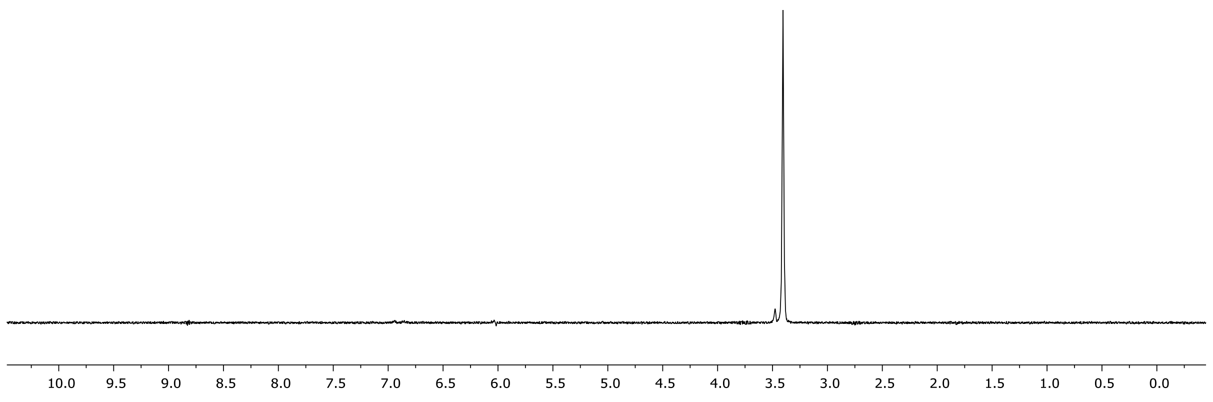

DSC Trace

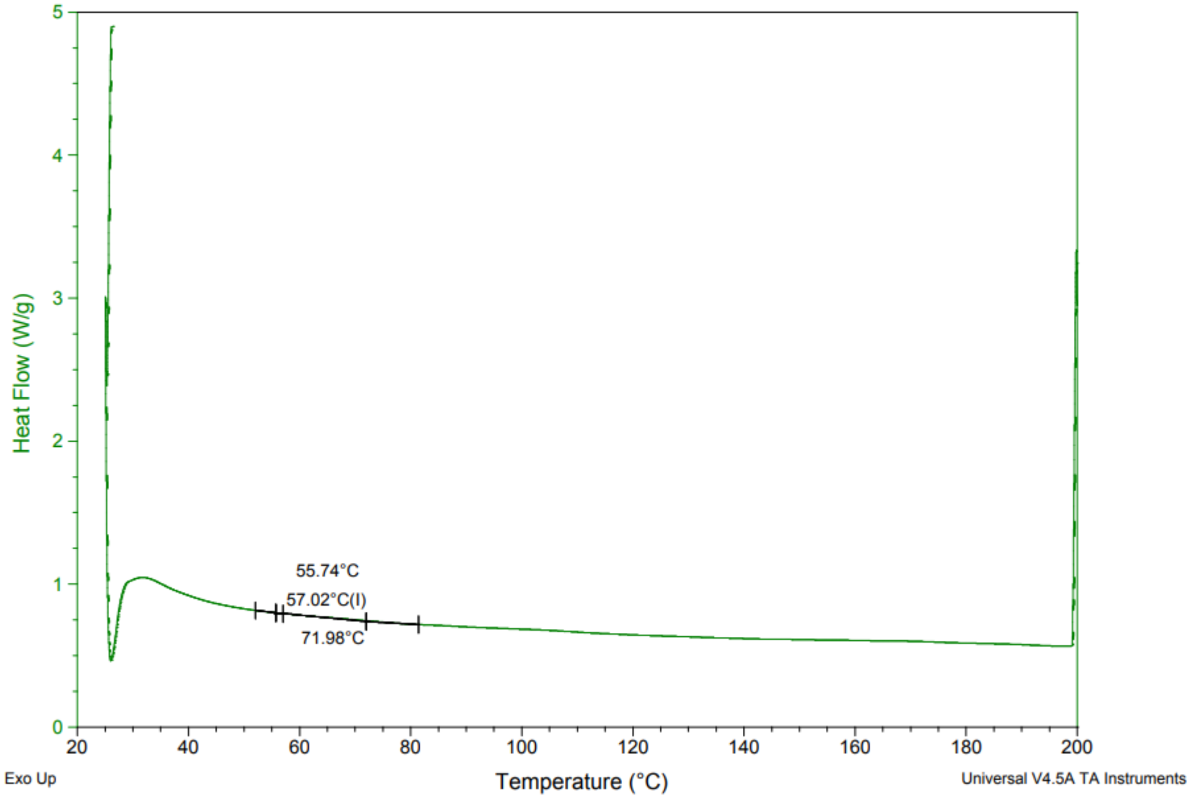

Heat Flow (W/g)
55.74°C
57.02°C(I)
71.98°C
Exo Up
Temperature (°C)
Universal V4.5A TA Instruments

GPC Data Report

Agilent GPC/SEC Software
Sample Triple Analysis Report

Agilent Technologies

cw674

**Workspace Details**	
Workspace name	Polylactide
Location	C:\ProgramData\Agilent Technologies\GPC\Workspaces\Polylactide\
Comments	
Created by	Administrator at 13:44:31 on 15 June 2015
**Sample Properties**	
Sample name	cw674
File name	ICF_21_09_2020-0008.sample
Collected by	GPC at 09:56:47 on 22 September 2020
Instrument name	Instrument 1
**System Calibration Used**	
Created by	Administrator at 13:44:30 on 15 June 2015
Last modified by	Administrator at 13:44:30 on 15 June 2015
Comments	
Sample file used for calibration	ICF_6_18_2015-0002.sample
K (RI)	(RI) 750.958
K (LS 90°)	(RI) 30220.130
K (LS 15°)	(RI) 9870.135
K (VS DP)	(RI) 1.107
**System IDD Used**	
Last modified by	Administrator at 15:52:14 on 18 June 2015
Comments	
Reference detector	LS 90°
IDD Light scatterer (secs)	0.0
IDD RI (secs)	-3.0
IDD Viscometer (secs)	-14.0

Analyst: .... **Date:** ....

Checked By: .... **Date:** ....

Agilent GPC/SEC Software
Sample Triple Analysis Report

Agilent Technologies

Processing Parameters

Method	Last modified by Administrator at 13:44:30 on 15 June 2015
Concentration Detector Used in Analysis	RI
Injection volume (µL)	100.00
Flow rate (mL/min)	1.00
Concentration options	Calculate Sample Properties from Entered Sample Concentration
Entered sample concentration (mg/mL)	1.000
Calculated dn/dc (mL/g)	0.408
Calculated Ext Coeff ([(mg/mL)-¹] [cm-¹])	0.000
MW calculation method	Use all angles
Log Mi-v-RT curve fit options	Set the fit limits using the limits at peak width of 10 %.
Polynomial curve fit order	1
Use Constant Inlet Pressure	No
Flory-Fox	2.86e+021
DP Multiplier (mV to Pa)	1.0000
IP Multiplier (mV to kPa)	0.1000
Use IV To Calculate Rg	No

MW Ranges Method

Calculate MW Ranges	No

Percentage Fractions Method

Calculate Percentage Fractions	No

Results

Analysed by	GPC at 11:26:06 on 22 September 2020
Comments	

Peak Results

	Detector Type	Peak Max RT (mins)	Bulk MW (g/mol)	Bulk IV (dL/g)	Peak Height (mV)	Peak Height (%)	Peak Area (mV.s)	Area (%)	Conc. (mg/mL)
Peak 1	RI	10.01667	866006	4.209279	17.809	79.16	1216.120	66.10	0.661
Peak 2	RI	13.08333	59891	0.670403	4.687	20.84	623.691	33.90	0.339
Recovery (%)	100.00								

Molecular Weight Averages

Peak	Mp (g/mol)	Mn (g/mol)	Mw (g/mol)	Mz (g/mol)	Mz+1 (g/mol)	Mv (g/mol)	PD
Peak 1	1062977	588632	868177	1108705	1292763	1074675	1.475
Peak 2	66504	53302	59710	66110	71837	64851	1.12

Rg Results

Peak	Slope	Intercept	Rgp (nm)	Rgn (nm)	Rgw (nm)	Rgz (nm)	Rgz+1 (nm)
Peak 1	0.4108	-0.8266	44.45	34.93	40.93	45.22	48.15
Peak 2	-0.4694	3.658	24.80	27.50	26.08	24.87	23.92

Analyst: .... **Date:** ....

Checked By: .... **Date:** ....

Agilent GPC/SEC Software
Sample Triple Analysis Report

Agilent Technologies

Rh Results

Peak	Slope	Intercept	Rhp (nm)	Rhn (nm)	Rhw (nm)	Rhz (nm)	Rhz+1 (nm)
Peak 1	0.5555	-1.707	43.59	31.42	38.97	44.61	48.57
Peak 2	0.5161	-1.528	9.13	8.14	8.64	9.11	9.51

IV Results

Peak	K ((10e-5) dL/g)	Alpha	IVp (dL/g)	IVn (dL/g)	IVw (dL/g)	IVz (dL/g)	IVz+1 (dL/g)
Peak 1	52.83	0.6586	4.921879	3.334823	4.307516	5.060330	5.599013
Peak 2	110.8	0.5842	0.727736	0.639485	0.683332	0.725216	0.761280

Peak Information

	Start (mins)	End (mins)
Baseline region 1	3.55000	5.01667
Baseline region 2	33.45000	35.85000
Peak 1	8.93333	11.83333
Peak 2	12.18333	14.86667

Peak Trace Information

Peak	Trace	Peak Max RT (mins)	Peak Area (mV.s)	Peak Height (mV)
Peak 1	RI	10.01667	1216.120	17.809
Peak 1	VS DP	9.95000	16001.364	281.110
Peak 1	VS IP	9.86667	512.769	9.196
Peak 1	LS 90°	9.95000	60841.231	1124.258
Peak 1	LS 15°	9.88333	27437.091	508.885
Peak 2	RI	13.08333	623.691	4.687
Peak 2	VS DP	12.18333	1300.413	12.589
Peak 2	VS IP	12.28333	39.604	0.632
Peak 2	LS 90°	12.18333	2772.870	34.439
Peak 2	LS 15°	12.18333	983.388	12.177

Log Mi Curves

Peak	Start (mins)	End (mins)	Polynomial Degree	Coeff a	Coeff b	Coeff c	Coeff d	Coeff e	Coeff f	L Point Y/N
Peak 1	9.4000	11.8167	1	10.745477	-0.47111012	0	0	0	0	N
Peak 2	12.1833	14.8500	1	7.4625526	-0.202018	0	0	0	0	N

Structural Plot Region

Peak	Start (mins)	End (mins)
Peak 1	9.4000	11.8167
Peak 2	12.1833	14.8500

Analyst: .... **Date:** ....

Checked By: .... **Date:** ....

Agilent Technologies

Chromatogram Plot

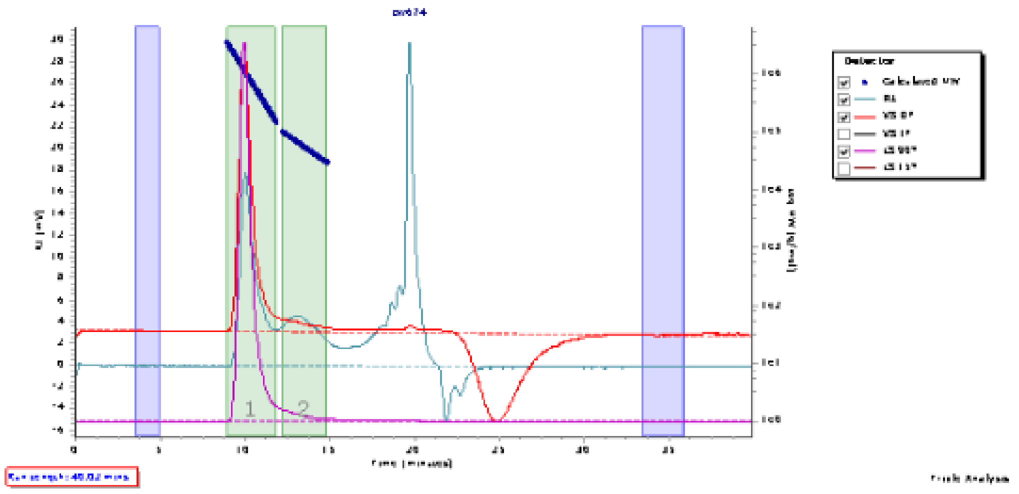

Distribution Plot

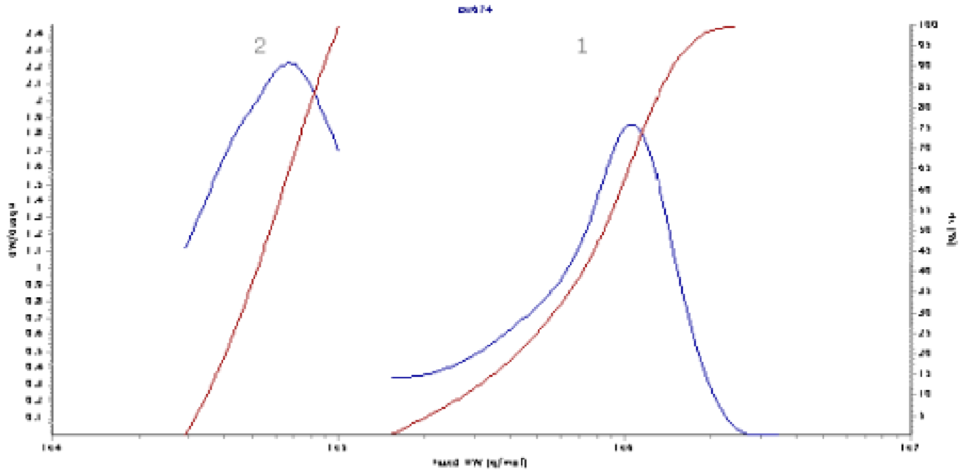

Analyst: .... **Date:** ....

Checked By: .... **Date:** ....

Conformation Plot

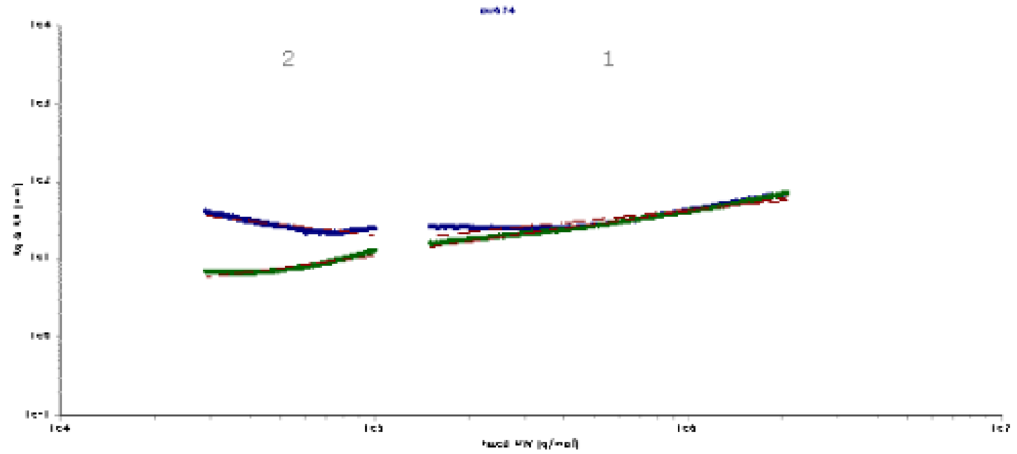

Peak 1 Rg (z3): Log Rg = (0.41) x Log MW + (-0.8[illegible]), Log Rh = (0.56) x Log MW + (-1.71)
Peak 2 Rg (z3): Log Rg = (-0.47) x Log MW + (3.66), Log Rh = (0.52) x Log MW + (-1.53)

Mark-Houwink Plot

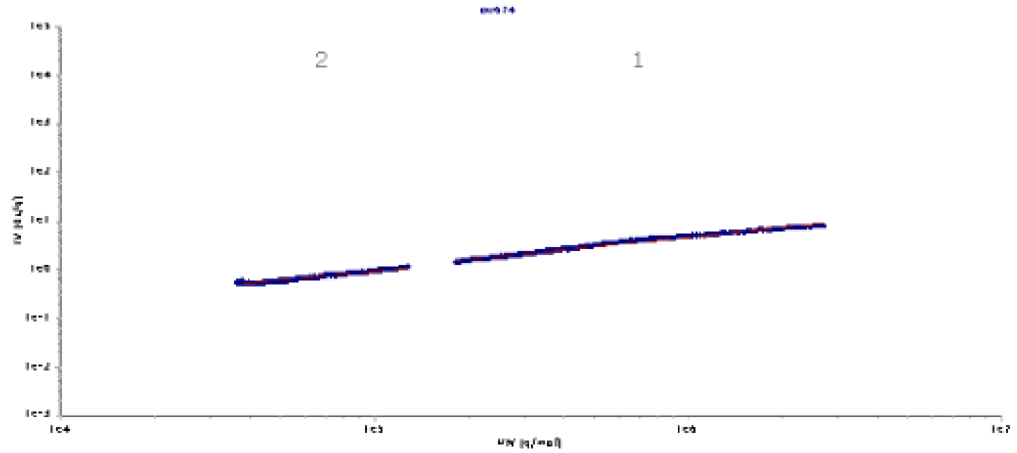

Peak 1: K = ((10e-5) dL/g) 52.[illegible], Alpha = 0.66
Peak 2: K = ((10e-5) dL/g) 110.77, Alpha = 0.52

Analyst: .... **Date:** ....

Checked By: .... **Date:** ....

Poly(4-chlorophenyl)allene (P7)

NMR

The major (2,3) polymer signals are denoted with ●, the minor (1,2) signals are denoted with ●

^{1}H

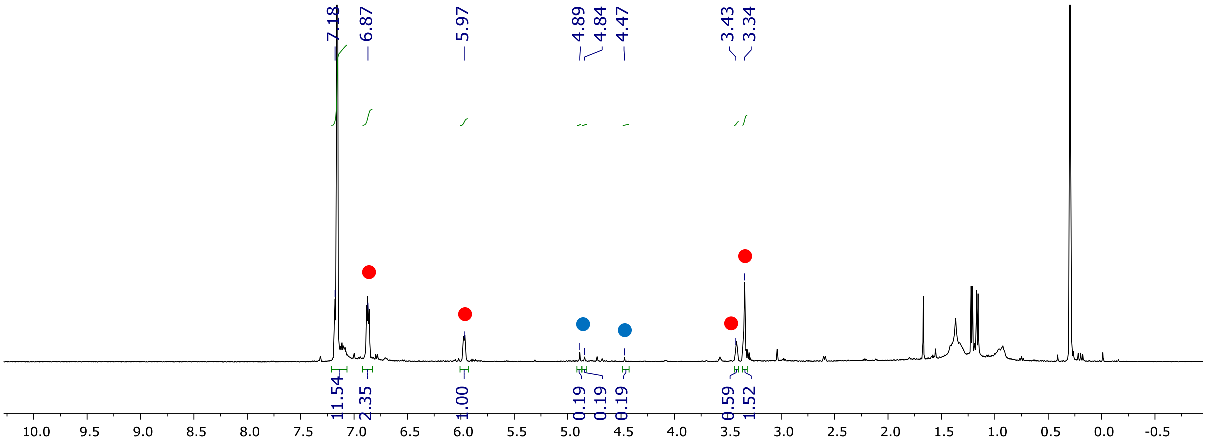

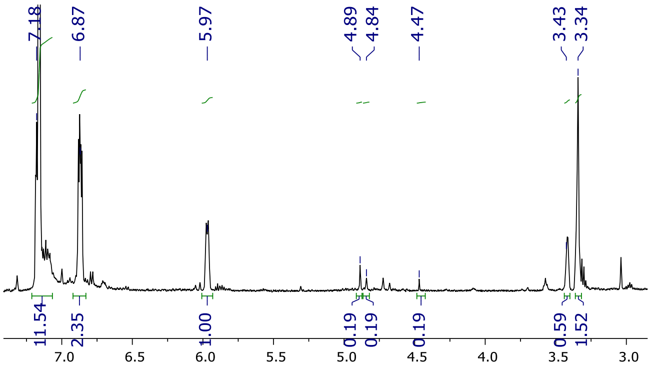

$^{13}C\{^{1}H\}$

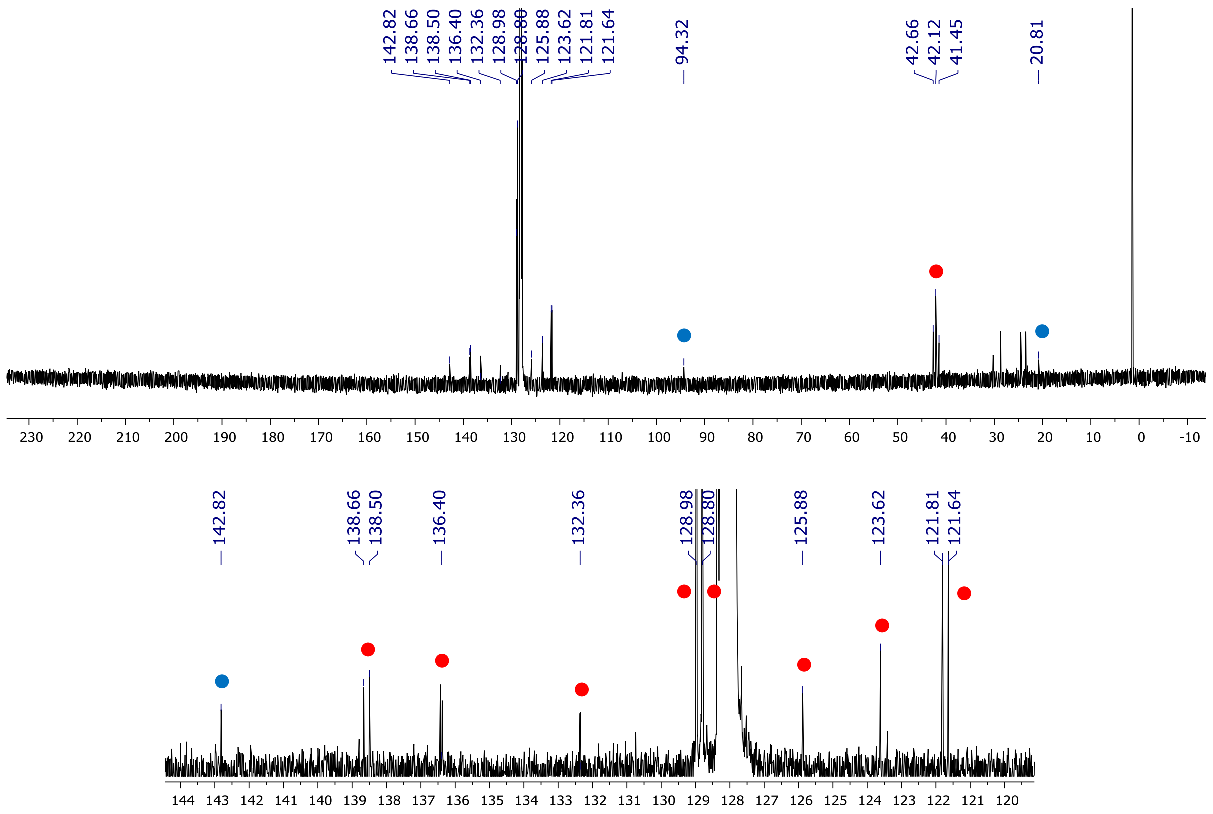

Summary of Correlating Peaks

It is likely that the ^{1}H δ = 3.34 ppm peak corresponds to two distinct environments as it correlates with two $^{13}C\{^{1}H\}$ signals

Nuclei	^{1}H δ / ppm	$^{13}C\{^{1}H\}$ δ / ppm
*H*C₃ / C₃	3.43	41.5
	3.34	42.1
		42.6

HSQC of correlating area (500 MHz, C_6D_6)

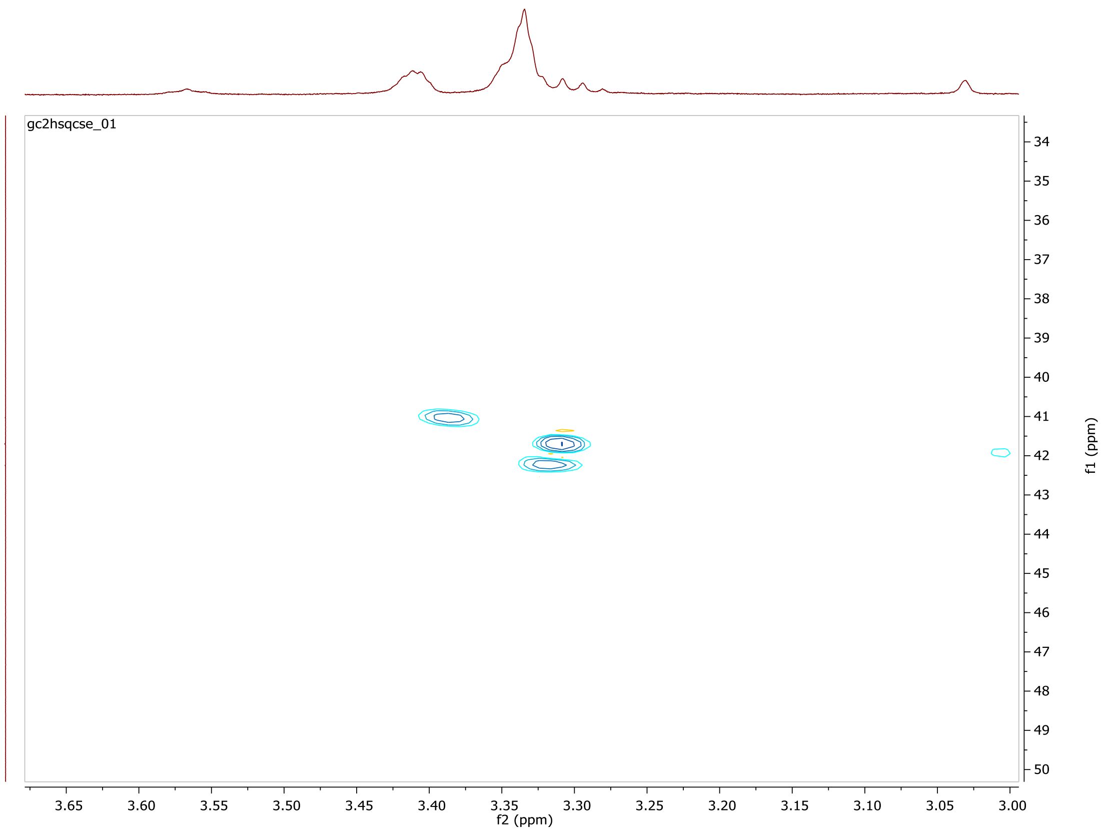

NOE NMR

nOe – 5.96 ppm peak

No strong correlation

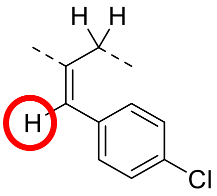

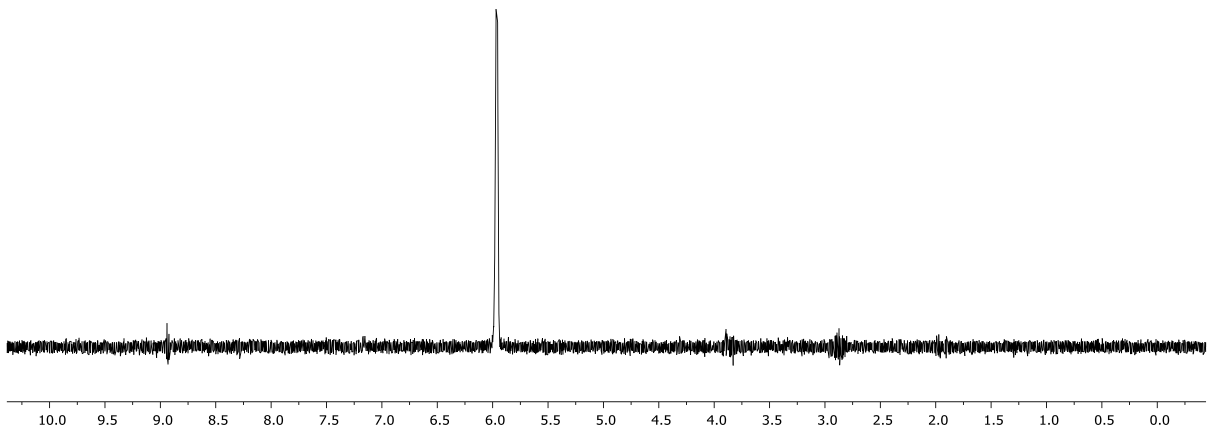

nOe – 3.43 ppm peak

Some correlation with aryl region

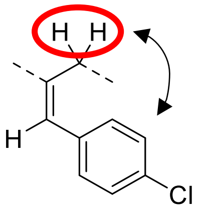

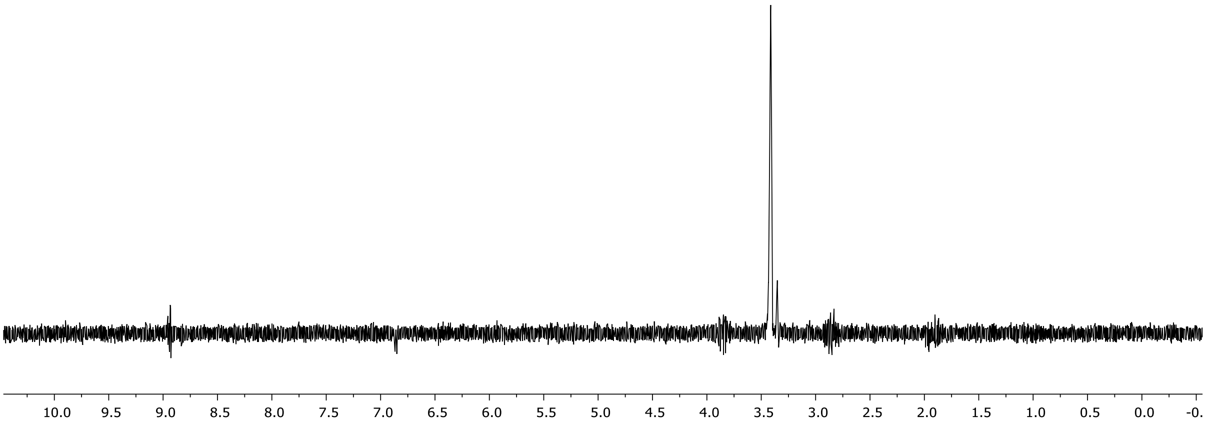

nOe – 3.34 ppm peak

No strong correlation observed

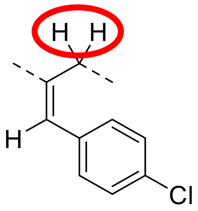

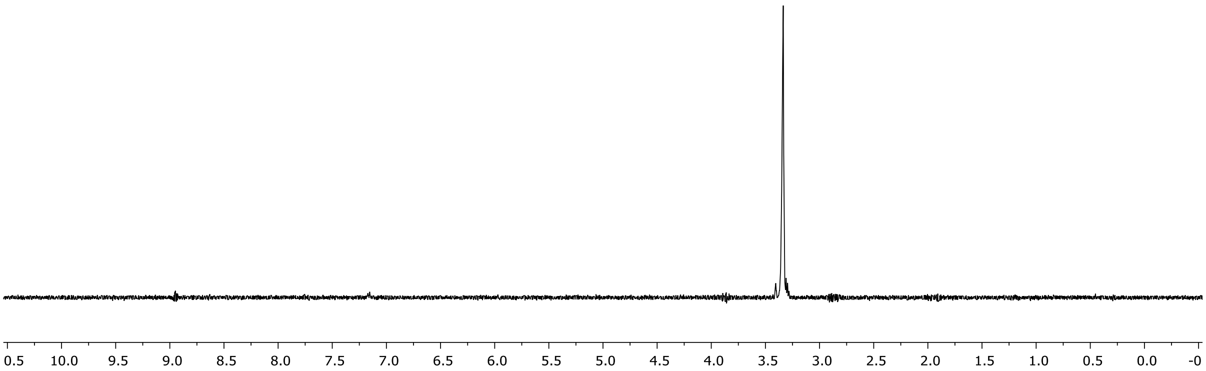

DSC Trace

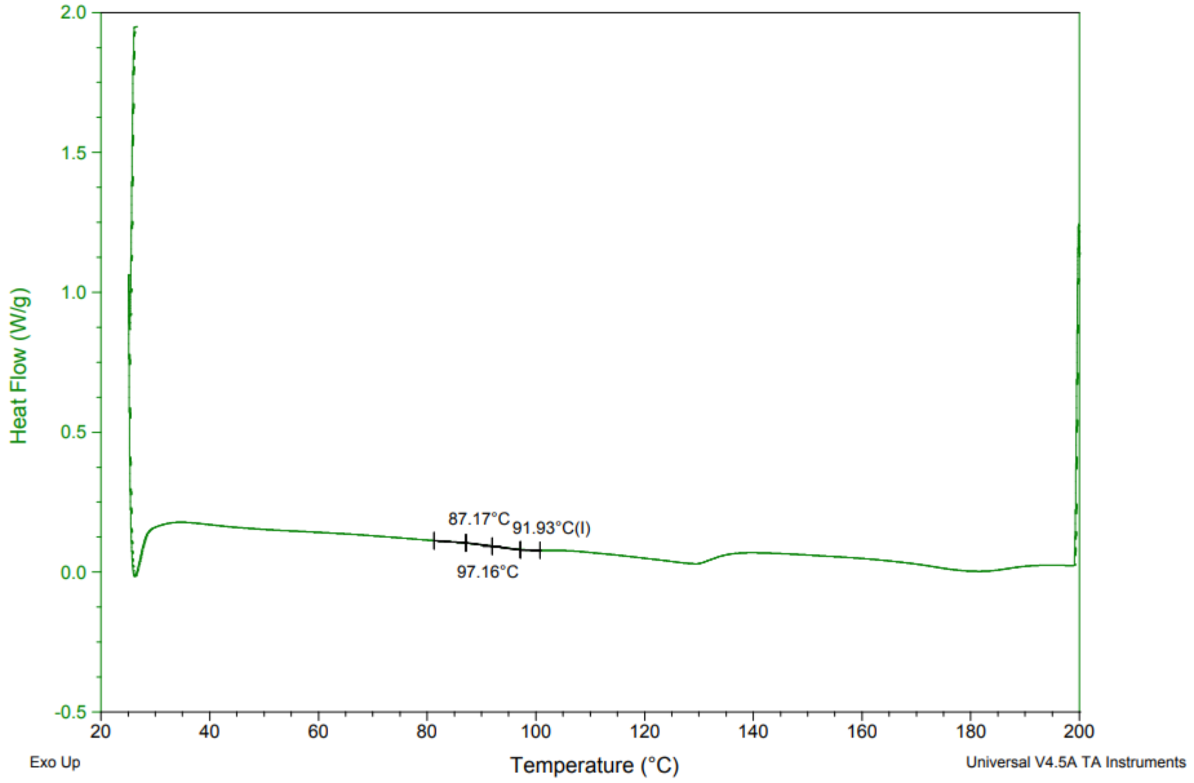

2.0
1.5
1.0
0.5
0.0
-0.5
Heat Flow (W/g)
87.17°C
91.93°C(I)
97.16°C
20
40
60
80
100
120
140
160
180
200
Exo Up
Temperature (°C)
Universal V4.5A TA Instruments

GPC Data Report

Agilent GPC/SEC Software
Sample Triple Analysis Report

Agilent Technologies

cw731

**Workspace Details**	
Workspace name	Polylactide
Location	C:\ProgramData\Agilent Technologies\GPC\Workspaces\Polylactide\
Comments	
Created by	Administrator at 13:44:31 on 15 June 2015
**Sample Properties**	
Sample name	cw731
File name	ICF_18_01_2021-0021.sample
Collected by	GPC at 13:16:31 on 20 January 2021
Instrument name	Instrument 1
**System Calibration Used**	
Created by	Administrator at 13:44:30 on 15 June 2015
Last modified by	Administrator at 13:44:30 on 15 June 2015
Comments	
Sample file used for calibration	ICF_6_18_2015-0002.sample
K (RI)	(RI) 750.958
K (LS 90°)	(RI) 30220.130
K (LS 15°)	(RI) 9870.135
K (VS DP)	(RI) 1.107
**System IDD Used**	
Last modified by	Administrator at 15:52:14 on 18 June 2015
Comments	
Reference detector	LS 90°
IDD Light scatterer (secs)	0.0
IDD RI (secs)	-3.0
IDD Viscometer (secs)	-14.0

Analyst: .... **Date:** ....

Checked By: .... **Date:** ....

Agilent GPC/SEC Software
Sample Triple Analysis Report

Agilent Technologies

Processing Parameters

Method	Last modified by Administrator at 13:44:30 on 15 June 2015
Concentration Detector Used in Analysis	RI
Injection volume (µL)	100.00
Flow rate (mL/min)	1.00
Concentration options	Calculate Sample Properties from Entered Sample Concentration
Entered sample concentration (mg/mL)	1.000
Calculated dn/dc (mL/g)	0.034
Calculated Ext Coeff ([(mg/mL)$^{-1}$] [cm^{-1}])	0.000
MW calculation method	Use all angles
Log Mi-v-RT curve fit options	Set the fit limits using the limits at peak width of 10 %.
Polynomial curve fit order	1
Use Constant Inlet Pressure	No
Flory-Fox	2.86e+021
DP Multiplier (mV to Pa)	1.0000
IP Multiplier (mV to kPa)	0.1000
Use IV To Calculate Rg	No

MW Ranges Method

Calculate MW Ranges	No

Percentage Fractions Method

Calculate Percentage Fractions	No

Results

Analysed by	GPC at 14:31:09 on 20 January 2021
Comments	

Peak Results

	Detector Type	Peak Max RT (mins)	Bulk MW (g/mol)	Bulk IV (dL/g)	Peak Height (mV)	Peak Height (%)	Peak Area (mV.s)	Area (%)	Conc. (mg/mL)
Peak 1	RI	10.58333	77234935	0.256350	0.119	10.62	8.936	5.85	0.059
Peak 2	RI	12.81667	590615	0.125327	1.002	89.38	143.775	94.15	0.941
Recovery (%)	100.00								

Molecular Weight Averages

Peak	Mp (g/mol)	Mn (g/mol)	Mw (g/mol)	Mz (g/mol)	Mz+1 (g/mol)	Mv (g/mol)	PD
Peak 1	11477368	34193445	84222538	198599398	319510623	0	2.463
Peak 2	351410	166591	611364	1373424	1960306	1142000	3.67

Rg Results

Peak	Slope	Intercept	Rgp (nm)	Rgn (nm)	Rgw (nm)	Rgz (nm)	Rgz+1 (nm)
Peak 1	0.3179	-0.8262	26.15	37.03	49.36	64.88	75.49
Peak 2	-9.068E-16	1.967	92.64	92.64	92.64	92.64	92.64

Analyst: .... **Date:** ....

Checked By: .... **Date:** ....

Agilent GPC/SEC Software
Sample Triple Analysis Report

Agilent Technologies

Rh Results

Peak	Slope	Intercept	Rhp (nm)	Rhn (nm)	Rhw (nm)	Rhz (nm)	Rhz+1 (nm)
Peak 1	0.2976	-0.5483	35.80	49.38	64.41	82.94	95.41
Peak 2	0.4731	-1.692	8.53	5.99	11.09	16.26	19.24

IV Results

Peak	K ((10e-5) dL/g)	Alpha	IVp (dL/g)	IVn (dL/g)	IVw (dL/g)	IVz (dL/g)	IVz+1 (dL/g)
Peak 1	7.484E+04	-0.07148	0.234136	0.216561	0.203046	0.190970	0.184588
Peak 2	193.8	0.3185	0.113204	0.089251	0.135040	0.174753	0.195722

Peak Information

	Start (mins)	End (mins)
Baseline region 1	3.43333	5.76667
Baseline region 2	32.96667	36.36667
Peak 1	8.35000	10.58333
Peak 2	11.18333	14.66667

Peak Trace Information

Peak	Trace	Peak Max RT (mins)	Peak Area (mV.s)	Peak Height (mV)
Peak 1	RI	10.58333	8.936	0.119
Peak 1	VS DP	10.03333	87.882	1.285
Peak 1	VS IP	9.98333	22.810	-0.830
Peak 1	LS 90°	9.10000	2820.164	33.865
Peak 1	LS 15°	9.05000	1480.341	19.684
Peak 2	RI	12.81667	143.775	1.002
Peak 2	VS DP	11.68333	691.253	5.820
Peak 2	VS IP	14.45000	36.947	-0.818
Peak 2	LS 90°	11.76667	698.314	6.090
Peak 2	LS 15°	11.65000	187.297	1.892

Log Mi Curves

Peak	Start (mins)	End (mins)	Polynomial Degree	Coeff a	Coeff b	Coeff c	Coeff d	Coeff e	Coeff f	L Point Y/N
Peak 1	8.7167	10.5833	1	15.257042	-0.77576026	0	0	0	0	N
Peak 2	11.1833	14.6500	1	13.301556	-0.60591732	0	0	0	0	N

Structural Plot Region

Peak	Start (mins)	End (mins)
Peak 1	8.7167	10.5833
Peak 2	11.1833	14.6500

Analyst: .... **Date:** ....

Checked By: .... **Date:** ....

Agilent GPC/SEC Software
Sample Triple Analysis Report

Agilent Technologies

Chromatogram Plot

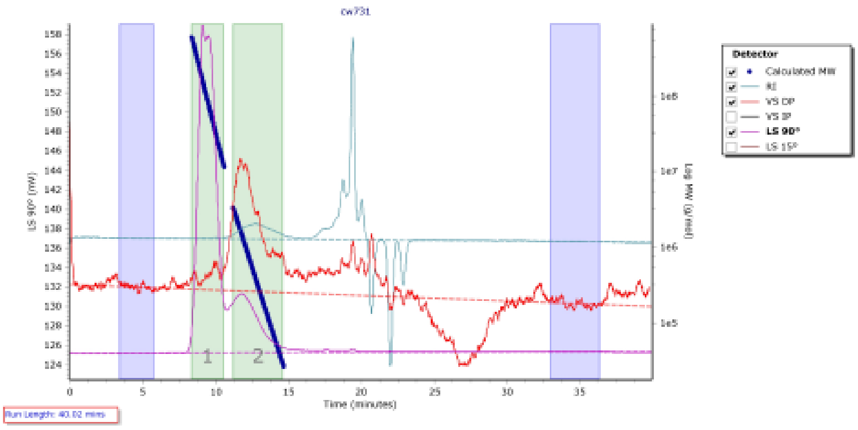

Distribution Plot

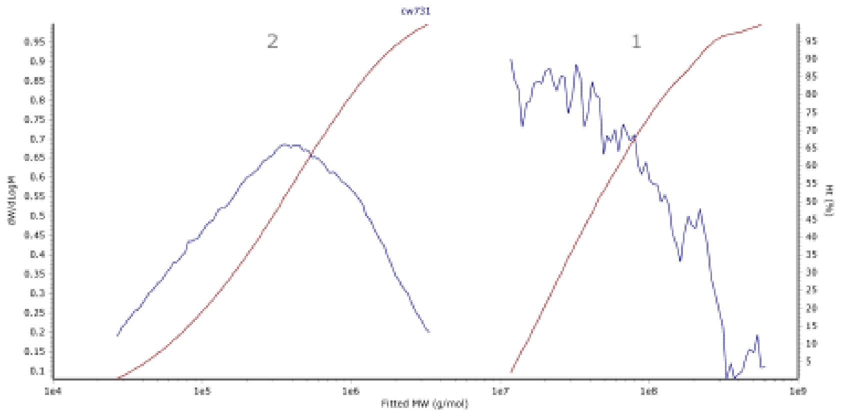

Analyst: .... **Date:** ....

Checked By: .... **Date:** ....

Agilent GPC/SEC Software A.02.01 [9] Page 4 of 5

Generated by GPC at 14:32 on 20 January 2021

Agilent GPC/SEC Software
Sample Triple Analysis Report

Agilent Technologies

Conformation Plot

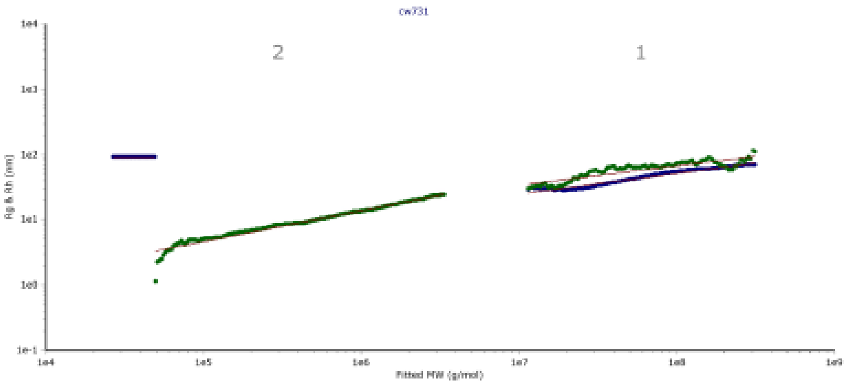

Peak 1 Rg (LS): Log Rg = (0.32) x Log MW + (-0.83), Log Rh = (0.30) x Log MW + (-0.55)
Peak 2 Rg (LS): Log Rg = (0.00) x Log MW + (1.97), Log Rh = (0.47) x Log MW + (-1.69)

Mark-Houwink Plot

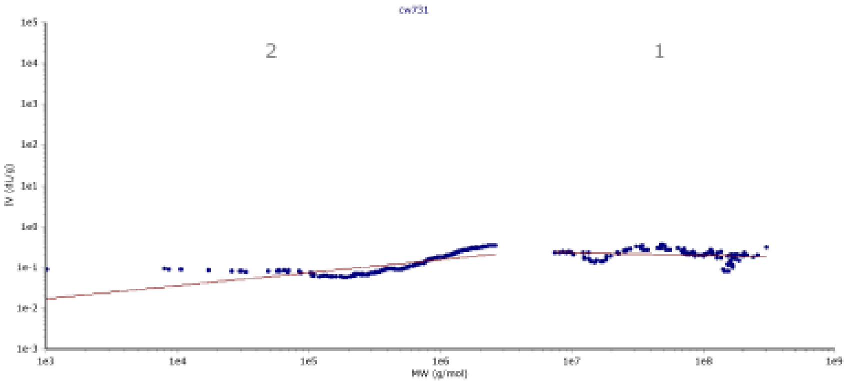

Peak 1: K = ((10e-5) dL/g) 74837.38, Alpha = -0.07
Peak 2: K = ((10e-5) dL/g) 193.83, Alpha = 0.32

Analyst: .... **Date:** ....

Checked By: .... **Date:** ....

Agilent GPC/SEC Software A.02.01 [9] Page 5 of 5

Generated by GPC at 14:32 on 20 January 2021

Dimeric Species

^1H

The *trans* dimer peaks are indicated by ● and the *cis* dimer peaks by ●. The aromatic carbons (C_1 through C_4) are indistinguishable between isomers

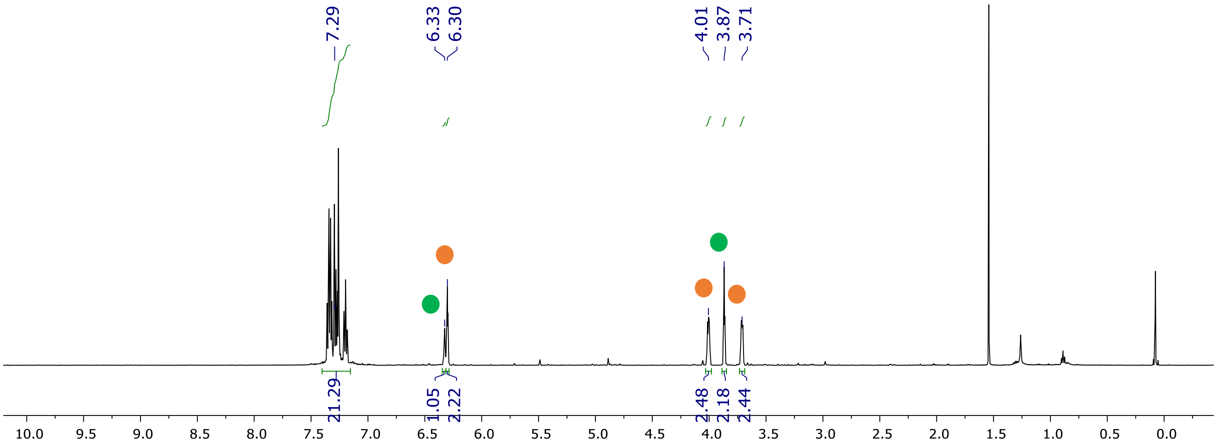

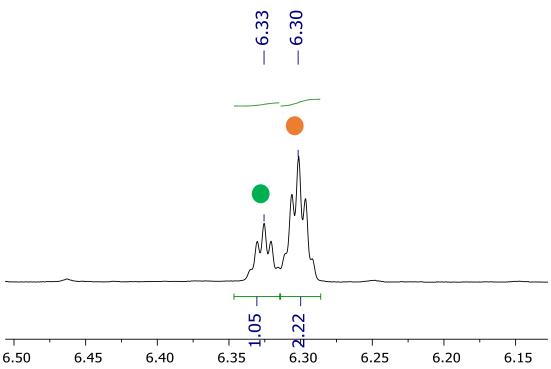

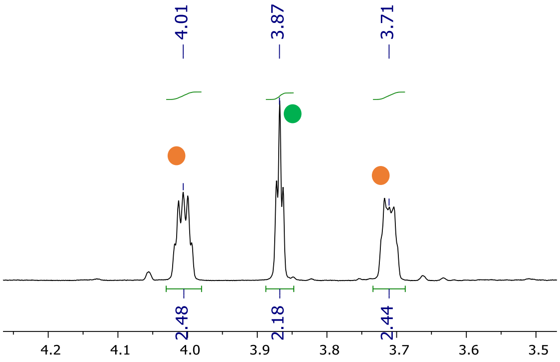

$^{13}C\{^{1}H\}$

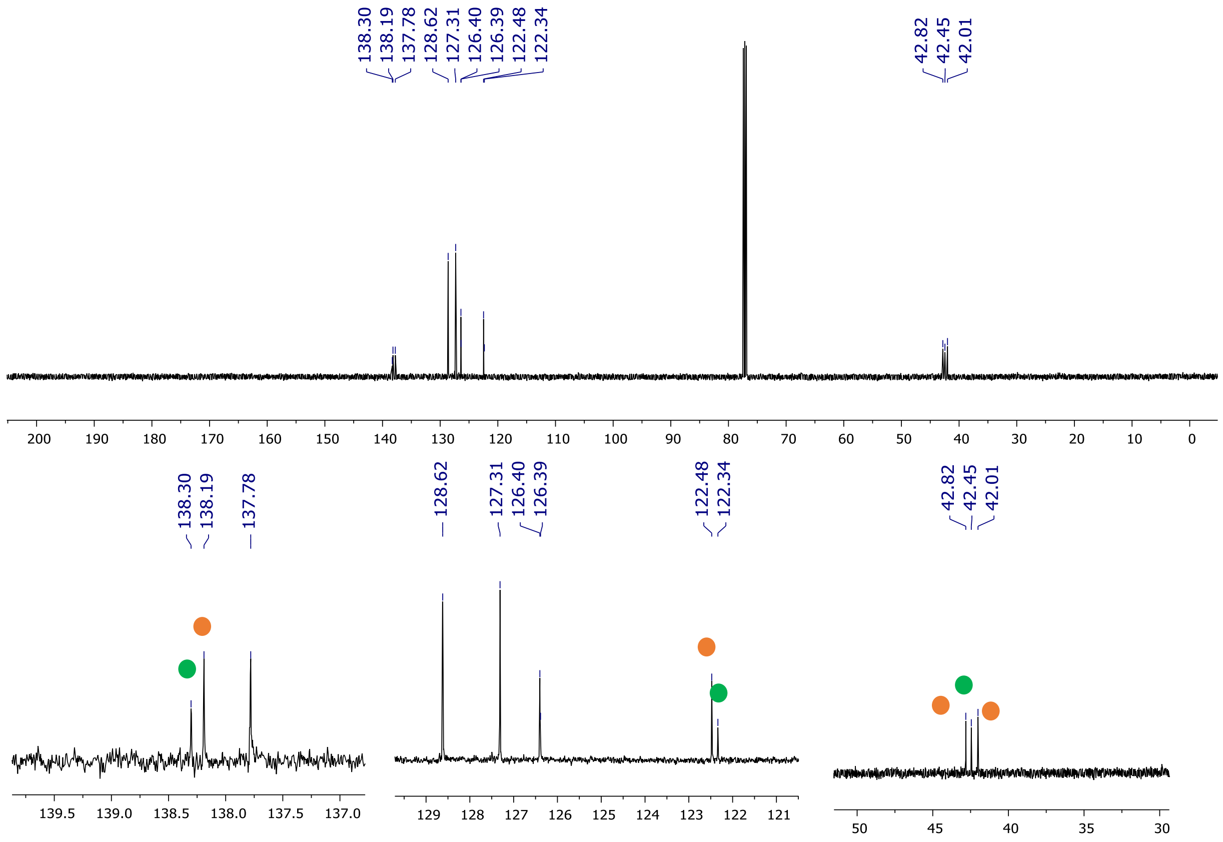
138.30
138.19
137.78
128.62
127.31
126.40
126.39
122.48
122.34
42.82
42.45
42.01
200 190 180 170 160 150 140 130 120 110 100 90 80 70 60 50 40 30 20 10 0
139.5 139.0 138.5 138.0 137.5 137.0
129 128 127 126 125 124 123 122 121
50 45 40 35 30

Further GPC Data

Using Fe(I) Species in Catalysis

Agilent GPC/SEC Software
Sample Triple Analysis Report

Agilent Technologies

CW753-1

**Workspace Details**	
Workspace name	Polylactide
Location	C:\ProgramData\Agilent Technologies\GPC\Workspaces\Polylactide\
Comments	
Created by	Administrator at 13:44:31 on 15 June 2015
**Sample Properties**	
Sample name	CW753-1
File name	ICF_02_03_2021-0030.sample
Collected by	GPC at 10:51:14 on 04 March 2021
Instrument name	Instrument 1
**System Calibration Used**	
Created by	Administrator at 13:44:30 on 15 June 2015
Last modified by	Administrator at 13:44:30 on 15 June 2015
Comments	
Sample file used for calibration	ICF_6_18_2015-0002.sample
K (RI)	(RI) 750.958
K (LS 90°)	(RI) 30220.130
K (LS 15°)	(RI) 9870.135
K (VS DP)	(RI) 1.107
**System IDD Used**	
Last modified by	Administrator at 15:52:14 on 18 June 2015
Comments	
Reference detector	LS 90°
IDD Light scatterer (secs)	0.0
IDD RI (secs)	-3.0
IDD Viscometer (secs)	-14.0

Analyst: .... **Date:** ....

Checked By: .... **Date:** ....

Agilent GPC/SEC Software A.02.01 [9] Page 1 of 5

Generated by GPC at 17:44 on 04 March 2021

Agilent GPC/SEC Software
Sample Triple Analysis Report

Agilent Technologies

Processing Parameters

Method	Last modified by Administrator at 13:44:30 on 15 June 2015
Concentration Detector Used in Analysis	RI
Injection volume (µL)	100.00
Flow rate (mL/min)	1.00
Concentration options	Calculate Sample Properties from Entered Sample Concentration
Entered sample concentration (mg/mL)	1.000
Calculated dn/dc (mL/g)	0.086
Calculated Ext Coeff ([(mg/mL)-¹] [cm-¹])	0.000
MW calculation method	Use all angles
Log Mi-v-RT curve fit options	Set the fit limits using the limits at peak width of 10 %.
Polynomial curve fit order	1
Use Constant Inlet Pressure	No
Flory-Fox	2.86e+021
DP Multiplier (mV to Pa)	1.0000
IP Multiplier (mV to kPa)	0.1000
Use IV To Calculate Rg	No

MW Ranges Method

Calculate MW Ranges	No

Percentage Fractions Method

Calculate Percentage Fractions	No

Results

Analysed by	GPC at 12:36:28 on 04 March 2021
Comments	

Peak Results

	Detector Type	Peak Max RT (mins)	Bulk MW (g/mol)	Bulk IV (dL/g)	Peak Height (mV)	Peak Height (%)	Peak Area (mV.s)	Area (%)	Conc. (mg/mL)
Peak 1	RI	9.71667	6273145	1.382789	1.980	51.81	122.135	31.54	0.315
Peak 2	RI	12.90000	280405	0.235553	1.842	48.19	265.084	68.46	0.685
Recovery (%)	100.00								

Molecular Weight Averages

Peak	Mp (g/mol)	Mn (g/mol)	Mw (g/mol)	Mz (g/mol)	Mz+1 (g/mol)	Mv (g/mol)	PD
Peak 1	9563666	5841782	8235440	10492958	12279977	10034796	1.41
Peak 2	277426	190061	286775	396470	495123	354320	1.509

Rg Results

Peak	Slope	Intercept	Rgp (nm)	Rgn (nm)	Rgw (nm)	Rgz (nm)	Rgz+1 (nm)
Peak 1	0.3861	-0.9137	60.51	50.02	57.12	62.72	66.65
Peak 2	-0.2866	2.732	14.87	16.57	14.73	13.42	12.59

Analyst: .... **Date:** ....

Checked By: .... **Date:** ....

Agilent GPC/SEC Software
Sample Triple Analysis Report

Agilent Technologies

Rh Results

Peak	Slope	Intercept	Rhp (nm)	Rhn (nm)	Rhw (nm)	Rhz (nm)	Rhz+1 (nm)
Peak 1	0.5142	-1.799	61.68	47.87	57.11	64.69	70.14
Peak 2	0.3688	-0.9882	10.45	9.09	10.58	11.92	12.94

IV Results

Peak	K ((10e-5) dL/g)	Alpha	IVp (dL/g)	IVn (dL/g)	IVw (dL/g)	IVz (dL/g)	IVz+1 (dL/g)
Peak 1	28.64	0.5345	1.540776	1.183926	1.422438	1.619063	1.761029
Peak 2	2236	0.1973	0.265178	0.246109	0.266918	0.284533	0.297286

Peak Information

	Start (mins)	End (mins)
Baseline region 1	5.03333	7.83333
Baseline region 2	27.08333	30.58333
Peak 1	9.03333	10.83333
Peak 2	11.25000	15.21667

Peak Trace Information

Peak	Trace	Peak Max RT (mins)	Peak Area (mV.s)	Peak Height (mV)
Peak 1	RI	9.71667	122.135	1.980
Peak 1	VS DP	9.53333	2553.706	53.952
Peak 1	VS IP	9.41667	83.111	2.154
Peak 1	LS 90°	9.53333	8813.981	179.095
Peak 1	LS 15°	9.45000	5439.498	152.149
Peak 2	RI	12.90000	265.084	1.842
Peak 2	VS DP	12.48333	943.245	5.395
Peak 2	VS IP	12.16667	38.420	0.771
Peak 2	LS 90°	12.20000	1214.527	9.481
Peak 2	LS 15°	11.25000	412.528	3.395

Log Mi Curves

Peak	Start (mins)	End (mins)	Polynomial Degree	Coeff a	Coeff b	Coeff c	Coeff d	Coeff e	Coeff f	L Point Y/N
Peak 1	9.2500	10.8167	1	13.439809	-0.66589535	0	0	0	0	N
Peak 2	11.2500	15.2000	1	9.339769	-0.30245444	0	0	0	0	N

Structural Plot Region

Peak	Start (mins)	End (mins)
Peak 1	9.2500	10.8167
Peak 2	11.2500	15.2000

Analyst: .... **Date:** ....

Checked By: .... **Date:** ....

Chromatogram Plot

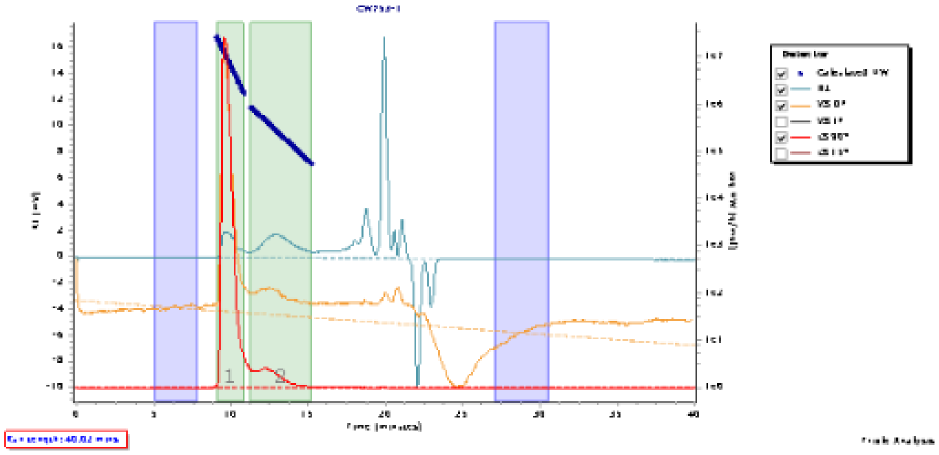

Distribution Plot

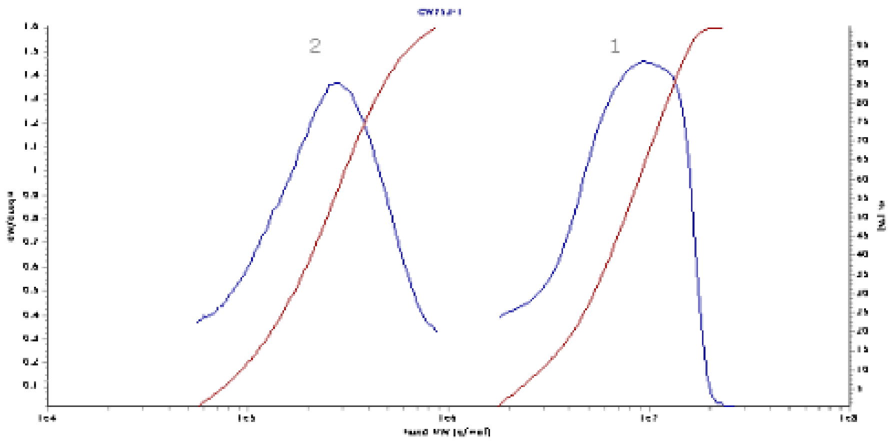

Analyst: .... **Date:** ....

Checked By: .... **Date:** ....

Conformation Plot

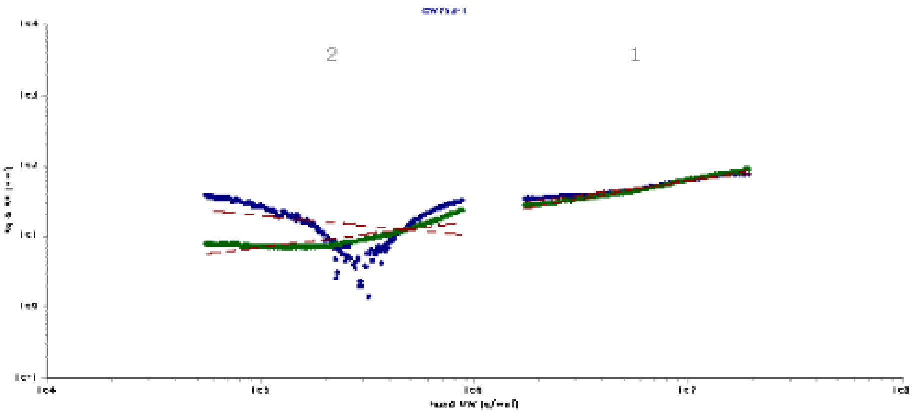

Peak 1 Rg (z3): Log Rg = (0.39) x Log MW + (-0.51), Log Rh = (0.51) x Log MW + (-1.30)
Peak 2 Rg (z3): Log Rg = (-0.26) x Log MW + (2.74), Log Rh = (0.47) x Log MW + (-0.89)

Mark-Houwink Plot

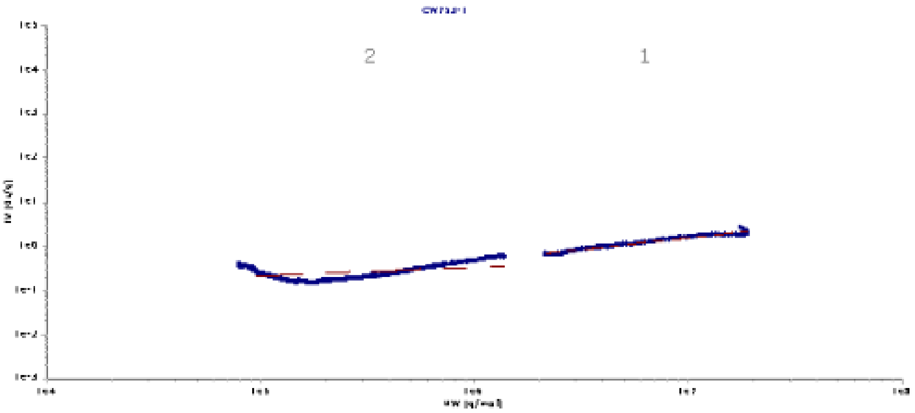

Peak 1: K = ((10e-5) dL/g) 28.64, Alpha = 0.52
Peak 2: K = ((10e-5) dL/g) 2238.27, Alpha = 0.28

Analyst: .... **Date:** ....

Checked By: .... **Date:** ....

Entries in Table 1

HBpin (1 equivalent relative to phenylallene)

Agilent GPC/SEC Software
Sample Triple Analysis Report

Agilent Technologies

cw675

**Workspace Details**	
Workspace name	Polylactide
Location	C:\ProgramData\Agilent Technologies\GPC\Workspaces\Polylactide\
Comments	
Created by	Administrator at 13:44:31 on 15 June 2015
**Sample Properties**	
Sample name	cw675
File name	ICF_21_09_2020-0009.sample
Collected by	GPC at 10:38:40 on 22 September 2020
Instrument name	Instrument 1
**System Calibration Used**	
Created by	Administrator at 13:44:30 on 15 June 2015
Last modified by	Administrator at 13:44:30 on 15 June 2015
Comments	
Sample file used for calibration	ICF_6_18_2015-0002.sample
K (RI)	(RI) 750.958
K (LS 90°)	(RI) 30220.130
K (LS 15°)	(RI) 9870.135
K (VS DP)	(RI) 1.107
**System IDD Used**	
Last modified by	Administrator at 15:52:14 on 18 June 2015
Comments	
Reference detector	LS 90°
IDD Light scatterer (secs)	0.0
IDD RI (secs)	-3.0
IDD Viscometer (secs)	-14.0

Analyst: .... **Date:** ....

Checked By: .... **Date:** ....

Agilent GPC/SEC Software A.02.01 [9] Page 1 of 5

Generated by GPC at 10:27 on 07 October 2020

Agilent GPC/SEC Software
Sample Triple Analysis Report

Agilent Technologies

Processing Parameters

Method	Last modified by Administrator at 13:44:30 on 15 June 2015
Concentration Detector Used in Analysis	RI
Injection volume (µL)	100.00
Flow rate (mL/min)	1.00
Concentration options	Calculate Sample Properties from Entered Sample Concentration
Entered sample concentration (mg/mL)	1.000
Calculated dn/dc (mL/g)	0.304
Calculated Ext Coeff ([(mg/mL)-¹] [cm-¹])	0.000
MW calculation method	Use all angles
Log Mi-v-RT curve fit options	Set the fit limits using the limits at peak width of 10 %.
Polynomial curve fit order	1
Use Constant Inlet Pressure	No
Flory-Fox	2.86e+021
DP Multiplier (mV to Pa)	1.0000
IP Multiplier (mV to kPa)	0.1000
Use IV To Calculate Rg	No

MW Ranges Method

Calculate MW Ranges	No

Percentage Fractions Method

Calculate Percentage Fractions	No

Results

Analysed by	GPC at 11:27:40 on 22 September 2020
Comments	

Peak Results

	Detector Type	Peak Max RT (mins)	Bulk MW (g/mol)	Bulk IV (dL/g)	Peak Height (mV)	Peak Height (%)	Peak Area (mV.s)	Area (%)	Conc. (mg/mL)
Peak 1	RI	9.96667	1106072	2.981197	9.732	64.37	641.136	46.83	0.468
Peak 2	RI	12.90000	73987	0.429858	5.387	35.63	728.048	53.17	0.532
Recovery (%)	100.00								

Molecular Weight Averages

Peak	Mp (g/mol)	Mn (g/mol)	Mw (g/mol)	Mz (g/mol)	Mz+1 (g/mol)	Mv (g/mol)	PD
Peak 1	1452019	765731	1121399	1435988	1664640	1393594	1.464
Peak 2	82413	62517	73824	85209	95060	83546	1.181

Rg Results

Peak	Slope	Intercept	Rgp (nm)	Rgn (nm)	Rgw (nm)	Rgz (nm)	Rgz+1 (nm)
Peak 1	0.4218	-0.9369	45.96	35.01	41.18	45.74	48.71
Peak 2	-0.2556	2.609	22.43	24.10	23.08	22.24	21.62

Analyst: .... **Date:** ....

Checked By: .... **Date:** ....

Agilent GPC/SEC Software
Sample Triple Analysis Report

Agilent Technologies

Rh Results

Peak	Slope	Intercept	Rhp (nm)	Rhn (nm)	Rhw (nm)	Rhz (nm)	Rhz+1 (nm)
Peak 1	0.5591	-1.802	43.92	30.70	38.01	43.65	47.41
Peak 2	0.5607	-1.826	8.50	7.29	8.00	8.66	9.21

IV Results

Peak	K ((10e-5) dL/g)	Alpha	IVp (dL/g)	IVn (dL/g)	IVw (dL/g)	IVz (dL/g)	IVz+1 (dL/g)
Peak 1	27.96	0.6681	3.662008	2.388050	3.081377	3.634945	4.012107
Peak 2	20.54	0.6838	0.472357	0.391032	0.438112	0.483257	0.520799

Peak Information

	Start (mins)	End (mins)
Baseline region 1	2.20000	4.51667
Baseline region 2	32.15000	34.30000
Peak 1	9.36667	11.55000
Peak 2	11.96667	14.78333

Peak Trace Information

Peak	Trace	Peak Max RT (mins)	Peak Area (mV.s)	Peak Height (mV)
Peak 1	RI	9.96667	641.136	9.732
Peak 1	VS DP	9.91667	8009.829	150.719
Peak 1	VS IP	9.86667	239.511	5.113
Peak 1	LS 90°	9.90000	31398.420	626.026
Peak 1	LS 15°	9.85000	13767.529	283.120
Peak 2	RI	12.90000	728.048	5.387
Peak 2	VS DP	11.98333	1307.078	12.600
Peak 2	VS IP	12.31667	8.982	0.353
Peak 2	LS 90°	11.98333	3025.028	36.734
Peak 2	LS 15°	11.98333	1055.992	12.915

Log Mi Curves

Peak	Start (mins)	End (mins)	Polynomial Degree	Coeff a	Coeff b	Coeff c	Coeff d	Coeff e	Coeff f	L Point Y/N
Peak 1	9.4333	11.5333	1	11.190413	-0.50452585	0	0	0	0	N
Peak 2	11.9667	14.7667	1	7.9647161	-0.23633481	0	0	0	0	N

Structural Plot Region

Peak	Start (mins)	End (mins)
Peak 1	9.4333	11.5333
Peak 2	11.9667	14.7667

Analyst: .... **Date:** ....

Checked By: .... **Date:** ....

Agilent GPC/SEC Software
Sample Triple Analysis Report

Agilent Technologies

Chromatogram Plot

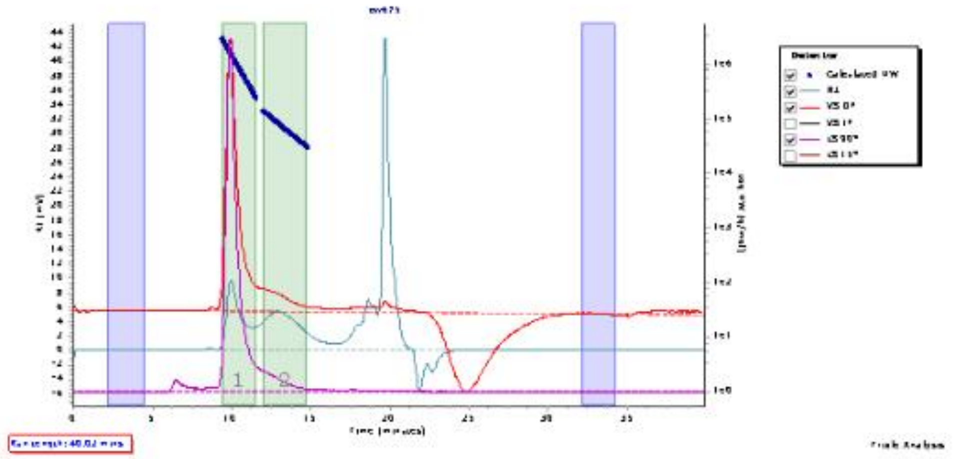

Distribution Plot

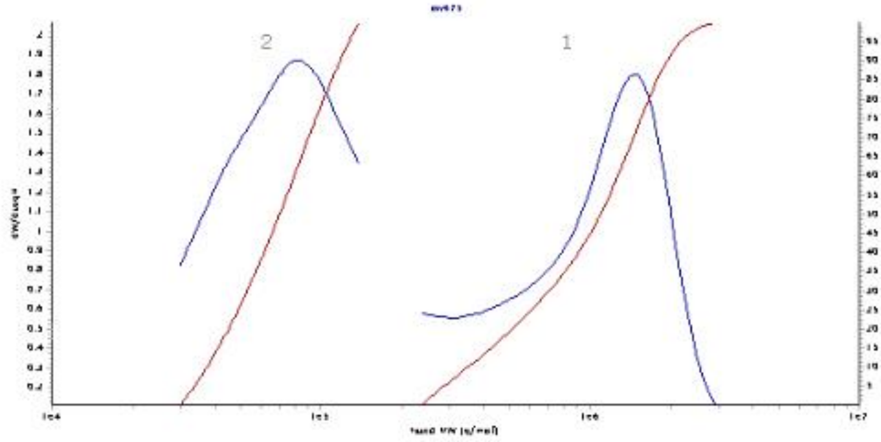

**Analyst:**	....	**Date:**	....
**Checked By:**	....	**Date:**	....

Agilent GPC/SEC Software
Sample Triple Analysis Report

Agilent Technologies

Conformation Plot

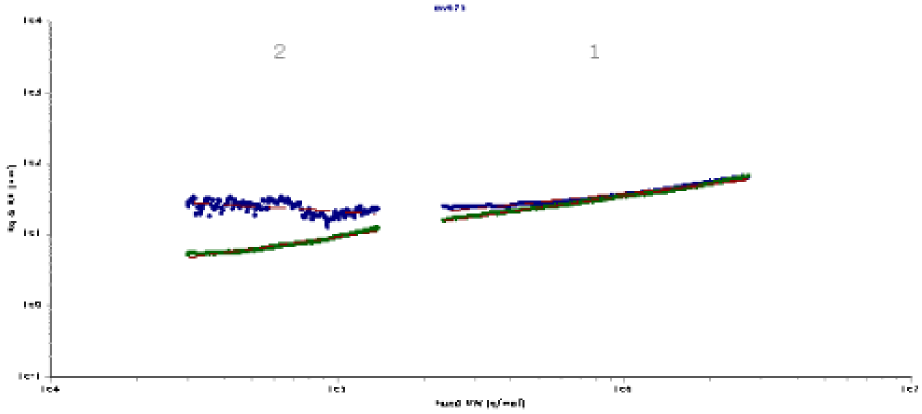

Peak 1 Rg (z5): log Rg = (0.42) x log MW + (-0.54), log Rh = (0.36) x log MW + (-1.20)
Peak 2 Rg (z5): log Rg = (-0.26) x log MW + ([illegible]), log Rh = (0.36) x log MW + ([illegible])

Mark-Houwink Plot

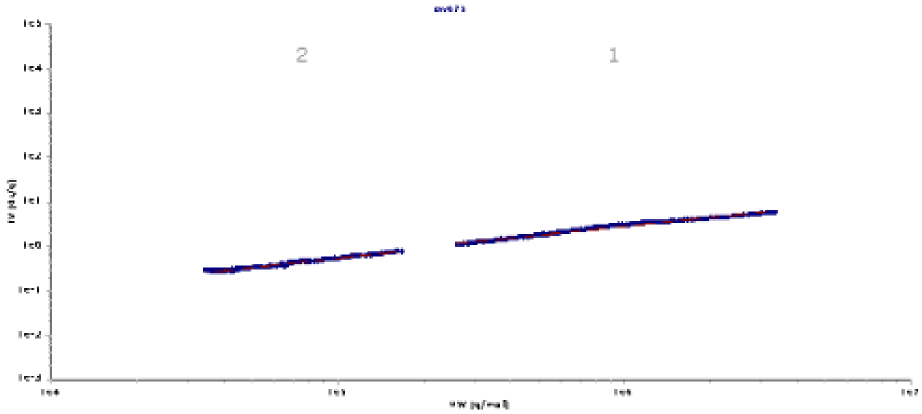

Peak 1: K = ((10e-5) dL/g) 27.86, Alpha = 0.67
Peak 2: K = ((10e-5) dL/g) 28.34, Alpha = 0.68

Analyst: .... **Date:** ....

Checked By: .... **Date:** ....

Agilent GPC/SEC Software A.02.01 [9]   Page 5 of 5

Generated by GPC at 10:27 on 07 October 2020

HBpin (5 mol%)

Agilent GPC/SEC Software
Sample Triple Analysis Report

Agilent Technologies

cw721c

**Workspace Details**	
Workspace name	Polylactide
Location	C:\ProgramData\Agilent Technologies\GPC\Workspaces\Polylactide\
Comments	
Created by	Administrator at 13:44:31 on 15 June 2015
**Sample Properties**	
Sample name	cw721c
File name	ICF_07_12_2020-0005.sample
Collected by	GPC at 13:17:08 on 07 December 2020
Instrument name	Instrument 1
**System Calibration Used**	
Created by	Administrator at 13:44:30 on 15 June 2015
Last modified by	Administrator at 13:44:30 on 15 June 2015
Comments	
Sample file used for calibration	ICF_6_18_2015-0002.sample
K (RI)	(RI) 750.958
K (LS 90°)	(RI) 30220.130
K (LS 15°)	(RI) 9870.135
K (VS DP)	(RI) 1.107
**System IDD Used**	
Last modified by	Administrator at 15:52:14 on 18 June 2015
Comments	
Reference detector	LS 90°
IDD Light scatterer (secs)	0.0
IDD RI (secs)	-3.0
IDD Viscometer (secs)	-14.0

Analyst: .... **Date:** ....

Checked By: .... **Date:** ....

Agilent GPC/SEC Software
Sample Triple Analysis Report

Agilent Technologies

Processing Parameters

Method	Last modified by Administrator at 13:44:30 on 15 June 2015
Concentration Detector Used in Analysis	RI
Injection volume (µL)	100.00
Flow rate (mL/min)	1.00
Concentration options	Calculate Sample Properties from Entered Sample Concentration
Entered sample concentration (mg/mL)	1.000
Calculated dn/dc (mL/g)	0.086
Calculated Ext Coeff ([(mg/mL)-¹] [cm-¹])	0.000
MW calculation method	Use all angles
Log Mi-v-RT curve fit options	Set the fit limits using the limits at peak width of 10 %.
Polynomial curve fit order	1
Use Constant Inlet Pressure	No
Flory-Fox	2.86e+021
DP Multiplier (mV to Pa)	1.0000
IP Multiplier (mV to kPa)	0.1000
Use IV To Calculate Rg	No

MW Ranges Method

Calculate MW Ranges	No

Percentage Fractions Method

Calculate Percentage Fractions	No

Results

Analysed by	GPC at 16:30:07 on 08 December 2020
Comments	

Peak Results

	Detector Type	Peak Max RT (mins)	Bulk MW (g/mol)	Bulk IV (dL/g)	Peak Height (mV)	Peak Height (%)	Peak Area (mV.s)	Area (%)	Conc. (mg/mL)
Peak 1	RI	10.03333	3525321	0.735411	0.765	34.19	38.109	9.86	0.099
Peak 2	RI	12.90000	205411	0.084365	1.472	65.81	348.307	90.14	0.901
Recovery (%)	100.00								

Molecular Weight Averages

Peak	Mp (g/mol)	Mn (g/mol)	Mw (g/mol)	Mz (g/mol)	Mz+1 (g/mol)	Mv (g/mol)	PD
Peak 1	4251277	3000110	3582928	4154862	4698846	4067441	1.194
Peak 2	271946	152438	207511	262433	306271	262933	1.361

Rg Results

Peak	Slope	Intercept	Rgp (nm)	Rgn (nm)	Rgw (nm)	Rgz (nm)	Rgz+1 (nm)
Peak 1	0.4832	-1.578	42.21	35.58	38.82	41.74	44.33
Peak 2	-0.8472	6.065	28.89	47.22	36.34	29.78	26.12

Analyst: .... **Date:** ....

Checked By: .... **Date:** ....

Rh Results

Peak	Slope	Intercept	Rhp (nm)	Rhn (nm)	Rhw (nm)	Rhz (nm)	Rhz+1 (nm)
Peak 1	0.5631	-2.149	38.28	31.45	34.76	37.79	40.51
Peak 2	0.7656	-3.262	7.93	5.09	6.44	7.72	8.69

IV Results

Peak	K ((10e-5) dL/g)	Alpha	IVp (dL/g)	IVn (dL/g)	IVw (dL/g)	IVz (dL/g)	IVz+1 (dL/g)
Peak 1	2.694	0.6771	0.828629	0.654430	0.738017	0.815858	0.886735
Peak 2	0.02848	1.022	0.101447	0.056160	0.076959	0.097823	0.114545

Peak Information

	Start (mins)	End (mins)
Baseline region 1	5.58333	7.40000
Baseline region 2	33.38333	36.56667
Peak 1	8.98333	11.11667
Peak 2	11.18333	17.33333

Peak Trace Information

Peak	Trace	Peak Max RT (mins)	Peak Area (mV.s)	Peak Height (mV)
Peak 1	RI	10.03333	38.109	0.765
Peak 1	VS DP	10.03333	424.201	9.392
Peak 1	VS IP	9.00000	3.616	-0.678
Peak 1	LS 90°	10.01667	1782.570	41.982
Peak 1	LS 15°	9.98333	736.084	17.404
Peak 2	RI	12.90000	348.307	1.472
Peak 2	VS DP	12.36667	444.750	2.853
Peak 2	VS IP	16.80000	55.431	0.460
Peak 2	LS 90°	12.06667	998.454	7.323
Peak 2	LS 15°	12.08333	393.899	2.483

Log Mi Curves

Peak	Start (mins)	End (mins)	Polynomial Degree	Coeff a	Coeff b	Coeff c	Coeff d	Coeff e	Coeff f	L Point Y/N
Peak 1	9.6000	11.1000	1	11.388167	-0.47438349	0	0	0	0	N
Peak 2	11.1833	17.3167	1	7.4456492	-0.15590435	0	0	0	0	N

Structural Plot Region

Peak	Start (mins)	End (mins)
Peak 1	9.6000	11.1000
Peak 2	11.1833	17.3167

Analyst: .... **Date:** ....

Checked By: .... **Date:** ....

Agilent GPC/SEC Software
Sample Triple Analysis Report

Agilent Technologies

Chromatogram Plot

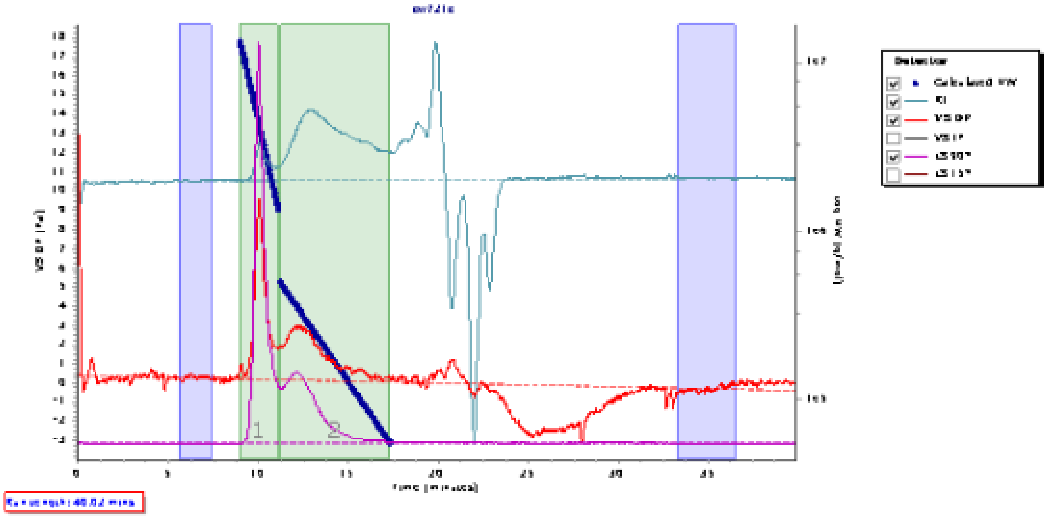

Distribution Plot

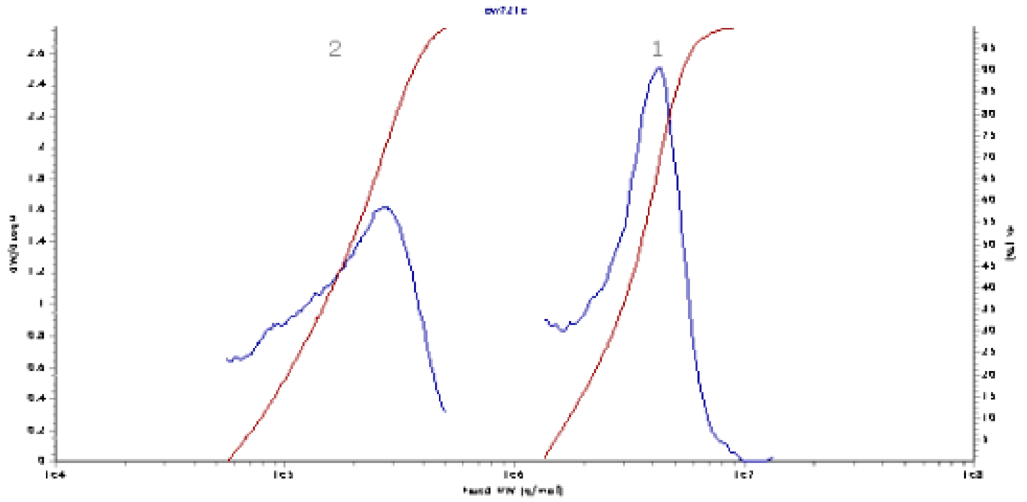

Analyst: .... **Date:** ....

Checked By: .... **Date:** ....

Agilent GPC/SEC Software
Sample Triple Analysis Report

Conformation Plot

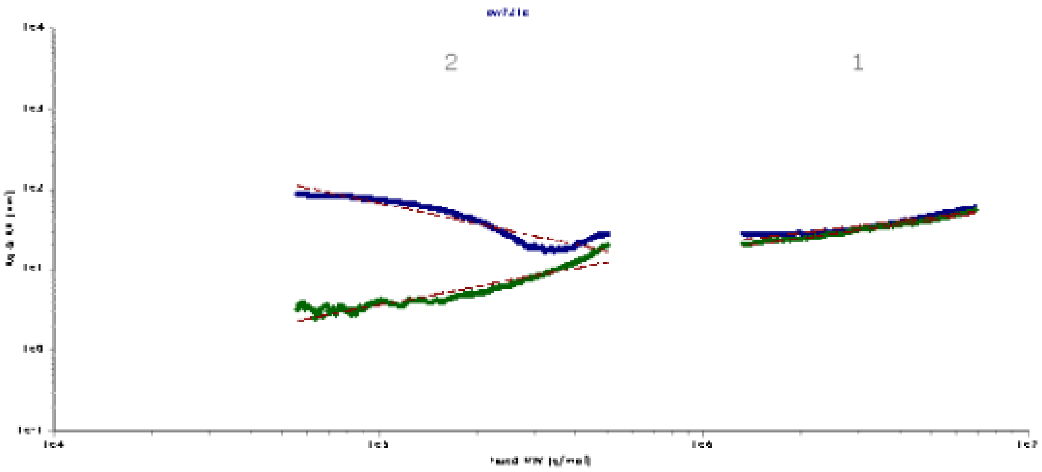

Peak 1 Rg (z3): Log Rg = (0.48) x Log MW + (-1.58), Log Rh = (0.56) x Log MW + (-2.15)
Peak 2 Rg (z3): Log Rg = (-0.25) x Log MW + ([illegible]), Log Rh = (0.77) x Log MW + (-3.26)

Mark-Houwink Plot

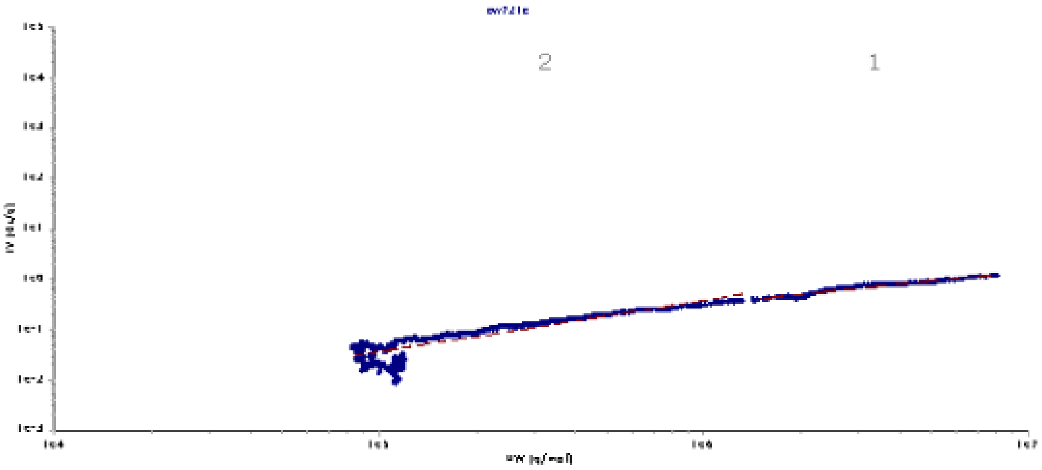

Peak 1 : K = ((10e-5) dL/g) 2.69, Alpha = 0.68
Peak 2 : K = ((10e-5) dL/g) [illegible], Alpha = [illegible]

Analyst: .... **Date:** ....

Checked By: .... **Date:** ....

$NH_3 \cdot BH_3$ (5 mol%)

As optimised conditions, see data for P1

$Me_2NH \cdot BH_3$ (5 mol%)

Agilent GPC/SEC Software
Sample Triple Analysis Report

Agilent Technologies

cw721b

**Workspace Details**	
Workspace name	Polylactide
Location	C:\ProgramData\Agilent Technologies\GPC\Workspaces\Polylactide\
Comments	
Created by	Administrator at 13:44:31 on 15 June 2015
**Sample Properties**	
Sample name	cw721b
File name	ICF_07_12_2020-0004.sample
Collected by	GPC at 12:35:17 on 07 December 2020
Instrument name	Instrument 1
**System Calibration Used**	
Created by	Administrator at 13:44:30 on 15 June 2015
Last modified by	Administrator at 13:44:30 on 15 June 2015
Comments	
Sample file used for calibration	ICF_6_18_2015-0002.sample
K (RI)	(RI) 750.958
K (LS 90°)	(RI) 30220.130
K (LS 15°)	(RI) 9870.135
K (VS DP)	(RI) 1.107
**System IDD Used**	
Last modified by	Administrator at 15:52:14 on 18 June 2015
Comments	
Reference detector	LS 90°
IDD Light scatterer (secs)	0.0
IDD RI (secs)	-3.0
IDD Viscometer (secs)	-14.0

Analyst: .... **Date:** ....

Checked By: .... **Date:** ....

Agilent GPC/SEC Software A.02.01 [9] Page 1 of 5

Generated by GPC at 16:32 on 08 December 2020

Agilent GPC/SEC Software
Sample Triple Analysis Report

Agilent Technologies

Processing Parameters

Method	Last modified by Administrator at 13:44:30 on 15 June 2015
Concentration Detector Used in Analysis	RI
Injection volume (µL)	100.00
Flow rate (mL/min)	1.00
Concentration options	Calculate Sample Properties from Entered Sample Concentration
Entered sample concentration (mg/mL)	1.000
Calculated dn/dc (mL/g)	0.099
Calculated Ext Coeff ([(mg/mL)-¹] [cm-¹])	0.000
MW calculation method	Use all angles
Log Mi-v-RT curve fit options	Set the fit limits using the limits at peak width of 10 %.
Polynomial curve fit order	1
Use Constant Inlet Pressure	No
Flory-Fox	2.86e+021
DP Multiplier (mV to Pa)	1.0000
IP Multiplier (mV to kPa)	0.1000
Use IV To Calculate Rg	No

MW Ranges Method

Calculate MW Ranges — No

Percentage Fractions Method

Calculate Percentage Fractions — No

Results

Analysed by — GPC at 16:28:30 on 08 December 2020

Comments

Peak Results

	Detector Type	Peak Max RT (mins)	Bulk MW (g/mol)	Bulk IV (dL/g)	Peak Height (mV)	Peak Height (%)	Peak Area (mV.s)	Area (%)	Conc. (mg/mL)
Peak 1	RI	14.26667	62362	0.099361	2.445	100.00	448.014	100.00	1.000
Recovery (%)	100.00								

Molecular Weight Averages

Peak	Mp (g/mol)	Mn (g/mol)	Mw (g/mol)	Mz (g/mol)	Mz+1 (g/mol)	Mv (g/mol)	PD
Peak 1	26947	10525	65785	300179	627498	216457	6.25

Rg Results

Peak	Slope	Intercept	Rgp (nm)	Rgn (nm)	Rgw (nm)	Rgz (nm)	Rgz+1 (nm)
Peak 1	-7.227E-16	1.967	92.64	92.64	92.64	92.64	92.64

Rh Results

Peak	Slope	Intercept	Rhp (nm)	Rhn (nm)	Rhw (nm)	Rhz (nm)	Rhz+1 (nm)
Peak 1	0.5116	-1.765	3.18	1.96	5.02	10.90	15.90

Analyst: .... **Date:** ....

Checked By: .... **Date:** ....

Agilent GPC/SEC Software A.02.01 [9] Page 2 of 5

Generated by GPC at 16:32 on 08 December 2020

Agilent GPC/SEC Software
Sample Triple Analysis Report

Agilent Technologies

IV Results

Peak	K ((10e-5) dL/g)	Alpha	IVp (dL/g)	IVn (dL/g)	IVw (dL/g)	IVz (dL/g)	IVz+1 (dL/g)
Peak 1	120	0.4129	0.081006	0.054945	0.117106	0.219184	0.297196

Peak Information

	Start (mins)	End (mins)
Baseline region 1	7.03333	8.48333
Baseline region 2	31.45000	34.03333
Peak 1	11.03333	16.66667

Peak Trace Information

Peak	Trace	Peak Max RT (mins)	Peak Area (mV.s)	Peak Height (mV)
Peak 1	RI	14.26667	448.014	2.445
Peak 1	VS DP	13.45000	580.862	2.731
Peak 1	VS IP	16.66667	50.898	-0.505
Peak 1	LS 90°	13.01667	732.798	4.092
Peak 1	LS 15°	12.93333	181.195	1.122

Log Mi Curves

Peak	Start (mins)	End (mins)	Polynomial Degree	Coeff a	Coeff b	Coeff c	Coeff d	Coeff e	Coeff f	L Point Y/N
Peak 1	11.7500	16.6500	1	11.85445	-0.52097874	0	0	0	0	N

Structural Plot Region

Peak	Start (mins)	End (mins)
Peak 1	11.7500	16.6500

Analyst: .... **Date:** ....

Checked By: .... **Date:** ....

Agilent GPC/SEC Software A.02.01 [9]   Page 3 of 5

Generated by GPC at 16:32 on 08 December 2020

Agilent GPC/SEC Software
Sample Triple Analysis Report

Agilent Technologies

Chromatogram Plot

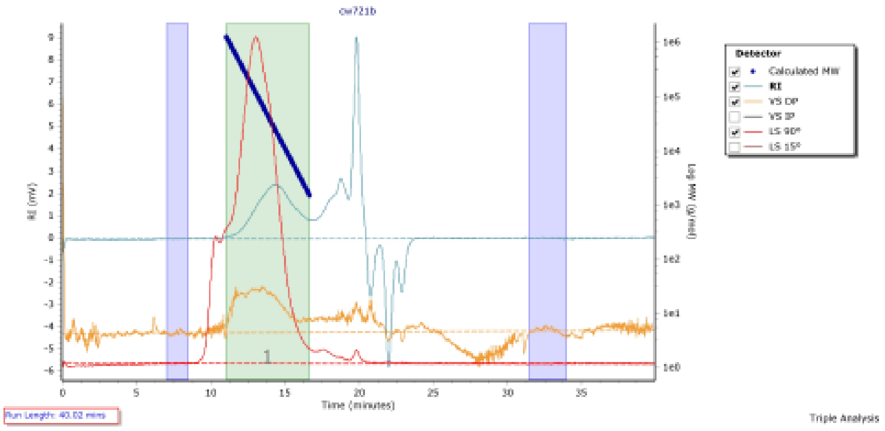

Distribution Plot

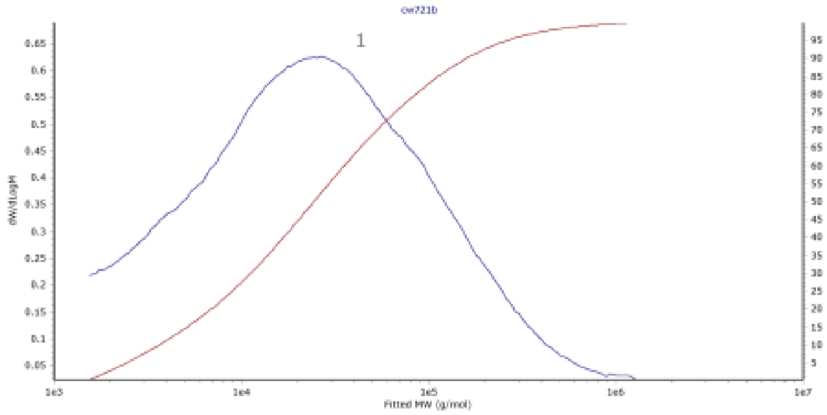

Analyst: .... **Date:** ....

Checked By: .... **Date:** ....

Agilent GPC/SEC Software A.02.01 [9]   Page 4 of 5

Generated by GPC at 16:32 on 08 December 2020

Agilent GPC/SEC Software
Sample Triple Analysis Report

Agilent Technologies

Conformation Plot

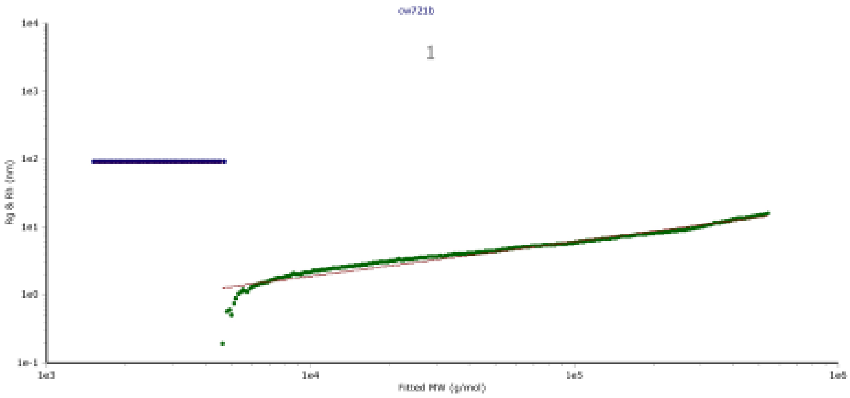

Log Rg = (0.00) x Log MW + (1.97), Log Rh = (0.51) x Log MW + (-1.76)

Mark-Houwink Plot

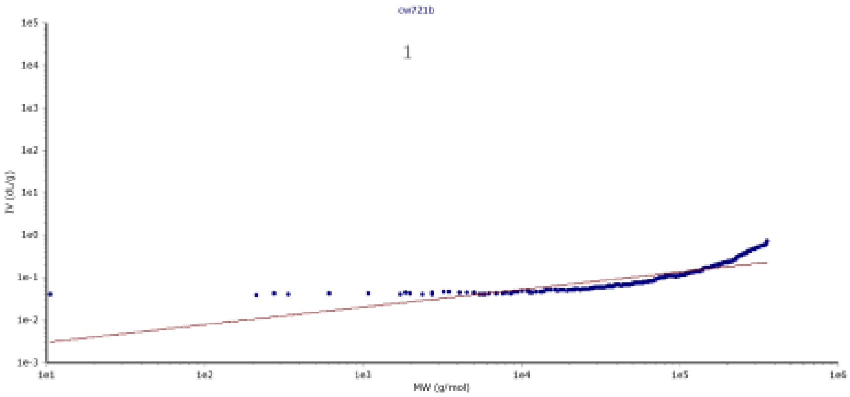

K = ((10e-5) dL/g) 119.95, Alpha = 0.41

Analyst: .... **Date:** ....

Checked By: .... **Date:** ....

Agilent GPC/SEC Software A.02.01 [9] Page 5 of 5

Generated by GPC at 16:32 on 08 December 2020

$HSi(OEt)_3$ (5 mol%)

Agilent GPC/SEC Software
Sample Triple Analysis Report

Agilent Technologies

cw675

**Workspace Details**	
Workspace name	Polylactide
Location	C:\ProgramData\Agilent Technologies\GPC\Workspaces\Polylactide\
Comments	
Created by	Administrator at 13:44:31 on 15 June 2015
**Sample Properties**	
Sample name	cw675
File name	ICF_21_09_2020-0009.sample
Collected by	GPC at 10:38:40 on 22 September 2020
Instrument name	Instrument 1
**System Calibration Used**	
Created by	Administrator at 13:44:30 on 15 June 2015
Last modified by	Administrator at 13:44:30 on 15 June 2015
Comments	
Sample file used for calibration	ICF_6_18_2015-0002.sample
K (RI)	(RI) 750.958
K (LS 90°)	(RI) 30220.130
K (LS 15°)	(RI) 9870.135
K (VS DP)	(RI) 1.107
**System IDD Used**	
Last modified by	Administrator at 15:52:14 on 18 June 2015
Comments	
Reference detector	LS 90°
IDD Light scatterer (secs)	0.0
IDD RI (secs)	-3.0
IDD Viscometer (secs)	-14.0

Analyst: .... **Date:** ....

Checked By: .... **Date:** ....

Agilent GPC/SEC Software
Sample Triple Analysis Report

Agilent Technologies

Processing Parameters

Method	Last modified by Administrator at 13:44:30 on 15 June 2015
Concentration Detector Used in Analysis	RI
Injection volume (µL)	100.00
Flow rate (mL/min)	1.00
Concentration options	Calculate Sample Properties from Entered Sample Concentration
Entered sample concentration (mg/mL)	1.000
Calculated dn/dc (mL/g)	0.304
Calculated Ext Coeff ([(mg/mL)-¹] [cm-¹])	0.000
MW calculation method	Use all angles
Log Mi-v-RT curve fit options	Set the fit limits using the limits at peak width of 10 %.
Polynomial curve fit order	1
Use Constant Inlet Pressure	No
Flory-Fox	2.86e+021
DP Multiplier (mV to Pa)	1.0000
IP Multiplier (mV to kPa)	0.1000
Use IV To Calculate Rg	No

MW Ranges Method

Calculate MW Ranges — No

Percentage Fractions Method

Calculate Percentage Fractions — No

Results

Analysed by — GPC at 11:27:40 on 22 September 2020
Comments

Peak Results

	Detector Type	Peak Max RT (mins)	Bulk MW (g/mol)	Bulk IV (dL/g)	Peak Height (mV)	Peak Height (%)	Peak Area (mV.s)	Area (%)	Conc. (mg/mL)
Peak 1	RI	9.96667	1106072	2.981197	9.732	64.37	641.136	46.83	0.468
Peak 2	RI	12.90000	73987	0.429858	5.367	35.63	728.048	53.17	0.532
Recovery (%)	100.00								

Molecular Weight Averages

Peak	Mp (g/mol)	Mn (g/mol)	Mw (g/mol)	Mz (g/mol)	Mz+1 (g/mol)	Mv (g/mol)	PD
Peak 1	1452019	765731	1121399	1435988	1664640	1393594	1.464
Peak 2	82413	62517	73824	85209	95060	83546	1.181

Rg Results

Peak	Slope	Intercept	Rgp (nm)	Rgn (nm)	Rgw (nm)	Rgz (nm)	Rgz+1 (nm)
Peak 1	0.4218	-0.9369	45.96	35.01	41.18	45.74	48.71
Peak 2	-0.2556	2.609	22.43	24.10	23.08	22.24	21.62

Analyst: .... **Date:** ....

Checked By: .... **Date:** ....

Agilent GPC/SEC Software
Sample Triple Analysis Report

Agilent Technologies

Rh Results

Peak	Slope	Intercept	Rhp (nm)	Rhn (nm)	Rhw (nm)	Rhz (nm)	Rhz+1 (nm)
Peak 1	0.5591	-1.802	43.92	30.70	38.01	43.65	47.41
Peak 2	0.5607	-1.826	8.50	7.29	8.00	8.66	9.21

IV Results

Peak	K ((10e-5) dL/g)	Alpha	IVp (dL/g)	IVn (dL/g)	IVw (dL/g)	IVz (dL/g)	IVz+1 (dL/g)
Peak 1	27.96	0.6681	3.662008	2.388050	3.081377	3.634945	4.012107
Peak 2	20.54	0.6838	0.472357	0.391032	0.438112	0.483257	0.520799

Peak Information

	Start (mins)	End (mins)
Baseline region 1	2.20000	4.51667
Baseline region 2	32.15000	34.30000
Peak 1	9.36667	11.55000
Peak 2	11.96667	14.78333

Peak Trace Information

Peak	Trace	Peak Max RT (mins)	Peak Area (mV.s)	Peak Height (mV)
Peak 1	RI	9.96667	641.136	9.732
Peak 1	VS DP	9.91667	8009.829	150.719
Peak 1	VS IP	9.86667	239.511	5.113
Peak 1	LS 90°	9.90000	31398.420	626.026
Peak 1	LS 15°	9.85000	13767.529	283.120
Peak 2	RI	12.90000	728.048	5.387
Peak 2	VS DP	11.98333	1307.078	12.600
Peak 2	VS IP	12.31667	8.982	0.353
Peak 2	LS 90°	11.98333	3025.028	36.734
Peak 2	LS 15°	11.98333	1055.992	12.915

Log Mi Curves

Peak	Start (mins)	End (mins)	Polynomial Degree	Coeff a	Coeff b	Coeff c	Coeff d	Coeff e	Coeff f	L Point Y/N
Peak 1	9.4333	11.5333	1	11.190413	-0.50452585	0	0	0	0	N
Peak 2	11.9667	14.7667	1	7.9647161	-0.23633481	0	0	0	0	N

Structural Plot Region

Peak	Start (mins)	End (mins)
Peak 1	9.4333	11.5333
Peak 2	11.9667	14.7667

Analyst: .... **Date:** ....

Checked By: .... **Date:** ....

Agilent GPC/SEC Software
Sample Triple Analysis Report

Agilent Technologies

Chromatogram Plot

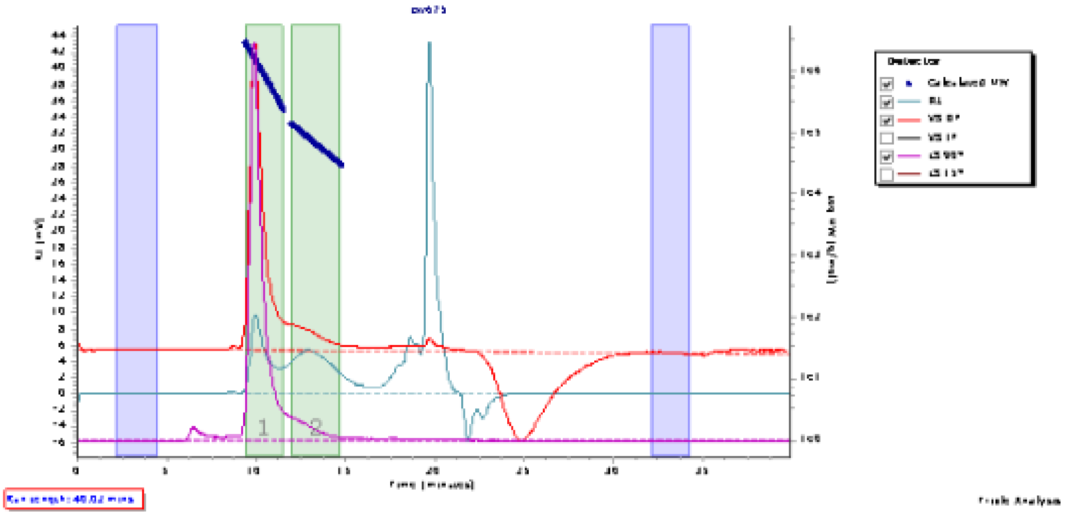

Distribution Plot

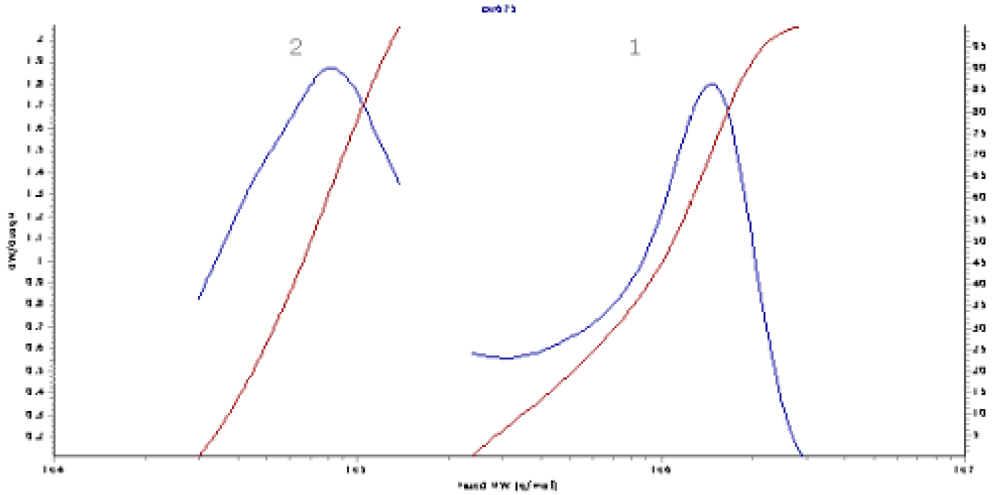

Analyst: .... **Date:** ....

Checked By: .... **Date:** ....

Conformation Plot

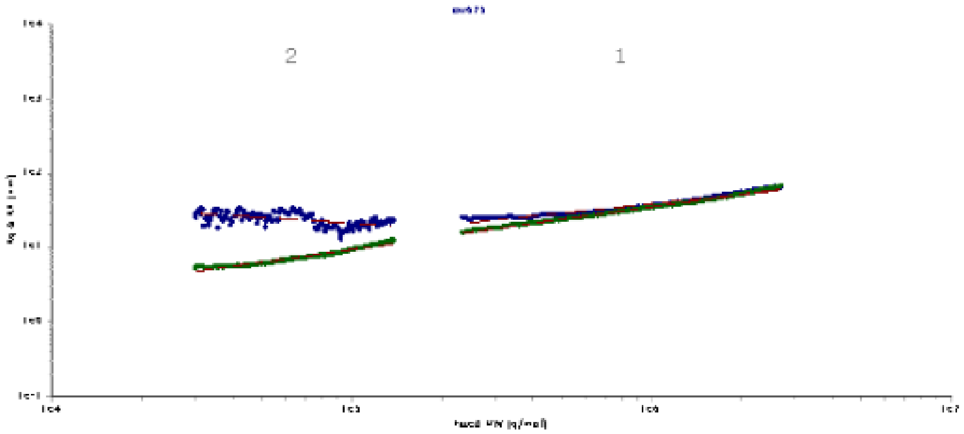

[illegible]
[illegible]

Mark-Houwink Plot

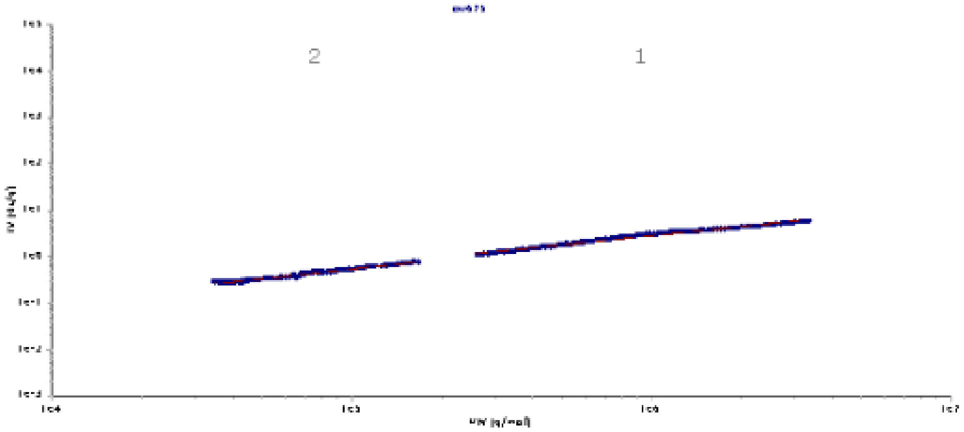

[illegible]
[illegible]

Analyst: .... **Date:** ....

Checked By: .... **Date:** ....

Entries in Table 2

Stirring

Agilent GPC/SEC Software
Sample GPC Analysis Report

Agilent Technologies

cw729e

Workspace Details

Workspace name	Polylactide
Location	C:\ProgramData\Agilent Technologies\GPC\Workspaces\Polylactide\
Comments	
Created by	Administrator at 13:44:31 on 15 June 2015

Sample Properties

Sample name	cw729e
File name	ICF_15_01_2021-0002.sample
Collected by	GPC at 12:38:44 on 15 January 2021
Instrument name	Instrument 1

Column Calibration Details

Name	PSty Jan2021
Created by	GPC at 15:02:17 on 11 January 2021
Last modified by	GPC at 15:02:55 on 11 January 2021
Comments	GPC Column Calibration created 11 January 2021 by GPC<br>GPC Column Calibration amended 11 January 2021 by GPC<br>GPC Column Calibration amended 11 January 2021 by GPC<br>GPC Column Calibration amended 11 January 2021 by GPC

Calibration Type	Narrow Standard	Curve Fit Used	3
Calibration Curve	y = - 0.002078x^3 + 0.09161x^2 - 1.726x + 16.29		
High Limit MW RT (mins)	11.35000	Low Limit MW RT (mins)	18.03333
High Limit MW (g/mol)	299400	Low Limit MW (g/mol)	580
Flow Rate Marker Name		Flow Marker RT (mins)	0.00000
K (Input) ((10e-5) dL/g)	14.100		
Alpha (Input)	0.700		
Residual Sum Of Squares	0.00343472	Corrected Sum Of Squares	7.33781
Coeff. Of Determination	0.999532	Standard Y Error Estimate	0.023926
Linear Correlation Coeff	-0.999617		

Column Calibration Data Points

Point	Peak Max RT (mins)	MW	Log MW	Point in Use?	Percent Error
1	11.35000	299400	5.48	Yes	4.11
2	11.93333	151700	5.18	Yes	-5.03
3	12.78333	66350	4.82	Yes	-6.69
4	13.55000	38100	4.58	Yes	7.47
5	14.23333	19880	4.30	Yes	3.14
6	14.96667	9920	4.00	Yes	-2.12
7	15.78333	4920	3.69	Yes	-0.16
8	16.55000	2360	3.37	Yes	-4.19
9	17.28333	1260	3.10	Yes	2.51
10	18.03333	580	2.76	Yes	0.07

Analyst: .... **Date:** ....

Checked By: .... **Date:** ....

Agilent GPC/SEC Software A.02.01 [9] Page 1 of 4

Generated by GPC at 12:17 on 03 February 2021

Agilent GPC/SEC Software
Sample GPC Analysis Report

Agilent Technologies

Processing Parameters

Method	Last modified by Administrator at 13:44:30 on 15 June 2015
Using Flow Rate Correction	No
Mark-Houwink K ((10e-5) dL/g)	14.100
Mark-Houwink Alpha	0.700
Concentration Detector Used in Analysis	RI
Injection volume (µL)	100.00
Flow rate (mL/min)	1.00

MW Ranges Method

Calculate MW Ranges No

Percentage Fractions Method

Calculate Percentage Fractions No

Results

Analysed by GPC at 12:17:19 on 03 February 2021

Comments

Molecular Weight Averages

Peak	Mp (g/mol)	Mn (g/mol)	Mw (g/mol)	Mz (g/mol)	Mz+1 (g/mol)	Mv (g/mol)	PD
Peak 1	1162965	733963	855135	981221	1092784	963490	1.165
Peak 2	96754	84912	117536	163853	213681	156576	1.384

Peak Information

	Start (mins)	End (mins)
Baseline region 1	3.33333	6.51667
Baseline region 2	29.60000	33.23333
Peak 1	9.56667	11.03333
Peak 2	11.03333	13.50000

Peak Trace Information

Peak	Trace	Peak Max RT (mins)	Peak Area (mV.s)	Peak Height (mV)
Peak 1	RI	10.08333	5.368	0.103
Peak 1	VS DP	10.16667	86.222	1.346
Peak 1	VS IP	10.65000	12.816	-0.607
Peak 1	LS 90°	10.08333	249.824	4.974
Peak 1	LS 15°	10.05000	88.177	1.879
Peak 2	RI	12.45000	46.051	0.441
Peak 2	VS DP	11.40000	128.640	1.457
Peak 2	VS IP	12.86667	18.456	0.561
Peak 2	LS 90°	11.91667	268.938	2.526
Peak 2	LS 15°	11.88333	63.067	0.665

Analyst: .... **Date:** ....

Checked By: .... **Date:** ....

Agilent GPC/SEC Software
Sample GPC Analysis Report

Agilent Technologies

Chromatogram Plot

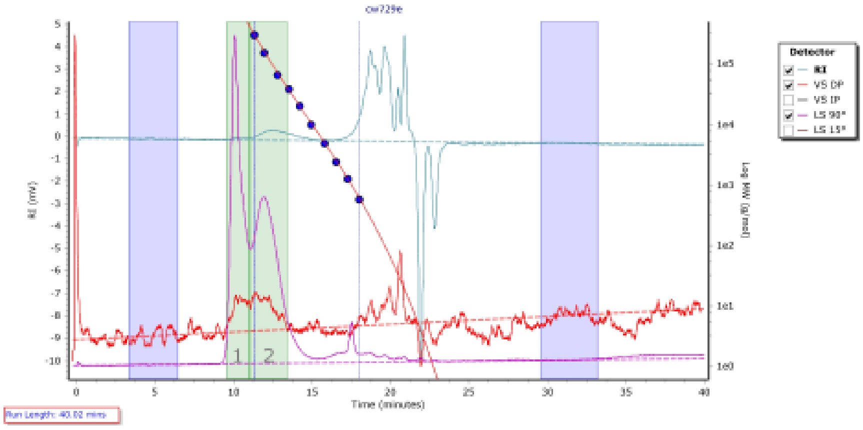

Analyst: .... **Date:** ....

Checked By: .... **Date:** ....

Agilent GPC/SEC Software
Sample GPC Analysis Report

Agilent Technologies

Distribution Plot

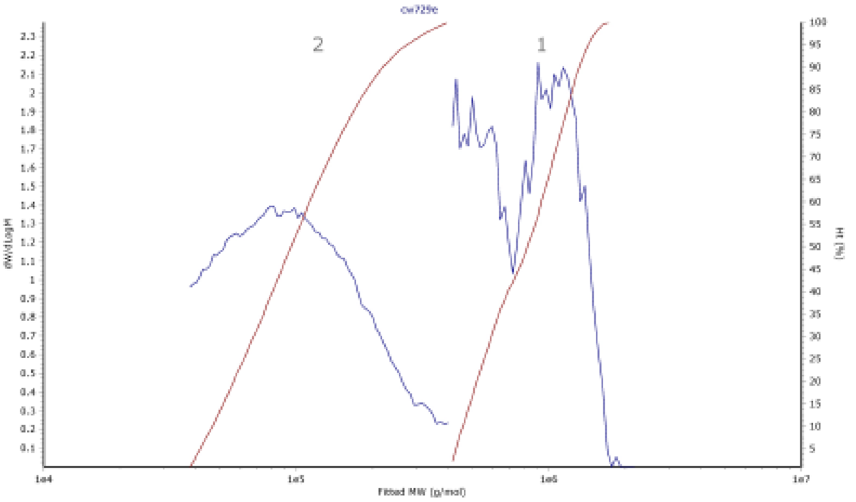

Analyst: .... **Date:** ....

Checked By: .... **Date:** ....

100ul of Solvent

Agilent GPC/SEC Software
Sample Triple Analysis Report

Agilent Technologies

CW431

**Workspace Details**	
Workspace name	Polylactide
Location	C:\ProgramData\Agilent Technologies\GPC\Workspaces\Polylactide\
Comments	
Created by	Administrator at 13:44:31 on 15 June 2015
**Sample Properties**	
Sample name	CW431
File name	ICF_09_07_2019-0002.sample
Collected by	GPC at 10:37:17 on 09 July 2019
Instrument name	Instrument 1
**System Calibration Used**	
Created by	Administrator at 13:44:30 on 15 June 2015
Last modified by	Administrator at 13:44:30 on 15 June 2015
Comments	
Sample file used for calibration	ICF_6_18_2015-0002.sample
K (RI)	(RI) 750.958
K (LS 90°)	(RI) 30220.130
K (LS 15°)	(RI) 9870.135
K (VS DP)	(RI) 1.107
**System IDD Used**	
Last modified by	Administrator at 15:52:14 on 18 June 2015
Comments	
Reference detector	LS 90°
IDD Light scatterer (secs)	0.0
IDD RI (secs)	-3.0
IDD Viscometer (secs)	-14.0

Analyst: .... **Date:** ....

Checked By: .... **Date:** ....

Agilent GPC/SEC Software A.02.01 [9] Page 1 of 5

Generated by GPC at 13:02 on 09 July 2019

Agilent GPC/SEC Software
Sample Triple Analysis Report

Agilent Technologies

Processing Parameters

Method	Last modified by Administrator at 13:44:30 on 15 June 2015
Concentration Detector Used in Analysis	RI
Injection volume (µL)	100.00
Flow rate (mL/min)	1.00
Concentration options	Calculate Sample Properties from Entered Sample Concentration
Entered sample concentration (mg/mL)	1.000
Calculated dn/dc (mL/g)	0.019
Calculated Ext Coeff ([(mg/mL)-¹] [cm-¹])	0.000
MW calculation method	Use 90° angle
Log Mi-v-RT curve fit options	Set the fit limits using the limits at peak width of 10 %.
Polynomial curve fit order	1
Use Constant Inlet Pressure	No
Flory-Fox	2.86e+021
DP Multiplier (mV to Pa)	1.0000
IP Multiplier (mV to kPa)	0.1000
Use IV To Calculate Rg	Yes

MW Ranges Method

Calculate MW Ranges	No

Percentage Fractions Method

Calculate Percentage Fractions	No

Results

Analysed by	GPC at 13:02:00 on 09 July 2019
Comments	

Peak Results

	Detector Type	Peak Max RT (mins)	Bulk MW (g/mol)	Bulk IV (dL/g)	Peak Height (mV)	Peak Height (%)	Peak Area (mV.s)	Area (%)	Conc. (mg/mL)
Peak 1	RI	13.16667	741688	0.043017	0.584	100.00	83.925	100.00	1.000
Recovery (%)	100.00								

Molecular Weight Averages

Peak	Mp (g/mol)	Mn (g/mol)	Mw (g/mol)	Mz (g/mol)	Mz+1 (g/mol)	Mv (g/mol)	PD
Peak 1	710751	480724	731872	1060076	1417600	1007014	1.522

Rg Results

Peak	Slope	Intercept	Rgp (nm)	Rgn (nm)	Rgw (nm)	Rgz (nm)	Rgz+1 (nm)
Peak 1	0.5538	-2.333	8.07	6.49	8.21	10.09	11.86

Rh Results

Peak	Slope	Intercept	Rhp (nm)	Rhn (nm)	Rhw (nm)	Rhz (nm)	Rhz+1 (nm)
Peak 1	0.5538	-2.345	7.86	6.32	7.99	9.82	11.55

Analyst: .... **Date:** ....

Checked By: .... **Date:** ....

Agilent GPC/SEC Software
Sample Triple Analysis Report

Agilent Technologies

IV Results

Peak	K ((10e-5) dL/g)	Alpha	IVp (dL/g)	IVn (dL/g)	IVw (dL/g)	IVz (dL/g)	IVz+1 (dL/g)
Peak 1	0.4343	0.6834	0.043315	0.033158	0.044191	0.056923	0.069429

Peak Information

	Start (mins)	End (mins)
Baseline region 1	4.10000	6.46667
Baseline region 2	32.31667	35.78333
Peak 1	10.61667	15.36667

Peak Trace Information

Peak	Trace	Peak Max RT (mins)	Peak Area (mV.s)	Peak Height (mV)
Peak 1	RI	13.16667	83.925	0.584
Peak 1	VS DP	12.46667	251.027	1.701
Peak 1	VS IP	10.78333	19.082	0.418
Peak 1	LS 90°	12.56667	229.571	1.831
Peak 1	LS 15°	12.56667	68.992	0.571

Log Mi Curves

Peak	Start (mins)	End (mins)	Polynomial Degree	Coeff a	Coeff b	Coeff c	Coeff d	Coeff e	Coeff f	L Point Y/N
Peak 1	11.5000	15.3500	1	10.002037	-0.31561367	0	0	0	0	N

Structural Plot Region

Peak	Start (mins)	End (mins)
Peak 1	11.5000	15.3500

Analyst: .... **Date:** ....

Checked By: .... **Date:** ....

Agilent GPC/SEC Software
Sample Triple Analysis Report

Agilent Technologies

Chromatogram Plot

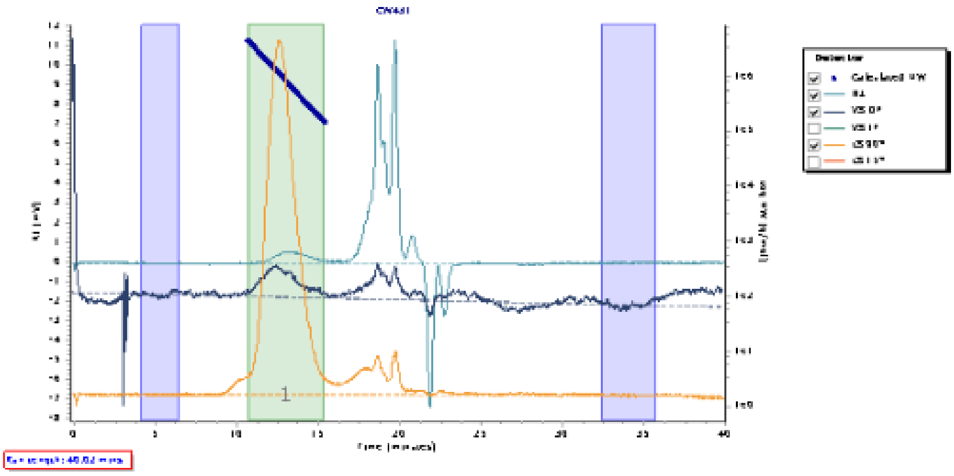

Distribution Plot

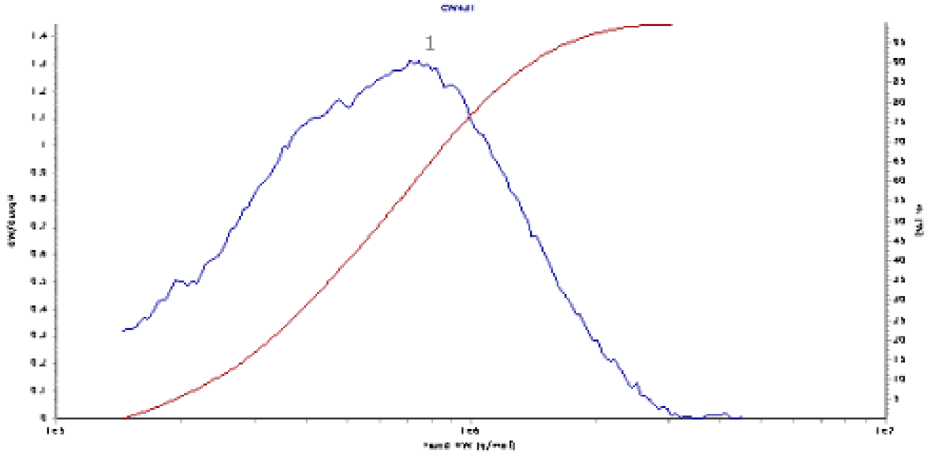

Analyst: .... **Date:** ....

Checked By: .... **Date:** ....

Agilent GPC/SEC Software
Sample Triple Analysis Report

Agilent Technologies

Conformation Plot

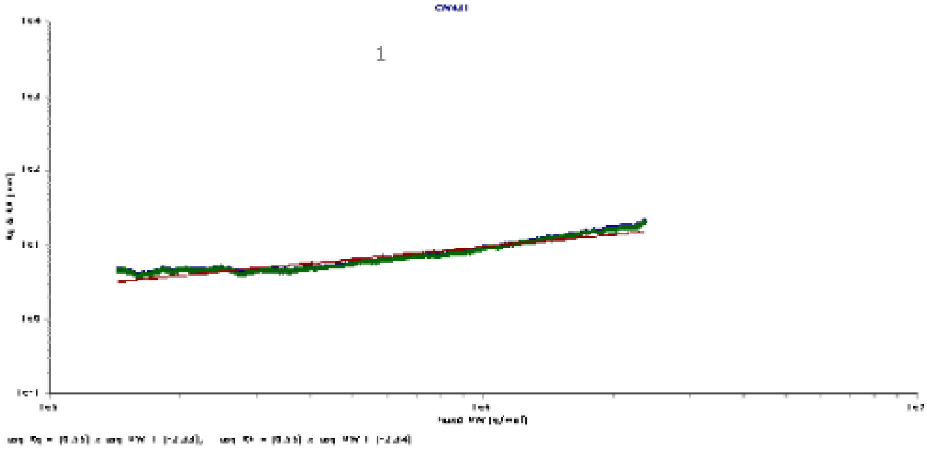

Mark-Houwink Plot

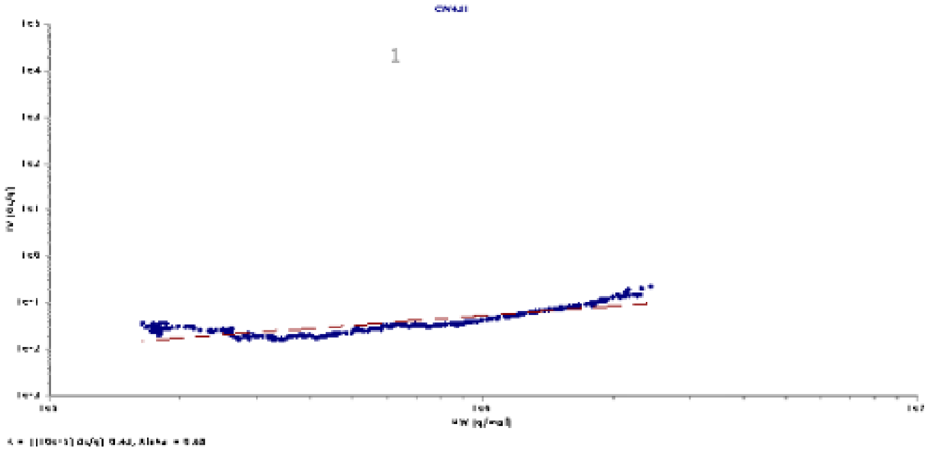

Analyst: .... **Date:** ....

Checked By: .... **Date:** ....

No solvent

Agilent GPC/SEC Software
Sample Triple Analysis Report

Agilent Technologies

CW430

Workspace Details

Workspace name	Polylactide
Location	C:\ProgramData\Agilent Technologies\GPC\Workspaces\Polylactide\
Comments	
Created by	Administrator at 13:44:31 on 15 June 2015

Sample Properties

Sample name	CW430
File name	ICF_09_07_2019-0001.sample
Collected by	GPC at 09:55:24 on 09 July 2019
Instrument name	Instrument 1

System Calibration Used

Created by	Administrator at 13:44:30 on 15 June 2015
Last modified by	Administrator at 13:44:30 on 15 June 2015
Comments	
Sample file used for calibration	ICF_6_18_2015-0002.sample
K (RI)	(RI) 750.958
K (LS 90°)	(RI) 30220.130
K (LS 15°)	(RI) 9870.135
K (VS DP)	(RI) 1.107

System IDD Used

Last modified by	Administrator at 15:52:14 on 18 June 2015
Comments	
Reference detector	LS 90°
IDD Light scatterer (secs)	0.0
IDD RI (secs)	-3.0
IDD Viscometer (secs)	-14.0

Analyst: .... **Date:** ....

Checked By: .... **Date:** ....

Agilent GPC/SEC Software
Sample Triple Analysis Report

Agilent Technologies

Processing Parameters

Method	Last modified by Administrator at 13:44:30 on 15 June 2015
Concentration Detector Used in Analysis	RI
Injection volume (µL)	100.00
Flow rate (mL/min)	1.00
Concentration options	Calculate Sample Properties from Entered Sample Concentration
Entered sample concentration (mg/mL)	1.000
Calculated dn/dc (mL/g)	0.084
Calculated Ext Coeff ([(mg/mL)-¹] [cm-¹])	0.000
MW calculation method	Use 90° angle
Log Mi-v-RT curve fit options	Set the fit limits using the limits at peak width of 10 %.
Polynomial curve fit order	1
Use Constant Inlet Pressure	No
Flory-Fox	2.86e+021
DP Multiplier (mV to Pa)	1.0000
IP Multiplier (mV to kPa)	0.1000
Use IV To Calculate Rg	Yes

MW Ranges Method

Calculate MW Ranges	No

Percentage Fractions Method

Calculate Percentage Fractions	No

Results

Analysed by	GPC at 12:58:22 on 09 July 2019
Comments	

Peak Results

	Detector Type	Peak Max RT (mins)	Bulk MW (g/mol)	Bulk IV (dL/g)	Peak Height (mV)	Peak Height (%)	Peak Area (mV.s)	Area (%)	Conc. (mg/mL)
Peak 1	RI	13.83333	27200	0.013404	1.677	100.00	380.640	100.00	1.000
Recovery (%)	100.00								

Molecular Weight Averages

Peak	Mp (g/mol)	Mn (g/mol)	Mw (g/mol)	Mz (g/mol)	Mz+1 (g/mol)	Mv (g/mol)	PD
Peak 1	12333	12655	22159	35388	46211	31097	1.751

Rg Results

Peak	Slope	Intercept	Rgp (nm)	Rgn (nm)	Rgw (nm)	Rgz (nm)	Rgz+1 (nm)
Peak 1	0.4845	-1.775	1.62	1.64	2.16	2.71	3.08

Rh Results

Peak	Slope	Intercept	Rhp (nm)	Rhn (nm)	Rhw (nm)	Rhz (nm)	Rhz+1 (nm)
Peak 1	0.4845	-1.88	1.27	1.29	1.69	2.13	2.42

Analyst: .... **Date:** ....

Checked By: .... **Date:** ....

Agilent GPC/SEC Software
Sample Triple Analysis Report

Agilent Technologies

IV Results

Peak	K ((10e-5) dL/g)	Alpha	IVp (dL/g)	IVn (dL/g)	IVw (dL/g)	IVz (dL/g)	IVz+1 (dL/g)
Peak 1	59.03	0.3105	0.010999	0.011087	0.013194	0.015258	0.016576

Peak Information

	Start (mins)	End (mins)
Baseline region 1	1.70000	4.95000
Baseline region 2	30.61667	33.18333
Peak 1	11.06667	15.36667

Peak Trace Information

Peak	Trace	Peak Max RT (mins)	Peak Area (mV.s)	Peak Height (mV)
Peak 1	RI	13.83333	380.640	1.677
Peak 1	VS DP	12.26667	78.288	0.635
Peak 1	VS IP	12.66667	29.174	0.438
Peak 1	LS 90°	12.51667	173.183	1.443
Peak 1	LS 15°	12.51667	69.431	0.525

Log Mi Curves

Peak	Start (mins)	End (mins)	Polynomial Degree	Coeff a	Coeff b	Coeff c	Coeff d	Coeff e	Coeff f	L Point Y/N
Peak 1	11.0667	15.3500	1	7.9744344	-0.28106271	0	0	0	0	N

Structural Plot Region

Peak	Start (mins)	End (mins)
Peak 1	11.0667	15.3500

Analyst: .... **Date:** ....

Checked By: .... **Date:** ....

Agilent GPC/SEC Software
Sample Triple Analysis Report

Agilent Technologies

Chromatogram Plot

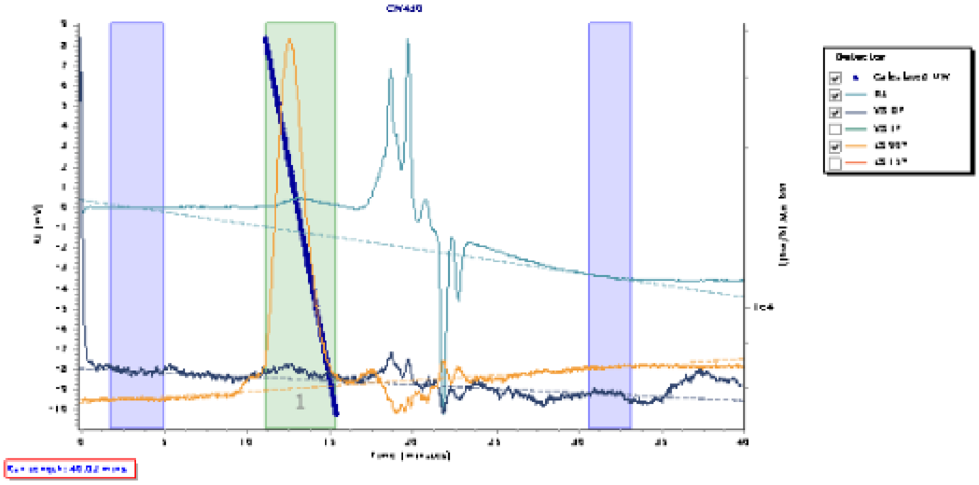

Distribution Plot

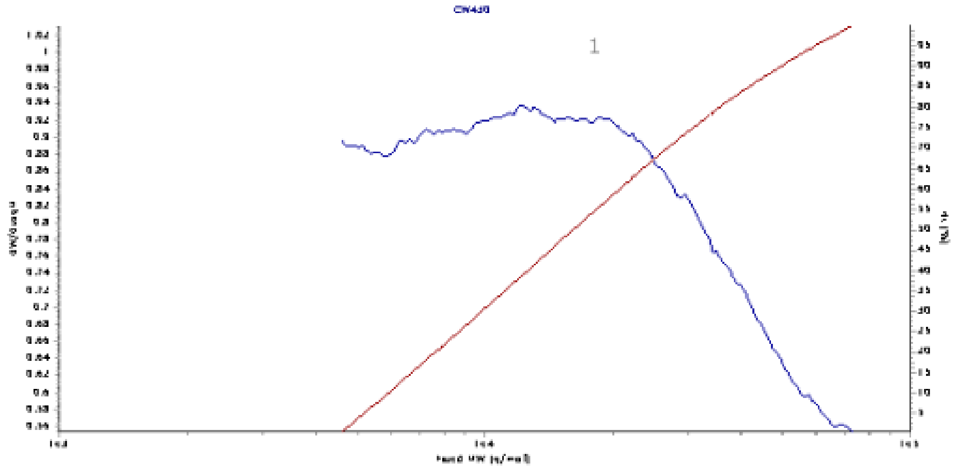

Analyst: .... **Date:** ....

Checked By: .... **Date:** ....

Agilent GPC/SEC Software
Sample Triple Analysis Report

Agilent Technologies

Conformation Plot

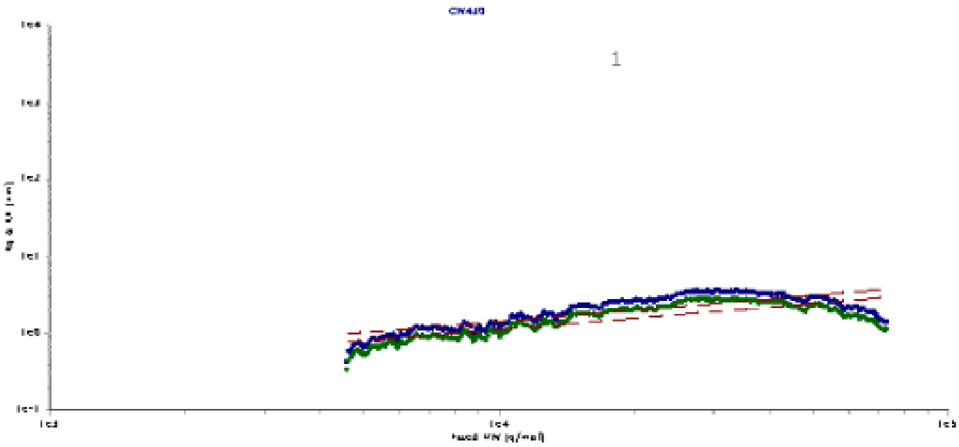

log Rg = (0.42) x log MW + (-1.77), log Rh = (0.42) x log MW + (-1.20)

Mark-Houwink Plot

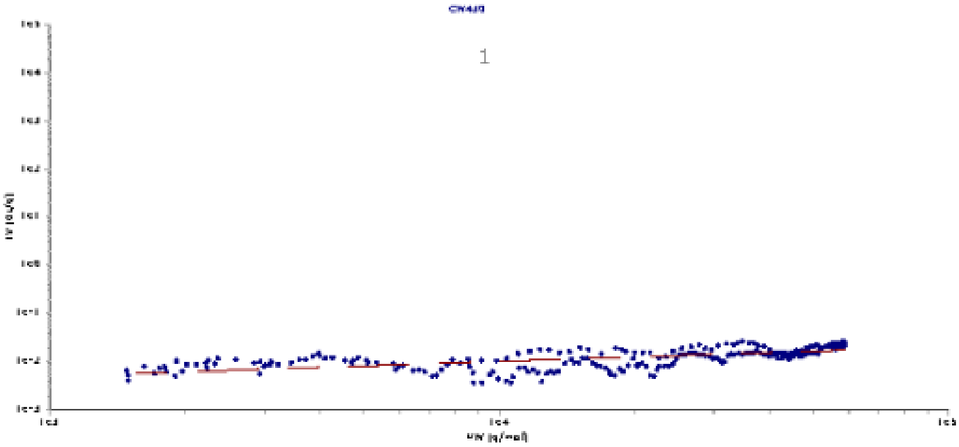

K = ((10e-5) dl/g) 59.03, Alpha = 0.31

Analyst: .... **Date:** ....

Checked By: .... **Date:** ....

Heated at 80°C

Agilent GPC/SEC Software
Sample Triple Analysis Report

Agilent Technologies

CW432

**Workspace Details**	
Workspace name	Polylactide
Location	C:\ProgramData\Agilent Technologies\GPC\Workspaces\Polylactide\
Comments	
Created by	Administrator at 13:44:31 on 15 June 2015
**Sample Properties**	
Sample name	CW432
File name	ICF_09_07_2019-0003.sample
Collected by	GPC at 11:19:08 on 09 July 2019
Instrument name	Instrument 1
**System Calibration Used**	
Created by	Administrator at 13:44:30 on 15 June 2015
Last modified by	Administrator at 13:44:30 on 15 June 2015
Comments	
Sample file used for calibration	ICF_6_18_2015-0002.sample
K (RI)	(RI) 750.958
K (LS 90°)	(RI) 30220.130
K (LS 15°)	(RI) 9870.135
K (VS DP)	(RI) 1.107
**System IDD Used**	
Last modified by	Administrator at 15:52:14 on 18 June 2015
Comments	
Reference detector	LS 90°
IDD Light scatterer (secs)	0.0
IDD RI (secs)	-3.0
IDD Viscometer (secs)	-14.0

Analyst: .... **Date:** ....

Checked By: .... **Date:** ....

Agilent GPC/SEC Software
Sample Triple Analysis Report

Agilent Technologies

Processing Parameters

Method	Last modified by Administrator at 13:44:30 on 15 June 2015
Concentration Detector Used in Analysis	RI
Injection volume (µL)	100.00
Flow rate (mL/min)	1.00
Concentration options	Calculate Sample Properties from Entered Sample Concentration
Entered sample concentration (mg/mL)	1.000
Calculated dn/dc (mL/g)	0.042
Calculated Ext Coeff ([(mg/mL)-¹] [cm-¹])	0.000
MW calculation method	Use 90° angle
Log Mi-v-RT curve fit options	Set the fit limits using the limits at peak width of 10 %.
Polynomial curve fit order	1
Use Constant Inlet Pressure	No
Flory-Fox	2.86e+021
DP Multiplier (mV to Pa)	1.0000
IP Multiplier (mV to kPa)	0.1000
Use IV To Calculate Rg	Yes

MW Ranges Method

Calculate MW Ranges	No

Percentage Fractions Method

Calculate Percentage Fractions	No

Results

Analysed by	GPC at 13:03:53 on 09 July 2019
Comments	

Peak Results

	Detector Type	Peak Max RT (mins)	Bulk MW (g/mol)	Bulk IV (dL/g)	Peak Height (mV)	Peak Height (%)	Peak Area (mV.s)	Area (%)	Conc. (mg/mL)
Peak 1	RI	13.68333	223625	0.030541	1.097	100.00	188.735	100.00	1.000
Recovery (%)	100.00								

Molecular Weight Averages

Peak	Mp (g/mol)	Mn (g/mol)	Mw (g/mol)	Mz (g/mol)	Mz+1 (g/mol)	Mv (g/mol)	PD
Peak 1	193503	120865	223332	366032	517685	357349	1.848

Rg Results

Peak	Slope	Intercept	Rgp (nm)	Rgn (nm)	Rgw (nm)	Rgz (nm)	Rgz+1 (nm)
Peak 1	0.6546	-2.868	3.91	2.87	4.30	5.94	7.45

Rh Results

Peak	Slope	Intercept	Rhp (nm)	Rhn (nm)	Rhw (nm)	Rhz (nm)	Rhz+1 (nm)
Peak 1	0.6546	-2.832	4.25	3.13	4.67	6.46	8.10

Analyst: .... **Date:** ....

Checked By: .... **Date:** ....

Agilent GPC/SEC Software
Sample Triple Analysis Report

Agilent Technologies

IV Results

Peak	K ((10e-5) dL/g)	Alpha	IVp (dL/g)	IVn (dL/g)	IVw (dL/g)	IVz (dL/g)	IVz+1 (dL/g)
Peak 1	0.0587	0.8786	0.025906	0.017133	0.029384	0.045354	0.061502

Peak Information

	Start (mins)	End (mins)
Baseline region 1	4.18333	6.68333
Baseline region 2	30.50000	33.65000
Peak 1	11.21667	16.30000

Peak Trace Information

Peak	Trace	Peak Max RT (mins)	Peak Area (mV.s)	Peak Height (mV)
Peak 1	RI	13.68333	188.735	1.097
Peak 1	VS DP	12.80000	178.182	1.403
Peak 1	VS IP	15.20000	4.456	-0.394
Peak 1	LS 90°	12.81667	350.057	2.631
Peak 1	LS 15°	12.81667	112.899	0.844

Log Mi Curves

Peak	Start (mins)	End (mins)	Polynomial Degree	Coeff a	Coeff b	Coeff c	Coeff d	Coeff e	Coeff f	L Point Y/N
Peak 1	11.6167	16.2833	1	9.6633297	-0.31985204	0	0	0	0	N

Structural Plot Region

Peak	Start (mins)	End (mins)
Peak 1	11.6167	16.2833

Analyst: .... **Date:** ....

Checked By: .... **Date:** ....

Agilent GPC/SEC Software
Sample Triple Analysis Report

Agilent Technologies

Chromatogram Plot

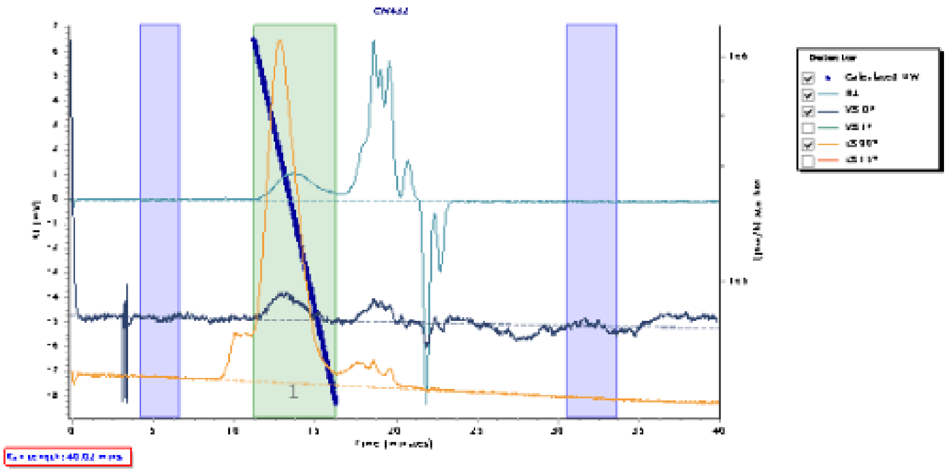

Distribution Plot

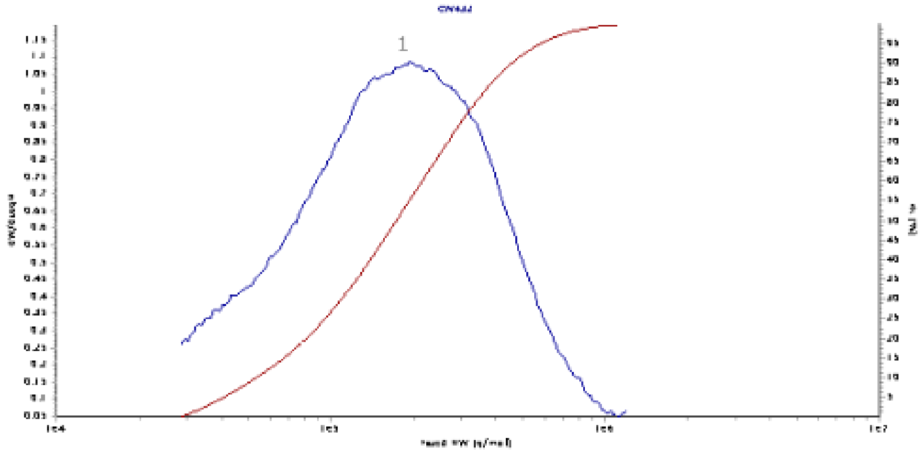

Analyst: .... **Date:** ....

Checked By: .... **Date:** ....

Agilent GPC/SEC Software
Sample Triple Analysis Report

Agilent Technologies

Conformation Plot

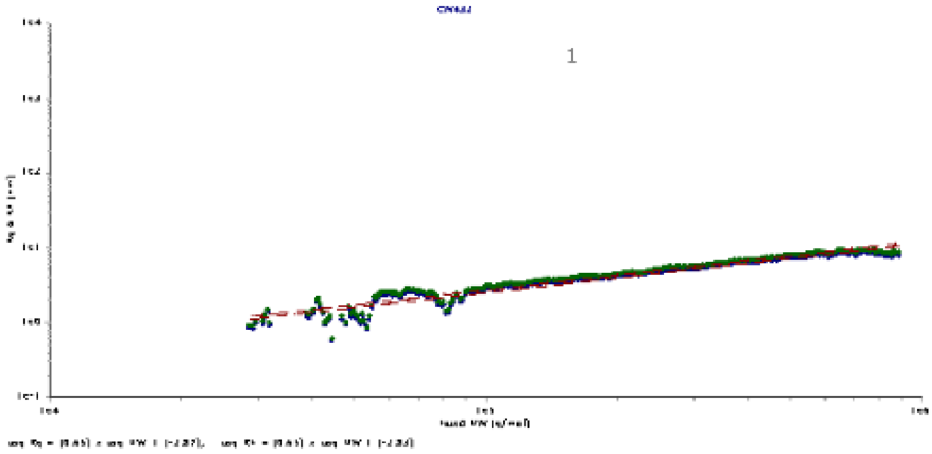

Mark-Houwink Plot

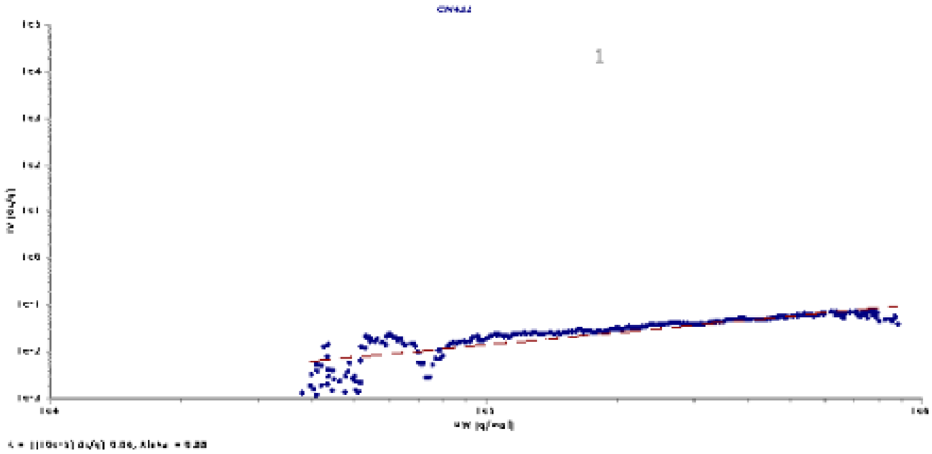

Analyst: .... **Date:** ....

Checked By: .... **Date:** ....

Quenched after 5 min

Agilent GPC/SEC Software
Sample Triple Analysis Report

Agilent Technologies

cw534_b

**Workspace Details**	
Workspace name	Polylactide
Location	C:\ProgramData\Agilent Technologies\GPC\Workspaces\Polylactide\
Comments	
Created by	Administrator at 13:44:31 on 15 June 2015
**Sample Properties**	
Sample name	cw534_b
File name	ICF_04_11_2019-0009.sample
Collected by	GPC at 15:10:19 on 04 November 2019
Instrument name	Instrument 1
**System Calibration Used**	
Created by	Administrator at 13:44:30 on 15 June 2015
Last modified by	Administrator at 13:44:30 on 15 June 2015
Comments	
Sample file used for calibration	ICF_6_18_2015-0002.sample
K (RI)	(RI) 750.958
K (LS 90°)	(RI) 30220.130
K (LS 15°)	(RI) 9870.135
K (VS DP)	(RI) 1.107
**System IDD Used**	
Last modified by	Administrator at 15:52:14 on 18 June 2015
Comments	
Reference detector	LS 90°
IDD Light scatterer (secs)	0.0
IDD RI (secs)	-3.0
IDD Viscometer (secs)	-14.0

Analyst: .... **Date:** ....

Checked By: .... **Date:** ....

Agilent GPC/SEC Software A.02.01 [9] Page 1 of 5

Generated by GPC at 14:42 on 16 September 2020

Agilent GPC/SEC Software
Sample Triple Analysis Report

Agilent Technologies

Processing Parameters

Method	Last modified by Administrator at 13:44:30 on 15 June 2015
Concentration Detector Used in Analysis	RI
Injection volume (µL)	100.00
Flow rate (mL/min)	1.00
Concentration options	Calculate Sample Properties from Entered Sample Concentration
Entered sample concentration (mg/mL)	1.000
Calculated dn/dc (mL/g)	0.168
Calculated Ext Coeff ([(mg/mL)-¹] [cm-¹])	0.000
MW calculation method	Use all angles
Log Mi-v-RT curve fit options	Set the fit limits using the limits at peak width of 10 %.
Polynomial curve fit order	1
Use Constant Inlet Pressure	No
Flory-Fox	2.86e+021
DP Multiplier (mV to Pa)	1.0000
IP Multiplier (mV to kPa)	0.1000
Use IV To Calculate Rg	No

MW Ranges Method

Calculate MW Ranges — No

Percentage Fractions Method

Calculate Percentage Fractions — No

Results

Analysed by — GPC at 10:24:16 on 06 November 2019

Comments

Peak Results

	Detector Type	Peak Max RT (mins)	Bulk MW (g/mol)	Bulk IV (dL/g)	Peak Height (mV)	Peak Height (%)	Peak Area (mV.s)	Area (%)	Conc. (mg/mL)
Peak 1	RI	18.08333	28806	0.090335	3.071	8.84	81.475	10.75	0.108
Peak 2	RI	18.76667	570	0.026365	31.686	91.16	676.407	89.25	0.892
Recovery (%)	100.00								

Molecular Weight Averages

Peak	Mp (g/mol)	Mn (g/mol)	Mw (g/mol)	Mz (g/mol)	Mz+1 (g/mol)	Mv (g/mol)	PD
Peak 1	5951	10455	13923	19292	25019	16659	1.332
Peak 2	967	950	965	979	995	973	1.015

Rg Results

Peak	Slope	Intercept	Rgp (nm)	Rgn (nm)	Rgw (nm)	Rgz (nm)	Rgz+1 (nm)
Peak 2	-0.9597	4.63	58.19	59.21	58.34	57.47	56.61

Rh Results

Peak	Slope	Intercept	Rhp (nm)	Rhn (nm)	Rhw (nm)	Rhz (nm)	Rhz+1 (nm)
Peak 1	0.5187	-1.711	1.73	2.36	2.76	3.31	3.82
Peak 2	0.4	-1.3	0.77	0.77	0.77	0.78	0.78

Analyst: .... **Date:** ....

Checked By: .... **Date:** ....

Agilent GPC/SEC Software
Sample Triple Analysis Report

Agilent Technologies

IV Results

Peak	K ((10e-5) dL/g)	Alpha	IVp (dL/g)	IVn (dL/g)	IVw (dL/g)	IVz (dL/g)	IVz+1 (dL/g)
Peak 1	4665	0.07886	0.092588	0.096795	0.099007	0.101587	0.103691
Peak 2	1217	0.1379	0.031401	0.031324	0.031389	0.031456	0.031523

Peak Information

	Start (mins)	End (mins)
Baseline region 1	4.33333	6.51667
Baseline region 2	25.86667	28.30000
Peak 1	17.15000	18.08333
Peak 2	18.38333	19.10000

Peak Trace Information

Peak	Trace	Peak Max RT (mins)	Peak Area (mV.s)	Peak Height (mV)
Peak 1	RI	18.08333	81.475	3.071
Peak 1	VS DP	18.06667	56.553	1.454
Peak 1	VS IP	17.26667	3.907	0.418
Peak 1	LS 90°	17.61667	97.689	4.856
Peak 1	LS 15°	17.63333	25.705	1.421
Peak 2	RI	18.76667	676.407	31.686
Peak 2	VS DP	18.76667	137.052	5.063
Peak 2	VS IP	18.73333	6.117	0.472
Peak 2	LS 90°	18.76667	19.443	0.740
Peak 2	LS 15°	19.10000	4.242	-0.199

Log Mi Curves

Peak	Start (mins)	End (mins)	Polynomial Degree	Coeff a	Coeff b	Coeff c	Coeff d	Coeff e	Coeff f	L Point Y/N
Peak 1	17.1500	18.0833	1	20.368361	-0.91847342	0	0	0	0	N
Peak 2	18.3833	19.0833	1	9.313952	-0.33752158	0	0	0	0	N

Structural Plot Region

Peak	Start (mins)	End (mins)
Peak 1	17.1500	18.0833
Peak 2	18.3833	19.0833

Analyst: .... **Date:** ....

Checked By: .... **Date:** ....

Agilent GPC/SEC Software
Sample Triple Analysis Report

Agilent Technologies

Chromatogram Plot

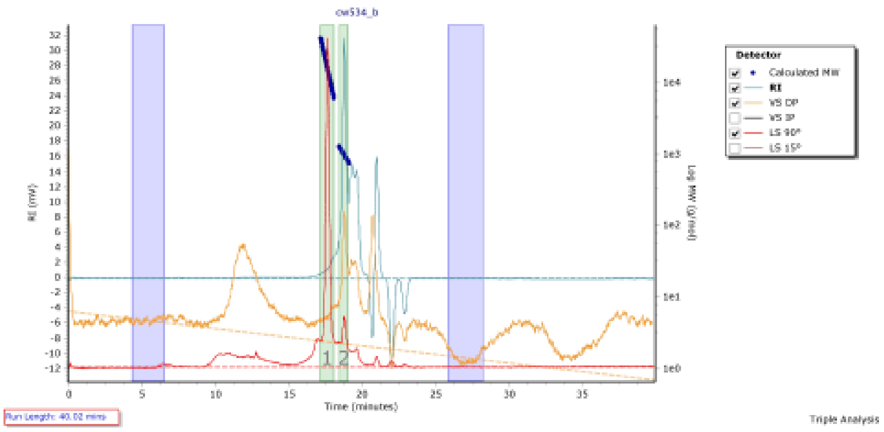

Distribution Plot

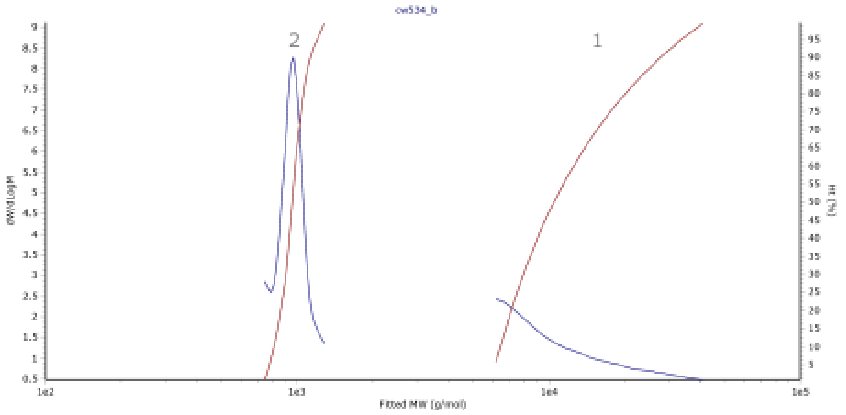

Analyst: .... **Date:** ....

Checked By: .... **Date:** ....

Agilent GPC/SEC Software A.02.01 [9] Page 4 of 5

Generated by GPC at 14:42 on 16 September 2020

Agilent GPC/SEC Software
Sample Triple Analysis Report

Agilent Technologies

Conformation Plot

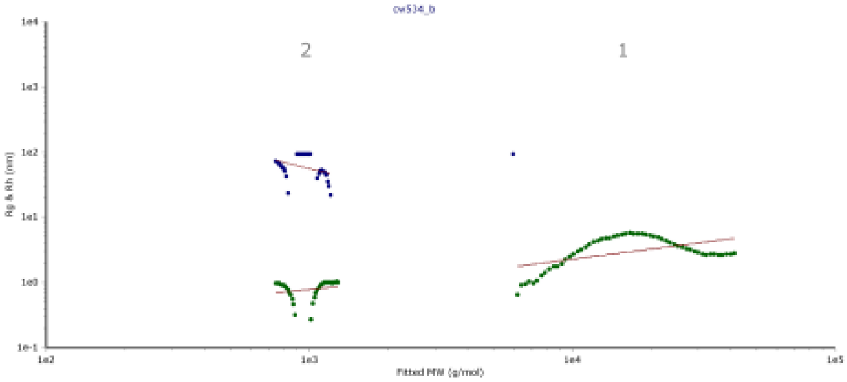

Peak 1 Rh (VS): Log Rh = (0.52) x Log MW + (-1.71)
Peak 2 Rg (LS): Log Rg = (-0.96) x Log MW + (4.63), Log Rh = (0.40) x Log MW + (-1.30)

Mark-Houwink Plot

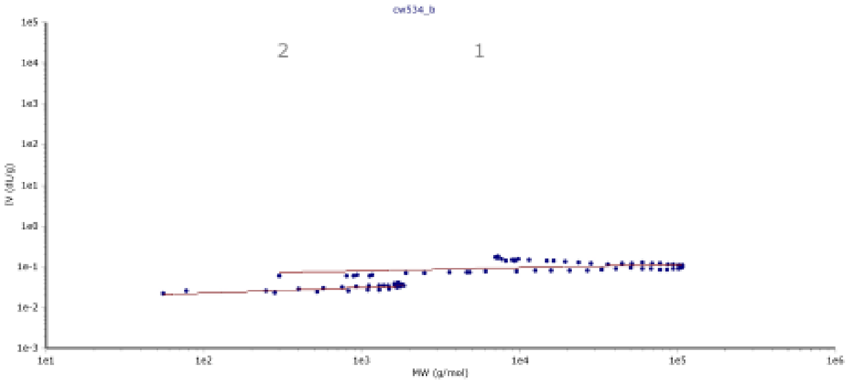

Peak 1: K = ((10e-5) dL/g) 4665.30, Alpha = 0.08
Peak 2: K = ((10e-5) dL/g) 1217.18, Alpha = 0.14

Analyst: .... **Date:** ....

Checked By: .... **Date:** ....

20 mol% catalyst loading

Agilent GPC/SEC Software
Sample Triple Analysis Report

Agilent Technologies

cw646

**Workspace Details**	
Workspace name	Polylactide
Location	C:\ProgramData\Agilent Technologies\GPC\Workspaces\Polylactide\
Comments	
Created by	Administrator at 13:44:31 on 15 June 2015
**Sample Properties**	
Sample name	cw646
File name	ICF_06_08_2020-0002.sample
Collected by	GPC at 10:31:30 on 06 August 2020
Instrument name	Instrument 1
**System Calibration Used**	
Created by	Administrator at 13:44:30 on 15 June 2015
Last modified by	Administrator at 13:44:30 on 15 June 2015
Comments	
Sample file used for calibration	ICF_6_18_2015-0002.sample
K (RI)	(RI) 750.958
K (LS 90°)	(RI) 30220.130
K (LS 15°)	(RI) 9870.135
K (VS DP)	(RI) 1.107
**System IDD Used**	
Last modified by	Administrator at 15:52:14 on 18 June 2015
Comments	
Reference detector	LS 90°
IDD Light scatterer (secs)	0.0
IDD RI (secs)	-3.0
IDD Viscometer (secs)	-14.0

Analyst: .... **Date:** ....

Checked By: .... **Date:** ....

Agilent GPC/SEC Software A.02.01 [9] Page 1 of 5

Generated by GPC at 11:31 on 14 August 2020

Agilent GPC/SEC Software
Sample Triple Analysis Report

Agilent Technologies

Processing Parameters

Method	Last modified by Administrator at 13:44:30 on 15 June 2015
Concentration Detector Used in Analysis	RI
Injection volume (µL)	100.00
Flow rate (mL/min)	1.00
Concentration options	Calculate Sample Properties from Entered Sample Concentration
Entered sample concentration (mg/mL)	1.000
Calculated dn/dc (mL/g)	0.016
Calculated Ext Coeff ([(mg/mL)-¹] [cm-¹])	0.000
MW calculation method	Use all angles
Log Mi-v-RT curve fit options	Set the fit limits using the limits at peak width of 10 %.
Polynomial curve fit order	1
Use Constant Inlet Pressure	No
Flory-Fox	2.86e+021
DP Multiplier (mV to Pa)	1.0000
IP Multiplier (mV to kPa)	0.1000
Use IV To Calculate Rg	No

MW Ranges Method

Calculate MW Ranges — No

Percentage Fractions Method

Calculate Percentage Fractions — No

Results

Analysed by — GPC at 11:30:52 on 14 August 2020

Comments

Peak Results

	Detector Type	Peak Max RT (mins)	Bulk MW (g/mol)	Bulk IV (dL/g)	Peak Height (mV)	Peak Height (%)	Peak Area (mV.s)	Area (%)	Conc. (mg/mL)
Peak 1	RI	10.68333	8683998	0.083867	0.280	37.31	32.414	43.83	0.438
Peak 2	RI	13.16667	1201161	0.024207	0.471	62.69	41.542	56.17	0.562
Recovery (%)	100.00								

Molecular Weight Averages

Peak	Mp (g/mol)	Mn (g/mol)	Mw (g/mol)	Mz (g/mol)	Mz+1 (g/mol)	Mv (g/mol)	PD
Peak 1	6365732	6079872	6102220	6124755	6147409	6118611	1.004
Peak 2	1210451	1073570	1203961	1343469	1475945	1289881	1.121

Rg Results

Peak	Slope	Intercept	Rgp (nm)	Rgn (nm)	Rgw (nm)	Rgz (nm)	Rgz+1 (nm)
Peak 1	7.5	-49.48	35.24	24.97	25.66	26.38	27.12
Peak 2	-0.6597	5.273	18.21	19.72	18.28	17.00	15.98

Analyst: .... **Date:** ....

Checked By: .... **Date:** ....

Agilent GPC/SEC Software
Sample Triple Analysis Report

Agilent Technologies

Rh Results

Peak	Slope	Intercept	Rhp (nm)	Rhn (nm)	Rhw (nm)	Rhz (nm)	Rhz+1 (nm)
Peak 1	0.9965	-5.482	19.88	18.99	19.06	19.13	19.20
Peak 2	0.4031	-1.562	7.76	7.40	7.75	8.10	8.41

IV Results

Peak	K ((10e-5) dL/g)	Alpha	IVp (dL/g)	IVn (dL/g)	IVw (dL/g)	IVz (dL/g)	IVz+1 (dL/g)
Peak 1	5.887	0.4562	0.074795	0.073243	0.073366	0.073489	0.073613
Peak 2	107.9	0.2227	0.024407	0.023764	0.024378	0.024981	0.025509

Peak Information

	Start (mins)	End (mins)
Baseline region 1	3.88333	7.18333
Baseline region 2	30.10000	32.20000
Peak 1	9.18333	11.91667
Peak 2	12.05000	13.76667

Peak Trace Information

Peak	Trace	Peak Max RT (mins)	Peak Area (mV.s)	Peak Height (mV)
Peak 1	RI	10.68333	32.414	0.280
Peak 1	VS DP	10.13333	213.602	2.840
Peak 1	VS IP	10.01667	7.004	0.342
Peak 1	LS 90°	10.15000	796.530	11.450
Peak 1	LS 15°	10.11667	297.287	4.380
Peak 2	RI	13.16667	41.542	0.471
Peak 2	VS DP	12.90000	79.013	1.122
Peak 2	VS IP	12.06667	6.009	0.344
Peak 2	LS 90°	12.05000	155.019	2.098
Peak 2	LS 15°	12.15000	52.884	0.714

Log Mi Curves

Peak	Start (mins)	End (mins)	Polynomial Degree	Coeff a	Coeff b	Coeff c	Coeff d	Coeff e	Coeff f	L Point Y/N
Peak 1	9.1833	11.9000	1	7.2054625	-0.039181874	0	0	0	0	N
Peak 2	12.0500	13.7500	1	10.133075	-0.31559438	0	0	0	0	N

Structural Plot Region

Peak	Start (mins)	End (mins)
Peak 1	9.1833	11.9000
Peak 2	12.0500	13.7500

Analyst: .... **Date:** ....

Checked By: .... **Date:** ....

Agilent GPC/SEC Software
Sample Triple Analysis Report

Agilent Technologies

Chromatogram Plot

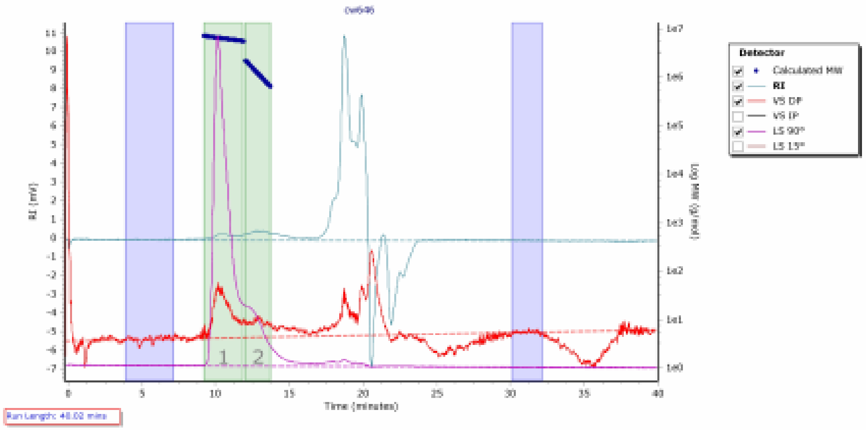

Distribution Plot

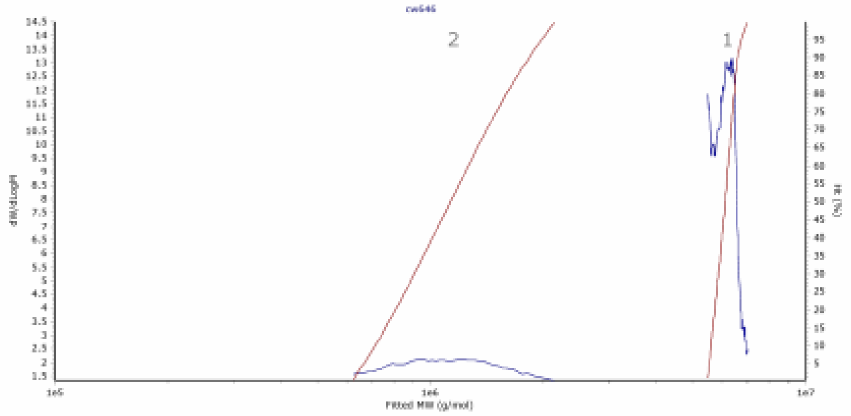

Analyst: .... **Date:** ....

Checked By: .... **Date:** ....

Agilent GPC/SEC Software A.02.01 [9] Page 4 of 5

Generated by GPC at 11:31 on 14 August 2020

Agilent GPC/SEC Software
Sample Triple Analysis Report

Agilent Technologies

Conformation Plot

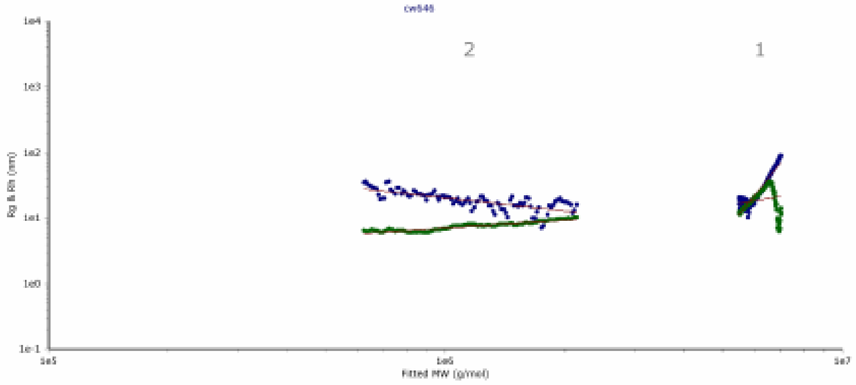

Peak 1 Rg (LS): Log Rg = (7.50) x Log MW + (-49.48), Log Rh = (1.00) x Log MW + (-5.48)
Peak 2 Rg (LS): Log Rg = (-0.66) x Log MW + (5.27), Log Rh = (0.40) x Log MW + (-1.56)

Mark-Houwink Plot

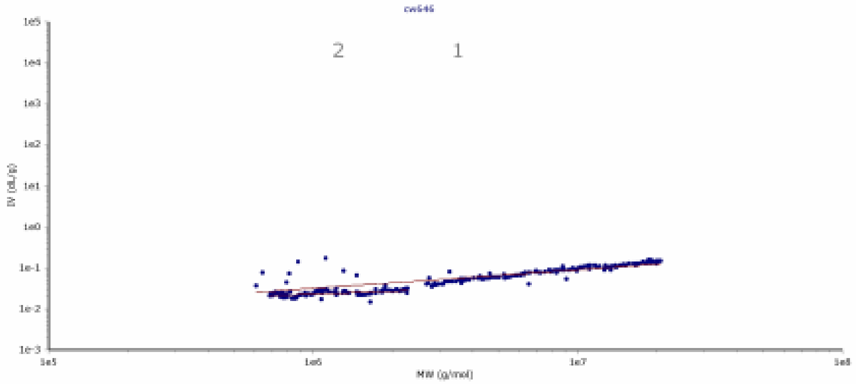

Peak 1 : K = ((10e-5) dL/g) 5.89, Alpha = 0.46
Peak 2: K = ((10e-5) dL/g) 107.87, Alpha = 0.22

Analyst: .... **Date:** ....

Checked By: .... **Date:** ....

Agilent GPC/SEC Software A.02.01 [9]

Generated by GPC at 11:31 on 14 August 2020

Computational Details

The seven-unit oligomer was chosen as a reasonable model to computationally evaluate the stability of the isomers of the allene polymer, as the structures (124 atoms) were a good balance between being able to model longer-range Van der Waals forces/non-covalent interactions, while also not being too computationally expensive to calculate. A conformational search was performed with PCM10 using the standard MMFF94 forcefield, with a minimum of 500 conformers screened and a maximum of 20 duplicates or 1000 conformers screened.

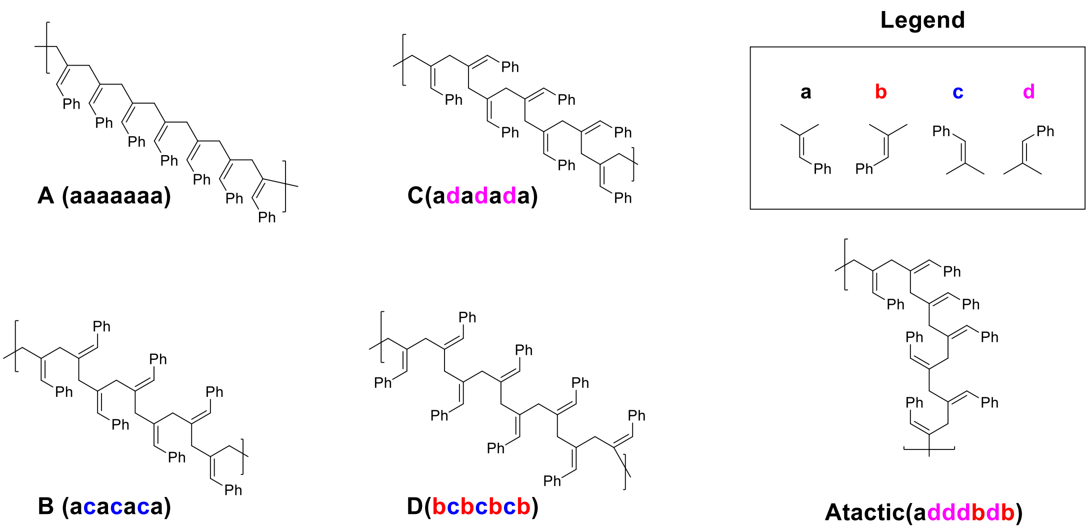

Figure S1: Structures of the five species identified as low-energy conformers of the seven-unit allene oligomer model using an MMFF94 conformational search.

Four species (**A-D**) featuring repeating units were identified as unique stereoisomers, as well as one atactic isomer (**Atactic**) which was identified as a low-energy conformer (see Figure S1). Density functional theory optimisations of these structures were performed using Jaguar 8.5 and the standard B3LYP hybrid density functional with Grimme's D3 dispersion correction. Atoms were modelled using the standard Pople basis set 6-31G(d), solvation was modelled using a polar continuum model with the solvent as benzene, and frequency calculations were carried out at 298.15 K to confirm species were intermediates, with additional thermochemistry corrections calculated at 323.15, 358.15 and 373.15 K to model the effect of reaction temperature on the relative populations of the different species. Convergence criteria were set to five times the standard for Jaguar, as no material changes in optimised geometry were observed when using the more computationally expensive criteria for test calculations. All calculations were run with the standard medium-fine grid density in Jaguar.

Single point potential energies (s.p.e's) were then calculated with the 6-311+G(d,p) basis set using the optimised geometries and the same DFT approach. A trial optimisation of

Conformer B using the B3LYP-D3/6-311+g(d,p) theory level revealed no notable structural changes to that observed using 6-31G(d).

Population distributions for the five species were calculated by combining these single point potential energies and the previously calculated free energy corrections at each temperature (see *Equation 1*) using the Maxwell-Boltzmann distribution (*Equation 2*), shown in Tables S1 & S2.

Equation 1: Calculation of Gibbs free energies using mixed basis set calculations:

$$\Delta G_{mixed} = \Delta E_{6-311+G**} + (\Delta G_{6-31G*} - \Delta E_{6-31G*})$$

Equation 2: The Maxwell Boltzmann distribution, where N_i is the expected number of particles in a given state, N is the total number of particles in a system, E_i is the energy of microstate *i*, the sum over index *j* takes into account all states considered, T is the temperature, and *k* is the Boltzmann constant.

$$\frac{N_i}{N} = \frac{\exp\left(-\frac{E_i}{kT}\right)}{\sum_j \exp\left(-\frac{E_j}{kT}\right)}$$

Table S1: Calculated relative free energies used for Maxwell-Boltzmann population distributions.

**Species**	**Relative ΔG (6-31G* 298K) (kcal mol-1)**	**(s.p.e + ΔG Correction @ 298.15 K)**	**(s.p.e + ΔG Correction @ 323.15 K)**	**(s.p.e + ΔG Correction @ 358.15 K)**	**(s.p.e + ΔG Correction @ 373.15 K)**
**A**	8.88	6.46	6.54	6.63	6.73
**B**	0.00	0.00	0.00	0.00	0.00
**C**	2.73	1.76	1.95	2.14	2.33
**D**	6.49	5.82	5.84	5.87	5.90
**Atactic**	6.12	4.43	4.77	5.13	5.49

Table S2: Population distributions calculated using the Maxwell-Boltzmann distribution.

Species	% Population 298.15 K	% Population 323.15 K	% Population 358.15 K	% Population 373.15 K
**A**	0.00	0.00	0.01	0.01
**B**	95.09	95.32	95.16	95.16
**C**	4.85	4.61	4.73	4.09
**D**	0.01	0.01	0.02	0.03
**Atactic**	0.05	0.06	0.07	0.06

The calculated populations at each temperature do not meaningful change in any way we would expect to be significant in experimental determinations of polymer composition for the four repeating unit structures over the 75 K range (298.15 – 373.15 K). While the atactic oligomer is observed to rise in relative energy (4.43 kcal mol^{-1} at 298.15 K to 5.49 kcal mol^{-1} at 373.15 K), this is likely due to additional degrees of freedom in this structure producing a greater entropic response over the temperature range. The atactic species is also not likely to produce a good model of the long-range interactions of the experimentally observed product polymer, as the lack of uniformity or repeating structure means it is not possible to estimate or extrapolate the relative stability of this species outside of our seven-unit oligomer model. This not the case for the repeating unit isomers **A-D**, where the repeating structure can, presumably, give us a reasonable estimate of their relative stability in larger systems which are simply extensions of the same repeating structure we have modelled with the seven-unit oligomer.

References

1. K, K.A., et al., *Facile, Catalytic Dehydrocoupling of Phosphines Using β-Diketiminate Iron(II) Complexes.* Chemistry – A European Journal. **21**(45): p. 15960-15963.
2. Gasperini, D., et al., *Seeking Heteroatom-Rich Compounds: Synthetic and Mechanistic Studies into Iron Catalyzed Dehydrocoupling of Silanes.* ACS Catalysis, 2020.
3. Spitzer, F., et al., *Influence of the nacnac Ligand in Iron(I)-Mediated P4 Transformations.* Angewandte Chemie International Edition, 2016. **55**(13): p. 4340-4344.
4. Woof, C.R., et al., *Iron catalyzed double bond isomerization: evidence for an Fe(I)/Fe(III) catalytic cycle.* Chemistry – A European Journal. **n/a**(n/a).
5. Sonnenberg, J.F. and R.H. Morris, *Distinguishing homogeneous from nanoparticle asymmetric iron catalysis.* Catal. Sci. Technol., 2014. **4**(10): p. 3426-3438.
6. Espinal-Viguri, M., et al., *Hydrophosphination of Unactivated Alkenes and Alkynes Using Iron(II): Catalysis and Mechanistic Insight.* ACS Catalysis, 2016. **6**(11): p. 7892-7897.
7. Clavier, H., et al., *Highly Selective Cobalt-Mediated [6 + 2] Cycloaddition of Cycloheptatriene and Allenes.* Organic Letters, 2011. **13**(2): p. 308-311.
8. Coles, N.T., M.F. Mahon, and R.L. Webster, *Phosphine- and Amine-Borane Dehydrocoupling Using a Three-Coordinate Iron(II) β-Diketiminate Precatalyst.* Organometallics, 2017. **36**(11): p. 2262-2268.
9. Zhao, Z., L. Racicot, and G.K. Murphy, *Fluorinative Rearrangements of Substituted Phenylallenes Mediated by (Difluoroiodo)toluene: Synthesis of α-(Difluoromethyl)styrenes.* Angewandte Chemie International Edition, 2017. **56**(38): p. 11620-11623.
